# Clinicopathologic Analysis of Sarcomas in the Oral and Maxillofacial Region: A Systematic Review

**DOI:** 10.1111/odi.70103

**Published:** 2025-09-30

**Authors:** Iara Vieira Ferreira, Reydson Alcides de Lima‐Souza, Talita de Carvalho Kimura, Alfio José Tincani, Marcelo Elias Schempf Cattan, Arthur Antolini‐Tavares, Albina Altemani, Fernanda Viviane Mariano

**Affiliations:** ^1^ Oral Diagnosis Department Piracicaba Dental School, State University of Campinas (UNICAMP) Piracicaba, São Paulo Brazil; ^2^ Pathology Department School of Medical Sciences, State University of Campinas (UNICAMP) Campinas, São Paulo Brazil; ^3^ Head and Neck Surgery Department School of Medical Sciences Campinas, São Paulo Brazil

**Keywords:** jaw neoplasms, mouth neoplasms, oral and maxillofacial, sarcoma, systematic review

## Abstract

**Objective:**

This study aimed to systematically review primary sarcomas in the oral and maxillofacial region, focusing on patient demographics and sarcoma‐specific characteristics, including clinical presentation, histopathology, treatment approaches, outcomes, and survival rates.

**Materials and Methods:**

A systematic review was conducted in accordance with PRISMA 2020 guidelines and based on the PECOS framework, including observational studies on primary oral and maxillofacial sarcomas. An electronic search in five databases identified eligible studies, and outcomes were analyzed via Kaplan–Meier and Cox regression.

**Results:**

The review included 35 studies comprising 687 cases of sarcomas in the oral and maxillofacial region. The mean patient age was 35.26 years, with a slight male predominance. Lesions predominantly involved the mandible, with osteosarcoma being the most common histological subtype. Multimodal treatment was most frequent. Of 616 patients with survival data, 58.1% were alive at analysis. The overall 5‐year survival and disease‐free survival rates were 54.3% and 60.4%, respectively. Factors such as age, histological type, T classification, stage grouping, surgical margins, local recurrence, and distant metastases significantly influenced survival (*p* < 0.05).

**Conclusion:**

This study provides a comprehensive review of oral and maxillofacial sarcomas, offering data to understand the clinicopathological characteristics of these lesions, helping to improve their diagnosis and management.

## 1 Introduction

Sarcomas are rare and heterogeneous solid tumors derived from mesenchymal progenitor cells. They can originate in both soft tissues, such as muscle, fat, blood vessels, neural tissue, and cartilage, and hard tissues, such as bone (de Carvalho et al. 2020; Kotecha et al. 2021). The current World Health Organization (WHO) classification of soft tissue and bone tumors divides sarcomas into three main groups: soft tissue sarcomas, bone sarcomas, and undifferentiated round cell sarcomas. This classification includes more than 50 distinct histologic subtypes (WHO 2020).

These mesenchymal tumors most commonly affect the extremities, accounting for 12%–28% of all sarcoma cases, followed by the abdominal viscera, which represent about 22% of sarcomas. Sarcomas in the head and neck region are less common, comprising approximately 5%–15% of all sarcoma diagnoses (Tran et al. 1992; Hui 2016; Mannelli et al. 2024). Oral sarcomas are even rarer, representing about 1% of all malignancies found in this anatomical region (Alishahi et al. 2015; de Carvalho et al. 2020).

Despite their rarity, sarcomas of the oral and maxillofacial region are associated with high morbidity and mortality rates, posing significant challenges in the management of these neoplasms (Kumar et al. 2019). Although several systematic reviews have addressed specific sarcoma subtypes or focused on sarcomas of the head and neck region (Andersen et al. 2019; de Souza et al. 2020; Coca‐Pelaz et al. 2021; Rodríguez‐Vargas and Villanueva‐Sánchez 2022; Houpe et al. 2023; Spiguel et al. 2024; Mannelli et al. 2024), to the best of our knowledge, no systematic review has been performed to synthesize the available data on oral and maxillofacial sarcomas. Therefore, the aim of this study is to comprehensively evaluate the clinicopathologic characteristics, therapeutic approaches, and survival rates associated with this condition. The guiding question of this review is: “What is the clinicopathologic profile and survival outcomes of primary sarcomas of the oral and maxillofacial region?”

## 2 Materials and Methods

### 2.1 Information Sources and Search Strategies

Electronic searches were conducted in June 2024 and updated in June 2025, with no restrictions on publication date or language, across the following databases: MEDLINE via PubMed, Web of Science, Scopus, Embase, and LILACS. The search strategy was conducted comprehensively, including all sarcomas located in the oral and maxillofacial region, without performing specific searches for each tumor subtype individually. Additionally, gray literature was searched in Google Scholar, Open Gray, and ProQuest. The search strategy is detailed in Table S1. Manual searches were also performed by cross‐referencing the reference lists of the included articles to identify additional publications that may have been missed during the electronic searches. The retrieved studies were imported into the reference manager Rayyan (Ouzzani et al. 2016), where duplicate references were removed.

### 2.2 Eligibility Criteria

The PECOS acronym (Population, Exposure, Comparison, Outcomes, and Study Design) was adapted to guide the formulation of the systematic review question. The following criteria were defined: P: Patients diagnosed with primary sarcoma in the oral and maxillofacial region; E: Diagnosis of sarcoma by histopathologic examination; C: Not applicable; O: Clinicopathologic findings and survival analysis; and S: Observational studies (cohort studies, case–control studies, or cross‐sectional studies) and case series with at least 10 cases. Only case series with ≥ 10 patients were included to reduce the risk of bias associated with small samples, such as case reports, which tend to present non‐generalizable results. This criterion ensures greater consistency and reliability of the analyzed data.

Inclusion was based on the confirmation of sarcoma diagnosis as reported in the original studies, primarily through histopathological analysis described by the authors, and complemented, when available, by ancillary techniques such as immunohistochemistry (IHC) or molecular testing. All reported histopathological subtypes were subsequently reviewed and reclassified according to the current WHO Classification of Tumours Editorial Board (2020). For cases described with outdated terminology, the diagnoses were updated to align with current WHO nomenclature. Importantly, no re‐evaluation of original histological slides or additional IHC/molecular analyses was performed. This strategy ensured consistent and up‐to‐date categorization of sarcoma subtypes across all included cases, while acknowledging the limitations inherent to relying solely on published data.

In this study, the oral and maxillofacial region was defined as comprising the structures of the oral cavity, mandible, maxilla, zygomatic bone, temporomandibular joint, maxillary sinus, bony nasal cavity, oropharynx, facial soft tissues, salivary glands, and regional nerves. When data in the included case series and observational studies were reported only as percentages, absolute values were calculated whenever the total number of cases was available. In instances where such conversion was not possible or the data remained ambiguous, these variables were excluded from the quantitative synthesis. This approach was adopted to ensure accuracy and consistency in the extracted information.

Exclusion criteria were as follows: (1) studies that did not specifically investigate the clinicopathologic profile of oral and maxillofacial sarcomas; (2) studies with incomplete or insufficient clinicopathological data for analysis, particularly regarding follow‐up information; (3) studies that did not use histopathology as the reference standard for diagnosis; (4) reviews, case reports, protocols, short communications, personal opinions, letters, conference abstracts, book chapters, and in vitro or in vivo studies; (5) studies that did not include primary oral and maxillofacial sarcomas; (6) studies that included cases from other anatomic sites combined with oral and maxillofacial sarcomas, where data were aggregated and could not be separated; (7) studies where the full text was not available; (8) studies with duplicate samples; and (9) studies with an insufficient number of cases for meaningful analysis, operationalized as fewer than 10 cases.

### 2.3 Study Selection and Data Collection Process

The selection process was then conducted in two phases by three independent authors (I.V.F., M.E.S.C., and T.C.K.). The first phase involved reading the titles and abstracts of studies selected in Rayyan (Ouzzani et al. 2016). Studies that met all inclusion criteria proceeded to the second stage of the selection process through full‐text review and confirmation of eligibility criteria. Disagreements between the initial three reviewers were resolved by a fourth reviewer (R.A.L.S.).

Data were extracted by three reviewers (I.V.F., M.E.S.C., and T.C.K.) and validated by the entire research team. The following key data were extracted, when available: study characteristics (author/year, country, and study design); population characteristics (sample size, sex, age, and conditions/comorbidities of the patients); sarcoma characteristics (location, clinical appearance, symptoms, histological subtype, molecular profile, clinical TNM, staging, recurrence/metastasis, treatment, margin status, patient condition, follow‐up); and survival analysis.

### 2.4 Risk of Bias Assessment

The risk of bias in individual studies was independently assessed by three authors (I.V.F., M.E.S.C., and T.C.K.) using the Joanna Briggs Institute critical appraisal tool for each study. The risk of bias was classified as high if the study reached up to 49% “yes”; moderate if the study reached 50%–69% “yes”; and low if the study reached at least 70% “yes”. Disagreements were resolved first by discussion and then by consulting a fourth author (R.A.L.S.).

### 2.5 Data Analysis

The collected data were organized using Microsoft Excel 2019 (Microsoft) and presented descriptively. For statistical analysis, only cases with individually reported follow‐up times and patient status were included. Sample size varied according to clinicopathological variables. The correlation between clinicopathologic characteristics and patient status was assessed by chi‐square test. Survival rates were estimated using the Kaplan–Meier method, and differences between survival curves were analyzed using the univariate log‐rank test. To identify potential prognostic factors, the univariate Cox proportional hazards regression model was used to determine the hazard ratio (HR) and its 95% confidence interval. Multivariate regression analysis was not conducted due to the limited number of cases with complete data for all relevant variables. Statistical analyses were performed using GraphPad Prism (version 9.3.0, Dotmatics) and the Statistical Package for the Social Sciences (SPSS, version 22.0, IBM Corporation), with a *p* < 0.05 considered statistically significant.

### 2.6 Protocol and Registration

This systematic review was conducted according to the PRISMA (Preferred Reporting Items for Systematic Reviews and Meta‐Analyses) guidelines (Page et al. 2021) and was registered on the PROSPERO (International Prospective Register of Systematic Reviews) database (CRD42024608805).

## 3 Results

### 3.1 Study Selection and Characteristics of the Studies

The electronic search in the databases yielded 10,072 references, from which 4610 duplicates were removed, resulting in 5462 studies screened by title and abstract. On the basis of the inclusion and exclusion criteria, 1039 studies were selected for full‐text reading, and 32 studies met all eligibility criteria and were included in the review. Reasons for the exclusion of studies after full‐text screening are detailed in Table S2.

In addition, 231 records were identified through gray literature and manual reference checking of the previously included studies. After removing 68 duplicates, 163 studies were screened by title and abstract, and 45 were selected for full‐text reading. Following full‐text analysis, 3 additional studies met the inclusion criteria and were incorporated into the review. Reasons for exclusion at this stage are detailed in Table S3. In total, 35 studies were included in the systematic review, comprising 687 cases of oral and maxillofacial sarcomas published between 1982 and 2024. Cohen's kappa statistic for inter‐reviewer agreement in phase 2 was 0.801 (*p* = 0.000). The flowchart illustrates the study selection process in detail (Figure S1).

The selected studies were from 16 countries, as shown below: United States (9), China (7), India (4), Spain (2), France (2), Germany (1), Canada (1), Japan (1), Egypt (1), Brazil (2), Greece and Germany (1), Taiwan (1), Italy (1), United Kingdom (1), and Mexico (1).

### 3.2 Description of the Individual Studies

#### 3.2.1 Clinical Features

The summarized data is presented in Table 1, with detailed descriptions of the 687 analyzed cases available in Table S4. Oral and maxillofacial sarcomas were more common in males, representing 57% of cases (385 out of 676), with a male‐to‐female ratio of 1.32:1. Age information was available for 448 cases, with an average patient age of 35.26 years (±21.53), ranging from 0.3 to 91 years. Rhabdomyosarcoma was the most common histological subtype among children and adolescents, with a mean age of 9.87 years (0.3–77 years). This was followed by Ewing sarcoma, with a mean age of 16.8 years (4–30 years). In contrast, osteosarcoma was the most frequent subtype in young adults, with a mean age of 33.87 years (1–84 years), while liposarcoma predominantly affected older patients, with a mean age of 50.40 years (28–83 years).

**TABLE 1:** Summary of 687 sarcoma cases in the oral and maxillofacial region.

Variables	*n* (%)
**Sex (*n* = 676)**	
Male	385 (57%)
Female	291 (43%)
**Age (years, *n* = 448)**	
Mean	35.26
Standard deviation	21.53
Range	0.3–91
**Anatomical location (*n* = 687)**	
Mandible	260 (37.8%)
Maxilla	202 (29.4%)
Nasal and maxillary sinus region^a^	72 (10.5%)
Oral cavity^b^	57 (8.3%)
Nasolabial fold	38 (5.5%)
Face^c^	31 (4.5%)
Parotid region	19 (2.8%)
Others^d^	8 (1.2%)
**Histological type (*n* = 687)**	
Osteosarcoma	306 (44.5%)
Rhabdomyosarcoma	67 (9.8%)
Chondrosarcoma	67 (9.8%)
Synovial sarcoma	52 (7.6%)
Undifferentiated pleomorphic sarcoma	49 (7.1%)
Ewing's sarcoma	40 (5.8%)
Leiomyosarcoma	37 (5.4%)
Liposarcoma	28 (4.1%)
Fibrosarcoma	11 (1.6%)
Spindle cell sarcoma	8 (1.2%)
Angiosarcoma	6 (0.9%)
Malignant peripheral nerve sheath tumor	4 (0.6%)
Kaposi sarcoma	3 (0.4%)
Myeloid sarcoma	3 (0.4%)
Dermatofibrosarcoma protuberans	3 (0.4%)
Low grade sarcoma	1 (0.1%)
Biphenotypic sinonasal sarcoma	1 (0.1%)
Alveolar soft part sarcoma	1 (0.1%)
**Conditions/comorbidities (*n* = 95)**	
Radiation‐induced sarcomas^e^	89 (93.6%)
Li‐Fraumeni syndrome	3 (3.2%)
History of trauma	2 (2.1%)
Polyostotic fibrous dysplasia	1 (1.1%)
**Clinical appearance/symptoms (*n* = 277)**	
Swelling	130 (46.9%)
Mass	65 (23.5%)
Pain	62 (22.4%)
Enlarging mass syndrome	29 (10.5%)
Nasal obstruction	21 (7.6%)
Trismus	18 (6.5%)
Numbness	15 (5.4%)
Cachexia	15 (5.4%)
Dental alterations	12 (4.3%)
Ocular manifestations	8 (2.9%)
Bleeding	8 (2.9%)
Osteoradionecrosis	5 (1.8%)
Epistaxis	5 (1.8%)
Garrington's sign	5 (1.8%)
Others^f^	38
**T classification (*n* = 67)**	
T1/T2	57 (85.1%)
T3/T4	10 (14.9%)
**N classification (*n* = 53)**	
N0	43 (81.1%)
N1	10 (18.9%)
**M classification (*n* = 33)**	
M0	30 (90.9%)
M1	3 (9.1%)
**Stage grouping (*n* = 221)**	
I/II	158 (71.5%)
III/IV	63 (28.5%)
**Treatment (*n* = 546)**	
Multimodal^g^	334 (61.2%)
S alone	181 (33.2%)
CT alone	20 (3.7%)
RT alone	3 (0.5%)
None	7 (1.3%)
Antiretroviral medication	1 (0.2%)
**Margin status (*n* = 263)**	
Negative	160 (60.8%)
Positive	102 (38.8%)
Marginal resection	1 (0.4%)
**Local recurrence (*n* = 299)**	
Yes	194 (64.9%)
No	105 (35.1%)
**Nodal metastasis (*n* = 128)**	
Yes	19 (14.8%)
No	109 (85.2%)
**Distant metastasis (*n* = 195)**	
Yes	55 (28.2%)
No	140 (71.8%)
**Follow‐up (months) (*n* = 417)**	
Mean	60.74
Standard deviation	79.7
Range	0.8–479
**Status (*n* = 616)**	
Alive	358 (58.1%)
Dead	258 (41.9%)

*Note:* CT, chemotherapy; RT, radiation therapy; S, surgery.

^a^ Maxillary sinus (39), nasal cavity (29), nasal fossa (2), nasal septum (2).

^b^ Tongue (19), gingiva (9), palate (6), lip (4), floor of mouth (4), buccal mucosa (6), alveolus (2), retromolar trigone (2), oral cavity NOS (3), buccal area (1), buccal vestibule (1).

^c^ Cheek (20), chin (1), face NOS (5), facial buccal pad (1), zygomatic area (1), submental (1), submaxillary region (1), submandibular region (1).

^d^ Temporomandibular (6), tonsil (2).

^e^ Osteosarcoma (65), undifferentiated pleomorphic sarcoma (16), fibrosarcoma (7), spindle cell sarcoma (1).

^f^ Paresthesia (5), weight loss (5), tenderness, ulceration (each 4); dysesthesia, proptosis, ocular signs, respiratory stridor, dysphagia, facial paralysis (each 2); epiphora, facial paralysis, lymphadenopathy, hemoptysis, polypoid lesion, pedicle‐like lesion, painful teeth, discharge, sinusitis, loss of smell, malocclusion of teeth, ptosis of eyelid, headache, hypoesthesia, expansive lesion, hard nodule, erythematous lesion (each 1).

^g^ S + CT (129), S + RT + CT (95), S + RT (82), RT + CT (21), S + RT + CT/target therapy (6), S + CT/target therapy (1).

Anatomically, the mandible was the most frequently affected site, accounting for 37.8% of cases (260/687), followed by the maxilla at 29.4% (202/687). Histopathologic subtypes were updated according to the (WHO 2020) classification, revealing over 16 distinct variants. Osteosarcoma was the most common subtype, representing 44.5% of cases (306/687), followed by rhabdomyosarcoma (9.8%; 67/687), and chondrosarcoma (9.8%; 67/687).

Predisposing conditions and comorbidities were reported in a subset of patients. Among the 687 sarcoma cases, 89 (13.0%) were classified as radiation‐associated sarcomas (RAS), most frequently osteosarcoma (65/89; 73.0%), followed by undifferentiated pleomorphic sarcoma (UPS) (16/89; 18.0%), fibrosarcoma (7/89; 7.9%), and spindle cell sarcoma (1/89; 1.1%). Information regarding the primary tumor was available for 86 of the 89 radiation‐induced cases, most commonly nasopharyngeal carcinoma (73/86; 84.9%), followed by squamous cell carcinoma (6/86; 7.0%), and, in single cases (1/86 each; 1.2%), melanoma, Hodgkin lymphoma, adenoid cystic carcinoma, basal cell carcinoma, mucoepidermoid carcinoma, malignant teratoma, and non‐Hodgkin lymphoma. Specifically, among the 65 cases of radiation‐induced osteosarcoma, primary tumor data were reported in 62 cases, with nasopharyngeal carcinoma accounting for the majority (52/62; 83.9%). According to three studies (Matthew Debnam et al. 2012; Liao et al. 2016; Zhu et al. 2016), the latency period between radiotherapy and sarcoma onset ranged from 2.5 to 34 years. Additionally, three osteosarcoma patients had a history of Li‐Fraumeni syndrome, one had polyostotic fibrous dysplasia, and prior trauma was reported in one case each of osteosarcoma and Ewing sarcoma.

Fourteen studies, covering 277 cases, reported clinical appearance and/or symptoms, with some patients presenting multiple symptoms. The most frequent clinical findings were swelling (130/277; 46.9%), mass formation (65/277; 23.5%), pain (62/277; 22.4%), and enlarging mass syndrome (29/277; 10.5%). Other commonly observed symptoms included nasal obstruction (21/277; 7.6%), trismus (18/277; 6.5%), numbness (15/277; 5.4%), and cachexia (15/277; 5.4%). Dental alterations were reported in 12/277 cases (4.3%), ocular manifestations in 8/277 cases (2.9%), and bleeding in 8/277 cases (2.9%). Less common symptoms such as osteoradionecrosis, epistaxis, and Garrington's sign were reported in 5/277 cases each (1.8%). Other symptoms with even lower frequencies were also identified and are detailed in Table 1.

#### 3.2.2 Staging

Complete staging data were not available for the majority of the 687 patients. The staging was performed at the diagnosis and based on limited subsets: T classification (*n* = 67), N classification (*n* = 53), M classification (*n* = 33), and stage grouping (*n* = 221). This partial availability should be taken into account when interpreting the results presented below.

The T classification distribution showed that 57/67 cases (85.1%) were classified as T1/T2, while 10/67 cases (14.9%) were classified as T3/T4. For the N classification, 43/53 cases (81.1%) showed no lymph node involvement (N0), while 10/53 cases (18.9%) presented with lymph node metastasis (N1). Regarding the M classification, 30/33 cases (90.9%) had no distant metastasis (M0), while 3/33 cases (9.1%) had distant metastasis (M1).

In 221 analyzed cases, 158 (71.5%) were classified as stages I/II, while 63 (28.5%) were classified as stages III/IV. Two studies that examined 37 cases of rhabdomyosarcoma classified the patients according to the IRS Group (Intergroup Rhabdomyosarcoma Study Group). The majority of cases were assigned to Group III (84.2%), while Groups II and IV accounted for 13.2% and 2.6%, respectively.

#### 3.2.3 Molecular Analysis

Molecular testing was performed in only 18 of the 687 cases analyzed. The presence of the *PAX3::FOXO1* or *PAX7::FOXO1* gene fusion was assessed by fluorescence in situ hybridization (FISH) in 17 patients diagnosed with rhabdomyosarcoma, being positive in 14 and negative in 3. Furthermore, the molecular characteristics of a case of UPS were investigated through whole exome sequencing. Cancer driver gene analysis identified *GBP4* as a potential driver gene associated with primary UPS of the oral and maxillofacial region. A missense mutation in the *PIK3CA* gene (p.E545K) was also detected.

#### 3.2.4 Treatment, Tumor Behavior, and Follow‐Up

Among a total of 687 cases, data availability varied across key variables: treatment information was reported for 546 cases, margin status for 263 cases, local recurrence for 299 cases, nodal metastasis for 128 cases, distant metastasis status for 195 cases, follow‐up duration for 417 cases, and patient status for 616 cases. These differences in data completeness should be taken into account when interpreting the following analyses.

Multimodal treatment was the most frequently applied, involving 334 patients (61.2%). The combinations included surgery combined with chemotherapy, radiotherapy, and targeted therapy. Surgery alone was performed in 181 cases (33.2%). Exclusive chemotherapy was administered to 20 patients (3.7%), while radiotherapy alone was used in 3 patients (0.5%). One patient (0.2%) received antiretroviral medication, and 7 patients (1.3%) did not receive any treatment. Tumor margin data were available for 263 cases. Among them, 160 cases (60.8%) had tumor‐free (negative) margins, 102 cases (38.8%) had compromised (positive) margins, and 1 case (0.4%) underwent marginal resection.

Local recurrence was observed in 194 of 299 cases (64.9%). Nodal metastases occurred in 19 of 128 cases (14.8%), while distant metastases were reported in 55 of 195 cases (28.2%). Metastatic sites were described in 18 cases and included 12 in the lungs, 4 in bones, and 2 in the brain. Median follow‐up time was available for 417 cases, with a mean of 60.74 months (±79.7), ranging from 0.8 to 479 months. Regarding patient status, most patients were alive at the time of analysis (358/616; 58.1%), whereas 258/616 (41.9%) had died.

### 3.3 Synthesis of the Results and Statistical Analysis

**FIGURE 1:**
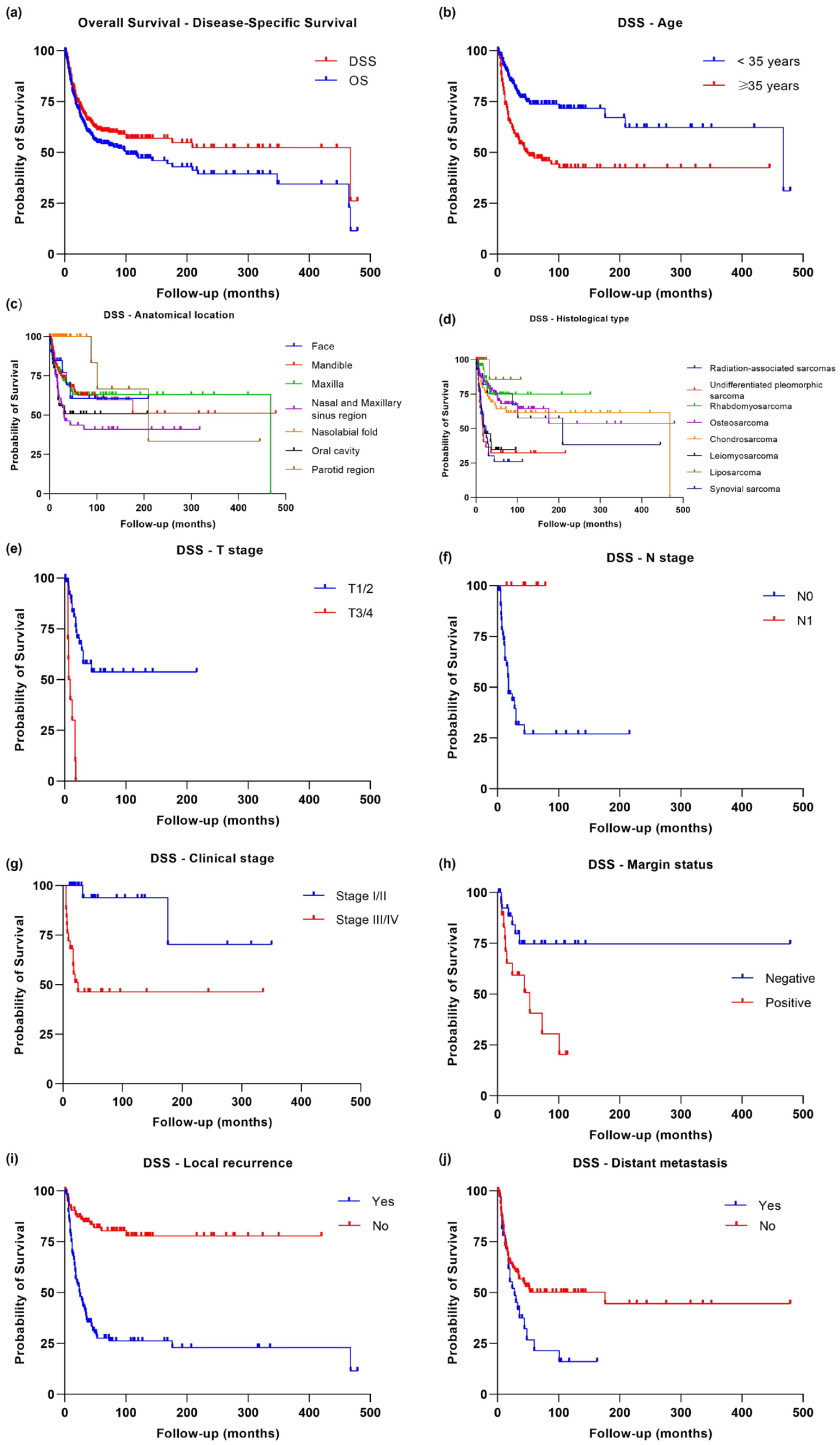
Disease‐specific survival curves (DSS). (a) Kaplan‐Meier curve demonstrating the comparative curve between OS and DSS of patients affected by oral and maxillofacial sarcomas. Using Log‐Rank univariate analysis, (b) age (*p* < 0.0001), (c) anatomical location (*p* = 0.0065), (d) histological type (*p* < 0.0001), (e) T classification (*p* < 0.0001), (f) N classification (*p* = 0.0066), (g) stage grouping (*p* = 0.0004), (h) margin status (*p* = 0.0107), (i) local recurrence (*p* < 0.0001), and (j) distant metastasis (*p* = 0.0224) significantly impact the survival rate of oral and maxillofacial sarcoma.

A total of 406/687 cases with complete information on follow‐up time and patient status were included in the statistical analyses. The 5‐, 10‐, and 15‐year overall survival (OS) rates were 54.3%, 47.2%, and 42.9%, respectively, and the disease‐specific survival (DSS) rates for the same intervals were 60.4%, 56.9%, and 54.7%. The Log‐rank analysis revealed significant correlations between decreased OS (Figure S4) and DSS (Figure 1) and factors such as age (*p* < 0.0001), histologic type (*p* < 0.0001), T classification (*p* < 0.0001), N classification (DSS: *p* = 0.0066; OS: *p* = 0.0314), stage grouping (DSS: *p* = 0.0004; OS: *p* = 0.0003), margin status (DSS: *p* = 0.0107; OS: *p* < 0.0001), local recurrence (*p* < 0.0001), and distant metastases (DSS: *p* = 0.0224; OS: *p* = 0.0021). However, nodal metastases (*p* = 0.0012) were significantly associated only with OS, whereas anatomical location (*p* = 0.0065) was specifically associated with DSS.

The univariate Cox regression analysis for DSS indicated that variables such as age (HR = 0.372; 95% CI: 0.251–0.551; *p* < 0.0001), histological subtype (HR = 0.373; 95% CI: 0.216–0.643; *p* < 0.0001), T classification (HR = 0.162; 95% CI: 0.072–0.366; *p* < 0.0001), stage grouping (HR = 0.108; 95% CI: 0.024–0.481; *p* < 0.0001), margin status (HR = 0.270; 95% CI: 0.142–0.514; *p* < 0.0001), local recurrence (HR = 0.182; 95% CI: 0.109–0.304; *p* < 0.0001), and distant metastases (HR = 0.575; 95% CI: 0.350–0.945; *p* < 0.029) significantly influenced the patient survival rate (Table S5). Notably, individuals younger than 35 years had a significantly lower risk of disease‐specific mortality compared to those aged 35 or older. Patients with advanced T classification (T3/T4) faced a 6.2‐fold higher risk of death than those with early‐stage tumor (T1/T2). Likewise, patients in stage groups III/IV had a 9.3‐fold increased mortality risk compared to stages I/II. The presence of local recurrence was associated with a 5.5‐fold increase in the risk of death, while distant metastases raised the risk by 1.7‐fold.

#### 3.3.1 Subtypes and Overall Survival

Kaplan–Meier curves for OS by histological subtype (*p* < 0.0001) are shown in Figure S4C, and Table 2 presents OS alongside clinicopathological characteristics and treatment. Cases with prior radiation exposure were analyzed separately, although they do not represent a histological subtype. The poorest 2‐year OS was observed in RAS (12%), leiomyosarcoma (12%), and UPS (13%), whereas rhabdomyosarcoma (45%) and osteosarcoma (67%) showed the highest survival rates. When stratified by treatment modality within each histological subtype, no statistically significant differences were observed (osteosarcoma, *p* = 0.0512; rhabdomyosarcoma, *p* = 0.5158; chondrosarcoma, *p* = 0.0639; RAS, *p* = 0.6606; synovial sarcoma, *p* = 0.2254; UPS, *p* = 0.6824; leiomyosarcoma, *p* = 0.2239). These findings underscore the heterogeneous clinical behavior of sarcomas and highlight the prognostic relevance of histological subtype and prior radiation exposure. Although no significant differences were observed by treatment modality, the limited sample size within subgroups should be considered when interpreting these results.

**TABLE 2:** Clinicopathological characteristics, treatment, and overall survival of sarcoma cases by histological subtype.

Histological type	Age, year (mean ± SD)	Sex	Treatment	LR	RM	DM	Follow‐up, m (mean ± SD)	Status
Osteosarcoma (*n* = 88)	35.25 ± 21.72	M (48) F (40)	Surgery (23) Multimodal (53) RT and/or CT (8) NI (4)	Yes (31) No (39) NI (18)	Yes (0) No (29) NI (59)	Yes (10) No (52) NI (26)	79.95 ± 95.58	Alive (55) Dead (33)
Rhabdomyosarcoma (*n* = 58)	10.94 ± 16.39	M (29) F (19) NI (10)	Surgery (2) Multimodal (45) RT and/or CT (8) NI (3)	Yes (15) No (10) NI (33)	Yes (13) No (12) NI (33)	Yes (9) No (9) NI (40)	36.74 ± 48.61	Alive (38) Dead (20)
Chondrosarcoma (*n* = 54)	38.38 ± 16.94	M (27) F (27)	Surgery (30) Multimodal (16) RT and/or CT (3) NI (5)	Yes (18) No (22) NI (14)	Yes (0) No (4) NI (50)	Yes (4) No (4) NI (46)	106.46 ± 118.54	Alive (31) Dead (23)
Radiation‐associated sarcoma (*n* = 38)	49.73 ± 13.15	M (21) F (17)	Surgery (26) Multimodal (8) RT and/or CT (3) NI (1)	Yes (19) No (1) NI (18)	Yes (0) No (27) NI (11)	Yes (2) No (13) NI (23)	24.36 ± 26.62	Alive (12) Dead (26)
Synovial sarcoma (*n* = 28)	38.10 ± 18.44	M (18) F (10)	Surgery (4) Multimodal (8) NI (16)	Yes (5) No (5) NI (18)	Yes (0) No (0) NI (28)	Yes (0) No (0) NI (28)	93.05 ± 98.20	Alive (10) Dead (18)
Undifferentiated pleomorphic sarcoma (*n* = 31)	46.12 ± 16.44	M (20) F (11)	Surgery (7) Multimodal (22) RT and/or CT (2)	Yes (18) No (11) NI (2)	Yes (1) No (12) NI (18)	Yes (5) No (17) NI (9)	42.91 ± 55.58	Alive (11) Dead (20)
Ewing's sarcoma (*n* = 5)	16.80 ± 9.25	M (4) F (1)	Multimodal (4) RT and/or CT (1)	Yes (1) No (0) NI (4)	Yes (0) No (0) NI (5)	Yes (0) No (0) NI (5)	75.20 ± 41.20	Alive (5)
Leiomyosarcoma (*n* = 30)	38.30 ± 22.50	M (14) F (16)	Surgery (16) Multimodal (13) NI (1)	Yes (15) No (6) NI (9)	Yes (2) No (17) NI (11)	Yes (1) No (17) NI (12)	25.26 ± 21.97	Alive (11) Dead (19)
Liposarcoma (*n* = 17)	51.82 ± 14.96	M (10) F (7)	Multimodal (2) RT and/or CT (1) NI (14)	Yes (5) No (1) NI (11)	Yes (0) No (2) NI (15)	Yes (1) No (2) NI (14)	34.58 ± 28.13	Alive (16) Dead (1)

*Note:* CT, chemotherapy; DM, distant metastasis; LR, local recurrence; m, months; NI, not informed; RM, regional metastasis; RT, radiotherapy; y, years.

### 3.4 Risk of Bias Within Studies

The risk of bias assessment, using the Joanna Briggs Institute tool, was conducted for 34 cross‐sectional studies and 1 case series. Among the 34 cross‐sectional studies and 1 case series, 19 (54.3%) demonstrated a low risk of bias, 14 (40.0%) showed a moderate risk, and 2 (5.7%) had a high risk. Studies with low risk typically demonstrated clearly defined inclusion criteria, comprehensive descriptions of participants, and appropriate statistical analyses. In contrast, studies with moderate to high risk frequently lacked adequate control of confounding factors and provided insufficient detail regarding the validity of outcome measurement methods.

Detailed assessments for each study are available in Figures S2 and S3.

## 4 Discussion

Sarcomas of the oral and maxillofacial region constitute a diverse group of cancers (O'Neill et al. 2013). Due to the low frequency of cases diagnosed specifically in the oral and maxillofacial region, there are few studies that investigate their characteristics in detail. In this systematic review, we analyzed 687 cases reported in 35 articles published between 1982 and 2024. This study consolidates information on the demographic, clinical, pathologic, and therapeutic aspects of these sarcomas, as well as data on patient follow‐up and related survival rates.

This review found that oral and maxillofacial sarcomas are slightly more common in men. Given the inclusion of various histologic subtypes, sex predilection may vary depending on the subtype, reflecting the broad age range observed. Our results are consistent with the literature, which shows that rhabdomyosarcoma predominantly occurs in children and adolescents (Gallagher et al. 2022), Ewing sarcoma mainly affects children and young adults with a slight male predominance (Tran et al. 2020), and osteosarcoma is most frequent among young adults without significant sex predilection (Ottaviani and Jaffe 2009; Tran et al. 2020). Additionally, liposarcoma tends to affect older patients (WHO 2020). These findings support the epidemiological patterns observed in our study.

Regarding the anatomical origin, these tumors may arise from any non‐epithelial tissue within the region (Wreesmann et al. 2022). Although sarcomas in this area are generally more common in soft tissues than in bone or cartilage (Kumar et al. 2019), some studies have reported a higher prevalence of bone and cartilage sarcomas specifically within the oral cavity (Alishahi et al. 2015). Consistent with these findings, the majority of cases in our study (70.46%) involved the bones of the oral and maxillofacial region. Tumor location also plays a crucial role in tumor behavior and patient prognosis. We found a significant association between location and DSS, with sarcomas in the nasal region and maxillary sinuses exhibiting a poorer 5‐year prognosis (43.6%). Concerning histological subtypes, osteosarcoma was the most frequent in our cohort, consistent with recent Brazilian data that also highlight osteosarcomas, Kaposi sarcomas, and chondrosarcomas as the most common types in the oral region (de Carvalho et al. 2020).

Oral and maxillofacial sarcomas often present with nonspecific signs and symptoms that can be mistaken for benign or malignant soft tissue neoplasms (Sturgis and Potter 2003). This similarity in clinical presentation represents a significant diagnostic challenge. In some cases, symptoms are related to the involvement of adjacent anatomical structures, such as the skull base, nasosinus tract, or larynx (Makary et al. 2017). Therefore, distinguishing sarcomas from other entities in the differential diagnosis is essential for appropriate and timely management. The most common clinical signs include mass growth, with or without pain, tooth mobility, cranial nerve dysfunction, unilateral sinusitis, frequent nasal bleeding, voice changes, and difficulty or pain in swallowing (Kalavrezos and Sinha 2020). The symptomatic findings reported in the literature are consistent with those observed in our study.

Exposure to external beam ionizing radiation in the oral and maxillofacial region has been linked to the development of sarcomas, as it can cause DNA damage and disrupt the cell cycle (Coca‐Pelaz et al. 2021). These sarcomas are rare and often have a poor prognosis (Liao et al. 2023), with a latency period of 10–12 years after radiation exposure (Giannini et al. 2018; Williams et al. 2018). In our review, we found that radiation‐induced sarcomas have the worst prognosis, with a 5‐year overall survival rate of 20.4%. These sarcomas show lower survival rates, likely due to factors such as local immune system suppression in the irradiated area, the effect of radiotherapy on the genetic makeup of tumor cells, challenges in effectively treating the irradiated area, and diagnostic delays due to anatomic and histologic changes in the affected area (Patel 2000; Wreesmann et al. 2022). Among the histological subtypes, osteosarcoma was the most prevalent radiation‐associated sarcoma. Nasopharyngeal carcinoma was the most common histological subtype of the primary tumor, which is consistent with findings reported in the literature (Coca‐Pelaz et al. 2021). Additionally, Li‐Fraumeni syndrome, caused by mutations in the p53 gene, increases the risk of soft tissue and bone sarcomas, which account for approximately one‐quarter of the tumors in affected individuals (Malkin et al. 1990; Zahm and Fraumeni 1997; Sturgis and Potter 2003; Makary et al. 2017). In our study, three patients with osteosarcoma were diagnosed with this syndrome.

Sarcomas exhibit significant complexity in their classification and subtyping, with over 50 recognized histological subtypes and potential involvement of various anatomical sites and organ systems (Bovée and Hogendoorn 2010; de Carvalho et al. 2020; Wreesmann et al. 2022). Although the identification of diagnostically relevant genetic alterations has progressed, most cases are still diagnosed based on histological criteria, which are often imprecise and subject to interobserver variability. This reduces diagnostic accuracy and complicates the distinction between benign and malignant soft tissue lesions, as well as between different sarcoma subtypes (Demicco 2013; Wreesmann et al. 2022). Molecular tests such as FISH allow for the detection of genetic rearrangements and specific mutations, supporting tumor diagnosis, classification, and treatment planning. These tools are crucial for identifying prognostic biomarkers and tailoring therapies, with the potential to improve clinical outcomes while minimizing adverse effects (Bovée and Hogendoorn 2010; Demicco 2013; Luk et al. 2019; Wreesmann et al. 2022).

Despite the increasing relevance of molecular profiling, only 18 out of 687 cases in the present study underwent genetic testing, 17 of which were rhabdomyosarcomas. FISH is not applicable to the embryonal subtype; however, approximately 80% of alveolar rhabdomyosarcomas present specific chromosomal translocations—t(2;13)(q35;q14) and t(1;13)(p36;q14)—resulting in *FOXO1::PAX3* fusions in about 55% of cases and *FOXO1::PAX7* in around 20% (Mehra et al. 2008; Downs‐Kelly et al. 2009; Luk et al. 2019). The low rate of molecular testing (2.7%) reveals a major gap and reinforces the need to incorporate these tools into the routine diagnosis of oral and maxillofacial sarcomas, particularly in complex or unclassified cases. Future efforts should prioritize broader use of molecular tests, multicenter studies with larger cohorts, and the creation of biobanks to identify novel genetic markers, improve diagnostic accuracy, and guide targeted therapies.

The standard treatment for sarcomas is surgical resection of the primary tumor, often combined with chemotherapy and/or radiotherapy, depending on the histologic subtype (de Bree et al. 2010; Barosa et al. 2014; Grünewald et al. 2020). Oral and maxillofacial sarcomas present particular therapeutic challenges due to the anatomic complexity of the region, which often hampers the achievement of adequate surgical margins (de Bree et al. 2010; Barosa et al. 2014). In our study, Cox regression analysis demonstrated that achieving negative surgical margins significantly impacted prognosis, with patients presenting a 73% lower risk of disease‐specific mortality. This finding reinforces the critical importance of complete tumor excision in improving survival outcomes for patients with sarcomas in this region.

The prognosis of oral and maxillofacial sarcomas is generally less favorable compared to sarcomas arising in other anatomical sites (Wreesmann et al. 2022). Progressive local recurrence remains a leading cause of mortality, often occurring before distant metastasis, which underscores the detrimental effect of incomplete surgical resection. Our findings reinforce that patients with more advanced tumor classifications and clinical stages experience significantly higher mortality, highlighting the importance of early diagnosis and effective initial management. In addition, features such as tumor size, histological grade, nodal involvement, and history of radiotherapy also play a crucial role in shaping clinical outcomes (Makary et al. 2017). These observations suggest that improved local control and timely intervention in early‐stage disease are key strategies to enhance survival.

This study has some important limitations that should be highlighted. First, many of the included articles did not provide clear demographic information or individualized clinical analyses and presented data in an aggregated form, making it difficult to assess specific characteristics. Additionally, there was a significant lack of essential information, such as TNM classification, staging, surgical margins, presence of recurrences, metastases, and/or molecular data, which prevented more in‐depth analyses and stronger conclusions. Furthermore, IHC data were inconsistently reported across studies, often absent, grouped, or lacking case‐specific detail. Combined with the high heterogeneity of sarcoma subtypes, this prevented systematic comparative analysis. For subtypes requiring IHC or molecular tests, results were recorded as reported in the original studies. In cases where such tests were not available or not reported, we relied on the diagnosis provided by the original authors. We emphasize that this is an inherent limitation of retrospective studies, in which it is not possible to perform additional tests or re‐evaluate individual cases.

Another limitation concerns the absence or low number of some subtypes, such as odontogenic sarcomas and Kaposi's sarcoma, which may not have met the inclusion criteria due to small sample sizes, lack of follow‐up information, or the aggregation of data from different anatomical sites, making it impossible to isolate cases located specifically in the oral and maxillofacial region. We also emphasize that, to ensure the quality and comparability of the data, only studies with case series including at least 10 patients were included, which may have limited the total number of cases analyzed and the scope of the findings, especially compared to systematic reviews that included studies with smaller samples, such as case reports. Finally, although sarcoma subtypes have distinct etiologies and clinical behaviors, this work chose to approach the cases primarily by anatomic location rather than prioritizing histological aspects, which may limit the detailed understanding of each specific subtype. However, despite these limitations, the work makes a significant contribution to the clinical understanding of oral and maxillofacial sarcomas, providing a valuable overview of the disease patterns and their potential clinical outcomes, which may guide future research and improve the management of these cases in clinical practice.

## 5 Conclusion

The findings of this study provide a relevant contribution to the understanding of the clinicopathological characteristics of oral and maxillofacial sarcomas. These tumors show a slight male predominance, affect a wide age range (0.3–91 years), and primarily involve the mandible, followed by the maxilla. Among the subtypes, osteosarcoma stands out as the most common and is also the subtype most frequently associated with prior radiation exposure. Multimodal treatment remains the main therapeutic approach. This study demonstrated the significant influence of factors such as age, histological subtype, T classification, clinical stage, surgical margins, local recurrence, and distant metastases on patient survival, emphasizing the importance of early diagnosis. However, the available evidence is limited by the heterogeneity of histological subtypes and the lack of comprehensive clinicopathological data.

Additionally, the study aimed to deepen the understanding of the molecular characteristics of oral and maxillofacial sarcomas, but the scarcity of available molecular information reinforces the need for further research. Future studies should focus on identifying molecular markers and developing personalized therapeutic approaches to improve prognosis and guide clinical decision‐making. The limited number of investigations specifically targeting this anatomical region highlights the importance of the present study, as maxillofacial sarcomas exhibit distinct features and biological behavior compared to sarcomas of long bones.

## Author Contributions

**Iara Vieira Ferreira:** conceptualization, investigation, writing – original draft, methodology, data curation, formal analysis. **Reydson Alcides de Lima‐Souza:** investigation, writing – original draft, methodology, data curation, formal analysis. **Talita de Carvalho Kimura:** investigation, methodology, data curation. **Alfio José Tincani:** investigation, methodology, data curation. **Marcelo Elias Schempf Cattan:** writing – review and editing, formal analysis. **Arthur Antolini‐Tavares:** writing – review and editing. **Albina Altemani:** writing – review and editing. **Fernanda Viviane Mariano:** conceptualization, writing – review and editing, supervision.

## Conflicts of Interest

The authors declare no conflicts of interest.

## Supporting information


**Table S1:** Search strategies in databases and gray literature.
**Table S2:** Excluded articles and reasons for exclusion—Database (*n* = 1007).
**Table S3:** Excluded articles and reasons for exclusion—Gray literature (*n* = 42).
**Table S4:** Demographic and clinicopathological characteristics of the 35 studies (687 cases) of oral and maxillofacial sarcomas included in the systematic review.
**Table S5:** Clinical and tumor characteristics influencing disease‐specific survival in oral and maxillofacial sarcomas: Univariate cox analysis.
**Figure S1:** Flow diagram of literature search and selection criteria adapted from PRISMA.
**Figure S2:** Summary of the risk of bias in cross‐sectional studies, assessed using the Joanna Briggs Institute Critical Appraisal Checklist.
**Figure S3:** Summary of the risk of bias in case report study, assessed using the Joanna Briggs Institute Critical Appraisal Checklist.
**Figure S4:** Overall Survival (OS) curves. (a) Kaplan–Meyer curve demonstrating the OS of patients affected by oral and maxillofacial sarcomas. Using Log‐Rank univariate analysis, (b) age (*p* < 0.0001), (c) histological type (*p* < 0,0001), (d) T classification (*p* < 0.0001), (e) N classification (*p* = 0.0314), (f) stage grouping (*p* = 0.0003), (g) margin status (*p* < 0.0001), (h) local recurrence (*p* < 0.0001), (i) nodal metastasis (*p* = 0.0012), and (j) distant metastasis (*p* = 0.0021) significantly impact the survival rate of oral and maxillofacial sarcoma.

## Data Availability

The data that supports the findings of this study is available in the Supporting Information of this article.
