# Supplementary material for: Clinicopathologic Analysis of Sarcomas in the Oral and Maxillofacial Region: A Systematic Review
Source: Oral Dis. 2025 Sep 30;32(2):338–49. doi: 10.1111/odi.70103 (PMC13077021; doi:10.1111/odi.70103)
Supplement: Supplementary file 1 — Table S1: Search strategies in databases and gray literature. Table S2: Excluded articles and reasons for exclusion—Database (n = 1007). Table S3: Excluded articles and reasons for exclusion—Gray literature (n = 42). Table S4: Demographic and clinicopathological characteristics of the 35 studies (687 cases) of oral and maxillofacial sarcomas included in the systematic review. Table S5: Clinical and tumor characteristics influencing disease‐specific survival in oral and maxillofacial sarcomas: Univariate cox analysis. Figure S1: Flow diagram of literature search and selection criteria adapted from PRISMA. Figure S2: Summary of the risk of bias in cross‐sectional studies, assessed using the Joanna Briggs Institute Critical Appraisal Checklist. Figure S3: Summary of the risk of bias in case report study, assessed using the Joanna Briggs Institute Critical Appraisal Checklist. Figure S4: Overall Survival (OS) curves. (a) Kaplan–Meyer curve demonstrating the OS of patients affected by oral and maxillofacial sarcomas. Using Log‐Rank univariate analysis, (b) age (p < 0.0001), (c) histological type (p < 0,0001), (d) T classification (p < 0.0001), (e) N classification (p = 0.0314), (f) stage grouping (p = 0.0003), (g) margin status (p < 0.0001), (h) local recurrence (p < 0.0001), (i) nodal metastasis (p = 0.0012), and (j) distant metastasis (p = 0.0021) significantly impact the survival rate of oral and maxillofacial sarcoma. [file ODI-32-338-s001.docx]

**Table S1** Search strategies in databases and grey literature.

| **Database** | (Search date: June 27, 2024, and June 7, 2025) | **Results** |
| --- | --- | --- |
| **PubMed** | ("sarcoma"[MeSH Terms] OR "sarcoma"[All Fields] OR "sarcomas"[All Fields] OR "sarcoma s"[All Fields] OR "sarcoma soft tissue"[All Fields] OR "sarcomas soft tissue"[All Fields] OR "Soft Tissue Sarcoma"[All Fields] OR "Soft Tissue Sarcomas"[All Fields] OR "sarcoma epithelioid"[All Fields] OR "Epithelioid Sarcoma"[All Fields] OR "Epithelioid Sarcomas"[All Fields] OR "sarcomas epithelioid"[All Fields] OR "sarcoma spindle cell"[All Fields] OR "sarcomas spindle cell"[All Fields] OR "Spindle Cell Sarcoma"[All Fields] OR "Spindle Cell Sarcomas"[All Fields]) **AND** ("head and neck" OR "oral and maxillofacial"[All Fields]) **AND** (prevalence[MeSH Terms] OR prevalence OR frequency OR frequencies OR epidemiology[MeSH Terms] OR epidemiology[MeSH Subheading] OR epidemiology OR epidemiologic OR epidemiological OR occurrence OR occurrences OR incidence[MeSH Terms] OR incidence OR “cross-sectional studies”[MeSH Terms] OR “cross-sectional studies” OR “cross-sectional study” OR “cross sectional study” OR “cross sectional studies” OR “cross-sectional analysis” OR “cross-sectional analyses” OR “cross sectional analysis” OR “cross-sectional analyses” OR survey OR surveys OR “retrospective studies”[MeSH Terms] OR “retrospective studies” OR “retrospective study” OR “prospective studies”[MeSH Terms] OR “prospective studies” OR “prospective study” OR “observational study” OR “observational studies” OR nationwide OR populational OR population OR populations OR database OR databases) | **2497** |
| **Scopus** | TITLE-ABS-KEY (sarcomas OR "Sarcoma, Soft Tissue" OR "Sarcomas, Soft Tissue" OR "Soft Tissue Sarcoma" OR "Soft Tissue Sarcomas" OR "Sarcoma, Epithelioid" OR "Epithelioid Sarcoma" OR "Epithelioid Sarcomas" OR "Sarcomas, Epithelioid" OR "Sarcoma, Spindle Cell" OR "Sarcomas, Spindle Cell" OR "Spindle Cell Sarcoma" OR "Spindle Cell Sarcomas") **AND** TITLE-ABS-KEY ("head and neck" OR "oral and maxillofacial") **AND** TITLE-ABS-KEY (prevalence OR frequency OR frequencies OR epidemiology OR epidemiologic OR epidemiological OR occurrence OR occurrences OR incidence OR "cross-sectional studies" OR "cross-sectional study" OR "cross sectional study" OR "cross sectional studies" OR "cross-sectional analysis" OR "cross-sectional analyses" OR "cross sectional analysis" OR "cross-sectional analyses" OR survey OR surveys OR "retrospective studies" OR "retrospective study" OR "prospective studies" OR "prospective study" OR "observational study" OR "observational studies" OR nationwide OR populational OR population OR populations OR database OR databases) | **3239** |
| **Embase** | (sarcomas OR 'sarcoma, soft tissue'/exp OR 'sarcoma, soft tissue' OR 'sarcomas, soft tissue' OR 'soft tissue sarcoma'/exp OR 'soft tissue sarcoma' OR 'soft tissue sarcomas' OR 'sarcoma, epithelioid' OR 'epithelioid sarcoma'/exp OR 'epithelioid sarcoma' OR 'epithelioid sarcomas' OR 'sarcomas, epithelioid' OR 'sarcoma, spindle cell'/exp OR 'sarcoma, spindle cell' OR 'sarcomas, spindle cell' OR 'spindle cell sarcoma'/exp OR 'spindle cell sarcoma' OR 'spindle cell sarcomas') **AND** ('head and neck' OR 'oral and maxillofacial') **AND** (prevalence OR frequency OR frequencies OR epidemiology OR epidemiologic OR epidemiological OR occurrence OR occurrences OR incidence OR 'cross-sectional studies' OR 'cross-sectional study' OR 'cross sectional study' OR 'cross sectional studies' OR 'cross-sectional analysis' OR 'cross sectional analysis' OR 'cross-sectional analyses' OR survey OR surveys OR 'retrospective studies' OR 'retrospective study' OR 'prospective studies' OR 'prospective study' OR 'observational study' OR 'observational studies' OR nationwide OR populational OR population OR populations OR database OR databases) | **3203** |
| **Web of Science** | TS=(sarcomas OR "Sarcoma, Soft Tissue" OR "Sarcomas, Soft Tissue" OR "Soft Tissue Sarcoma" OR "Soft Tissue Sarcomas" OR "Sarcoma, Epithelioid" OR "Epithelioid Sarcoma" OR "Epithelioid Sarcomas" OR "Sarcomas, Epithelioid" OR "Sarcoma, Spindle Cell" OR "Sarcomas, Spindle Cell" OR "Spindle Cell Sarcoma" OR "Spindle Cell Sarcomas") **AND** TS=("head and neck" OR "oral and maxillofacial") **AND** TS=(prevalence OR frequency OR frequencies OR epidemiology OR epidemiologic OR epidemiological OR occurrence OR occurrences OR incidence OR "cross-sectional studies" OR "cross-sectional study" OR "cross sectional study" OR "cross sectional studies" OR "cross-sectional analysis" OR "cross-sectional analyses" OR "cross sectional analysis" OR "cross-sectional analyses" OR survey OR surveys OR "retrospective studies" OR "retrospective study" OR "prospective studies" OR "prospective study" OR "observational study" OR "observational studies" OR nationwide OR populational OR population OR populations OR database OR databases ) | **1058** |
| **Lilacs (via VHL)** | (sarcomas) **AND** ("head and neck" OR "cabeza y cuello" OR "cabeça e pescoço" OR "oral and maxillofacial" OR "oral y maxilofacial" OR "oral e maxilofacial") **AND** (“prevalence” OR “prevalência” OR “prevalencia” OR “epidemiology” OR “epidemiologia” OR “epidemiología” OR “incidence” OR “incidência” OR “incidencia”) | **75** |
| **Grey Literature** | | |
| **Google Scholar** | First 100 more relevant hits.  (sarcoma) **AND** ("head and neck" OR "oral and maxillofacial") **AND** (prevalence OR incidence OR frequency) | **100** |
| **Open Gray** | (sarcoma) **AND** ("head and neck" OR "oral and maxillofacial") **AND** (prevalence OR incidence OR frequency) | **0** |
| **ProQuest** | TI,AB(sarcoma) **AND** TI,AB("head and neck" OR "oral and maxillofacial") **AND** TI,AB(prevalence OR incidence OR frequency) | **125** |
| **Total** |  | **10297** |

**Table S2** Excluded articles and reasons for exclusion – Database (n=1007)

| References | Reasons for exclusion |
| --- | --- |
| 1. SUTOW WW. CANCER OF THE HEAD AND NECK IN CHILDREN. JAMA. 1964 Nov 2;190:414-6. doi: 10.1001/jama.1964.03070180012002. PMID: 14197990. | 2 |
| 1. Nosanchuk JS, Weatherbee L, Brody GL. Osteogenic sarcoma. Prognosis related to epiphyseal closure. JAMA. 1969 Jun 30;208(13):2439-41. doi: 10.1001/jama.208.13.2439. PMID: 5254334. | 6 |
| 1. Li FP, Fraumeni JF Jr. Rhabdomyosarcoma in children: epidemiologic study and identification of a familial cancer syndrome. J Natl Cancer Inst. 1969 Dec;43(6):1365-73. PMID: 5396222. | 2 |
| 1. Berge T, Toremalm NG. Malignant tumors of head and neck in childhood. Acta Otolaryngol. 1969 Dec;68(6):551-60. doi: 10.3109/00016486909121596. PMID: 5374160. | 2 |
| 1. Tikka U, Malmio K. Clinical and radiotherapeutic aspects of reticulum cell sarcoma. Acta Radiol Ther Phys Biol. 1969 Dec;8(6):459-70. doi: 10.3109/02841866909134472. PMID: 4983175. | 2 |
| 1. Bizer LS. Fibrosarcoma. Report of sixty-four cases. Am J Surg. 1971 May;121(5):586-7. doi: 10.1016/0002-9610(71)90146-2. PMID: 4326760. | 2 |
| 1. Ehrlich FE, Haas JE, Kiesewetter WB. Rhabdomyosarcoma in infants and children: factors affecting long-term survival. J Pediatr Surg. 1971 Oct;6(5):571-7. doi: 10.1016/0022-3468(71)90381-2. PMID: 4108254. | 2 |
| 1. Rerón E. Nowotwory głowy i szyi u dzieci w materiale krakowskiej kliniki laryngologicznej [Head and neck neoplasms in children in the records of the Department of Laryngology of the Medical Academy in Cracow]. Otolaryngol Pol. 1973;27(5):609-15. Polish. PMID: 4764872. | 7 |
| 1. Möpert S, Müller A, Rick K. Zur Therapie und Prognose maligner, mesenchymaler tumoren im Mund- und Kieferbereich919h [Therapy and prognosis of malignant mesenchymal tumors in the mouth and jaw region]. Stomatol DDR. 1973 Jul;23(7):525-32. German. PMID: 4532349. | 7 |
| 1. Wood DK, Das Gupta TK. Soft tissue sarcomas in infancy and childhood. J Surg Oncol. 1973;5(4):387-404. doi: 10.1002/jso.2930050414. PMID: 4745887. | 2 |
| 1. Chabalko JJ, Creagan ET, Fraumeni JF Jr. Epidemiology of selected sarcomas in children. J Natl Cancer Inst. 1974 Sep;53(3):675-9. doi: 10.1093/jnci/53.3.675. PMID: 4213086. | 2 |
| 1. Gutjahr P, Jung H. Zur Diagnostik, Therapie und Prognose kindlicher Kopf-Hals-Malignome: Erfahrung mit 94 Fällen [Contribution to the diagnosis, therapy and prognosis of head and neck tumors in childhood and adolescence: experience with 94 own cases (author's transl)]. Laryngol Rhinol Otol (Stuttg). 1975 Jul;54(7):591-7. German. PMID: 129628. | 7 |
| 1. Huvos AG, Higinbotham NL. Primary fibrosarcoma of bone. A clinicopathologic study of 130 patients. Cancer. 1975 Mar;35(3):837-47. doi: 10.1002/1097-0142(197503)35:3<837::aid-cncr2820350341>3.0.co;2-u. PMID: 1053940. | 2 |
| 1. Bale PM, Reye RD. Rhabdomyosarcoma in childhood. Pathology. 1975 Apr;7(2):101-11. doi: 10.3109/00313027509092704. PMID: 1153222. | 7 |
| 1. Ichikawa, A., Hirano, M., Mihashi, S. [Long term results of treatments for malignant neoplasm of the oropharynx (Japanese)](https://www.scopus.com/record/display.uri?eid=2-s2.0-0017158729&origin=reflist&sort=plf-f&cite=2-s2.0-0017158729&src=s&imp=t&sid=c99f5020dd69643fe126e27e97f5d77f&sot=cite&sdt=a&sl=0) (1976) Otologia Fukuoka, 22 (sup.3), pp. 729-736. | 7 |
| 1. Muldoon CJ. Head and neck tumours in Rhodesia. Ann R Coll Surg Engl. 1976 Mar;58(2):147-8. PMID: 178267; PMCID: PMC2490645. | 2 |
| 1. Dahl I. Atypical fibroxanthoma of the skin. A clinico-pathological study of 57 cases. Acta Pathol Microbiol Scand A. 1976 Mar;84(2):183-97. PMID: 1258644. | 2 |
| 1. Kaufman JH, Douglass HO Jr, Blake W, Moore R, Rao UN. The importance of initial presentation and treatment upon the survival of patients with chondrosarcoma. Surg Gynecol Obstet. 1977 Sep;145(3):357-63. PMID: 888055. | 7 |
| 1. Garg SK, Subbuswamy SG, Alabi JO, Lawrie JH. Dermatofibrosarcoma protuberans in Northern Nigeria: a clinico-pathological review of 17 cases. Clin Oncol. 1978 Jun;4(2):113-22. PMID: 149627. | 7 |
| 1. Neifeld JP, Berg JW, Godwin D, Saizberg AM. A retrospective epidemiologic study of pediatric fibrosarcomas. J Pediatr Surg. 1978 Dec;13(6D):735-9. doi: 10.1016/s0022-3468(78)80124-9. PMID: 731375. | 2 |
| 1. Raney RB Jr, Zimmerman RA, Bilaniuk LT, Littman P, Mandell G, Potsic W. Management of craniofacial sarcoma in childhood assisted by computed tomography. Int J Radiat Oncol Biol Phys. 1979 Apr;5(4):529-34. doi: 10.1016/0360-3016(79)90817-4. PMID: 457499. | 2 |
| 1. Magillo, Paolo. "Childhood rhabdomyosarcoma: A retrospective study of 70 cases: M. Carli, B. DeBernardi, E. Madon, G. Paolucci, M. Castello and C. Messina. Med Surg Ped 1,497–504, 1979." *Journal of Pediatric Surgery* 17.2 (1982): 219. | 7 |
| 1. Wilhelm HJ, Dietz R, Schätzle W. Primäre maligne mesenchymale Tumoren im Kopf-Hals-Bereich. Zur Problematik ihrer Diagnose und Therapie [Primary malignant mesenchymal tumors in the head and neck area--the problems in diagnosis and therapy (author's transl)]. Laryngol Rhinol Otol (Stuttg). 1980 Apr;59(4):211-20. German. PMID: 6255268. | 7 |
| 1. Harwood AR, Krajbich JI, Fornasier VL. Radiotherapy of chondrosarcoma of bone. Cancer. 1980 Jun 1;45(11):2769-77. doi: 10.1002/1097-0142(19800601)45:11<2769::aid-cncr2820451111>3.0.co;2-x. PMID: 7379008. | 9 |
| 1. Abbas JS, Holyoke ED, Moore R, Karakousis CP. The surgical treatment and outcome of soft-tissue sarcoma. Arch Surg. 1981 Jun;116(6):765-9. doi: 10.1001/archsurg.1981.01380180025006. PMID: 7235973. | 2 |
| 1. Merck C, Angervall L, Kindblom LG, Odén A. Myxofibrosarcoma. A malignant soft tissue tumor of fibroblastic-histiocytic origin. A clinicopathologic and prognostic study of 110 cases using multivariate analysis. Acta Pathol Microbiol Immunol Scand Suppl. 1983;282:1-40. PMID: 6444190. | 7 |
| 1. Bale PM, Parsons RE, Stevens MM. Diagnosis and behavior of juvenile rhabdomyosarcoma. Hum Pathol. 1983 Jul;14(7):596-611. doi: 10.1016/s0046-8177(83)80203-2. PMID: 6862431. | 2 |
| 1. Benoit Y, Delbeke MJ, Van Cauwenberge P, Kluyskens P. Malignant otolaryngological tumors in children. Acta Otorhinolaryngol Belg. 1984;38(3):288-301. PMID: 6516833. | 7 |
| 1. Cutchavaree A, Shuangshoti S, Kumut N. Neurogenic tumors of head and neck: study of 171 cases. J Med Assoc Thai. 1984 Oct;67(10):549-52. PMID: 6097624. | 7 |
| 1. Rosenberg RA, Schneider KL, Cohen NL. Head and neck presentations of acquired immunodeficiency syndrome. Laryngoscope. 1984 May;94(5 Pt 1):642-6. PMID: 6717222. | 2 |
| 1. Brooks JS, Freeman M, Enterline HT. Malignant "Triton" tumors. Natural history and immunohistochemistry of nine new cases with literature review. Cancer. 1985 Jun 1;55(11):2543-9. doi: 10.1002/1097-0142(19850601)55:11<2543::aid-cncr2820551105>3.0.co;2-4. PMID: 3922610. | 9 |
| 1. Swart JG, Klopper S, Hamersma T. Rhabdomyosarcoma of the head and neck. A review of 25 cases. S Afr J Surg. 1985 Sep;23(3):88-9. PMID: 4049151. | 7 |
| 1. Dupree WB, Langloss JM, Weiss SW. Pigmented dermatofibrosarcoma protuberans (Bednar tumor). A pathologic, ultrastructural, and immunohistochemical study. Am J Surg Pathol. 1985 Sep;9(9):630-9. doi: 10.1097/00000478-198509000-00002. PMID: 3901787. | 9 |
| 1. Franceschi D, Van den Eeckhaut J, Sonnet J. Les manifestations ORL du SIDA [ORL manifestations of AIDS]. Acta Otorhinolaryngol Belg. 1986;40(3):528-34. French. PMID: 3788554. | 7 |
| 1. Abbatucci JS, Boulier N, de Ranieri J, Mandard AM, Tanguy A, Vernhes JC, Lozier JC, Busson A. Local control and survival in soft tissue sarcomas of the limbs, trunk walls and head and neck: a study of 113 cases. Int J Radiat Oncol Biol Phys. 1986 Apr;12(4):579-86. doi: 10.1016/0360-3016(86)90066-0. PMID: 3009369. | 9 |
| 1. Greager JA, Das Gupta TK. Adult head and neck soft-tissue sarcomas. Otolaryngol Clin North Am. 1986 Aug;19(3):565-72. PMID: 3748583. | 7 |
| 1. Helsper J, Formenti S, Levine A. Initial manifestation of acquired immunodeficiency syndrome in the head and neck region. Am J Surg. 1986 Oct;152(4):403-6. doi: 10.1016/0002-9610(86)90312-0. PMID: 3766871. | 9 |
| 1. Katenkamp D. Soft tissue tumors of the head and neck. Arch Geschwulstforsch. 1987;57(2):115-27. PMID: 3592924. | 7 |
| 1. Hadderingh RJ, Tange RA, Danner SA, Eeftinck Schattenkerk JK. Otorhinolaryngological findings in AIDS patients: a study of 63 cases. Arch Otorhinolaryngol. 1987;244(1):11-4. doi: 10.1007/BF00453483. PMID: 3039952. | 2 |
| 1. Beppu Y, Fukuma H, Chuma H, Ise T, Ohira M. [Treatment of rhabdomyosarcoma at the National Cancer Center Hospital]. Gan To Kagaku Ryoho. 1987 May;14(5 Pt 2):1603-13. Japanese. PMID: 3592704. | 7 |
| 1. Watkins EB, Findlay P, Gelmann E, Lane HC, Zabell A. Enhanced mucosal reactions in AIDS patients receiving oropharyngeal irradiation. Int J Radiat Oncol Biol Phys. 1987 Sep;13(9):1403-8. doi: 10.1016/0360-3016(87)90237-9. PMID: 3624048. | 1 |
| 1. Shaver TR, Lee YT. Nonosseous sarcomas in a military hospital. J Surg Oncol. 1987 Dec;36(4):284-9. doi: 10.1002/jso.2930360414. PMID: 3695535. | 9 |
| 1. Alessi DM, Karin R, Abemayor E, Crockett DM. Granulocytic sarcomas of the head and neck. Arch Otolaryngol Head Neck Surg. 1988 Dec;114(12):1467-70. doi: 10.1001/archotol.1988.01860240117036. PMID: 3056456. | 9 |
| 1. Robinson LD, Smith RJ, Rightmire J, Torpy JM, Fernbach DJ. Head and neck malignancies in children: an age-incidence study. Laryngoscope. 1988 Jan;98(1):11-3. doi: 10.1288/00005537-198801000-00003. PMID: 3257285. | 2 |
| 1. Jürgens H, Bier V, Harms D, Beck J, Brandeis W, Etspüler G, Gadner H, Schmidt D, Treuner J, Winkler K, et al. Malignant peripheral neuroectodermal tumors. A retrospective analysis of 42 patients. Cancer. 1988 Jan 15;61(2):349-57. doi: 10.1002/1097-0142(19880115)61:2<349::aid-cncr2820610226>3.0.co;2-0. PMID: 3334970. | 2 |
| 1. Tsujimoto M, Aozasa K, Ueda T, Sakurai M, Ishiguro S, Kurata A, Ono K, Matsumoto K. Soft tissue sarcomas in Osaka, Japan (1962-1985): review of 290 cases. Jpn J Clin Oncol. 1988 Sep;18(3):231-4. PMID: 3411787. | 2 |
| 1. Olsen WL, Jeffrey RB Jr, Sooy CD, Lynch MA, Dillon WP. Lesions of the head and neck in patients with AIDS: CT and MR findings. AJR Am J Roentgenol. 1988 Oct;151(4):785-90. doi: 10.2214/ajr.151.4.785. PMID: 3262281. | 2 |
| 1. Salloum E, Flamant F, Rey A, Caillaud JM, Friedman S, Valteau D, Lemerle J. Rhabdomyosarcoma in infants under one year of age: experience of the Institut Gustave-Roussy. Med Pediatr Oncol. 1989;17(5):424-8. doi: 10.1002/mpo.2950170513. PMID: 2796858. | 2 |
| 1. Mandard AM, Petiot JF, Marnay J, Mandard JC, Chasle J, de Ranieri E, Dupin P, Herlin P, de Ranieri J, Tanguy A, et al. Prognostic factors in soft tissue sarcomas. A multivariate analysis of 109 cases. Cancer. 1989 Apr 1;63(7):1437-51. doi: 10.1002/1097-0142(19890401)63:7<1437::aid-cncr2820630735>3.0.co;2-q. PMID: 2646010. | 2 |
| 1. You MG. [Analysis of 7878 patients with tumors in the head and neck]. Zhonghua Zhong Liu Za Zhi. 1989 Jul;11(4):282-7. Chinese. PMID: 2625111. | 2 |
| 1. Temple WJ, Russell JA, Arthur K, Schachar NS, Crabtree TS, Anderson MA. Neoadjuvant treatment in conservative surgery of peripheral sarcomas. Can J Surg. 1989 Sep;32(5):361-5. PMID: 2766142. | 7 |
| 1. Ruka W, Emrich LJ, Driscoll DL, Karakousis CP. Clinical factors and treatment parameters affecting prognosis in adult high-grade soft tissue sarcomas: a retrospective review of 267 cases. Eur J Surg Oncol. 1989 Oct;15(5):411-23. PMID: 2792392. | 7 |
| 1. Bukhny AF, Belkina BM, Blinov VM, Ivanova NM, Lobanov GV, Nechushkina IV, Poliakov VG, Telepneva SI, Tseĭtlin GIa. Rabdomiosarkomy u deteĭ [Rhabdomyosarcoma in children]. Vopr Onkol. 1990;36(11):1312-6. Russian. PMID: 2281635. | 7 |
| 1. Matsuno Y, Mukai K, Itabashi M, Yamauchi Y, Hirota T, Nakajima T, Shimosato Y. Alveolar soft part sarcoma. A clinicopathologic and immunohistochemical study of 12 cases. Acta Pathol Jpn. 1990 Mar;40(3):199-205. doi: 10.1111/j.1440-1827.1990.tb03323.x. PMID: 2360459. | 9 |
| 1. Skowrońska-Gardas A. Wstepna analiza metod radioterapii w kompleksowym leczeniu dzieci chorych na miesaki tkanek miekkich [Preliminary evaluation of the methods of radiotherapy in the complex treatment of children with soft tissue sarcomas]. Nowotwory. 1990 Jul-Sep;40(3):214-20. Polish. PMID: 2123034. | 7 |
| 1. Riederer A, Bujía J, Vogel T, Wilmes E, Kastenbauer E. Manifestaciones otorrinolaringológicas en pacientes infectados por el virus del síndrome de inmunodeficiencia adquirida [Otorhinolaryngologic manifestations in patients infected by the acquired immunodeficiency virus]. Acta Otorrinolaringol Esp. 1990 Sep-Oct;41(5):277-80. Spanish. PMID: 2076306. | 7 |
| 1. Bhatia PL. Head and neck cancer in Plateau state of Nigeria. West Afr J Med. 1990 Oct-Dec;9(4):304-10. PMID: 2083210. | 7 |
| 1. Costa MJ, Weiss SW. Angiomatoid malignant fibrous histiocytoma. A follow-up study of 108 cases with evaluation of possible histologic predictors of outcome. Am J Surg Pathol. 1990 Dec;14(12):1126-32. PMID: 2174650. | 9 |
| 1. Sant'Anna GD, Franche GL, Tabajara LM, Favero RM, Kuhl G. Otorhinolaryngologic manifestations of acquired immunodeficiency syndrome. Rev Laryngol Otol Rhinol (Bord). 1991;112(1):63-6. PMID: 2052790. | 7 |
| 1. Schweitzer Vanessa G.Photodynamic therapy for treatment of AIDS-related mucocutaneous Kaposi's sarcoma (Invited Paper)*Proceedings of SPIE - The International Society for Optical Engineering*Volume 1645, Pages 10 - 321992 Optical Methods for Tumor Treatment and Detection: Mechanisms and Techniques in Photodynamic Therapy20 January 1992through 21 January 1992 Code 17479. | 9 |
| 1. De Paoli A, Bertola G, Boz G, Gherlinzoni F, Frustaci S, Fumo G, Innocente R, Trovò MG, Rossi C, Carbone A, et al. Radiation therapy and conservative surgery for soft tissue sarcomas of the extremities, torso and head and neck. Ann Oncol. 1992 Apr;3 Suppl 2:S97-101. doi: 10.1093/annonc/3.suppl_2.s97. PMID: 1622879. | 2 |
| 1. Chin T, Wei CF. Rhabdomyosarcoma in children: clinical analysis of 20 cases. J Formos Med Assoc. 1993 Jan;92(1):29-33. PMID: 8099823. | 7 |
| 1. Pradier, R. N., González, A. R., Adan, R. S., Saco, P. A., Califano, L. L., Loria, D., & Celeste, F. (1993). Sarcomas de partes blandas de cabeza y cuello en adultos. *Rev. argent. cir*, 26-35. | 7 |
| 1. Vitali ML, Ricci G, Acquaviva A, Neri E, Cominetti M, Tomassini E, Boni L, Rasore-Quartino A, Bistolfi F, De Bernardi B. Trattamento multidisciplinare del rabdomiosarcoma in età pediatrica. Esperienza pluricentrica decennale (1978-1988) in Liguria [The multidisciplinary treatment of rhabdomyosarcoma in childhood. A 10-year multicenter experience (1978-1988) in Liguria]. Pediatr Med Chir. 1993 May-Jun;15(3):279-85. Italian. PMID: 7692428. | 7 |
| 1. Kodet R, Newton WA Jr, Hamoudi AB, Asmar L, Jacobs DL, Maurer HM. Childhood rhabdomyosarcoma with anaplastic (pleomorphic) features. A report of the Intergroup Rhabdomyosarcoma Study. Am J Surg Pathol. 1993 May;17(5):443-53. doi: 10.1097/00000478-199305000-00002. PMID: 8470759. | 2 |
| 1. Vendroux J, Revol M, Banzet P. Traitement des tumeurs de Darier-Ferrand de la tête et du cou. Analyse rétrospective de vingt cas [Treatment of Darier-Ferrand tumors of the head and neck. Retrospective analysis of 20 cases]. Ann Chir Plast Esthet. 1994 Apr;39(2):184-90. French. PMID: 7872635. | 7 |
| 1. Barthélémy I, Coustal B, Michelet V, Pinsolle J, Siberchicot F, Caix P, Michelet FX. Traitement des sarcomes osseux primitifs des maxillaires [Treatment of primary bone sarcoma of the jaws]. Rev Stomatol Chir Maxillofac. 1994;95(4):302-5. French. PMID: 7939361. | 7 |
| 1. Wong K.T.; Sara Ahmad T.; Fernandez F.; Azizah N.W.K.; Swaminathan M. Alveolar soft part sarcoma with reference to unusual clinical presentations. [Asian Journal of Surgery](about:blank)Volume 17, Issue 3, Pages 283 – 2851994 | 7 |
| 1. Coffin CM, Jaszcz W, O'Shea PA, Dehner LP. So-called congenital-infantile fibrosarcoma: does it exist and what is it? Pediatr Pathol. 1994 Jan-Feb;14(1):133-50. doi: 10.3109/15513819409022033. PMID: 8159611. | 9 |
| 1. Paez, H., & Valencia, E. (1994). Análisis de 28 casos de sarcomas óseos servicio de oncología Hospital Carlos Andrade Marín. *Educ. méd. contin*, 2-6. | 7 |
| 1. Mullen JR, Zagars GK. Synovial sarcoma outcome following conservation surgery and radiotherapy. Radiother Oncol. 1994 Oct;33(1):23-30. doi: 10.1016/0167-8140(94)90082-5. PMID: 7878206. | 9 |
| 1. Birchall MA, Horner PD, Stafford ND. Changing patterns of HIV infection in otolaryngology. Clin Otolaryngol Allied Sci. 1994 Dec;19(6):473-7. doi: 10.1111/j.1365-2273.1994.tb01272.x. PMID: 7895376. | 2 |
| 1. Yang P, Grufferman S, Khoury MJ, Schwartz AG, Kowalski J, Ruymann FB, Maurer HM. Association of childhood rhabdomyosarcoma with neurofibromatosis type I and birth defects. Genet Epidemiol. 1995;12(5):467-74. doi: 10.1002/gepi.1370120504. PMID: 8557179. | 9 |
| 1. Dijkstra MD, Balm AJ, Gregor RT, Hilgers FJ, Loftus BM. Soft tissue sarcomas of the head and neck associated with surgical trauma. J Laryngol Otol. 1995 Feb;109(2):126-9. doi: 10.1017/s0022215100129457. PMID: 7706917. | 9 |
| 1. Koh CK, Ko CB, Bury HP, Wyatt EH. Dermatofibrosarcoma protuberans. Int J Dermatol. 1995 Apr;34(4):256-60. doi: 10.1111/j.1365-4362.1995.tb01591.x. PMID: 7790140. | 9 |
| 1. Costa MJ, McGlothlen L, Pierce M, Munn R, Vogt PJ. Angiomatoid features in fibrohistiocytic sarcomas. Immunohistochemical, ultrastructural, and clinical distinction from vascular neoplasms. Arch Pathol Lab Med. 1995 Nov;119(11):1065-71. PMID: 7487409. | 7 |
| 1. Michalski JM, Sur RK, Harms WB, Purdy JA. Three dimensional conformal radiation therapy in pediatric parameningeal rhabdomyosarcomas. Int J Radiat Oncol Biol Phys. 1995 Dec 1;33(5):985-91. doi: 10.1016/0360-3016(95)00551-X. PMID: 7493860. | 9 |
| 1. Riederer AP, Grein GO, Bogner JR. High prevalence of opportunistic infections in the head and neck related to human immunodeficiency virus. A prospective study of the distribution of otorhinolaryngologic disorders in 250 patients. Infection. 1996 Nov-Dec;24(6):440-6. doi: 10.1007/BF01713046. PMID: 9007592. | 2 |
| 1. Mbonde MP, Amir H, Kitinya JN. Dermatofibrosarcoma protuberans: a clinicopathological study in an African population. East Afr Med J. 1996 Jun;73(6):410-3. PMID: 8840606. | 7 |
| 1. Odell PF. Head and neck sarcomas: a review. J Otolaryngol. 1996 Feb;25(1):7-13. PMID: 8816102. | 7 |
| 1. Naka N, Ohsawa M, Tomita Y, Kanno H, Uchida A, Myoui A, Aozasa K. Prognostic factors in angiosarcoma: a multivariate analysis of 55 cases. J Surg Oncol. 1996 Mar;61(3):170-6. doi: 10.1002/(SICI)1096-9098(199603)61:3<170::AID-JSO2>3.0.CO;2-8. PMID: 8637202. | 2 |
| 1. Békássy AN, Hermans J, Gorin NC, Gratwohl A. Granulocytic sarcoma after allogeneic bone marrow transplantation: a retrospective European multicenter survey. Acute and Chronic Leukemia Working Parties of the European Group for Blood and Marrow Transplantation. Bone Marrow Transplant. 1996 May;17(5):801-8. PMID: 8733701. | 7 |
| 1. Zohar Y, Wulikh M, Aminov H, Sadov R, Gal R, Schwartz A, Herskovitz P, Kots M, Klein B. [Head and neck sarcoma]. Harefuah. 1996 Jun 2;130(11):740-4, 799. Hebrew. PMID: 8794675. | 7 |
| 1. Costa MJ, Campman SC, Davis RL, Howell LP. Fine-needle aspiration cytology of sarcoma: retrospective review of diagnostic utility and specificity. Diagn Cytopathol. 1996 Jul;15(1):23-32. doi: 10.1002/(SICI)1097-0339(199607)15:1<23::AID-DC6>3.0.CO;2-R. PMID: 8807248. | 9 |
| 1. Conill C, Alsina M, Verger E, Henríquez I. Radiation therapy in AIDS-related cutaneous Kaposi's sarcoma. Dermatology. 1997;195(1):40-2. doi: 10.1159/000245682. PMID: 9267735. | 2 |
| 1. Postma, G. N. (1997). Laryngeal manifestations of AIDS. *Current Opinion in Otolaryngology & Head and Neck Surgery*, *5*(2), 112-116. | 7 |
| 1. Kaddu S, Beham A, Cerroni L, Humer-Fuchs U, Salmhofer W, Kerl H, Soyer HP. Cutaneous leiomyosarcoma. Am J Surg Pathol. 1997 Sep;21(9):979-87. doi: 10.1097/00000478-199709000-00001. PMID: 9298873. | 7 |
| 1. Hirsch RJ, Yousem DM, Loevner LA, Montone KT, Chalian AA, Hayden RE, Weinstein GS. Synovial sarcomas of the head and neck: MR findings. AJR Am J Roentgenol. 1997 Oct;169(4):1185-8. doi: 10.2214/ajr.169.4.9308488. PMID: 9308488. | 2 |
| 1. Kimber C, Michalski A, Spitz L, Pierro A. Primitive neuroectodermal tumours: anatomic location, extent of surgery, and outcome. J Pediatr Surg. 1998 Jan;33(1):39-41. doi: 10.1016/s0022-3468(98)90357-8. PMID: 9473096. | 2 |
| 1. Jaworski W, Sawicz-Birkowska K, Jaworska M, Kazanowska B, Chybicka A, Czernik J. Nietypowe miesaki wieku dzieciecego zlokalizowane w obrebie głowy i szyi [Atypical sarcomas localized within a head and neck in children]. Wiad Lek. 1998;51 Suppl 4:157-62. Polish. PMID: 10731961. | 7 |
| 1. SEO, J. J., WON, Y. H., KIM, S. J., LEE, S. C., & CHUN, I. K. (1998). A clinical observation of cutaneous malignant tumors over 10 years (1987-1996, Chonnam Province). *Korean Journal of Dermatology*, 812-819. | 7 |
| 1. Su W.-F.,Ho C.-Y.,Lin C.-Z.Rhabdomyosarcoma of the nose and paranasal sinuses. Journal of the Otolaryngological Society of the Republic of China 1998 33:3 (244-249) | 7 |
| 1. Oujilal, A., El Alami, M. N., Jortay, A., Jazouli, N., Lazrak, A., Benchekroun, L., ... & Kzadri, M. (1998). Parameningeal rhabdomyosarcoma. Report of 19 cases. *Acta oto-rhino-laryngologica belgica*, *52*(3), 235-240. | 7 |
| 1. Jonsson, N., Zimmerman, M., Chidzonga, M. M., & Jonsson, K. (1998). Oral manifestations in 100 Zimbabwean HIV/AIDS patients referred to a specialist centre. *The Central African Journal of Medicine*, *44*(2), 31-34. | 7 |
| 1. Ballo MT, Zagars GK, Pisters P, Pollack A. The role of radiation therapy in the management of dermatofibrosarcoma protuberans. Int J Radiat Oncol Biol Phys. 1998 Mar 1;40(4):823-7. doi: 10.1016/s0360-3016(97)00895-x. PMID: 9531366. | 2 |
| 1. Meis-Kindblom JM, Kindblom LG. Angiosarcoma of soft tissue: a study of 80 cases. Am J Surg Pathol. 1998 Jun;22(6):683-97. doi: 10.1097/00000478-199806000-00005. PMID: 9630175. | 9 |
| 1. Talati N, Pervez S. Soft tissue sarcomas: pattern diagnosis or entity? J Pak Med Assoc. 1998 Sep;48(9):272-5. PMID: 10028795. | 2 |
| 1. Bradford WB, Jose BO, Butler D, Lindberg RD, Paris K, Spanos WJ Jr, Patel CC, Bertolone SJ. Rhabdomyosarcoma in children--a ten year review. J Ky Med Assoc. 1998 Oct;96(10):399-402. PMID: 9803061. | 7 |
| 1. Akyüz C, Sancak R, Büyükpamukçu N, Atahan L, Göğüş S, Kutluk T, Büyükpamukçu M. Turkish experience with rhabdomyosarcoma: an analysis of 255 patients for 20 years. Turk J Pediatr. 1998 Oct-Dec;40(4):491-501. PMID: 10028857. | 7 |
| 1. Fanburg-Smith JC, Miettinen M. Angiomatoid "malignant" fibrous histiocytoma: a clinicopathologic study of 158 cases and further exploration of the myoid phenotype. Hum Pathol. 1999 Nov;30(11):1336-43. doi: 10.1016/s0046-8177(99)90065-5. PMID: 10571514. | 7 |
| 1. Cahali M.B.,Sennes L.U.,Murakami M.S.,D'Antonio W.E.P.A.,Ikino C.M.Y.,Santoro P.P., De Medeiros I.R.T.,Butugan O. Malignant nasosinusal tumors: Presentation spectrum in the latest 15 years. Revista Brasileira de Otorrinolaringologia 1999 65:4 (296-300) | 7 |
| 1. Vaccani JP, Forte V, de Jong AL, Taylor G. Ewing's sarcoma of the head and neck in children. Int J Pediatr Otorhinolaryngol. 1999 May 25;48(3):209-16. doi: 10.1016/s0165-5876(99)00030-0. PMID: 10402117. | 9 |
| 1. de Saint Aubain Somerhausen N, Fletcher CD. Leiomyosarcoma of soft tissue in children: clinicopathologic analysis of 20 cases. Am J Surg Pathol. 1999 Jul;23(7):755-63. doi: 10.1097/00000478-199907000-00002. PMID: 10403297. | 9 |
| 1. Kao GD, Devine P, Mirza N. Oral cavity and oropharyngeal tumors in human immunodeficiency virus-positive patients: acute response to radiation therapy. Arch Otolaryngol Head Neck Surg. 1999 Aug;125(8):873-6. doi: 10.1001/archotol.125.8.873. PMID: 10448734. | 9 |
| 1. Carew JF, Singh B, Kraus DH. Hemangiopericytoma of the head and neck. Laryngoscope. 1999 Sep;109(9):1409-11. doi: 10.1097/00005537-199909000-00009. PMID: 10499045. | 5 |
| 1. Ben Arush MW, Nahum MP, Meller I, Ben Itzhak O, Kuten A, el Hassid R, Linn S. The role of chemotherapy in childhood soft tissue sarcomas other than rhabdomyosarcomas: experience of the Northern Israel Oncology Center. Pediatr Hematol Oncol. 1999 Sep-Oct;16(5):397-406. doi: 10.1080/088800199276949. PMID: 10505315. | 2 |
| 1. Wang SJ, Borges A, Lufkin RB, Sercarz JA, Wang MB. Chondroid tumors of the larynx: computed tomography findings. Am J Otolaryngol. 1999 Nov-Dec;20(6):379-82. doi: 10.1016/s0196-0709(99)90077-7. PMID: 10609482. | 9 |
| 1. Espat NJ, Lewis JJ, Woodruff JM, Antonescu C, Xia J, Leung D, Brennan MF. Confirmed angiosarcoma: prognostic factors and outcome in 50 prospectively followed patients. Sarcoma. 2000;4(4):173-7. doi: 10.1155/2000/575781. PMID: 18521298; PMCID: PMC2395443. | 2 |
| 1. Sharov TA. Klinicheskie proiavleniia i diagnostika rabdomiosarkomy razlichnoĭ lokalizatsii u deteĭ [Clinical manifestations and diagnosis of rhabdomyosarcoma of childhood at different sites]. Vestn Ross Akad Med Nauk. 2000;(7):16-9. Russian. PMID: 10961142. | 7 |
| 1. Sigel JE, Bergfeld WF, Goldblum JR. A morphologic study of dermatofibrosarcoma protuberans: expansion of a histologic profile. J Cutan Pathol. 2000 Apr;27(4):159-63. doi: 10.1034/j.1600-0560.2000.027004159.x. PMID: 10774935. | 2 |
| 1. Karakas Z, Agaoglu L, Biner B, Devecioglu O, Anak S, Yalman N, Unuvar A, Celik A, Gedikoglu G. Results of rhabdomyosarcoma treatment in a developing country. Acta Med Okayama. 2000 Aug;54(4):173-7. doi: 10.18926/AMO/32277. PMID: 10985177. | 2 |
| 1. Kim EE, Valenzuela RF, Kumar AJ, Raney RB, Eftekari F. Imaging and clinical spectrum of rhabdomyosarcoma in children. Clin Imaging. 2000 Sep-Oct;24(5):257-62. doi: 10.1016/s0899-7071(00)00222-9. PMID: 11331151. | 9 |
| 1. Sun LM, Wang CJ, Huang CC, Leung SW, Chen HC, Fang FM, Huang EY, Lee SP. Dermatofibrosarcoma protuberans: treatment results of 35 cases. Radiother Oncol. 2000 Nov;57(2):175-81. doi: 10.1016/s0167-8140(00)00228-0. PMID: 11054521. | 9 |
| 1. Migotto Cabrera, W. H., León Rivera, M., León Atoche, L., Vigil Rojas, C., Velarde Galdos, R., Iberico Ocampo, W., & Abugattas Saba, J. E. (2000). Sarcomas de tejido blandos en el Instituto de Enfermedades Neoplásicas. *Acta cancerol*, 37-43. | 7 |
| 1. Portera CA Jr, Ho V, Patel SR, Hunt KK, Feig BW, Respondek PM, Yasko AW, Benjamin RS, Pollock RE, Pisters PW. Alveolar soft part sarcoma: clinical course and patterns of metastasis in 70 patients treated at a single institution. Cancer. 2001 Feb 1;91(3):585-91. doi: 10.1002/1097-0142(20010201)91:3<585::aid-cncr1038>3.0.co;2-0. PMID: 11169942. | 9 |
| 1. Goldenberg D, Golz A, Fradis M, Mârtu D, Netzer A, Joachims HZ. Malignant tumors of the nose and paranasal sinuses: a retrospective review of 291 cases. Ear Nose Throat J. 2001 Apr;80(4):272-7. PMID: 11338653. | 2 |
| 1. Tan LL, Ahmad K, Kareem BA, Harwant S. Pattern of primary musculoskeletal sarcomas referred to Institute of Radiotherapy and Oncology, Hospital Kuala Lumpur, 1995-1999. Med J Malaysia. 2001 Jun;56 Suppl C:52-6. PMID: 11814250. | 7 |
| 1. Loos BM, Wieneke JA, Thompson LD. Laryngeal angiosarcoma: a clinicopathologic study of five cases with a review of the literature. Laryngoscope. 2001 Jul;111(7):1197-202. doi: 10.1097/00005537-200107000-00012. PMID: 11568541. | 9 |
| 1. Xiao Q, Huang S, Guo Z, Huang Z. [Eight cases of rhabdomyosarcoma in head and neck]. Lin Chuang Er Bi Yan Hou Ke Za Zhi. 2001 Jul;15(7):311-2. Chinese. PMID: 12541790. | 9 |
| 1. Völter C, Baier G, Hoppe F, Schwager K, Helms J. Diagnostik, Therapie und Ergebnisse in der Behandlung maligner Schädelbasistumoren1 [Diagnosis, treatment and results of malignant skull base tumours]. Laryngorhinootologie. 2001 Sep;80(9):512-6. German. doi: 10.1055/s-2001-17084. PMID: 11555782. | 9 |
| 1. Funk GF, Karnell LH, Robinson RA, Zhen WK, Trask DK, Hoffman HT. Presentation, treatment, and outcome of oral cavity cancer: a National Cancer Data Base report. Head Neck. 2002 Feb;24(2):165-80. doi: 10.1002/hed.10004. PMID: 11891947. | 1 |
| 1. Saha, S., Saha, V. P., & Chattopadhyay, S. (2002). Orbital and paraorbital tumors-clinicopathological profile and surgical management. *Indian Journal of Otolaryngology and Head and Neck Surgery*, *54*, 117-122 | 2 |
| 1. Cho KJ, Khang SK, Lee SS, Koh JS, Chung JH, Lee YS, Shim YS. Cancers of the upper aerodigestive tract in Korea. J Korean Med Sci. 2002 Feb;17(1):18-22. doi: 10.3346/jkms.2002.17.1.18. PMID: 11850583; PMCID: PMC3054836. | 2 |
| 1. Bridge JA, Liu J, Qualman SJ, Suijkerbuijk R, Wenger G, Zhang J, Wan X, Baker KS, Sorensen P, Barr FG. Genomic gains and losses are similar in genetic and histologic subsets of rhabdomyosarcoma, whereas amplification predominates in embryonal with anaplasia and alveolar subtypes. Genes Chromosomes Cancer. 2002 Mar;33(3):310-21. doi: 10.1002/gcc.10026. PMID: 11807989. | 9 |
| 1. Al-Khateeb T, Bataineh AB. Rhabdomyosarcoma of the oral and maxillofacial region in Jordanians: a retrospective analysis. Oral Surg Oral Med Oral Pathol Oral Radiol Endod. 2002 May;93(5):580-5. doi: 10.1067/moe.2002.123860. PMID: 12075208. | 9 |
| 1. Sylvestre Begnis, G., Sylvestre Begnis, D., Viú Armengol, R., Grunfeld, P., & Rastelli, C. (2002). Sarcomas de partes blandas: resultados de tratamiento y factores pronósticos. *Rev. argent. cir*, 199-206. | 7 |
| 1. Espat NJ, Lewis JJ, Leung D, Woodruff JM, Antonescu CR, Shia J, Brennan MF. Conventional hemangiopericytoma: modern analysis of outcome. Cancer. 2002 Oct 15;95(8):1746-51. doi: 10.1002/cncr.10867. PMID: 12365023. | 5 |
| 1. Kartha SS, Bumpous JM. Synovial cell sarcoma: diagnosis, treatment, and outcomes. Laryngoscope. 2002 Nov;112(11):1979-82. doi: 10.1097/00005537-200211000-00013. PMID: 12439166. | 9 |
| 1. Simon JH, Paulino AC, Ritchie JM, Mayr NA, Buatti JM. Presentation, prognostic factors and patterns of failure in adult rhabdomyosarcoma. Sarcoma. 2003;7(1):1-7. doi: 10.1080/1357714031000114147. PMID: 18521362; PMCID: PMC2395512. | 2 |
| 1. Bhattacharyya N. Survival and staging characteristics for non-squamous cell malignancies of the maxillary sinus. Arch Otolaryngol Head Neck Surg. 2003 Mar;129(3):334-7. doi: 10.1001/archotol.129.3.334. PMID: 12622544. | 1 |
| 1. Neves, B. M., Pontes, P. A. D. L., Caran, E. M., Figueiredo, C., Weckx, L. L., & Fujita, R. R. (2003). Rabdomiosarcoma de cabeça e pescoço na infância. *Revista Brasileira de Otorrinolaringologia*, *69*, 24-28. | 8 |
| 1. Neville H, Corpron C, Blakely ML, Andrassy R. Pediatric neurofibrosarcoma. J Pediatr Surg. 2003 Mar;38(3):343-6; discussion 343-6. doi: 10.1053/jpsu.2003.50105. PMID: 12632346. | 9 |
| 1. Wu GH, Chen FJ, Zeng ZY, Li H, Lin GW, Song M, Wei MW, Xu GP, Yang AK, Chen WK. [Treatment of second primary malignant tumor induced by radiotherapy]. Zhonghua Zhong Liu Za Zhi. 2003 May;25(3):275-7. Chinese. PMID: 12839694. | 2 |
| 1. Heffner DK. Prognostic factors in head and neck rhabdomyosarcoma. Head Neck. 2003 May;25(5):416-7; author reply 417. doi: 10.1002/hed.10270. PMID: 12692881. | 4 |
| 1. Jones DA, Dillard SC, Bradford CR, Wolf GT, Prince ME. Cartilaginous tumours of the larynx. J Otolaryngol. 2003 Oct;32(5):332-7. doi: 10.2310/7070.2003.11351. PMID: 14974866. | 9 |
| 1. Khatri VP, Galante JM, Bold RJ, Schneider PD, Ramsamooj R, Goodnight JE Jr. Dermatofibrosarcoma protuberans: reappraisal of wide local excision and impact of inadequate initial treatment. Ann Surg Oncol. 2003 Nov;10(9):1118-22. doi: 10.1245/aso.2003.03.581. PMID: 14597453. | 9 |
| 1. Al-Maghrabi JA, Al-Ghamdi AS, Elhakeem HA. Pattern of skin cancer in Southwestern Saudi Arabia. Saudi Med J. 2004 Jun;25(6):776-9. PMID: 15195210. | 2 |
| 1. Amusa YB, Olabanji JK, Akinpelu VO, Olateju SO, Agbakwuru EA, Ndukwe N, Fatusi OA, Ojo OS. Pattern of head and neck malignant tumours in a Nigerian teaching hospital--a ten year review. West Afr J Med. 2004 Oct-Dec;23(4):280-5. doi: 10.4314/wajm.v23i4.28141. Erratum in: West Afr J   Med. 2005 Apr-Jun;24(2):183. Ogundipe, OV [removed]; Akinpelu, VO [added]; Ojo, OS [added]. PMID: 15730084. | 2 |
| 1. Ondzotto G, Ibara JR, Mowondabeka P, Galiba J. Les manifestations ORL et cervico-faciales de l'infection par le VIH en zone tropicale. A propos de 253 cas congolais [Cervico-facial and ENT symptoms due to HIV infection in tropical area. About 253 Congolese cases]. Bull Soc Pathol Exot. 2004 Feb;97(1):59-63. French. PMID: 15104161. | 1 |
| 1. Ballo MT, Zagars GK, Cormier JN, Hunt KK, Feig BW, Patel SR, Pisters PW. Interval between surgery and radiotherapy: effect on local control of soft tissue sarcoma. Int J Radiat Oncol Biol Phys. 2004 Apr 1;58(5):1461-7. doi: 10.1016/j.ijrobp.2003.09.079. PMID: 15050324. | 2 |
| 1. Bezabih M. Superficial malignant neoplasms in southwestern Ethiopia: a cytopathological approach. Diagn Cytopathol. 2004 Nov;31(5):347-51. doi: 10.1002/dc.20129. PMID: 15468117. | 1 |
| 1. Sale KA, Wallace DI, Girod DA, Tsue TT. Radiation-induced malignancy of the head and neck. Otolaryngol Head Neck Surg. 2004 Nov;131(5):643-5. doi: 10.1016/j.otohns.2004.05.012. PMID: 15523441. | 9 |
| 1. Skitarelic N.Clinical manifestations of infection with human immunodeficiency virus (HIV) in otorhinolaryngology.Medica Jadertina 2005 35:3-4 (67-74) | 7 |
| 1. Palacios E, Restrepo S, Mastrogiovanni L, Lorusso GD, Rojas R. Sinonasal hemangiopericytomas: clinicopathologic and imaging findings. Ear Nose Throat J. 2005 Feb;84(2):99-102. PMID: 15794546. | 9 |
| 1. Drut R, Drut RM, Pollono D, Tomarchio S, Ibáñez O, Urrutia A, Ripoll MC. Fine-needle aspiration biopsy in pediatric oncology patients: a review of experience with 829 patients (899 biopsies). J Pediatr Hematol Oncol. 2005 Jul;27(7):370-6. doi: 10.1097/01.mph.0000173177.40894.8d. PMID: 16012326. | 2 |
| 1. Kim DH, Murovic JA, Tiel RL, Moes G, Kline DG. A series of 146 peripheral non-neural sheath nerve tumors: 30-year experience at Louisiana State University Health Sciences Center. J Neurosurg. 2005 Feb;102(2):256-66. doi: 10.3171/jns.2005.102.2.0256. PMID: 15739553. | 2 |
| 1. Shah H, Pervez S. Immunophenotypic characterization of high grade pleomorphic sarcomas: a demographic and immunohistochemical study in a major referral center of Pakistan. J Pak Med Assoc. 2005 Mar;55(3):101-4. PMID: 15852744. | 7 |
| 1. Lee SY, Lim YC, Song MH, Seok JY, Lee WS, Choi EC. Chondrosarcoma of the head and neck. Yonsei Med J. 2005 Apr 30;46(2):228-32. doi: 10.3349/ymj.2005.46.2.228. PMID: 15861495; PMCID: PMC2823018. | 9 |
| 1. Kanhere HA, Pai PS, Neeli SI, Kantharia R, Saoji RR, D'cruz AK. Alveolar soft part sarcoma of the head and neck. Int J Oral Maxillofac Surg. 2005 May;34(3):268-72. doi: 10.1016/j.ijom.2004.05.008. PMID: 15741035. | 9 |
| 1. Kim HS, Lee HK, Weon YC, Kim HJ. Alveolar soft-part sarcoma of the head and neck: clinical and imaging features in five cases. AJNR Am J Neuroradiol. 2005 Jun-Jul;26(6):1331-5. PMID: 15956492; PMCID: PMC8149073. | 9 |
| 1. Bieań E, Irga N, Stachowicz-Stencel T, Stefanowicz J, Połczyńska K, Sierota D, Szołkiewicz A, Adamkiewicz-Drozyńska E, Birkholz D, Zawitkowska-Klaczytska J, Dudkiewicz E, Katski K, Nurzyńska-Flak J, Balcerska A, Stankiewicz C, Kowalczyk JR. Nowotwory złośliwe o lokalizacji okołooponowej u dzieci w materiale dwóch ośrodków onkologicznych--trudności diagnostyczne i terapeutyczne [Malignant neoplasms localised in the parameningeal region in children treated at two Polish oncological centres-diagnostic and therapeutic dilemmas]. Med Wieku Rozwoj. 2005 Jul-Sep;9(3 Pt 2):449-61. Polish. PMID: 16719157. | 7 |
| 1. Stachowicz-Stencel T, Bień E, Stefanowicz J, Połczytńska K, Sierota D, Szołkiewicz A, Drozyńska E, Kosiak W, Stankiewicz C, Pietniczka M, Kukwa A, Czauderna P, Balcerska A. Trudności diagnostyczne i terapeutyczne w przypadkach miesaków tkanek mieskkich zlokalizowanych w obrebie głowy i szyi u dzieci--doświadczenia własne [Diagnostic and therapeutic difficulties in soft tissue sarcomas localized in nonparameningeal head and neck region--own experiences]. Med Wieku Rozwoj. 2005 Jul-Sep;9(3 Pt 2):487-94. Polish. PMID: 16719161. | 9 |
| 1. Gil Z, Constantini S, Spektor S, Abergel A, Khafif A, Beni-Adani L, Leonor TL, DeRowe A, Fliss DM. Skull base approaches in the pediatric population. Head Neck. 2005 Aug;27(8):682-9. doi: 10.1002/hed.20226. PMID: 15957193. | 9 |
| 1. Fiore M, Miceli R, Mussi C, Lo Vullo S, Mariani L, Lozza L, Collini P, Olmi P, Casali PG, Gronchi A. Dermatofibrosarcoma protuberans treated at a single institution: a surgical disease with a high cure rate. J Clin Oncol. 2005 Oct 20;23(30):7669-75. doi: 10.1200/JCO.2005.02.5122. PMID: 16234529. | 2 |
| 1. Oghalai JS, Buxbaum JL, Jackler RK, McDermott MW. Skull base chondrosarcoma originating from the petroclival junction. Otol Neurotol. 2005 Sep;26(5):1052-60. doi: 10.1097/01.mao.0000185076.65822.f7. PMID: 16151358. | 6 |
| 1. Isoda H, Imai M, Inagawa S, Miura K, Sakahara H. Magnetic resonance imaging findings of angiosarcoma of the scalp. J Comput Assist Tomogr. 2005 Nov-Dec;29(6):858-62. doi: 10.1097/01.rct.0000183274.70422.0a. PMID: 16272865. | 9 |
| 1. Dagan R, Morris CG, Zlotecki RA, Scarborough MT, Mendenhall WM. Radiotherapy in the treatment of dermatofibrosarcoma protuberans. Am J Clin Oncol. 2005 Dec;28(6):537-9. doi: 10.1097/01.coc.0000171278.69291.64. PMID: 16317260. | 9 |
| 1. Suita S, Noguchi S, Takamatsu H, Mizote H, Nagasaki A, Inomata Y, Hara T, Okamura J, Miyazaki S, Kawakami K, Eguchi H, Tsuneyoshi M; Committee for Pediatric Solid Malignant Tumors in the Kyushu Area. Clinical characteristics and the prognosis of rhabdomyosarcoma - a report from the Study Group for Pediatric Solid Malignant Tumors in the Kyushu Area, Japan. Eur J Pediatr Surg. 2005 Dec;15(6):409-13. doi: 10.1055/s-2005-872927. PMID: 16418958. | 2 |
| 1. [Andrade, Wesley Pereira](https://pesquisa.bvsalud.org/portal/?lang=pt&q=au:%22Andrade,%20Wesley%20Pereira%22); [Ribeiro, Héber Salvador de Castro](https://pesquisa.bvsalud.org/portal/?lang=pt&q=au:%22Ribeiro,%20H%C3%A9ber%20Salvador%20de%20Castro%22); [Barrozo, Abner Jorge Jácome](https://pesquisa.bvsalud.org/portal/?lang=pt&q=au:%22Barrozo,%20Abner%20Jorge%20J%C3%A1come%22); [Ikeda, Mauro Kasuo](https://pesquisa.bvsalud.org/portal/?lang=pt&q=au:%22Ikeda,%20Mauro%20Kasuo%22); [David Filho, Waldec Jorge](https://pesquisa.bvsalud.org/portal/?lang=pt&q=au:%22David%20Filho,%20Waldec%20Jorge%22). Sarcomas de partes moles de cabeça e pescoço / Sarcomas of the head and neck.In. Kowalski, Luiz Paulo; Guimarães, Gustavo Cardoso; Salvajoli, João Victor; Feher, Olavo; Antoneli, Célia Beatriz Gianotti. Manual de Condutas Diagnósticas e Terapêuticas em Oncologia. São Paulo, Âmbito Editores, 3 ed; 2006. p.425-429. | 4 |
| 1. Wanebo JE, Bristol RE, Porter RR, Coons SW, Spetzler RF. Management of cranial base chondrosarcomas. Neurosurgery. 2006 Feb;58(2):249-55; discussion 249-55. doi: 10.1227/01.NEU.0000194834.74873.FB. PMID: 16462478. | 6 |
| 1. Abd El-Aal HH, Habib EE, Mishrif MM. Rhabdomyosarcoma: the experience of the pediatric unit of Kasr El-Aini Center of Radiation Oncology and Nuclear Medicine (NEMROCK) (from January 1992 to January 2001). J Egypt Natl Canc Inst. 2006 Mar;18(1):51-60. PMID: 17237856. | 2 |
| 1. Onyango JF, Awange DO, Njiru A, Macharia IM. Pattern of occurrence of head and neck cancer presenting at Kenyatta National Hospital, Nairobi. East Afr Med J. 2006 May;83(5):288-91. doi: 10.4314/eamj.v83i5.9435. PMID: 16866224. | 7 |
| 1. Timmermann B, Schuck A, Niggli F, Weiss M, Lomax A, Goitein G. Protonentherapie mit "Spot-Scanning" bei Rhabdomyosarkomen im frühen Kindesalter: Erste Erfahrungen am PSI ["Spot-scanning" proton therapy for rhabdomyosarcomas of early childhood. First experiences at PSI]. Strahlenther Onkol. 2006 Nov;182(11):653-9. German. doi: 10.1007/s00066-006-1592-y. PMID: 17072523. | 9 |
| 1. Kazanowska B, Reich A, Stegmaier S, Békássy AN, Leuschner I, Chybicka A, Koscielniak E. Pax3-fkhr and pax7-fkhr fusion genes impact outcome of alveolar rhabdomyosarcoma in children. Fetal Pediatr Pathol. 2007 Jan-Feb;26(1):17-31. doi: 10.1080/15513810701394702. PMID: 17613043. | 2 |
| 1. Svarvar C, Böhling T, Berlin O, Gustafson P, Follerås G, Bjerkehagen B, Domanski HA, Sundby Hall K, Tukiainen E, Blomqvist C; Scandinavian Sarcoma Group Leiomyosarcoma Working Group. Clinical course of nonvisceral soft tissue leiomyosarcoma in 225 patients from the Scandinavian Sarcoma Group. Cancer. 2007 Jan 15;109(2):282-91. doi: 10.1002/cncr.22395. PMID: 17154171. | 2 |
| 1. KIM, A. Y., ROH, J. L., KIM, J. M., RHA, K. S., & PARK, C. I. (2007). Association of Epstein-Barr virus and head and neck cancer in an endemic area. *Korean Journal of Otolaryngology-Head and Neck Surgery*, 235-239. | 7 |
| 1. Lewandowski L, Osmola K, Nowaczyk M. Nowotwory złośiwe jamy ustnej i szyi w materiale kliniki chirurgii szczekowo-twarzowej w Poznaniu w okresie 2002-2004 [Malignant tumors of the oral cavity and neck in clinic of maxillo-facial surgery in Poznań from 2002-2004]. Otolaryngol Pol. 2007;61(3):286-9. Polish. doi: 10.1016/S0030-6657(07)70427-4. PMID: 17847782. | 1 |
| 1. Chiu CS, Lin CY, Kuo TT, Kuan YZ, Chen MJ, Ho HC, Yang LC, Chen CH, Shih IH, Hong HS, Chuang YH. Malignant cutaneous tumors of the scalp: a study of demographic characteristics and histologic distributions of 398 Taiwanese patients. J Am Acad Dermatol. 2007 Mar;56(3):448-52. doi: 10.1016/j.jaad.2006.08.060. Epub 2006 Dec 1. PMID: 17141358. | 1 |
| 1. Paulino AC, Nguyen TX, Mai WY. An analysis of primary site control and late effects according to local control modality in non-metastatic Ewing sarcoma. Pediatr Blood Cancer. 2007 Apr;48(4):423-9. doi: 10.1002/pbc.20754. PMID: 16421909. | 9 |
| 1. Hosoi H, Teramukai S, Matsumoto Y, Tsuchiya K, Iehara T, Hara J, Mitsui T, Kaneko M, Hatae Y, Hayashi Y, Mabuchi O, Adachi N, Morikawa Y, Nishimura S, Kumagai M, Takamatsu H, Sawada T, Sugimoto T. A review of 331 rhabdomyosarcoma cases in patients treated between 1991 and 2002 in Japan. Int J Clin Oncol. 2007 Apr;12(2):137-45. doi: 10.1007/s10147-006-0638-6. Epub 2007 Apr 27. PMID: 17443282. | 2 |
| 1. Nagano T, Yamada Y, Ikeda T, Kanki H, Kamo T, Nishigori C. Docetaxel: a therapeutic option in the treatment of cutaneous angiosarcoma: report of 9 patients. Cancer. 2007 Aug 1;110(3):648-51. doi: 10.1002/cncr.22822. PMID: 17582627. | 9 |
| 1. Fleshman R, Mayerson J, Wakely PE Jr. Fine-needle aspiration biopsy of high-grade sarcoma: a report of 107 cases. Cancer. 2007 Dec 25;111(6):491-8. doi: 10.1002/cncr.23122. PMID: 17941014. | 9 |
| 1. Wu X, Li P, Xie L, Zhang X, Wang W, Xiao J, Wang F. [Clinical analysis of 13 cases of embryonal rhabdomyosarcoma in the nose]. Lin Chuang Er Bi Yan Hou Tou Jing Wai Ke Za Zhi. 2008 Apr;22(8):338-41. Chinese. PMID: 18595516. | 7 |
| 1. Mandonnet E, Kolb F, Tran Ba Huy P, George B. Spectrum of skull base tumors in children and adolescents: a series of 42 patients and review of the literature. Childs Nerv Syst. 2008 Jun;24(6):699-706. doi: 10.1007/s00381-008-0580-1. Epub 2008 Mar 15. PMID: 18343930. | 6 |
| 1. Zhang HT, Guo L, Su Q. [Clinicopathologic analysis of spindle cell rhabdomyosarcoma: report of 8 cases]. Zhonghua Zhong Liu Za Zhi. 2008 Feb;30(2):141-3. Chinese. PMID: 18646700. | 9 |
| 1. Resto VA, Chan AW, Deschler DG, Lin DT. Extent of surgery in the management of locally advanced sinonasal malignancies. Head Neck. 2008 Feb;30(2):222-9. doi: 10.1002/hed.20681. PMID: 17902164. | 1 |
| 1. Kirova YM, De Rycke Y, Gambotti L, Pierga JY, Asselain B, Fourquet A; Institut Curie Breast Cancer Study Group. Second malignancies after breast cancer: the impact of different treatment modalities. Br J Cancer. 2008 Mar 11;98(5):870-4. doi: 10.1038/sj.bjc.6604241. Epub 2008 Feb 12. PMID: 18268495; PMCID: PMC2266852. | 1 |
| 1. Yuan XJ, Chan GC, Chan SK, Shek TW, Kwong DL, Wei WI, Ha SY, Chiang AK. Treatment outcome of rhabdomyosarcoma in Hong Kong Chinese children. Hong Kong Med J. 2008 Apr;14(2):116-23. PMID: 18382018. | 9 |
| 1. De Smet S, Vandermeeren L, Christiaens MR, Samson I, Stas M, Van Limbergen E, De Wever I. Radiation-induced sarcoma: analysis of 46 cases. Acta Chir Belg. 2008 Sep-Oct;108(5):574-9. doi: 10.1080/00015458.2008.11680288. PMID: 19051469. | 9 |
| 1. Franco Gutiérrez V, Llorente Pendás JL, Coca Pelaz A, Cabanillas Farpón R, Suárez Nieto C. Radiation-induced sarcomas of the head and neck. J Craniofac Surg. 2008 Sep;19(5):1287-91. doi: 10.1097/SCS.0b013e3181869df1. PMID: 18812853. | 9 |
| 1. Paradisi A, Abeni D, Rusciani A, Cigna E, Wolter M, Scuderi N, Rusciani L, Kaufmann R, Podda M. Dermatofibrosarcoma protuberans: wide local excision vs. Mohs micrographic surgery. Cancer Treat Rev. 2008 Dec;34(8):728-36. doi: 10.1016/j.ctrv.2008.06.002. Epub 2008 Aug 5. PMID: 18684568. | 9 |
| 1. Wang JN, Li RY, Cui MY. [Radiation-induced maxillary malignancies: complications of radiotherapy treatment for head-neck malignant tumors]. Zhonghua Kou Qiang Yi Xue Za Zhi. 2008 Dec;43(12):713-5. Chinese. PMID: 19134344. | 1 |
| 1. Oakley GJ, Fuhrer K, Seethala RR. Brachyury, SOX-9, and podoplanin, new markers in the skull base chordoma vs chondrosarcoma differential: a tissue microarray-based comparative analysis. Mod Pathol. 2008 Dec;21(12):1461-9. doi: 10.1038/modpathol.2008.144. Epub 2008 Sep 26. PMID: 18820665; PMCID: PMC4233461. | 6 |
| 1. Acioğlu E, Cansiz H, Mercan H, Dervişoğlu S. Head and neck hemangiopericytomas: diagnostic contradictions. J Craniofac Surg. 2009 May;20(3):930-5. doi: 10.1097/SCS.0b013e3181a28a2e. PMID: 19461334. | 5 |
| 1. Chowdhury T, Barnacle A, Haque S, Sebire N, Gibson S, Anderson J, Roebuck D. Ultrasound-guided core needle biopsy for the diagnosis of rhabdomyosarcoma in childhood. Pediatr Blood Cancer. 2009 Sep;53(3):356-60. doi: 10.1002/pbc.22059. PMID: 19418540. | 9 |
| 1. Chan, A. Y. K., Luk, N. M., & Lee, K. C. (2009). Dermatofibrosarcoma protuberans: a report of 36 cases in Hong Kong and review of the literature. *Hong Kong Journal of Dermatology and Venereology*, *17*, 6-11. | 9 |
| 1. Laco J, Mentzel T, Hornychova H, Kohout A, Jirousek Z, Ryska A. Atypical lipomatous tumors of the tongue: report of six cases. Virchows Arch. 2009 Oct;455(4):383-8. doi: 10.1007/s00428-009-0835-6. Epub 2009 Oct 9. PMID: 19816710. | 9 |
| 1. Gao N, Li Y, Li LJ, Wen YM. Clinical analysis of head and neck cancer cases in south-west China 1953 - 2002. J Int Med Res. 2009 Jan-Feb;37(1):189-97. doi: 10.1177/147323000903700123. PMID: 19215690. | 2 |
| 1. Gil Z, Patel SG, Cantu G, Fliss DM, Kowalski LP, Singh B, Snyderman C, Kraus DH, Shah JP; International Collaborative Study Group; Bridger PG, Cheesman AD, Donald P, Gullane P, Janecka I, Kamata SE, Levine PA, Medina LR, Pradhan S, Schramm V, Wei WI. Outcome of craniofacial surgery in children and adolescents with malignant tumors involving the skull base: an international collaborative study. Head Neck. 2009 Mar;31(3):308-17. doi: 10.1002/hed.20958. PMID: 19073003. | 6 |
| 1. Buhari, M. O., Adigun, I. A., Rahman, G. A., Omotayo, J. A., & Ogundipe, K. O. (2009). Soft tissue sarcoma of the head and neck: description of 27 cases and review of the literature. | 7 |
| 1. Mojarro I.A.M.,Lara C.S.,Bojorquez D.R.,Castillejos M.,Moreno S.,Vega R.,Suck M.L.T. Adults cerebral sarcoma.Archivos de Neurociencias 2009 14:4 (243-248) | 6 |
| 1. Jan, A. (2009, November). CLINICO INVESTIGATIVE PROFILE AND TREATMENT OUTCOME OF CHILDREN WITH RMS. In *PEDIATRIC BLOOD & CANCER* (Vol. 53, No. 5, pp. 818-818). DIV JOHN WILEY & SONS INC, 111 RIVER ST, HOBOKEN, NJ 07030 USA: WILEY-LISS. | 4 |
| 1. Merrot O, Gleizal A, Poupart M, Pignat JC. Cartilaginous tumors of the larynx: endoscopic laser management using YAG/KTP. Head Neck. 2009 Feb;31(2):145-52. doi: 10.1002/hed.20932. PMID: 18972422. | 9 |
| 1. Shapiro NL, Bhattacharyya N. Staging and survival for sinus cancer in the pediatric population. Int J Pediatr Otorhinolaryngol. 2009 Nov;73(11):1568-71. doi: 10.1016/j.ijporl.2009.08.006. Epub 2009 Aug 31. PMID: 19720405. | 2 |
| 1. Salas S, Stoeckle E, Collin F, Bui B, Terrier P, Guillou L, Trassard M, Ranchere-Vince D, Gregoire F, Coindre JM. Superficial soft tissue sarcomas (S-STS): a study of 367 patients from the French Sarcoma Group (FSG) database. Eur J Cancer. 2009 Aug;45(12):2091-102. doi: 10.1016/j.ejca.2009.03.006. Epub 2009 Apr 6. PMID: 19351580. | 2 |
| 1. Akhiwu, W. O., Igbe, A. P., Aligbe, J. U., Eze, G. I., & Akang, E. E. (2009). Malignant childhood solid tumours in Benin City, Nigeria. *West African journal of medicine*, *28*(4), 222-226. | 7 |
| 1. Abrigo JM, King AD, Leung SF, Vlantis AC, Wong JK, Tong MC, Tse GM, Ahuja AT. MRI of radiation-induced tumors of the head and neck in post-radiation nasopharyngeal carcinoma. Eur Radiol. 2009 May;19(5):1197-205. doi: 10.1007/s00330-008-1265-6. Epub 2009 Jan 14. PMID: 19142643. | 9 |
| 1. Baccarani U, Adani GL, Serraino D, Lorenzin D, Gambato M, Buda A, Zanus G, Vitale A, Piselli P, De Paoli A, Bresadola V, Risaliti A, Toniutto P, Cillo U, Bresadola F, Burra P. De novo tumors are a major cause of late mortality after orthotopic liver transplantation. Transplant Proc. 2009 May;41(4):1303-5. doi: 10.1016/j.transproceed.2009.03.079. PMID: 19460546. | 1 |
| 1. Asuquo ME, Ebughe G. Cutaneous cancers in Calabar, Southern Nigeria. Dermatol Online J. 2009 Apr 15;15(4):11. PMID: 19450404. | 9 |
| 1. Adeyemi, B. F., Kolude, B. M., Ogun, G. O., & Akang, E. E. U. (2009). Paediatric head and neck malignancies in Ibadan, Nigeria. | 2 |
| 1. Alabi, S., Rahman, G., & Badmos, K. (2009). Pattern of head and neck cancers in a Nigerian tertiary health center: A 10-year review. *Journal of Clinical Oncology*, *27*(15_suppl), e17054-e17054. | 2 |
| 1. Neuhaus SJ, Pinnock N, Giblin V, Fisher C, Thway K, Thomas JM, Hayes AJ. Treatment and outcome of radiation-induced soft-tissue sarcomas at a specialist institution. Eur J Surg Oncol. 2009 Jun;35(6):654-9. doi: 10.1016/j.ejso.2008.11.008. Epub 2008 Dec 27. PMID: 19112005. | 9 |
| 1. Aljumaily, U., Ismael, T., Ayyad, O., Masarweh, M., Ghandour, K., Almousa, A., ... & Sultan, I. (2010, November). EVIDENCE-BASED DECISIONS AND BETTER CARE OF RHABDOMYOSARCOMA IN JORDAN. In *PEDIATRIC BLOOD & CANCER* (Vol. 55, No. 5, pp. 903-903). DIV JOHN WILEY & SONS INC, 111 RIVER ST, HOBOKEN, NJ 07030 USA: WILEY-LISS. | 4 |
| 1. Li SR, Yang ZY, Zheng SY. [Appearance and clinical value of MRI in sinonasal embryonal rhabdomyosarcoma]. Zhonghua Er Bi Yan Hou Tou Jing Wai Ke Za Zhi. 2010 May;45(5):393-6. Chinese. PMID: 20654175. | 9 |
| 1. Osman TA, Satti AA, Boe OE, Yang YH, Ibrahim SO, Suleiman AM. Pattern of malignant tumors registered at a referral oral and maxillofacial hospital in Sudan during 2006 and 2007. J Cancer Res Ther. 2010 Oct-Dec;6(4):473-7. doi: 10.4103/0973-1482.77112. PMID: 21358083. | 2 |
| 1. Missaoui N, Landolsi H, Jaidene L, Anjorin A, Abdelkader AB, Yaacoubi MT, Hmissa S. Pediatric rhabdomyosarcomas in Tunisia. Asian Pac J Cancer Prev. 2010;11(5):1325-7. PMID: 21198286. | 2 |
| 1. Baccarani U, Piselli P, Serraino D, Adani GL, Lorenzin D, Gambato M, Buda A, Zanus G, Vitale A, De Paoli A, Cimaglia C, Bresadola V, Toniutto P, Risaliti A, Cillo U, Bresadola F, Burra P. Comparison of de novo tumours after liver transplantation with incidence rates from Italian cancer registries. Dig Liver Dis. 2010 Jan;42(1):55-60. doi: 10.1016/j.dld.2009.04.017. Epub 2009 Jun 3. PMID: 19497797. | 1 |
| 1. Chang CS, Bergeron L, Liao CC, Liao HT, Chang CN, Chen PK, Chen YR. Craniofacial reconstruction of primary osteogenic sarcoma of the skull. J Plast Reconstr Aesthet Surg. 2010 Aug;63(8):1265-8. doi: 10.1016/j.bjps.2009.07.020. Epub 2009 Sep 1. PMID: 19726258. | 9 |
| 1. Loghdey, M. S., Varma, S., Rajpara, S. M., Al-Rawi, H., Perks, G., & Perkins, W. (2014). Mohs micrographic surgery for dermatofibrosarcoma protuberans (DFSP): a single-centre series of 76 patients treated by frozen-section Mohs micrographic surgery with a review of the literature. *Journal of Plastic, Reconstructive & Aesthetic Surgery*, *67*(10), 1315-1321. | 9 |
| 1. Hessissen L, Kanouni L, Kili A, Nachef MN, El Khorassani M, Benjaafar N, Khattab M, El Gueddari Bel K. Pediatric rhabdomyosarcoma in Morocco. Pediatr Blood Cancer. 2010 Jan;54(1):25-8. doi: 10.1002/pbc.22173. PMID: 19746454. | 2 |
| 1. Welte B, Suhr P, Bottke D, Bartkowiak D, Dörr W, Trott KR, Wiegel T. Second malignancies in high‑dose areas of previous tumor radiotherapy. Strahlenther Onkol. 2010 Mar;186(3):174-9. doi: 10.1007/s00066-010-2050-4. Epub 2010 Feb 22. PMID: 20339826. | 1 |
| 1. Li L, Shi YH, Guo ZJ, Qiu T, Guo L, Yang HY, Zhang X, Zhao XM, Su Q. Clinicopathological features and prognosis assessment of extranodal follicular dendritic cell sarcoma. World J Gastroenterol. 2010 May 28;16(20):2504-19. doi: 10.3748/wjg.v16.i20.2504. PMID: 20503450; PMCID: PMC2877180. | 9 |
| 1. Meguerditchian AN, Wang J, Lema B, Kraybill WG, Zeitouni NC, Kane JM 3rd. Wide excision or Mohs micrographic surgery for the treatment of primary dermatofibrosarcoma protuberans. Am J Clin Oncol. 2010 Jun;33(3):300-3. doi: 10.1097/COC.0b013e3181aaca87. PMID: 19858696. | 2 |
| 1. Whaley JT, Indelicato DJ, Morris CG, Hinerman RW, Amdur RJ, Mendenhall WM, Keole SR, Marcus RB Jr. Ewing tumors of the head and neck. Am J Clin Oncol. 2010 Aug;33(4):321-6. doi: 10.1097/COC.0b013e3181aaca71. PMID: 19841575. | 9 |
| 1. Healy JN, Borg MF. Paediatric nasopharyngeal rhabdomyosarcoma: A case series and literature review. J Med Imaging Radiat Oncol. 2010 Aug;54(4):388-94. doi: 10.1111/j.1754-9485.2010.02187.x. PMID: 20718921. | 2 |
| 1. Wagemans J, Beuselinck B, Nuyts S, Sciot R, Delaere P, Vander Poorten V, Dumez H, Hermans R, Schöffski P, Van den Bogaert W, Jorissen M, Clement PM. A case series of embryonal rhabdomyosarcoma of the head and neck in adults. Acta Clin Belg. 2010 Nov-Dec;65(6):404-10. doi: 10.1179/acb.2010.65.6.006. PMID: 21268954. | 9 |
| 1. Holsinger FC, Hafemeister AC, Hicks MJ, Sulek M, Huh WW, Friedman EM. Differential diagnosis of pediatric tumors of the nasal cavity and paranasal sinuses: a 45-year multi-institutional review. Ear Nose Throat J. 2010 Nov;89(11):534-40. PMID: 21086277. | 2 |
| 1. Almadori, G., Bussu, F., Rigante, M., Galli, J., Cadoni, G., Dinapoli, N., ... & Gaetano, P. (2011). PRIMARY MALIGNANCIES OF NOSE AND PARANASAL SINUSES. A SURGICAL SERIES OF 91 CASES. *Radiotherapy and Oncology*, (98), S32. | 4 |
| 1. Özfindik, M., Elli, M., & Dağdemir, A. (2011). Retrospective analysis of childhood cancer of the head and neck Çocukluk çaǧi malign baş-boyun tümörlerinin Retrospektif incelenmesi. *Journal of Experimental and Clinical Medicine (Turkey)*, *28*(2). | 7 |
| 1. Gardner, J. M., Dandekar, M., Thomas, D. G., Goldblum, J. R., Weiss, S. W., Billings, S., ... & Patel, R. M. (2011, February). Cutaneous Pleomorphic Liposarcoma (PL): A Clinicopathologic Study of 33 Cases with Evaluation of MDM2 Gene Amplification in 19. In *LABORATORY INVESTIGATION* (Vol. 91, pp. 116A-116A). 75 VARICK ST, 9TH FLR, NEW YORK, NY 10013-1917 USA: NATURE PUBLISHING GROUP. | 9 |
| 1. Deyrup AT, Miettinen M, North PE, Khoury JD, Tighiouart M, Spunt SL, Parham DM, Shehata BM, Weiss SW. Pediatric cutaneous angiosarcomas: a clinicopathologic study of 10 cases. Am J Surg Pathol. 2011 Jan;35(1):70-5. doi: 10.1097/PAS.0b013e3181ffd9d5. PMID: 21164289. | 9 |
| 1. Naser H.,Tang P.H.,Tan T.A.,Chan M.Y.,Al-Hashimi H.Imaging findings of childhood rhabdomyosarcoma. Pediatric Radiology 2011 41 (S415-S416) SUPPL. 1 | 9 |
| 1. Ahn HK, Uhm JE, Lee J, Lim DH, Seo SW, Sung KS, Lee SJ, Lee DJ, Baek KK, Kim WS, Park JO. Analysis of prognostic factors of pediatric-type sarcomas in adult patients. Oncology. 2011;80(1-2):21-8. doi: 10.1159/000327222. Epub 2011 May 23. PMID: 21606660. | 2 |
| 1. Lanoy E, Spano JP, Bonnet F, Guiguet M, Boué F, Cadranel J, Carcelain G, Couderc LJ, Frange P, Girard PM, Oksenhendler E, Poizot-Martin I, Semaille C, Agut H, Katlama C, Costagliola D; ONCOVIH study group. The spectrum of malignancies in HIV-infected patients in 2006 in France: the ONCOVIH study. Int J Cancer. 2011 Jul 15;129(2):467-75. doi: 10.1002/ijc.25903. Epub 2011 Apr 27. PMID: 21207370. | 2 |
| 1. Gimenez, C., & Pereira, A. (2011, November). RHABDOMYOSARCOMA IN CHILDREN AND ADOLESCENTS. In *PEDIATRIC BLOOD & CANCER* (Vol. 57, No. 5, pp. 820-820). COMMERCE PLACE, 350 MAIN STREET, MALDEN, MA 02148-529 USA: WILEY PERIODICALS, INC. | 9 |
| 1. Kerboua E.Head and neck sarcoma, what's about treatment?Radiotherapy and Oncology 2011 98 (S38) SUPPL. 1 | 4 |
| 1. Mandong BM, Ngbea JA. Childhood rhabdomyosarcoma: a review of 35 cases and literature. Niger J Med. 2011 Oct-Dec;20(4):466-9. PMID: 22288325. | 2 |
| 1. Al Yamani, A. O., Al Sebaei, M. O., Bassyoni, L. J., Badghaish, A. J., & Shawly, H. H. (2011). Variation of pediatric and adolescents head and neck pathology in the city of Jeddah: A retrospective analysis over 10 years. *The Saudi dental journal*, *23*(4), 197-200. | 1 |
| 1. Chacón, M., Vilchez, V., Angel, M., Arganaraz, F., Kaplan, J., & Chacón, R. (2011). 9432 POSTER Age, Location and Histology in Soft Tissue Sarcomas–Single Institutional Review. *European Journal of Cancer*, (47), S672. | 4 |
| 1. Ahrens, M., Hoiczyk, M., Grabellus, F., Taeger, G., Poettgen, C., Schuler, M., & Bauer, S. (2011, September). Angiosarcomas of the adulthood-a large single center analysis. In *ONKOLOGIE* (Vol. 34, pp. 254-254). ALLSCHWILERSTRASSE 10, CH-4009 BASEL, SWITZERLAND: KARGER. | 4 |
| 1. Bompas, E., Campion, L., Italiano, A., Cesne, A. L., Giaj Levra, M., Chevreau, C., ... & Blay, J. (2011). Outcome of 157 adult rhabdomyosarcoma (RMS) patients: A retrospective study from the French Group Sarcoma (GSF-GETO). *Journal of Clinical Oncology*, *29*(15_suppl), 10070-10070. | 4 |
| 1. Tan WP, Barlow RJ, Robson A, Kurwa HA, McKenna J, Mallipeddi R. Dermatofibrosarcoma protuberans: 35 patients treated with Mohs micrographic surgery using paraffin sections. Br J Dermatol. 2011 Feb;164(2):363-6. doi: 10.1111/j.1365-2133.2010.10095.x. PMID: 20973768. | 9 |
| 1. Zevallos JP, Jain KS, Roberts D, El-Naggar A, Hanna EY, Kupferman ME. Sinonasal malignancies in children: a 10-year, single-institutional review. Laryngoscope. 2011 Sep;121(9):2001-3. doi: 10.1002/lary.21793. Epub 2011 Jul 7. PMID: 21739433. | 2 |
| 1. Gradoni P, Giordano D, Oretti G, Fantoni M, Barone A, La Cava S, Ferri A, Sesenna E, Ferri T, Izzi GC. Clinical outcomes of rhabdomyosarcoma and Ewing's sarcoma of the head and neck in children. Auris Nasus Larynx. 2011 Aug;38(4):480-6. doi: 10.1016/j.anl.2010.12.004. Epub 2011 Jan 11. PMID: 21227608. | 9 |
| 1. Zhao M, Feng C, Wang JW, Liu Y, Tang SQ. [Childhood rhabdomyosarcoma: a retrospective review of 23 cases]. Zhongguo Dang Dai Er Ke Za Zhi. 2011 Aug;13(8):657-60. Chinese. PMID: 21849118. | 7 |
| 1. Wollina U, Hansel G, Schönlebe J, Averbeck M, Paasch U, Uhl J, Hindemann W, Simon JC. Cutaneous angiosarcoma is a rare aggressive malignant vascular tumour of the skin. J Eur Acad Dermatol Venereol. 2011 Aug;25(8):964-8. doi: 10.1111/j.1468-3083.2010.03905.x. Epub 2010 Nov 25. PMID: 21108661. | 9 |
| 1. Goda JS, Ferguson PC, O'Sullivan B, Catton CN, Griffin AM, Wunder JS, Bell RS, Kandel RA, Chung PW. High-risk extracranial chondrosarcoma: long-term results of surgery and radiation therapy. Cancer. 2011 Jun 1;117(11):2513-9. doi: 10.1002/cncr.25806. Epub 2011 Jan 18. PMID: 21246520. | 9 |
| 1. Adisa AO, Adeyemi BF, Oluwasola AO, Kolude B, Akang EE, Lawoyin JO. Clinico-pathological profile of head and neck malignancies at University College Hospital, Ibadan, Nigeria. Head Face Med. 2011 May 13;7:9. doi: 10.1186/1746-160X-7-9. PMID: 21569492; PMCID: PMC3115889. | 2 |
| 1. Albores-Saavedra J, Schwartz AM, Henson DE, Kostun L, Hart A, Angeles-Albores D, Chablé-Montero F. Cutaneous angiosarcoma. Analysis of 434 cases from the Surveillance, Epidemiology, and End Results Program, 1973-2007. Ann Diagn Pathol. 2011 Apr;15(2):93-7. doi: 10.1016/j.anndiagpath.2010.07.012. Epub 2010 Dec 28. PMID: 21190880. | 2 |
| 1. Liu JC, Givi B, Wolden S, Kleinerman RA, Dunkel IJ, Lee N, Shah JP, Abramson DH, Kraus DH. Secondary skull base malignancies in survivors of retinoblastoma: the memorial sloan kettering cancer center experience. Skull Base. 2011 Mar;21(2):103-8. doi: 10.1055/s-0031-1275256. PMID: 22451810; PMCID: PMC3312595. | 6 |
| 1. Das K, Jain S, Chichra A, Gupta H, Kapoor G. Non-hematological tumors of head and neck region in the pediatric age group in a tertiary care cancer center. Pediatr Surg Int. 2011 Sep;27(9):919-23. doi: 10.1007/s00383-011-2916-2. Epub 2011 May 5. PMID: 21544644. | 2 |
| 1. Cotrufo, S., Balasundram, S., Shake, A., Liew, C., Govender, R., Rogers, P., & Kalavrezos, N. (2011). Assessment of oral intake in advanced oral and oro-pharyngeal cancer patients. A 5 years retrospective study. *British Journal of Oral and Maxillofacial Surgery*, *49*, S15. | 4 |
| 1. Mine, S., Saeki, N., Horiguchi, K., Hanazawa, T., & Okamoto, Y. (2011). Craniofacial resection for sinonasal malignant tumors: statistical analysis of surgical outcome over 17 years at a single institution. *Skull Base*, *21*(04), 243-248. | 1 |
| 1. Chu Y, Liu HG, Yu ZK. Patterns and incidence of sinonasal malignancy with orbital invasion. Chin Med J (Engl). 2012 May;125(9):1638-42. PMID: 22800835. | 2 |
| 1. Rekhi B, Ingle A, Agarwal M, Puri A, Laskar S, Jambhekar NA. Alveolar soft part sarcoma 'revisited': clinicopathological review of 47 cases from a tertiary cancer referral centre, including immunohistochemical expression of TFE3 in 22 cases and 21 other tumours. Pathology. 2012 Jan;44(1):11-7. doi: 10.1097/PAT.0b013e32834d7ba4. PMID: 22173238. | 9 |
| 1. Radhakrishnan, S., Vora, T., Chinnaswamy, G., Dwivedi, P., Patil, V., Avinash, P., ... & Kurkure, P. (2012, December). CLINICAL PROFILE AND OUTCOMES OF RHABDOMYOSARCOMA PATIENTS TREATED AT TATA MEMORIAL HOSPITAL: A RETROSPECTIVE ANALYSIS. In *PEDIATRIC BLOOD & CANCER* (Vol. 59, No. 6, pp. 1067-1067). ONE MONTGOMERY ST, SUITE 1200, SAN FRANCISCO, CA 94104 USA: WILEY PERIODICALS, INC. | 4 |
| 1. Kuo, W., Lin, C., & Yen, T. (2012, October). 18F-FDG PET/CT in Angiosarcoma. In *EUROPEAN JOURNAL OF NUCLEAR MEDICINE AND MOLECULAR IMAGING* (Vol. 39, pp. S476-S476). 233 SPRING ST, NEW YORK, NY 10013 USA: SPRINGER. | 4 |
| 1. Nabiha M.,Yossra B.Y.,Wiem M.,Ahlem B.,Nizar L.,Atef B.A.,Samira M.,Lilia J.,Mohamed Tahar Y., Sihem H. Rhabdomyosarcomas in Central Tunisia: Epidemiological and anatomoclinical study of 47 cases. Virchows Archiv 2012 461:1 (S229) SUPPL. 1 | 4 |
| 1. Abdulai, A. E., Nuamah, I. K., & Gyasi, R. (2012). Head and neck tumours in Ghanaian children. A 20 year review. *International journal of oral and maxillofacial surgery*, *41*(11), 1378-1382. | 2 |
| 1. Thiele O.,Seeberger R., Hoffmann J., Freier K. A monocenter evaluation of the frequency of malignant tumours in the head and neck area. Journal of Cancer Research and Clinical Oncology 2012 138 (111-112) SUPPL. 1 | 4 |
| 1. Gardner JM, Dandekar M, Thomas D, Goldblum JR, Weiss SW, Billings SD, Lucas DR, McHugh JB, Patel RM. Cutaneous and subcutaneous pleomorphic liposarcoma: a clinicopathologic study of 29 cases with evaluation of MDM2 gene amplification in 26. Am J Surg Pathol. 2012 Jul;36(7):1047-51. doi: 10.1097/PAS.0b013e3182517b96. PMID: 22472959. | 9 |
| 1. Dziegielewski, P. T., O'Hara, C., Erickson, B., Seikaly, H., & Harris, J. R. (2012). Idiopathic Vocal Cord Paralysis in the Radiated Neck: A Harbinger for Malignant Peripheral Nerve Sheath Tumour. *Journal of Otolaryngology--Head & Neck Surgery*, *41*(4). | 9 |
| 1. Kuzel P, Metelitsa AI, Dover DC, Salopek TG. Epidemiology of dermatofibrosarcoma protuberans in Alberta, Canada, from 1988 to 2007. Dermatol Surg. 2012 Sep;38(9):1461-8. doi: 10.1111/j.1524-4725.2012.02482.x. Epub 2012 Jun 12. PMID: 22691126. | 2 |
| 1. Gibson TN, Hanchard B, Waugh N, McNaughton D. A fifty-year review of soft tissue sarcomas in Jamaica: 1958-2007. West Indian Med J. 2012 Oct;61(7):692-7. PMID: 23620966. | 2 |
| 1. Wakely PE Jr, Ali SZ, Bishop JA. The cytopathology of malignant peripheral nerve sheath tumor: a report of 55 fine-needle aspiration cases. Cancer Cytopathol. 2012 Oct 25;120(5):334-41. doi: 10.1002/cncy.21195. Epub 2012 Mar 20. PMID: 22434579. | 9 |
| 1. Irarrazaval I, Redondo P. Three-dimensional histology for dermatofibrosarcoma protuberans: case series and surgical technique. J Am Acad Dermatol. 2012 Nov;67(5):991-6. doi: 10.1016/j.jaad.2012.03.034. Epub 2012 May 30. PMID: 22657156. | 9 |
| 1. Buda I, Hod R, Feinmesser R, Shvero J. Chondrosarcoma of the larynx. Isr Med Assoc J. 2012 Nov;14(11):681-4. PMID: 23240373. | 9 |
| 1. Asuquo ME, Ebughe G. Major dermatological malignancies encountered in the University of Calabar Teaching Hospital, Calabar, southern Nigeria. Int J Dermatol. 2012 Nov;51 Suppl 1:32-6, 36-40. English, French. doi: 10.1111/j.1365-4632.2012.05562.x. PMID: 23210953. | 9 |
| 1. Badr MA, Al-Tonbary YA, Mansour AK, Hassan TH, Beshir MR, Darwish A, El-Ashry RA. Epidemiological characteristics and survival studies of rhabdomyosarcoma in East egypt: a five-year multicenter study. ISRN Oncol. 2012;2012:674523. doi: 10.5402/2012/674523. Epub 2012 May 17. PMID: 22675642; PMCID: PMC3362855. | 2 |
| 1. Shama M.,Soliman H.,Hafez M.,Khorshed E.,Shoman T., El Baradie T.Retrospective analyses of 67 patients with head and neck soft tissue sarcomas: Subtypes and prognostic indicators.European Archives of Oto-Rhino-Laryngology 2012 269:4 (1356) | 4 |
| 1. Shinagare AB, Krajewski KM, Hornick JL, Zukotynski K, Kurra V, Jagannathan JP, Ramaiya NH. MRI for evaluation of myeloid sarcoma in adults: a single-institution 10-year experience. AJR Am J Roentgenol. 2012 Dec;199(6):1193-8. doi: 10.2214/AJR.12.9057. PMID: 23169708. | 9 |
| 1. Suh JD, Ramakrishnan VR, Chi JJ, Palmer JN, Chiu AG. Outcomes and complications of endoscopic approaches for malignancies of the paranasal sinuses and anterior skull base. Ann Otol Rhinol Laryngol. 2013 Jan;122(1):54-9. doi: 10.1177/000348941312200110. PMID: 23472317. | 1 |
| 1. Qasim, S. (2013). Outcome of myxofibrosarcoma of soft tissue. *European Journal of Surgical Oncology*, *39*(11), S67. | 4 |
| 1. Sen S., Tanksh R.A. Adult head and neck sarcomas-a 5 year retrospective study with review of literature. Indian Journal of Surgical Oncology 2013 4:2 (214-215) | 4 |
| 1. Peng, K. A., & Wang, M. B. (2013). Prognostic Factors in Head and Neck Sarcomas: Analysis of the SEER Database. *Otolaryngology—Head and Neck Surgery*, *149*(2_suppl), P187-P187. | 4 |
| 1. Durnali A, Alkis N, Cangur S, Yukruk FA, Inal A, Tokluoglu S, Seker MM, Bal O, Akman T, Inanc M, Isikdogan A, Demirci A, Helvaci K, Oksuzoglu B. Prognostic factors for teenage and adult patients with high-grade osteosarcoma: an analysis of 240 patients. Med Oncol. 2013;30(3):624. doi: 10.1007/s12032-013-0624-6. Epub 2013 Jun 9. PMID: 23749307. | 2 |
| 1. Hoekstra, H. J., Bongers, B. G. H., Van der Graaf, W. T. A., Haas, R. L. M., Van Rijswijk, C. S. P., Siesling, S., ... & Ho, V. K. Y. (2013). Soft tissue sarcoma care in the Netherlands: epidemiology, patterns of care and outcome data. *European Journal of Cancer*, *49*, S877-S877. | 4 |
| 1. Pearce, C., Al-Asaadi, Z., Williams, H., Peake, D., Rehman, K. U., Martin, T., & Parmar, S. (2013). Head and neck sarcomas: a 12 year retrospective review of 40 cases. *British Journal of Oral and Maxillofacial Surgery*, *51*(6), e113-e114. | 4 |
| 1. Stanelle EJ, Christison-Lagay ER, Healey JH, Singer S, Meyers PA, La Quaglia MP. Pediatric and adolescent synovial sarcoma: multivariate analysis of prognostic factors and survival outcomes. Ann Surg Oncol. 2013 Jan;20(1):73-9. doi: 10.1245/s10434-012-2587-9. Epub 2012 Aug 10. PMID: 22878620. | 2 |
| 1. Yusuf, I., Mohammed, A. Z., & Iliyasu, Y. (2013). Histopathological study of soft tissue sarcomas seen in a teaching hospital in Kano, Nigeria. *Nigerian Journal of Basic and Clinical Sciences*, *10*(2), 70-75. | 2 |
| 1. Jackson RS, Leon ME, McCaffrey TV. Chondrosarcoma of the subglottic larynx: submucosal microdissection with the operating microscope. Laryngoscope. 2013 May;123(5):1216-9. doi: 10.1002/lary.23957. Epub 2013 Feb 12. PMID: 23404226. | 9 |
| 1. Schütz A, Smeets R, Driemel O, Hakim SG, Kosmehl H, Hanken H, Kolk A. Primary and secondary leiomyosarcoma of the oral and perioral region--clinicopathological and immunohistochemical analysis of a rare entity with a review of the literature. J Oral Maxillofac Surg. 2013 Jun;71(6):1132-42. doi: 10.1016/j.joms.2012.12.011. Epub 2013 Feb 20. PMID: 23434173. | 9 |
| 1. Akinmoladun V, Pindiga U, Akintububo O, Kokong D, Akinyamoju C. Head and neck malignant tumours in gombe, northeast Nigeria. J West Afr Coll Surg. 2013 Jul-Sep;3(3):1-15. PMID: 25717459; PMCID: PMC4337212. | 2 |
| 1. Roby BB, Drehner D, Sidman JD. Granulocytic sarcoma of pediatric head and neck: an institutional experience. Int J Pediatr Otorhinolaryngol. 2013 Aug;77(8):1364-6. doi: 10.1016/j.ijporl.2013.06.008. Epub 2013 Jun 28. PMID: 23810551. | 9 |
| 1. Ettorre GM, Piselli P, Galatioto L, Rendina M, Nudo F, Sforza D, Miglioresi L, Fantola G, Cimaglia C, Vennarecci G, Vizzini GB, Di Leo A, Rossi M, Tisone G, Zamboni F, Santoro R, Agresta A, Puro V, Serraino D. De novo malignancies following liver transplantation: results from a multicentric study in central and southern Italy, 1990-2008. Transplant Proc. 2013 Sep;45(7):2729-32. doi: 10.1016/j.transproceed.2013.07.050. PMID: 24034034. | 1 |
| 1. Perez MC, Padhya TA, Messina JL, Jackson RS, Gonzalez RJ, Bui MM, Letson GD, Cruse CW, Lavey RS, Cheong D, Forster MR, Fulp WJ, Sondak VK, Zager JS. Cutaneous angiosarcoma: a single-institution experience. Ann Surg Oncol. 2013 Oct;20(11):3391-7. doi: 10.1245/s10434-013-3083-6. Epub 2013 Jul 9. PMID: 23835652; PMCID: PMC4509495. | 2 |
| 1. Sbaihat A, Bacciu A, Pasanisi E, Sanna M. Skull base chondrosarcomas: surgical treatment and results. Ann Otol Rhinol Laryngol. 2013 Dec;122(12):763-70. doi: 10.1177/000348941312201206. PMID: 24592579. | 6 |
| 1. Ramanathan, P., Deo, S. V. S., & Shukla, N. K. (2013, September). Prognostic factors of head and neck sarcomas in a tertiary care centre. In *EUROPEAN JOURNAL OF CANCER* (Vol. 49, pp. S753-S753). THE BOULEVARD, LANGFORD LANE, KIDLINGTON, OXFORD OX5 1GB, OXON, ENGLAND: ELSEVIER SCI LTD. | 4 |
| 1. Chang, A., Chai, X., Futran, N., & Jones, R. (2013, January). RETROSPECTIVE ANALYSIS OF ADULT HEAD AND NECK SARCOMA: A SINGLE-CENTER EXPERIENCE FROM 2000-2012. In *JOURNAL OF INVESTIGATIVE MEDICINE* (Vol. 61, No. 1, pp. 228-228). 530 WALNUT ST, PHILADELPHIA, PA 19106-3621 USA: LIPPINCOTT WILLIAMS & WILKINS. | 4 |
| 1. Bagri, P. K., Singh, D., Singhal, M. K., Singh, G., Mathur, G., Jakhar, S. L., ... & Bardia, M. R. (2014). Double primary malignancies: a clinical & pathological analysis report from a regional cancer institute in India. *Iranian journal of cancer prevention*, *7*(2), 66. | 1 |
| 1. Iqbal, N., Thakar, A., Agarwala, S., Sharma, D. N., Sharma, M. C., & Bakhshi, S. (2014, December). LOCALIZED RHABDOMYOSARCOMA OF HEAD AND NECK: A RETROSPECTIVE ANALYSIS OF 80 PATIENTS TREATED AT A TERTIARY CARE CENTRE IN INDIA. In *PEDIATRIC BLOOD & CANCER* (Vol. 61, pp. S383-S383). 111 RIVER ST, HOBOKEN 07030-5774, NJ USA: WILEY-BLACKWELL. | 4 |
| 1. Ramirez-Urenda, X. A., Paniagua-Padilla, J., & Sanchez-Zubieta, F. A. (2014, December). OCCIDENT OF MEXICO RHABDOMYOSARCOMA (RMS) EXPERIENCE: SLIGHT IMPROVEMENT WITH A LOT OF WORK TO DO. In *PEDIATRIC BLOOD & CANCER* (Vol. 61, pp. S384-S384). 111 RIVER ST, HOBOKEN 07030-5774, NJ USA: WILEY-BLACKWELL. | 4 |
| 1. Di Monta G, Caracò C, Benedetto L, La Padula S, Marone U, Tornesello ML, Buonaguro FM, Simeone E, Ascierto PA, Mozzillo N. Electrochemotherapy as "new standard of care" treatment for cutaneous Kaposi's sarcoma. Eur J Surg Oncol. 2014 Jan;40(1):61-6. doi: 10.1016/j.ejso.2013.09.002. Epub 2013 Sep 12. PMID: 24075826. | 6 |
| 1. Martínez Martínez, M., Mosqueda‐Taylor, A., Carlos, R., Delgado‐Azañero, W., & De Almeida, O. P. (2014). Malignant odontogenic tumors: a multicentric Latin American study of 25 cases. *Oral diseases*, *20*(4), 380-385. | 9 |
| 1. Williams, L., Wong, T., Newman, L., Liew, C., Michelagnoli, M., & Kalavrezos, N. (2014). Paediatric head and neck sarcoma: The London Sarcoma Service series. *British Journal of Oral and Maxillofacial Surgery*, *52*(8), e63-e64. | 4 |
| 1. Huang, P., Jacobson, A., Giraud, C., Choy, E., Harmon, D., DeLaney, T. F., & Chen, Y. (2014). Outcomes of Head and Neck Osteosarcoma: Ten-Year Experience at a Single Institution. *International Journal of Radiation Oncology, Biology, Physics*, *90*(1), S757-S758. | 4 |
| 1. Zhang X, Ma K, Wang J, Wu W, Ma L, Huang D. A prospective evaluation of the combined helical tomotherapy and chemotherapy in pediatric patients with unresectable rhabdomyosarcoma of the temporal bone. Cell Biochem Biophys. 2014 Sep;70(1):103-8. doi: 10.1007/s12013-014-9864-0. PMID: 24619819. | 9 |
| 1. Aydil U, Kızıl Y, Bakkal FK, Köybaşıoğlu A, Uslu S. Neoplasms of the hard palate. J Oral Maxillofac Surg. 2014 Mar;72(3):619-26. doi: 10.1016/j.joms.2013.08.019. Epub 2013 Oct 16. PMID: 24139293. | 9 |
| 1. Nonaka D, Bishop PW. Sarcoma-like tumor of head and neck skin. Am J Surg Pathol. 2014 Jul;38(7):956-65. doi: 10.1097/PAS.0000000000000210. PMID: 24705313. | 5 |
| 1. Huang G, Ye F, Fu M, Chen Y. [Clinical analysis of low grade myofibroblastic sarcoma in head and neck]. Lin Chuang Er Bi Yan Hou Tou Jing Wai Ke Za Zhi. 2014 Aug;28(15):1148-50. Chinese. PMID: 25322605. | 9 |
| 1. Singla S, Papavasiliou P, Powers B, Gaughan J, von Mehren M, Watson JC, Farma JM. Challenges in the treatment of angiosarcoma: a single institution experience. Am J Surg. 2014 Aug;208(2):254-9. doi: 10.1016/j.amjsurg.2014.01.007. Epub 2014 Apr 15. PMID: 24811931. | 2 |
| 1. Loghdey MS, Varma S, Rajpara SM, Al-Rawi H, Perks G, Perkins W. Mohs micrographic surgery for dermatofibrosarcoma protuberans (DFSP): a single-centre series of 76 patients treated by frozen-section Mohs micrographic surgery with a review of the literature. J Plast Reconstr Aesthet Surg. 2014 Oct;67(10):1315-21. doi: 10.1016/j.bjps.2014.05.021. Epub 2014 May 23. PMID: 25012249. | 2 |
| 1. Buehler D, Rice SR, Moody JS, Rush P, Hafez GR, Attia S, Longley BJ, Kozak KR. Angiosarcoma outcomes and prognostic factors: a 25-year single institution experience. Am J Clin Oncol. 2014 Oct;37(5):473-9. doi: 10.1097/COC.0b013e31827e4e7b. PMID: 23428947; PMCID: PMC3664266. | 2 |
| 1. Ahmad Z, Din NU, Ahmad A, Imran S, Pervez S, Ahmed R, Kayani N. Rhabdomyosarcoma--an epidemiological and histopathologic study of 277 cases from a major tertiary care center in Karachi, Pakistan. Asian Pac J Cancer Prev. 2015;16(2):757-60. doi: 10.7314/apjcp.2015.16.2.757. PMID: 25684521. | 2 |
| 1. Lee, J., Ali, S., VandenBussche, C., Kazmi, S., & Bishop, J. (2015). Fine Needle Aspiration Cytopathology of Head and Neck Soft Tissue Neoplasms. *Journal of the American Society of Cytopathology*, *4*(6), S85. | 4 |
| 1. Darré, T., Bissa, H., Akloa, K. E., Pegbessou, E., Amana, B., N’guessan, A. A., ... & Napo-Koura, G. (2015). Epidemiological and pathological aspects of head and neck cancers in Togo. *Journal of Analytical Oncology*, *4*(1), 30-34. | 9 |
| 1. Ma X, Huang D, Zhao W, Sun L, Xiong H, Zhang Y, Jin M, Zhang D, Huang C, Wang H, Zhang W, Sun N, He L, Tang J. Clinical characteristics and prognosis of childhood rhabdomyosarcoma: a ten-year retrospective multicenter study. Int J Clin Exp Med. 2015 Oct 15;8(10):17196-205. PMID: 26770312; PMCID: PMC4694212. | 2 |
| 1. Armando A, Bozzetti MC, de Medeiros Zelmanowicz A, Miguel F. The epidemiology of cancer in Angola-results from the cancer registry of the national oncology centre of Luanda, Angola. Ecancermedicalscience. 2015 Feb 17;9:510. doi: 10.3332/ecancer.2015.510. PMID: 25729423; PMCID: PMC4335969. | 1 |
| 1. Hayward C.M.,Mehta V.Analysis of head and neck dermatofibrosarcoma protuberans. Otolaryngology - Head and Neck Surgery (United States) 2015 153:1 (162) SUPPL. 1 | 4 |
| 1. DeVito N, Henderson E, Han G, Reed D, Bui MM, Lavey R, Robinson L, Zager JS, Gonzalez RJ, Sondak VK, Letson GD, Conley A. Clinical Characteristics and Outcomes for Solitary Fibrous Tumor (SFT): A Single Center Experience. PLoS One. 2015 Oct 15;10(10):e0140362. doi: 10.1371/journal.pone.0140362. PMID: 26469269; PMCID: PMC4607370. | 9 |
| 1. Sultania, M., Shukla, N. K., Deo, S. V. S., Bakhshi, S., Sharma, D. N., & Thulkar, S. (2015). 3424 Clinical profile, treatment patterns and outcome of head and neck sarcomas in a tertiary care cancer centre. *European Journal of Cancer*, *3*(51), S696. | 4 |
| 1. El-Nadi, E., Elzomor, H., Labib, R. M., Alfaar, A. S., Zaghloul, M. S., Taha, H., ... & Elwakeel, M. (2015). Childhood orbital rhabdomyosarcoma: report from children’s cancer Hospital-57357-Egypt. *JST*, *5*, 94-104. | 4 |
| 1. Brandmaier, A., Wu, X., Christos, P., Mann, J. M., Wernicke, A. G., Nori, D., & Parashar, B. (2015). A Population-Based Comparative Outcome of Adjuvant Radiation Therapy in Patients With Soft Tissue Sarcoma of the Head and Neck. *International Journal of Radiation Oncology, Biology, Physics*, *93*(3), E635-E636. | 4 |
| 1. Hamid, S. A. (2015, November). PEDIATRIC RHABDOMYOSARCOMA; CLINICOPATHOLOGICAL FEATURES AND OUTCOME; A SINGLE INSTITUTION EXPERIENCE IN PAKISTAN. In *PEDIATRIC BLOOD & CANCER* (Vol. 62, pp. S232-S232). 111 RIVER ST, HOBOKEN 07030-5774, NJ USA: WILEY-BLACKWELL. | 4 |
| 1. Amiruddin, S., Yunus, M. R. M., & Baki, M. M. (2017). A review of adult head and neck soft tissue sarcoma in a tertiary centre: malaysia experience. *Bangladesh Journal of Medical Science*, *16*(1), 69. | 4 |
| 1. Wei, H., Zhiyong, W., Guowen, S., Shengwei, H., & Gingang, H. (2015). P0165 The management of head and neck sarcomas: 10-Year experience from a clinical institute in east China. *European Journal of Cancer*, *51*, e31. | 4 |
| 1. Ferraz M.E.L.,Cerqueira F.N.,De Carvalho A.Y.,Chulam T.C.,Ikeda M.K. Sarcomas of the oral and maxillofacial region: A review of 29 cases. Head and Neck 2015 37 (E111-E112) SUPPL. 1 | 4 |
| 1. Goldberg C, Hoang D, McRae M, Chung C, Leffell DJ, Narayan D. A strategy for the successful management of dermatofibrosarcoma protuberans. Ann Plast Surg. 2015 Jan;74(1):80-4. doi: 10.1097/SAP.0b013e3182898692. PMID: 23788146. | 9 |
| 1. Hu K, Wan J, Ni S, Li X, Liu S, Meng X, Qian H. [Clinical features and comprehensive treatment of skull base osteosarcoma]. Zhonghua Zhong Liu Za Zhi. 2015 May;37(5):383-6. Chinese. PMID: 26463032. | 6 |
| 1. Khosla D, Sapkota S, Kapoor R, Kumar R, Sharma SC. Adult rhabdomyosarcoma: Clinical presentation, treatment, and outcome. J Cancer Res Ther. 2015 Oct-Dec;11(4):830-4. doi: 10.4103/0973-1482.144637. PMID: 26881526. | 2 |
| 1. Fatima SS, Din NU, Ahmad Z. Primary Synovial Sarcoma of the Pharynx: A Series of Five Cases and Literature Review. Head Neck Pathol. 2015 Dec;9(4):458-62. doi: 10.1007/s12105-015-0634-0. Epub 2015 May 29. PMID: 26022274; PMCID: PMC4651928. | 9 |
| 1. Sookprasert A, Ungareewittaya P, Manotepitipongse A, Wirasorn K, Chindaprasirt J. Treatment Outcome and Predictors of Survival in Thai Adult Rhabdomyosarcoma Cases. Asian Pac J Cancer Prev. 2016;17(3):1449-52. doi: 10.7314/apjcp.2016.17.3.1449. PMID: 27039788. | 2 |
| 1. Konuthula N.,Parasher A.,Del Signore A.,Iloreta A.M.,Stepan K.Treatment modalities in sinonasal rhabdomyosarcoma. Otolaryngology - Head and Neck Surgery (United States) 2016 155 (P86-P87) Supplement 1 | 4 |
| 1. Nana, M., Cores, M., & García Lombardi, M. (2016, November). EPIDEMIOLOGY OF HEAD AND NECK PEDIATRIC TUMORS. 30 YEARS EXPERIENCE. In *PEDIATRIC BLOOD & CANCER* (Vol. 63, pp. S154-S154). 111 RIVER ST, HOBOKEN 07030-5774, NJ USA: WILEY-BLACKWELL. | 4 |
| 1. Montejano, R., Ramon Arribas, J., Ignacio Bernardino, J., Martin-Carbonero, L., Luisa Montes, M., Moreno, V., ... & Valencia, E. (2016, October). A descriptive study of cancer incidence in a cohort of HIV-infected patients followed since 1986. In *JOURNAL OF THE INTERNATIONAL AIDS SOCIETY* (Vol. 19). THE ATRIUM, SOUTHERN GATE, CHICHESTER PO19 8SQ, W SUSSEX, ENGLAND: JOHN WILEY & SONS LTD. | 4 |
| 1. Cengiz, M., Dauletkazin, A., Yildiz, F., Yazici, G., Akyol, F., Zorlu, F., ... & Ozyigit, G. (2016). Hypofractionated Stereotactic Body Radiation Therapy for the Definitive Treatment of Sarcoma. | 4 |
| 1. Raldow, A., Jacobson, A., Goldberg, S., Wang, H., Choy, E., Cote, G., ... & Chen, Y. L. E. (2016). Adult Rhabdomyosarcoma: A Retrospective Analysis of 40 Patients Treated at a Single Institution. *International Journal of Radiation Oncology, Biology, Physics*, *96*(2), E710. | 4 |
| 1. Saguem, I., Mellouli, M., Kallel, R., Triki, M., Sellami, M., Ksentini, M., ... & Boudawara, T. (2016, September). Clinico-pathologic characteristics of head and neck sarcomas: A retrospective analysis of 50 cases in a Tunisian institution. In *VIRCHOWS ARCHIV* (Vol. 469, No. SUPPL 1, pp. S116-S116). ONE NEW YORK PLAZA, SUITE 4600, NEW YORK, NY, UNITED STATES: SPRINGER. | 4 |
| 1. Moura I.M.,Delgado I.,Pacheco R.,Estibeiro H.,Ferreira L.,Montalvao P.,Magalhaes M.Pediatric Otolaryngology. Otolaryngol Head Neck Surg. 2016 Sep;155(1_suppl):P251-P265. doi: 10.1177/0194599816655337i. PMID: 30129830. | 4 |
| 1. Alex-Okoro J, Orji FT, Umedum NG, Akpeh JO. The comparison of the pathological data of oropharyngeal masses between HIV and non-HIV patients. Acta Otolaryngol. 2016 Sep;136(9):969-72. doi: 10.3109/00016489.2016.1170878. Epub 2016 Apr 22. PMID: 27103220. | 1 |
| 1. Ten Thije, L., Boon, E., Desar, I., Flucke, U. E., Van der Graaf, W. T., Ho, V. K., & Van Herpen, C. M. (2016). Prognostic factors for overall survival of patients with head and neck soft tissue sarcoma based on 25 years data from the Netherlands Cancer registry. | 4 |
| 1. Gopalakrishnan, V., Wagner, M., Amini, B., Lazar, A., Lin, P. P., Benjamin, R. S., & Araujo, D. M. (2016). Head and neck synovial sarcomas: Clinical characteristics and survival. | 4 |
| 1. Abdelfattah, A. M., Mohsen, E., Younes, A., Zagloul, M. S., Akosh, H., Taha, H., ... & Hafez, H. (2016, November). Outcome and Factors Affecting Survival in Children with Intermediate Risk Rhabdomyosarcoma: Children's Cancer Hospital Egypt 57357 Experience. In *PEDIATRIC BLOOD & CANCER* (Vol. 63, pp. S256-S256). 111 RIVER ST, HOBOKEN 07030-5774, NJ USA: WILEY-BLACKWELL. | 4 |
| 1. Carifi M, Dall'Olio D. Letter on the article "Head and neck sarcoma: Analysis of 29 cases". Eur Ann Otorhinolaryngol Head Neck Dis. 2016 Feb;133(1):77. doi: 10.1016/j.anorl.2015.11.009. Epub 2015 Dec 21. PMID: 26718847. | 4 |
| 1. Yoshida A, Goto K, Kodaira M, Kobayashi E, Kawamoto H, Mori T, Yoshimoto S, Endo O, Kodama N, Kushima R, Hiraoka N, Motoi T, Kawai A. CIC-rearranged Sarcomas: A Study of 20 Cases and Comparisons With Ewing Sarcomas. Am J Surg Pathol. 2016 Mar;40(3):313-23. doi: 10.1097/PAS.0000000000000570. PMID: 26685084. | 9 |
| 1. Karatayli-Ozgursoy S, Bishop JA, Hillel AT, Akst LM, Best SR. Non-epithelial tumors of the larynx: a single institution review. Am J Otolaryngol. 2016 May-Jun;37(3):279-85. doi: 10.1016/j.amjoto.2016.01.005. Epub 2016 Jan 27. PMID: 27178524. | 9 |
| 1. Gambarotti M, Righi A, Picci P, Bertoni F, Manfrini M, Donati DM, Dei Tos AP, Vanel D. Paediatric chondrosarcomas: a retrospective review of 17 cases. Histopathology. 2016 Jun;68(7):1073-8. doi: 10.1111/his.12881. Epub 2015 Nov 25. PMID: 26408960. | 6 |
| 1. Bains R, Magdum A, Bhat W, Roy A, Platt A, Stanley P. Soft tissue sarcoma - A review of presentation, management and outcomes in 110 patients. Surgeon. 2016 Jun;14(3):129-35. doi: 10.1016/j.surge.2014.06.002. Epub 2014 Sep 30. PMID: 25261278. | 2 |
| 1. Shao R, Lao IW, Wang L, Yu L, Wang J, Fan Q. Clinicopathologic and radiologic features of extraskeletal myxoid chondrosarcoma: a retrospective study of 40 Chinese cases with literature review. Ann Diagn Pathol. 2016 Aug;23:14-20. doi: 10.1016/j.anndiagpath.2016.04.004. Epub 2016 Apr 13. PMID: 27402218. | 9 |
| 1. Owosho AA, Brady P, Wolden SL, Wexler LH, Antonescu CR, Huryn JM, Estilo CL. Long-term effect of chemotherapy-intensity-modulated radiation therapy (chemo-IMRT) on dentofacial development in head and neck rhabdomyosarcoma patients. Pediatr Hematol Oncol. 2016 Sep;33(6):383-392. doi: 10.1080/08880018.2016.1219797. Epub 2016 Sep 30. PMID: 27689858; PMCID: PMC5175398. | 1 |
| 1. Rubio GA, Alvarado A, Gerth DJ, Tashiro J, Thaller SR. Incidence and Outcomes of Dermatofibrosarcoma Protuberans in the US Pediatric Population. J Craniofac Surg. 2017 Jan;28(1):182-184. doi: 10.1097/SCS.0000000000003203. PMID: 27922973. | 2 |
| 1. Malave B.C.D.,Barazarte A.,Acosta L.,Bastidas Y. Sinonasal rhabdomyosarcoma: Survival rate in children. Otolaryngology - Head and Neck Surgery (United States) 2017 157:1 (P295) Supplement 1 | 4 |
| 1. Akbari, M. E., Atarbashi-Moghadam, F., Atarbashi-Moghadam, S., Bastani, Z., & Zalani, S. S. (2017). Primary malignant neoplasms of parotid gland in Iranian population. *International Journal of Cancer Management*, *10*(11). | 1 |
| 1. Patel, M., Chaston, N., & Kichenaradjou, A. (2017). A single centre experience of head and neck cutaneous pleomorphic dermal sarcoma. *International Journal of Oral and Maxillofacial Surgery*, *46*, 292-293. | 4 |
| 1. Yau, B., Goh, M., McCormack, C., Tran, P., Daniel, B., & Webb, A. (2017, May). Atypical fibroxanthoma/pleomorphic dermal sarcoma at the Peter MacCallum Cancer Centre 2002-2016. In *AUSTRALASIAN JOURNAL OF DERMATOLOGY* (Vol. 58, pp. 23-24). 111 RIVER ST, HOBOKEN 07030-5774, NJ USA: WILEY. | 4 |
| 1. ChuOngsakul S.,Piyakulvorawat S.Surgical Management of Dermatofibrosarcoma Protuberans: A Siriraj Experience. [In Process] Journal of the Medical Association of Thailand 2017 100:5 (S121-S129) | 2 |
| 1. Madi, D., Aliasgar, A., & Ramapuram, J. (2017). P3. 50 Spectrum of malignancies among people living with hiv (PLHIV) in southern india. | 4 |
| 1. Logan, I., Vroobel, K., & Perrett, C. (2017, July). Pleomorphic dermal sarcoma: clinicopathological features and outcomes from a 5-year tertiary referral centre experience. In *BRITISH JOURNAL OF DERMATOLOGY* (Vol. 177, pp. 128-129). 111 RIVER ST, HOBOKEN 07030-5774, NJ USA: WILEY. | 4 |
| 1. Alfaar AS, Hassan WM, Bakry MS, Qaddoumi I. Neonates with cancer and causes of death; lessons from 615 cases in the SEER databases. Cancer Med. 2017 Jul;6(7):1817-1826. doi: 10.1002/cam4.1122. Epub 2017 Jun 22. PMID: 28639735; PMCID: PMC5504346. | 1 |
| 1. Chung SY, Unsal AA, Kılıç S, Baredes S, Liu JK, Eloy JA. Pediatric sinonasal malignancies: A population-based analysis. Int J Pediatr Otorhinolaryngol. 2017 Jul;98:97-102. doi: 10.1016/j.ijporl.2017.04.032. Epub 2017 Apr 25. PMID: 28583514. | 2 |
| 1. Lin, C. (2017). A Comparison of Pediatric Versus Adult Patients With Rhabdomyosarcoma. *International Journal of Radiation Oncology, Biology, Physics*, *99*(2), E569-E570. | 4 |
| 1. Ali, S., & Mohamedbhai, H. (2017). A Review of Malignancy and Site of Head and Neck Cancers Treated in a Tertiary Oral and Maxillofacial Surgery Unit: 0763. *International Journal of Surgery*, *47*, S51-S52. | 4 |
| 1. Lombardi D, Mattavelli D, Redaelli De Zinis LO, Accorona R, Morassi ML, Facchetti F, Ferrari V, Farina D, Bertulli R, Nicolai P. Primary Ewing's sarcoma of the sinonasal tract in adults: A challenging disease. Head Neck. 2017 Mar;39(3):E45-E50. doi: 10.1002/hed.24649. Epub 2016 Nov 29. PMID: 27898190. | 9 |
| 1. Tsuda Y, Ogura K, Hakozaki M, Kikuta K, Ae K, Tsuchiya H, Iwata S, Ueda T, Kawano H, Kawai A. Mesenchymal chondrosarcoma: A Japanese Musculoskeletal Oncology Group (JMOG) study on 57 patients. J Surg Oncol. 2017 May;115(6):760-767. doi: 10.1002/jso.24567. PMID: 29044531. | 9 |
| 1. Requena C, Sendra E, Llombart B, Sanmartín O, Guillén C, Lavernia J, Traves V, Cruz J. Cutaneous Angiosarcoma: Clinical and Pathology Study of 16 Cases. Actas Dermosifiliogr. 2017 Jun;108(5):457-465. English, Spanish. doi: 10.1016/j.ad.2017.01.014. Epub 2017 Mar 17. PMID: 28318524. | 9 |
| 1. Harati K, Daigeler A, Lange K, Niggemann H, Stricker I, Steinau HU, Lehnhardt M, Goertz O. Somatic Leiomyosarcoma of the Soft Tissues: A Single-Institutional Analysis of Factors Predictive of Survival in 164 Patients. World J Surg. 2017 Jun;41(6):1534-1541. doi: 10.1007/s00268-017-3899-5. PMID: 28116485. | 9 |
| 1. Iatrou I, Theologie-Lygidakis N, Schoinohoriti O, Tzermpos F, Vessala AM. Rhabdomyosarcoma of the maxillofacial region in children and adolescents: Report of 9 cases and literature review. J Craniomaxillofac Surg. 2017 Jun;45(6):831-838. doi: 10.1016/j.jcms.2017.03.005. Epub 2017 Mar 23. PMID: 28431807. | 9 |
| 1. Patel PB, Kuan EC, Peng KA, Yoo F, Nelson SD, Abemayor E. Angiosarcoma of the tongue: A case series and literature review. Am J Otolaryngol. 2017 Jul-Aug;38(4):475-478. doi: 10.1016/j.amjoto.2017.04.013. Epub 2017 Apr 21. PMID: 28478092. | 9 |
| 1. Raza SM, Gidley PW, Meis JM, Grosshans DR, Bell D, DeMonte F. Multimodality Treatment of Skull Base Chondrosarcomas: The Role of Histology Specific Treatment Protocols. Neurosurgery. 2017 Sep 1;81(3):520-530. doi: 10.1093/neuros/nyx042. Erratum in: Neurosurgery. 2017 Dec 1;81(6):1047. doi: 10.1093/neuros/nyx557. PMID: 28368506. | 6 |
| 1. Chen E, Ricciotti R, Futran N, Oda D. Head and Neck Rhabdomyosarcoma: Clinical and Pathologic Characterization of Seven Cases. Head Neck Pathol. 2017 Sep;11(3):321-326. doi: 10.1007/s12105-016-0771-0. Epub 2016 Nov 28. PMID: 27896667; PMCID: PMC5550390. | 9 |
| 1. Bansal D, Das A, Trehan A, Kapoor R, Panda NK, Srinivasan R, Panda NK, Srinivasan R, Kakkar N, Sodhi KS, Saxena AK, Narasimha Rao KL. Pediatric Rhabdomyosarcoma in India: A Single-center Experience. Indian Pediatr. 2017 Sep 15;54(9):735-738. doi: 10.1007/s13312-017-1164-5. PMID: 28984250. | 2 |
| 1. Kakkar N, Gupta A, Sharma NK, Agarwal P, Kaur J. Adolescents and young adults: A study of distribution of cancer at ages 15-39 years in a tertiary care hospital from North India: Epidemiological considerations. South Asian J Cancer. 2017 Oct-Dec;6(4):180-182. doi: 10.4103/sajc.sajc_263_16. PMID: 29404301; PMCID: PMC5763633. | 1 |
| 1. Swain, S. K., Samal, S., Sahu, M. C., & Debta, P. (2018). Synovial Sarcoma of the Head and Neck Region-Our Experiences at a Tertiary Care Hospital of Eastern India. *Indian Journal of Public Health Research & Development*, *9*(11). | 9 |
| 1. Kakkar, A., Rajeshwari, M., Sakthivel, P., Sharma, M. C., & Sharma, S. C. (2018). Biphenotypic sinonasal sarcoma: a series of six cases with evaluation of role of β-catenin immunohistochemistry in differential diagnosis. *Annals of Diagnostic Pathology*, *33*, 6-10. | 9 |
| 1. Sun, M., Liu, J. G., Weng, Q. Y., Yu, L., & Wang, J. (2018). Pleomorphic and dedifferentiated leiomyosarcoma: a clinicopathologic analysis. *Zhonghua Bing li xue za zhi= Chinese Journal of Pathology*, *47*(2), 87-93. | 9 |
| 1. Gore, M. R. (2018). Survival in sinonasal and middle ear malignancies: a population-based study using the SEER 1973–2015 database. *BMC Ear, Nose and Throat Disorders*, *18*, 1-11. | 2 |
| 1. Khiavi, M. M., Haghi-Ashtiani, M. T., Kharazi-Fard, M. J., & Kalantar, R. (2018). Frequency of head and neck masses in iranian children during a 21-year period. *Iranian Journal of Pediatrics*, *28*(6). | 1 |
| 1. Effiom OA, Olojede ACO, Akinde OR, Olawuyi AB, Amoo AT, Arotiba GT. Dermatofibrosarcoma protuberans: clinicopathologic presentation in Nigerians. Pan Afr Med J. 2018 Sep 12;31:25. doi: 10.11604/pamj.2018.31.25.13665. PMID: 30918552; PMCID: PMC6430858. | 9 |
| 1. Yazici S, Zorlu O, Bulbul Baskan E, Balaban Adim S, Aydogan K, Saricaoglu H. Retrospective Analysis of 91 Kaposi's Sarcoma Cases: A Single-Center Experience and Review of the Literature. Dermatology. 2018;234(5-6):205-213. doi: 10.1159/000492112. Epub 2018 Sep 28. PMID: 30269141. | 2 |
| 1. Adoga, A. A., Kokong, D. D., Ma’an, N. D., Silas, O. A., Dauda, A. M., Yaro, J. P., ... & Yabak, C. J. (2018). The epidemiology, treatment, and determinants of outcome of primary head and neck cancers at the Jos University Teaching Hospital. *South Asian journal of cancer*, *7*(03), 183-187. | 1 |
| 1. Hei, Y., Kang, L., Yang, X., Wang, Y., Lu, X., Li, Y., ... & Xiao, L. (2018). Orbital alveolar soft part sarcoma: a report of 8 cases and review of the literature. *Oncology Letters*, *15*(1), 304-314. | 9 |
| 1. Innocent, E., Manasseh, A. N., Badoe, E. V., Daniel, Y., Vandi, K. B., Olanrewaju, A., & Graham, E. U. (2018). Rhabdomyosarcoma in All Age Groups at the Jos University Teaching Hospital. *West African Journal of Medicine*, *35*(2), 97-101. | 2 |
| 1. Kim YH, Jeon C, Se YB, Hong SD, Seol HJ, Lee JI, Park CK, Kim DG, Jung HW, Han DH, Nam DH, Kong DS. Clinical outcomes of an endoscopic transclival and transpetrosal approach for primary skull base malignancies involving the clivus. J Neurosurg. 2018 May;128(5):1454-1462. doi: 10.3171/2016.12.JNS161920. Epub 2017 Jun 2. PMID: 28574308. | 1 |
| 1. Schreiber A, Rampinelli V, Ferrari M, Mattavelli D, Farina D, Battocchio S, Nicolai P. Diagnostic reliability of pretreatment biopsy in malignant nasoethmoidal tumors: A retrospective study of 77 cases. Laryngoscope. 2018 Aug;128(8):1772-1777. doi: 10.1002/lary.27077. Epub 2018 Jan 4. PMID: 29314070. | 9 |
| 1. Albasri AM, Borhan WM. Histopathological pattern of skin cancer in Western region of Saudi Arabia. An 11 years experience. Saudi Med J. 2018 Oct;39(10):994-998. doi: 10.15537/smj.2018.10.22679. PMID: 30284581; PMCID: PMC6201021. | 9 |
| 1. Agaimy A, Semrau S, Koch M, Thompson LDR. Sinonasal Leiomyosarcoma: Clinicopathological Analysis of Nine Cases with Emphasis on Common Association with Other Malignancies and Late Distant Metastasis. Head Neck Pathol. 2018 Dec;12(4):463-470. doi: 10.1007/s12105-017-0876-0. Epub 2017 Dec 21. PMID: 29270859; PMCID: PMC6232217. | 9 |
| 1. Raza SM, Gidley PW, Kupferman ME, Hanna EY, Su SY, DeMonte F. Site-Specific Considerations in the Surgical Management of Skull Base Chondrosarcomas. Oper Neurosurg (Hagerstown). 2018 Jun 1;14(6):611-619. doi: 10.1093/ons/opx171. PMID: 28962038. | 6 |
| 1. Soyele OO, Effiom OA, Lawal AO, Nwoga MC, Adebiyi KE, Aborisade A, Olatunji AS, Olawuyi AB, Ladeji AM, Okiti RO, Adeola HA. A multi-centre evaluation of malignant odontogenic tumours in Nigeria. Pan Afr Med J. 2019 May 10;33:18. doi: 10.11604/pamj.2019.33.18.16179. PMID: 31312334; PMCID: PMC6615768. | 2 |
| 1. Gootee J, Aurit S, Curtin C, Silberstein P. Primary anatomical site, adjuvant therapy, and other prognostic variables for dedifferentiated liposarcoma. J Cancer Res Clin Oncol. 2019 Jan;145(1):181-192. doi: 10.1007/s00432-018-2777-3. Epub 2018 Oct 25. PMID: 30361927. | 2 |
| 1. Bai YX, Ma YY, Feng JY, Liu XM, Chen L. [Clinicopathological features and prognosis of pediatric alveolar rhabdomyosarcoma]. Zhonghua Bing Li Xue Za Zhi. 2019 Sep 8;48(9):710-714. Chinese. doi: 10.3760/cma.j.issn.0529-5807.2019.09.009. PMID: 31495092. | 9 |
| 1. Miglani, A., Lal, D., Weindling, S. M., Wood, C. P., & Hoxworth, J. M. (2019). Imaging characteristics and clinical outcomes of biphenotypic sinonasal sarcoma. *Laryngoscope investigative otolaryngology*, *4*(5), 484-488. | 9 |
| 1. Turpin B, Pressey JG, Nagarajan R, Weiss BD, Trout AT, Gelfand MJ, Pater L, Vatner RE, Breneman JC, Dasgupta R. Sentinel lymph node biopsy in head and neck rhabdomyosarcoma. Pediatr Blood Cancer. 2019 Mar;66(3):e27532. doi: 10.1002/pbc.27532. Epub 2018 Nov 4. PMID: 30393936. | 9 |
| 1. Tsai JW, ChangChien YC, Lee JC, Kao YC, Li WS, Liang CW, Liao IC, Chang YM, Wang JC, Tsao CF, Yu SC, Huang HY. The expanding morphological and genetic spectrum of MYOD1-mutant spindle cell/sclerosing rhabdomyosarcomas: a clinicopathological and molecular comparison of mutated and non-mutated cases. Histopathology. 2019 May;74(6):933-943. doi: 10.1111/his.13819. Epub 2019 Apr 4. PMID: 30604891. | 9 |
| 1. Saluja TS, Iyer J, Singh SK. Leiomyosarcoma: Prognostic outline of a rare head and neck malignancy. Oral Oncol. 2019 Aug;95:100-105. doi: 10.1016/j.oraloncology.2019.06.010. Epub 2019 Jun 13. PMID: 31345375. | 4 |
| 1. Saraydaroglu O, Narter S, Ozsen M, Coskun H. Non-epithelial tumors of the larynx: case series of 12 years. Eur Arch Otorhinolaryngol. 2019 Oct;276(10):2843-2847. doi: 10.1007/s00405-019-05527-0. Epub 2019 Jun 29. PMID: 31256243. | 9 |
| 1. Chou PY, Kao D, Denadai R, Huang CY, Lin CH, Lin CH. Anterolateral thigh free flaps for the reconstruction of scalp angiosarcoma - 18-year experience in Chang Gung memorial hospital. J Plast Reconstr Aesthet Surg. 2019 Dec;72(12):1900-1908. doi: 10.1016/j.bjps.2019.07.024. Epub 2019 Aug 8. PMID: 31519502. | 9 |
| 1. Huang AY, Lin CL, Chen GS, Hu SC. Clinical features of Kaposi's sarcoma: experience from a Taiwanese medical center. Int J Dermatol. 2019 Dec;58(12):1388-1397. doi: 10.1111/ijd.14476. Epub 2019 May 17. PMID: 31102268. | 9 |
| 1. Yuce Sari S, Cengiz M, Dauletkazin A, Yazici G, Gultekin M, Hurmuz P, Yildiz F, Zorlu F, Gurkaynak M, Akyol F, Ozyigit G. Hypofractionated radiotherapy for non-metastatic bone and soft tissue sarcomas. Cancer Radiother. 2019 Dec;23(8):853-859. doi: 10.1016/j.canrad.2019.06.011. Epub 2019 Oct 19. PMID: 31640927. | 2 |
| 1. Raza SM, Habib A, Wang WL, Gildey PW, Conley AP, Nader ME, Hanna EY, Su SY, DeMonte F. Surgical Management of Primary Skull Base Osteosarcomas: Impact of Margin Status and Patterns of Relapse. Neurosurgery. 2020 Jan 1;86(1):E23-E32. doi: 10.1093/neuros/nyz360. PMID: 31515560. | 6 |
| 1. Masele, A., Sohal, K. S., Kalyanyama, B. M., Owibingire, S. S., & Simon, E. N. (2020). Head and neck lesions among HIV/AIDS patients on highly active antiretroviral therapy attending the Muhimbili National Hospital in Dar es Salaam, Tanzania. *Frontiers of Oral and Maxillofacial Medicine*, *2*. | 2 |
| 1. Rahman, A. A., Begum, M., Kibria, C. S. H., Akter, M., Siddiqua, K. A., Siddiqua, F., ... & Shah, S. (2020). Outcome of paediatric rhabdomyosarcoma attended in a tertiary care hospital, Dhaka, Bangladesh. *Bangladesh Medical Research Council Bulletin*, *46*(1), 17-21. | 2 |
| 1. Bastian, T., Mattheis, S., Lang, S., & Hussain, T. (2020). Treatment approaches and oncologic outcomes of 35 patients with sarcomas of the head and neck–a retrospective analysis. *Laryngo-Rhino-Otologie*, *99*(S 02). | 4 |
| 1. Cheng J, Jung SH, Gao J. Clinical Factors Affecting Survival in Pediatric Vascular Tumors of the Head and Neck. J Craniofac Surg. 2020 May/Jun;31(3):628-629. doi: 10.1097/SCS.0000000000006040. PMID: 32028358. | 9 |
| 1. Smrke, A., Hamm, J., Karvat, A., Simmons, C., & Srikanthan, A. (2020). A retrospective review of 145 patients with angiosarcoma: Radiation therapy, extent of resection and chemotherapy are important predictors of survival. *Molecular and Clinical Oncology*, *13*(2), 179-185. | 2 |
| 1. Yang L, Zhang HJ, Yang SJ. [Spindle cell/sclerosing rhabdomyosarcoma: a clinicopathological study of 20 cases]. Zhonghua Bing Li Xue Za Zhi. 2020 Apr 8;49(4):336-342. Chinese. doi: 10.3760/cma.j.cn112151-20190816-00453. PMID: 32268670. | 9 |
| 1. Colas M, Gérazime A, Popescu D, Puzenat E, Chaigneau L, Woronoff AS, Dupond AS, Nardin C, Aubin F. Angiosarcoma: A population-based cancer registry descriptive study of 45 consecutive cases diagnosed between 1979 and 2016. Rare Tumors. 2020 Dec 14;12:2036361320979216. doi: 10.1177/2036361320979216. PMID: 33403092; PMCID: PMC7739202. | 9 |
| 1. Fritchie K, Ghosh T, Graham RP, Roden AC, Schembri-Wismayer D, Folpe A, Rivera M. Well-Differentiated/Dedifferentiated Liposarcoma Arising in the Upper Aerodigestive Tract: 8 Cases Mimicking Non-adipocytic Lesions. Head Neck Pathol. 2020 Dec;14(4):974-981. doi: 10.1007/s12105-020-01171-x. Epub 2020 May 14. PMID: 32410132; PMCID: PMC7669978. | 9 |
| 1. Ning Z, Liu X, Qin G, Wei L, Li X, Shen J. Evaluation of clinical efficacy of Chemotherapy for Rhabdomyosarcoma in children. Pak J Med Sci. 2020 Jul-Aug;36(5):1069-1074. doi: 10.12669/pjms.36.5.1829. PMID: 32704291; PMCID: PMC7372667. | 2 |
| 1. Verma, H., Sehgal, K., Panchal, K. B., Chakraborty, S., Biswas, B., Mukherjee, G., ... & Biswas, G. (2020). Presentation and management of dermatofibrosarcoma protuberans: a single center protocol. *Indian Journal of Surgical Oncology*, *11*, 35-40. | 9 |
| 1. Martinez AP, Zapata M, North PE, Folpe AL, Weiss SW. Lymphatic-type "Angiosarcoma" With Prominent Lymphocytic Infiltrate. Am J Surg Pathol. 2020 Feb;44(2):271-279. doi: 10.1097/PAS.0000000000001398. PMID: 31688141. | 2 |
| 1. George, A. P., Markiewicz, M. R., Garzon, S., & Choi, D. K. (2020). Adolescent and young adult oral maxillofacial tumors: a single-institution case series and literature review. *Journal of Adolescent and Young Adult Oncology*, *9*(2), 307-312. | 9 |
| 1. Hasan, S. (2020). AL-Ghamdi. The prevalence and histological patterns of malignant skin tumors in Albaha, Saudi Arabia: A retrospective study. *Medical Science*, *24*(105), 2767-2774. | 9 |
| 1. Hagerty, B. L., Aversa, J., Diggs, L. P., Dominguez, D. A., Ayabe, R. I., Blakely, A. M., ... & Hernandez, J. M. (2020). Characterization of alveolar soft part sarcoma using a large national database. *Surgery*, *168*(5), 825-830. | 2 |
| 1. Bansal M. Otorhinolaryngology Malignancies in Children: A Case Series. Indian J Otolaryngol Head Neck Surg. 2020 Dec;72(4):443-447. doi: 10.1007/s12070-020-01881-2. Epub 2020 May 14. PMID: 33088772; PMCID: PMC7544770. | 9 |
| 1. Scheer M, Vokuhl C, Bauer S, Fuchs J, Loff S, Timmermann B, Münter M, Henssen AG, Kazanowska B, Niggli F, Ladenstein R, Ljungman G, Koscielniak E, Klingebiel T; European Cooperative Weichteilsarkom Studiengruppe [CWS]. The effect of adjuvant therapies on long-term outcome for primary resected synovial sarcoma in a series of mainly children and adolescents. J Cancer Res Clin Oncol. 2021 Dec;147(12):3735-3747. doi: 10.1007/s00432-021-03614-6. Epub 2021 Jul 17. PMID: 34272609; PMCID: PMC8557198. | 2 |
| 1. Lin N, Liu X, Zhang F, Pan Y, Qi M, Sha Y. Sinonasal synovial sarcoma: evaluation of the role of radiological and clinicopathological features in diagnosis. Clin Radiol. 2021 Jan;76(1):78.e1-78.e8. doi: 10.1016/j.crad.2020.08.007. Epub 2020 Sep 28. PMID: 32896427. | 9 |
| 1. Azman, M., Sahab, S. H., Zahedi, F. D., bin Mohamed Yunus, M. R., Abd Jabar, N., & Ismail, F. (2021). A 10-Year Kuala Lumpur Review on Maxillary Sinus Tumour. *International Medical Journal*, *28*(1), 59-63. | 9 |
| 1. Stradomskaya, T. V., Kachanov, D. Y., Grachev, N. S., Nechesnyuk, A. V., Teleshova, M. V., Akhaladze, D. G., ... & Novichkova, G. A. (2021). Rhabdomyosarcoma in the first year of life. Experience of Dmitry Rogachev National Medical Research Center of Pediatric Hematology, Oncology and Immunology. *Prognostic significance of various 11q23/KMT2A rearrangements in infants with acute lymphoblastic leuekemia*, *20*(1), 77. | 9 |
| 1. Sharma MR, Puj KS, Salunke AA, Pandya SJ, Gandhi JS, Parikh AR. Malignant peripheral nerve sheath tumor with analysis of various prognostic factors: A single-institutional experience. J Cancer Res Ther. 2021 Jan-Mar;17(1):106-113. doi: 10.4103/jcrt.JCRT_854_19. PMID: 33723140. | 9 |
| 1. Renzi S, Cullinan N, Cohen-Gogo S, Langenberg-Ververgaert K, Michaeli O, Alkendi J, Kanwar N, Lo W, Villani A, Shlien A, Malkin D, Ryan AL, Gallinger B, Ingley K, Hopyan S, Gupta A, Chami R. Non-rhabdomyosarcoma soft tissue sarcomas diagnosed in patients at a young age. An overview of clinical, pathological, and molecular findings. Pediatr Blood Cancer. 2021 Aug;68(8):e29022. doi: 10.1002/pbc.29022. Epub 2021 Mar 25. PMID: 33764675. | 9 |
| 1. Priya K.,Vandana,Bariar N.K.A Retrospective Evaluation of Soft Tissue Tumors in Tertiary Care Centre: A Clinic-Pathological Investigation. International Journal of Pharmaceutical and Clinical Research 2021 13:6 (676-682) | 9 |
| 1. Bastakoti, S., Shrestha, G., Gautam, D. K., Dhungana, I., Jha, N., Pandey, G., ... & Bhatta, R. R. (2021). Clinico-pathological spectrum of oral cavity lesions at a tertiary care center in central nepal: a descriptive cross-sectional study. *JNMA: Journal of the Nepal Medical Association*, *59*(234), 124. | 9 |
| 1. Benoit C, Orbach D, Cyrille S, Belhous K, Minard-Colin V, Kadlub N, Kolb F, Reguerre Y, Carton M, Bolle S, Helfre S, Van Den Abbeele T, Luscan R, Hartl DM, Galmiche L, Petit A, Maiz M, Couloigner V, Elmaleh M, Bernard S. Head and neck tumors in children and adolescents: Impact of a multidisciplinary tumor board. Oral Oncol. 2021 Mar;114:105145. doi: 10.1016/j.oraloncology.2020.105145. Epub 2021 Jan 20. PMID: 33482589. | 1 |
| 1. Xu Y, Xu G, Wang X, Mao M, Wu H, Baklaushev VP, Chekhonin VP, Peltzer K, Wang G, Zhang C. Is there a role for chemotherapy and radiation in the treatment of patients with low-grade myofibroblastic sarcoma? Clin Transl Oncol. 2021 Feb;23(2):344-352. doi: 10.1007/s12094-020-02425-4. Epub 2020 Jun 30. PMID: 32607812. | 2 |
| 1. Buszek SM, Ludmir EB, Grosshans DR, McAleer MF, McGovern SL, Harrison DJ, Okcu MF, Chintagumpala MM, Mahajan A, Paulino AC. Disease Control and Patterns of Failure After Proton Beam Therapy for Rhabdomyosarcoma. Int J Radiat Oncol Biol Phys. 2021 Mar 1;109(3):718-725. doi: 10.1016/j.ijrobp.2020.09.050. PMID: 33516439. | 2 |
| 1. Ogun GO, Ezenkwa US, Babatunde TO, Obiagwu AE, Nweke MC, Adegoke OO, Olulana OO, Brown BJ. Paediatric soft tissue sarcomas in a resource constraint setting: Grade and stage at presentation and at oncologic intervention are usually of poor prognostic characteristics. Int J Clin Pract. 2021 Apr;75(4):e13951. doi: 10.1111/ijcp.13951. Epub 2021 Jan 9. PMID: 33342027. | 2 |
| 1. Soares Queirós C, Filipe P, Soares de Almeida L. Cutaneous leiomyosarcoma: a 20-year retrospective study and review of the literature. An Bras Dermatol. 2021 May-Jun;96(3):278-283. doi: 10.1016/j.abd.2020.10.003. Epub 2021 Mar 21. PMID: 33775481; PMCID: PMC8178579. | 9 |
| 1. Brown BJ, Ogun GO, Akinmoladun VI, Ogundoyin OO, Abdus-Salam A, Olulana DI, Lawal TA. Clinicopathologic Features of Childhood Rhabdomyosarcoma and Treatment Outcomes in Ibadan, Nigeria: A 10-year Review. J Pediatr Hematol Oncol. 2021 Jul 1;43(5):e625-e629. doi: 10.1097/MPH.0000000000002093. PMID: 33625089. | 2 |
| 1. Merna C, Lehrich BM, Diaz-Aguilar LD, Goshtasbi K, Sahyouni R, Hsu FPK, Kuan EC. Determinants of Survival in Skull Base Osteosarcoma: A National Cancer Database Study. World Neurosurg. 2021 Jul;151:e828-e838. doi: 10.1016/j.wneu.2021.04.135. Epub 2021 May 8. PMID: 33974986. | 6 |
| 1. Gassenmaier M, Weber E, Leiter U, Hahn M, Forchhammer S, Häfner HM, Scheu A, Garbe C, Schnabl S. Micrographic Surgery Allows Fascia Preservation in Dermatofibro-sarcoma Protuberans. Acta Derm Venereol. 2021 Sep 28;101(9):adv00561. doi: 10.2340/00015555-3915. PMID: 34490467; PMCID: PMC9425625. | 9 |
| 1. Shim T, Chillakuru Y, Darwish C, Chalif E, Strum D, Benito DA, Mulcahy CF, Monfared A. Head and neck osteosarcomas: Analysis of treatment trends and survival outcomes in the United States (2004-2016). Head Neck. 2021 Nov;43(11):3294-3305. doi: 10.1002/hed.26817. Epub 2021 Jul 17. PMID: 34272901. | 8 |
| 1. Koka K, Rahim FE, El-Hadad C, Bell D, Debnam JM, Guo Y, Esmaeli B. Primary Ewing's sarcoma with orbit involvement: Survival and visual outcomes after eye-sparing multidisciplinary management in eight patients. Head Neck. 2021 Dec;43(12):3857-3865. doi: 10.1002/hed.26884. Epub 2021 Oct 10. PMID: 34632670. | 9 |
| 1. Houdek MT, Tsoi KM, Mallett KE, Claxton RM, Ferguson PC, Griffin AM, Baum CL, Brewer JD, Rose PS, Wunder JS. Surgical Outcomes of Primary Dermatofibrosarcoma Protuberans: A Retrospective, Multicenter Study. Ann Surg Oncol. 2022 Dec;29(13):8632-8638. doi: 10.1245/s10434-022-12351-0. Epub 2022 Aug 6. PMID: 35933538. | 6 |
| 1. Swain PK, Singh S, Bhalgat BS, Kumar P, Lakhera KK, Patel P, Sharma RG. Distribution of Cutaneous Malignancies in Eastern Rajasthan: A Five-Year Study. Indian J Dermatol. 2022 Nov-Dec;67(6):728-731. doi: 10.4103/ijd.ijd_953_21. PMID: 36998877; PMCID: PMC10043657. | 9 |
| 1. Darrigo Junior LG, Ferraz VEF, Cormedi MCV, Araujo LHH, Magalhães MPS, Carneiro RC, Sales LHN, Suchmacher M, Cunha KS, Filho AB, Azulay DR, Geller M. Epidemiological profile and clinical characteristics of 491 Brazilian patients with neurofibromatosis type 1. Brain Behav. 2022 Jun;12(6):e2599. doi: 10.1002/brb3.2599. Epub 2022 May 4. PMID: 35506373; PMCID: PMC9226847. | 1 |
| 1. Long S, Asimakopoulos P, McGill M, Cohen MA, Patel SG, Shah JP, Ganly I. Anterior Skull Base Sarcomas: Report of Characteristics and Outcomes at a Tertiary Care Cancer Center. J Neurol Surg B Skull Base. 2021 Jan 21;83(3):265-269. doi: 10.1055/s-0040-1722667. PMID: 35769803; PMCID: PMC9236737. | 6 |
| 1. Velez Torres JM, Duarte EM, Diaz-Perez JA, Leibowitz J, Weed DT, Thomas G, Sargi Z, Civantos FJ, Arnold DJ, Gomez-Fernandez C, Montgomery EA, Rosenberg AE. Mesenchymal Neoplasms of Salivary Glands: A Clinicopathologic Study of 68 Cases. Head Neck Pathol. 2022 Jun;16(2):353-365. doi: 10.1007/s12105-021-01360-2. Epub 2021 Jul 12. PMID: 34251596; PMCID: PMC9187808. | 9 |
| 1. Rosenbaum E, Antonescu CR, Smith S, Bradic M, Kashani D, Richards AL, Donoghue M, Kelly CM, Nacev B, Chan JE, Chi P, Dickson MA, Keohan ML, Gounder MM, Movva S, Avutu V, Thornton K, Zehir A, Bowman AS, Singer S, Tap W, D'Angelo S. Clinical, genomic, and transcriptomic correlates of response to immune checkpoint blockade-based therapy in a cohort of patients with angiosarcoma treated at a single center. J Immunother Cancer. 2022 Apr;10(4):e004149. doi: 10.1136/jitc-2021-004149. PMID: 35365586; PMCID: PMC8977792. | 2 |
| 1. Agaram NP, Huang SC, Tap WD, Wexler LH, Antonescu CR. Clinicopathologic and survival correlates of embryonal rhabdomyosarcoma driven by RAS/RAF mutations. Genes Chromosomes Cancer. 2022 Mar;61(3):131-137. doi: 10.1002/gcc.23010. Epub 2021 Nov 16. PMID: 34755412; PMCID: PMC8956004. | 9 |
| 1. Shen Y, Wang L, Cao H. [Clinical analysis of 5 adult laryngeal rhabdomyosarcoma]. Lin Chuang Er Bi Yan Hou Tou Jing Wai Ke Za Zhi. 2023 Dec;37(12):1014-1018. Chinese. doi: 10.13201/j.issn.2096-7993.2023.12.018. PMID: 38114324; PMCID: PMC10985694. | 9 |
| 1. Lopez J, Subramanian T, Stambuk H, Schreyer M, Woods R, Scholfield D, Wong R, Cohen MA, Shah J, Ganly I. CAD/CAM-assisted ablative surgery and intraoperative brachytherapy for pediatric skull-base sarcomas. Head Neck. 2023 Dec;45(12):E61-E66. doi: 10.1002/hed.27534. Epub 2023 Oct 10. PMID: 37814997; PMCID: PMC11187774. | 6 |
| 1. Li L, Ma XK, Gao Y, Wang DC, Dong RF, Yan J, Zhang R. Clinicopathological study of malignant peripheral nerve sheath tumors in the head and neck: Case reports and review of literature. World J Clin Cases. 2023 Sep 6;11(25):5910-5918. doi: 10.12998/wjcc.v11.i25.5910. PMID: 37727493; PMCID: PMC10506041. | 9 |
| 1. AlOtaibi MN, Basfar AS, Jawhari AM, Alzahrani ES, Althomali MA, Alhindi AE, Alam SS, Al Aboud DM, Abdel-Moneim AS. The Burden of Skin Cancers in Saudi Arabia Through 2011-2022. Cureus. 2023 Sep 11;15(9):e45052. doi: 10.7759/cureus.45052. PMID: 37829962; PMCID: PMC10566748. | 9 |
| 1. Zhu S, Xu N, Zhi T, Gao Y, Zhong D, Zhang W, Jin M, Sun Q, Xie Y, Zhang X, Li L, Wang S, Wang H, Liu R, Zhao W, Huang D, Ni X, Ma X. Clinical features and outcomes of infantile soft-tissue sarcoma: A multicenter retrospective study in Beijing. J Cancer Res Ther. 2023 Aug;19(4):876-880. doi: 10.4103/jcrt.jcrt_1950_22. PMID: 37675711. | 2 |
| 1. Tomassen T, Versleijen-Jonkers YMH, Hillebrandt-Roeffen MHS, Van Cleef PHJ, van Dalen T, Weidema ME, Desar IME, Flucke U, van Gorp JM. Prognostic Factors in Epithelioid Hemangioendothelioma: Analysis of a Nationwide Molecularly/Immunohistochemically Confirmed Cohort of 57 Cases. Cancers (Basel). 2023 Jun 23;15(13):3304. doi: 10.3390/cancers15133304. PMID: 37444414; PMCID: PMC10340645. | 9 |
| 1. Liu D, Wang JZ, Sun JB, Li Z, Zhang T, Sai N, Zhu YH, Shen WD, Huang DL, Dai P, Yang SM, Han DY, Han WJ. [Differential diagnosis and surgical management in chondrosarcoma of the jugular foramen]. Zhonghua Er Bi Yan Hou Tou Jing Wai Ke Za Zhi. 2023 Jun 7;58(6):544-551. Chinese. doi: 10.3760/cma.j.cn115330-20220607-00334. PMID: 37339893. | 6 |
| 1. Kromer CM, Yacoub N, Xiong D, Knackstedt T. Analysis of Survival Differences Between Cutaneous and Subcutaneous Malignant Peripheral Nerve Sheath Tumors. Dermatol Surg. 2023 Apr 1;49(4):322-329. doi: 10.1097/DSS.0000000000003717. Epub 2023 Feb 9. PMID: 36763849. | 7 |
| 1. Pehlivan M, İribaş A, Bilgiç B, Başaran M, Ekenel M. Clinical course and features of soft tissue sarcomas in geriatric patients: a single-center experience. J Int Med Res. 2023 Mar;51(3):3000605231159319. doi: 10.1177/03000605231159319. PMID: 36879483; PMCID: PMC9996734. | 9 |
| 1. McClure E, Carr MJ, Patel A, Hussnain Naqvi SM, Kim Y, Harrington M, Cruse W, Gonzalez RJ, Sondak VK, Sarnaik AA, Messina JL, Zager JS. Atypical Fibroxanthoma: Outcomes from a Large Single Institution Series. Cancer Control. 2023 Jan-Dec;30:10732748231155699. doi: 10.1177/10732748231155699. PMID: 36764930; PMCID: PMC9926370. | 2 |
| 1. Sanal M.S.,Rasheed A. A Study of the Incidence, Demographic Distribution, Predisposing Factors of Malignancies of the Oral Cavity at a Tertiary Care Center, South India. International Journal of Pharmaceutical and Clinical Research 2023 15:5 (1815-1820) | 7 |
| 1. Srijan R.B.,Vijaya Praveen Kumar P.,Ramya S.,Ramana P.V. Analysis of spectrum of neoplastic lesions of nasal cavity and paranasal sinuses. Journal of Cardiovascular Disease Research 2023 14:5 (1738-1748) | 9 |
| 1. Anschuetz, L., Hohenberger, R., Kaecker, C., Elicin, O., Giger, R., & Caversaccio, M. (2023). Sinonasal malignancies: Histopathological entities, regional involvement and long-term outcome. *Journal of Otolaryngology-Head & Neck Surgery*, *52*(1), s40463-023. | 9 |
| 1. Novillo M, Albergo JI, Huespe I, Latallade V, Farfalli GL, Ayerza MÁ, Roitman P, Cayol F, Aponte-Tinao LA. Resultados oncológicos y factores pronósticos en pacientes con sarcoma sinovial tratados quirúrgicamente [Oncological outcomes and prognostic factors in surgically treated patients with synovial sarcoma]. Medicina (B Aires). 2023;83(5):737-743. Spanish. PMID: 37870331. | 9 |
| 1. Markiz SN, Khan S, Wagley ZB, Viqaruddin MK, Khafaga YM, AlFawaz IA, AlAnazi AE, AlKofide A, Khoja HA, Ali AA. Rhabdomyosarcoma in children: Retrospective analysis from a single tertiary care center in Saudi Arabia. Cancer Rep (Hoboken). 2023 Jan;6(1):e1683. doi: 10.1002/cnr2.1683. Epub 2022 Aug 9. PMID: 35942988; PMCID: PMC9875671. | 2 |
| 1. Krishnan, S., Salian, V., D'souza, N., & Shetty, P. (2023). Mesenchymal tumours of the head and neck: a 10-year institutional archival study. *Revista Cubana de Investigaciones Biomédicas*, *42*. | 2 |
| 1. Coleman M, Liang J, Rastatter JC, Arch RS, Gartrell J, Chelius DC Jr, Sheyn A, Li C, Richard C. Exploring the Epidemiology and Survival Trends in Pediatric Major Salivary Gland Malignancies: Insights from the National Cancer Database. Curr Oncol. 2023 Jun 25;30(7):6134-6147. doi: 10.3390/curroncol30070456. PMID: 37504316; PMCID: PMC10378439. | 2 |
| 1. Sharma J, Deo SVS, Kumar S, Bhoriwal S, Kar M, Barwad AW, Thulkar S, Bakhshi S, Sharma DN. Demographic and clinical profile of 1106 adult soft tissue sarcoma patients: A single institutional prospective database experience from India. Asia Pac J Clin Oncol. 2024 Jun;20(3):386-394. doi: 10.1111/ajco.14050. Epub 2024 Feb 21. PMID: 38383968. | 2 |
| 1. Bielack SS, Mettmann V, Baumhoer D, Blattmann C, Burkhardt B, Deinzer CKW, Kager L, Kevric M, Mauz-Körholz C, Müller-Abt P, Reinhardt D, Sabo AA, Schrappe M, Sorg B, Windhager R, Hecker-Nolting S. Osteosarcoma Arising as a Secondary Malignancy following Treatment for Hematologic Cancer: A Report of 33 Affected Patients from the Cooperative Osteosarcoma Study Group (COSS). Cancers (Basel). 2024 May 11;16(10):1836. doi: 10.3390/cancers16101836. PMID: 38791915; PMCID: PMC11120238. | 9 |
| 1. Kobayashi H, Okajima K, Zhang L, Hirai T, Ishibashi Y, Tsuda Y, Ikegami M, Kawai A, Tanaka S. Prognostic factors and treatment outcomes in patients with pleomorphic rhabdomyosarcoma: a population-based cohort study. Jpn J Clin Oncol. 2024 Apr 6;54(4):471-478. doi: 10.1093/jjco/hyad188. PMID: 38183215. | 9 |
| 1. Tsuji T, Asato R, Kada S, Kitamura M, Tamaki H, Mizuta M, Tanaka S, Watanabe Y, Hori R, Kojima T, Shinohara S, Takebayashi S, Maetani T, Harada H, Kitani Y, Kumabe Y, Tsujimura T, Honda K, Ichimaru K, Ushiro K, Omori K. A multi-institutional retrospective study of 340 cases of sinonasal malignant tumor. Auris Nasus Larynx. 2024 Feb;51(1):86-98. doi: 10.1016/j.anl.2023.05.002. Epub 2023 May 27. PMID: 37248104. | 9 |
| 1. Mandava H, Venkata Renuka I, Ramamoorthy S. Unraveling the Clinicopathological Diversity and Subtypes of Rhabdomyosarcoma: A Study From a Tertiary Care Center. Cureus. 2024 Feb 17;16(2):e54341. doi: 10.7759/cureus.54341. PMID: 38500901; PMCID: PMC10945286. | 9 |
| 1. Marcoval J, Moreno-Vílchez C, Torrecilla-Vall-Llosera C, Muntaner-Virgili C, Pérez Sidelnikova D, Sanjuán X, Penín RM. Dermatofibrosarcoma Protuberans: A Study of 148 Patients. Dermatology. 2024;240(3):487-493. doi: 10.1159/000536172. Epub 2024 Jan 16. PMID: 38228098; PMCID: PMC11168446. | 2 |
| 1. Sahoo AK, Nair RR, Mishra UP, Behera G, Sidam S, Gupta V. Malignancy of Nose and Paranasal Sinuses: An Institutional Study in Central India. Cureus. 2024 Jan 11;16(1):e52074. doi: 10.7759/cureus.52074. PMID: 38344535; PMCID: PMC10858372. | 9 |
| 1. Muto Y, Fujimura T, Takahashi A, Namikawa K, Ogata D, Nakano E, Jinnai S, Hashimoto A, Kambayashi Y, Asano Y, Yamazaki N. Analysis of surgical margins and prognostic factors in dermatofibrosarcoma protuberans after wide local excision: A multicenter study of 116 Japanese patients. J Dermatol. 2024 Sep;51(9):1225-1232. doi: 10.1111/1346-8138.17280. Epub 2024 May 22. PMID: 38775205. | 2 |
| 1. Holm CE, Ørholt M, Talman ML, Abebe K, Thorn A, Baad-Hansen T, Petersen MM. A Population-Based Long-Term Follow-Up of Soft Tissue Angiosarcomas: Characteristics, Treatment Outcomes, and Prognostic Factors. Cancers (Basel). 2024 May 11;16(10):1834. doi: 10.3390/cancers16101834. PMID: 38791913; PMCID: PMC11120488. | 2 |
| 1. SILVA, E. S. E., COUTO, M. F. N., OLIVEIRA, G. L., DE LIMA, L. U. N. A., ALVES, C., SILVA, M. T. B., ... & GUIMARÃES, D. (2024). Prevalence of Paediatric Head and Neck Malignancies in the North Region of Brazil: A Cross-sectional Study. *Journal of Clinical & Diagnostic Research*, *18*(6). | 2 |
| 1. Linhares, L. D. (2024). Estudo multicêntrico das neoplasias malignas de boca em pacientes pediátricos e adolescentes. | 7 |
| 1. Jain E, Munjal G, Sharma S, Brar Z, Bhardwaj N, Dewan A, Jain D, Jha S, Lobo A, Malik V, Arora S, Varshney J, Beg A, Sampat NY, Parwani AV, Balzer B, Varma M, Yadav BS, Sharma SK, Singh HP, Gogoi K, Kumar D, Bhandari V, Fulara LM, Kumar A, Singh H, Bhattacharya M, Dixit M, Mohanty SK. Multifaceted Spindle Cell/Sclerosing Rhabdomyosarcoma With Role of Immunohistochemistry in Avoiding Misdiagnosis: A Multi-Institutional Study of 45 Distinct Tumors. Int J Surg Pathol. 2024 May;32(3):496-506. doi: 10.1177/10668969231188422. Epub 2023 Jul 25. PMID: 37489265. | 2 |
| 1. Sutow, W. W., Sullivan, M. P., Ried, H. L., Taylor, H. G., & Griffith, K. M. (1970). Prognosis in childhood rhabdomyosarcoma. *Cancer*, *25*(6), 1384-1390. | 2 |
| 1. Ma C, Ow A, Shan OH, Wu Y, Zhang C, Sun J, Ji T, Pingarron Martin L, Wang L. Malignant peripheral nerve sheath tumours in the head and neck region: retrospective analysis of clinicopathological features and treatment outcomes. Int J Oral Maxillofac Surg. 2014 Aug;43(8):924-32. doi: 10.1016/j.ijom.2014.03.006. Epub 2014 Mar 28. PMID: 24685259. | 2 |
| 1. Peng KA, Grogan T, Wang MB. Head and neck sarcomas: analysis of the SEER database. Otolaryngol Head Neck Surg. 2014 Oct;151(4):627-33. doi: 10.1177/0194599814545747. Epub 2014 Aug 18. PMID: 25135525; PMCID: PMC4598178. | 6 |
| 1. Chang AE, Chai X, Pollack SM, Loggers E, Rodler E, Dillon J, Parvathaneni U, Moe KS, Futran N, Jones RL. Analysis of clinical prognostic factors for adult patients with head and neck sarcomas. Otolaryngol Head Neck Surg. 2014 Dec;151(6):976-83. doi: 10.1177/0194599814551539. Epub 2014 Sep 25. PMID: 25257906. | 6 |
| 1. Modesto A, Filleron T, Chevreau C, Le Péchoux C, Rochaix P, Le Guellec S, Ducassou A, Gangloff D, Ferron G, Delannes M. Place de la radiothérapie dans le traitement conservateur des sarcomes en territoire irradié [Radiotherapy as conservative therapy for sarcomas within the irradiated field]. Cancer Radiother. 2014 Jun;18(3):171-6. French. doi: 10.1016/j.canrad.2014.02.006. Epub 2014 Apr 18. PMID: 24746453. | 2 |
| 1. Modesto A, Filleron T, Chevreau C, Le Pechoux C, Rochaix P, Le Guellec S, Ducassou A, Gangloff D, Ferron G, Delannes M. Role of radiation therapy in the conservative management of sarcoma within an irradiated field. Eur J Surg Oncol. 2014 Feb;40(2):187-92. doi: 10.1016/j.ejso.2013.07.088. Epub 2013 Sep 26. PMID: 24074728. | 2 |
| 1. Yun X, Wang P, Yu Y, Gao M. [Angiosarcoma of the head and face: a retrospective study of 15 patients]. Zhonghua Er Bi Yan Hou Tou Jing Wai Ke Za Zhi. 2014 Feb;49(2):136-40. Chinese. PMID: 24742513. | 7 |
| 1. Tajudeen BA, Fuller J, Lai C, Grogan T, Elashoff D, Abemayor E, St John M. Head and neck sarcomas: the UCLA experience. Am J Otolaryngol. 2014 Jul-Aug;35(4):476-81. doi: 10.1016/j.amjoto.2014.02.003. Epub 2014 Feb 15. PMID: 24721744; PMCID: PMC4230788. | 5 |
| 1. Oualla, K., Mellas, N., El’mrabet, F., & Ari, S. (2014). Adult head and neck sarcomas: rare localization with difficult therapeutic management. J Cancer Sci Ther, 6, 052-055. | 9 |
| 1. Federico SM, Gilpin D, Samant S, Billups CA, Spunt SL. Clinical features and outcomes of young patients with head and neck non-rhabdomyosarcoma soft tissue sarcomas. Head Neck. 2015 Jan;37(1):76-83. doi: 10.1002/hed.23564. Epub 2014 Feb 12. PMID: 24327514. | 6 |
| 1. Lindford A, McIntyre B, Marsh R, MacKinnon CA, Davis C, Tan ST. Outcomes of the treatment of head and neck sarcomas in a tertiary referral center. Front Surg. 2015 May 19;2:19. doi: 10.3389/fsurg.2015.00019. PMID: 26042220; PMCID: PMC4436802. | 6 |
| 1. Larizadeh MH, Damghani MA, Shabani M. Epidemiological characteristics of head and neck cancers in southeast of iran. Iran J Cancer Prev. 2014 Spring;7(2):80-6. PMID: 25250154; PMCID: PMC4142945. | 2 |
| 1. Frezza AM, Cesari M, Baumhoer D, Biau D, Bielack S, Campanacci DA, Casanova J, Esler C, Ferrari S, Funovics PT, Gerrand C, Grimer R, Gronchi A, Haffner N, Hecker-Nolting S, Höller S, Jeys L, Jutte P, Leithner A, San-Julian M, Thorkildsen J, Vincenzi B, Windhager R, Whelan J. Mesenchymal chondrosarcoma: prognostic factors and outcome in 113 patients. A European Musculoskeletal Oncology Society study. Eur J Cancer. 2015 Feb;51(3):374-81. doi: 10.1016/j.ejca.2014.11.007. Epub 2014 Dec 16. PMID: 25529371. | 2 |
| 1. Millare GG, Guha-Thakurta N, Sturgis EM, El-Naggar AK, Debnam JM. Imaging findings of head and neck dermatofibrosarcoma protuberans. AJNR Am J Neuroradiol. 2014 Feb;35(2):373-8. doi: 10.3174/ajnr.A3650. Epub 2013 Aug 1. PMID: 23907249; PMCID: PMC7965745. | 2 |
| 1. Marrugo-Pardo, G. E., Restrepo-Ángel, F., Cabrera-Bernal, E. V., & Sierra-Ávila, A. D. P. (2013). Ear rhabdomyosarcoma: Fundacion Hospital de la Misericordia Experience. *Revista de la Facultad de Medicina*, *61*(1), 71-75. | 9 |
| 1. Yu, D. X., Pi, S. J., & Zhang, W. S. (2013). Clinical manifestation of Kaposi sarcoma in otorhinolaryngology head and neck surgery. *Zhonghua er bi yan hou tou Jing wai ke za zhi= Chinese Journal of Otorhinolaryngology Head and Neck Surgery*, *48*(3), 241-243. | 7 |
|  |  |
| 1. Chung J, Lee V, Tsang R, Chan J, Kwong DL, Lam KO, Sze HC, Leung TW. Treatment outcomes of postradiation second head and neck malignancies managed by a multidisciplinary approach. Head Neck. 2015 Jun;37(6):815-22. doi: 10.1002/hed.23674. Epub 2014 Jun 19. PMID: 24623597. | 2 |
| 1. Andrä C, Rauch J, Li M, Ganswindt U, Belka C, Saleh-Ebrahimi L, Ballhausen H, Nachbichler SB, Roeder F. Excellent local control and survival after postoperative or definitive radiation therapy for sarcomas of the head and neck. Radiat Oncol. 2015 Jul 10;10:140. doi: 10.1186/s13014-015-0449-x. PMID: 26156022; PMCID: PMC4496934. | 2 |
| 1. Alishahi B, Kargahi N, Homayouni S. Epidemiological Evaluation of Head and Neck Sarcomas in Iran (the Study of 105 Cases Over 13 Years). Iran J Cancer Prev. 2015 Aug;8(4):e3432. doi: 10.17795/ijcp-3432. Epub 2015 Aug 24. PMID: 26478791; PMCID: PMC4606377. | 2 |
| 1. Reilly BK, Kim A, Peña MT, Dong TA, Rossi C, Murnick JG, Choi SS. Rhabdomyosarcoma of the head and neck in children: review and update. Int J Pediatr Otorhinolaryngol. 2015 Sep;79(9):1477-83. doi: 10.1016/j.ijporl.2015.06.032. Epub 2015 Jul 3. PMID: 26231745. | 6 |
| 1. Gounder M, Desai V, Kuk D, Agaram N, Arcila M, Durham B, Keohan ML, Dickson MA, D'Angelo SP, Shukla N, Moskowitz C, Noy A, Maki RG, Herrera DA, Sanchez A, Krishnan A, Pourmoussa A, Qin LX, Tap WD. Impact of surgery, radiation and systemic therapy on the outcomes of patients with dendritic cell and histiocytic sarcomas. Eur J Cancer. 2015 Nov;51(16):2413-22. doi: 10.1016/j.ejca.2015.06.109. Epub 2015 Aug 19. PMID: 26298731; PMCID: PMC5087129. | 2 |
| 1. Poursadegh M, Poursadegh F, Esmaeili M, Bakhshaee M. Epidemiological Survey of Sinonasal Malignancy in North-East Iran. Iran J Otorhinolaryngol. 2015 May;27(80):225-9. PMID: 26082905; PMCID: PMC4461847. | 2 |
| 1. Khalid M. Alwunais, Sohail Ahmad. Pattern of skin cancer at Dammam Medical Complex in Dammam, Saudi Arabia. Journal of Dermatology & Dermatologic Surgery. Volume 20, Issue 1. 2016. Pages 51-54, | 2 |
| 1. Kim et al. Radiation-Induced Sarcoma: A 15-Year Experience in a Single Large Tertiary Referral Center. Cancer Res Treat. 2016;48 (2): 650-657.Publication Date (Web): 2015 September 09 (Original Article). doi:https://doi.org/10.4143/crt.2015.171 | 2 |
| 1. Chao et al. Combined chemotherapy and125I-particle implantation for treatment of children with head and neck soft tissue sarcomas improve the short-term efficacy: Beijing children’s hospital experience over 2 years. International Journal of Clinical and Experimental MedicineVolume 9, Issue 6, Pages 11901 - 1190630 June 2016 | 6 |
| 1. Grevener K, Haveman LM, Ranft A, van den Berg H, Jung S, Ladenstein R, Klco-Brosius S, Juergens H, Merks JH, Dirksen U. Management and Outcome of Ewing Sarcoma of the Head and Neck. Pediatr Blood Cancer. 2016 Apr;63(4):604-10. doi: 10.1002/pbc.25830. Epub 2015 Dec 24. PMID: 26702872. | 6 |
| 1. Tan YG, Chia CS, Loh WL, Teo MC. Single-institution review of managing dermatofibrosarcoma protuberans. ANZ J Surg. 2016 May;86(5):372-6. doi: 10.1111/ans.13276. Epub 2015 Sep 2. PMID: 26334110. | 2 |
| 1. Chen Y, Shen Q, Gokavarapu S, Lin C, Yahiya, Cao W, Chauhan S, Liu Z, Ji T, Tian Z. Osteosarcoma of head and neck: A retrospective study on prognostic factors from a single institute database. Oral Oncol. 2016 Jul;58:1-7. doi: 10.1016/j.oraloncology.2016.04.008. Epub 2016 Apr 30. PMID: 27311395. | 6 |
| 1. Carlson ML, O'Connell BP, Breen JT, Wick CC, Driscoll CL, Haynes DS, Thompson RC, Isaacson B, Gidley PW, Kutz JW Jr, Van Gompel JJ, Wanna GB, Raza SM, DeMonte F, Barnett SL, Link MJ. Petroclival Chondrosarcoma: A Multicenter Review of 55 Cases and New Staging System. Otol Neurotol. 2016 Aug;37(7):940-50. doi: 10.1097/MAO.0000000000001037. PMID: 27273403. | 6 |
| 1. Carlson ML, O'Connell BP, Breen JT, Wick CC, Driscoll CL, Haynes DS, Thompson RC, Isaacson B, Gidley PW, Kutz JW Jr, Van Gompel JJ, Wanna GB, Raza SM, DeMonte F, Barnett SL, Link MJ. Petroclival Chondrosarcoma: A Multicenter Review of 55 Cases and New Staging System. Otol Neurotol. 2016 Aug;37(7):940-50. doi: 10.1097/MAO.0000000000001037. PMID: 27273403. | 6 |
| 1. Radzikowska J, Kukwa W, Kukwa A, Czarnecka AM, Kawecki M, Lian F, Szczylik C, Krzeski A. Management of pediatric head and neck rhabdomyosarcoma: A case-series of 36 patients. Oncol Lett. 2016 Nov;12(5):3555-3562. doi: 10.3892/ol.2016.5072. Epub 2016 Sep 1. PMID: 27900036; PMCID: PMC5104052. | 6 |
| 1. Vern-Gross TZ, Indelicato DJ, Bradley JA, Rotondo RL. Patterns of Failure in Pediatric Rhabdomyosarcoma After Proton Therapy. Int J Radiat Oncol Biol Phys. 2016 Dec 1;96(5):1070-1077. doi: 10.1016/j.ijrobp.2016.08.028. Epub 2016 Aug 30. PMID: 27742542. | 2 |
| 1. Yang Q, Mo Y, Zhao Q, Ban X, He M, Cai P, Liu X, Xie C, Zhang R. Radiation-induced sarcomas of the head and neck in post-radiation nasopharyngeal carcinoma. Radiol Med. 2017 Jan;122(1):53-60. doi: 10.1007/s11547-016-0695-5. Epub 2016 Oct 13. PMID: 27738788; PMCID: PMC5219029. | 2 |
| 1. Vitzthum L.K. et al. Head and neck soft tissue sarcomas treated with radiation therapy. Rare Tumors 2016 8:2 (60-65) Article Number 6165 | 2 |
| 1. Savar A, Trent J, Al-Zubidi N, Huh W, Anderson P, Shinder R, Esmaeli B. Efficacy of adjuvant and neoadjuvant therapies for adult orbital sarcomas. Ophthalmic Plast Reconstr Surg. 2010 May-Jun;26(3):185-9. doi: 10.1097/IOP.0b013e3181ba75e6. PMID: 20489544. | 2 |
| 1. Zevallos JP, Jain K, Roberts D, Santillan AA, Huh W, Hanna EY, Kupferman ME. Modern multimodality therapy for pediatric nonorbital parameningeal sarcomas. Head Neck. 2010 Nov;32(11):1501-5. doi: 10.1002/hed.21353. PMID: 20175111. | 2 |
| 1. Pacheco IA, Alves AP, Mota MR, Almeida PC, Holanda ME, Souza EF, Sousa FB. Clinicopathological study of patients with head and neck sarcomas. Braz J Otorhinolaryngol. 2011 Jun;77(3):385-90. doi: 10.1590/s1808-86942011000300019. PMID: 21739016; PMCID: PMC9443710. | 2 |
| 1. Clark DW, Moore BA, Patel SR, Guadagnolo BA, Roberts DB, Sturgis EM. Malignant fibrous histiocytoma of the head and neck region. Head Neck. 2011 Mar;33(3):303-8. doi: 10.1002/hed.21449. PMID: 20629084. | 2 |
| 1. Bakst R, Wolden S, Yahalom J. Radiation therapy for chloroma (granulocytic sarcoma). Int J Radiat Oncol Biol Phys. 2012 Apr 1;82(5):1816-22. doi: 10.1016/j.ijrobp.2011.02.057. Epub 2011 Sep 28. PMID: 21962486; PMCID: PMC5045241. | 2 |
| 1. Tejani, M. A., Galloway, T. J., Lango, M., Ridge, J. A., & Von Mehren, M. (2013). Head and neck sarcomas: a comprehensive cancer center experience. *Cancers*, *5*(3), 890-900. | 2 |
| 1. Stavrakas M, Nixon I, Andi K, Oakley R, Jeannon JP, Lyons A, McGurk M, Urbano TG, Thavaraj S, Simo R. Head and neck sarcomas: clinical and histopathological presentation, treatment modalities, and outcomes. J Laryngol Otol. 2016 Sep;130(9):850-9. doi: 10.1017/S0022215116008604. Epub 2016 Aug 1. PMID: 27476336. | 2 |
| 1. Boon E, van der Graaf WT, Gelderblom H, Tesselaar ME, van Es RJ, Oosting SF, de Bree R, van Meerten E, Hoeben A, Smeele LE, Willems SM, Witjes MJ, Buter J, Baatenburg de Jong RJ, Flucke UE, Peer PG, Bovée JV, Van Herpen CM. Impact of chemotherapy on the outcome of osteosarcoma of the head and neck in adults. Head Neck. 2017 Jan;39(1):140-146. doi: 10.1002/hed.24556. Epub 2016 Aug 10. PMID: 27507299; PMCID: PMC5215442. | 6 |
| 1. Vassiliou LV, Lalabekyan B, Jay A, Liew C, Whelan J, Newman L, Kalavrezos N. Head and neck sarcomas: A single institute series. Oral Oncol. 2017 Feb;65:16-22. doi: 10.1016/j.oraloncology.2016.12.005. Epub 2016 Dec 18. PMID: 28109463. | 6 |
| 1. Ellis MA, Gerry DR, Neskey DM, Lentsch EJ. Ewing Sarcoma of the Head and Neck. Ann Otol Rhinol Laryngol. 2017 Mar;126(3):179-184. doi: 10.1177/0003489416681322. Epub 2017 Jan 5. PMID: 28056517; PMCID: PMC6477678. | 6 |
| 1. Mahmoud O, Beck R, Kalyoussef E, Chan Park R, Baredes S, Kim S, Samuels MA. Adjuvant therapies utilization pattern and survival outcomes in high-grade head and neck soft tissue sarcoma; a population based study. Oral Oncol. 2017 Mar;66:28-37. doi: 10.1016/j.oraloncology.2016.12.030. Epub 2017 Jan 6. PMID: 28249645. | 6 |
| 1. Owosho AA, Estilo CL, Rosen EB, Yom SK, Huryn JM, Antonescu CR. A clinicopathologic study on SS18 fusion positive head and neck synovial sarcomas. Oral Oncol. 2017 Mar;66:46-51. doi: 10.1016/j.oraloncology.2016.12.021. Epub 2017 Jan 10. PMID: 28249647; PMCID: PMC5640264. | 6 |
| 1. Lee et al. 2017. Mesenchymal neoplasms of the head and neck: a cytopathologic analysis on fine needle aspiration. Journal of the American Society of Cytopathology. Volume 6, Issue 3, May–June 2017, Pages 105-113 | 6 |
| 1. Rutkowski P, Klimczak A, Ługowska I, Jagielska B, Wągrodzki M, Dębiec-Rychter M, Pieńkowska-Grela B, Świtaj T. Long-term results of treatment of advanced dermatofibrosarcoma protuberans (DFSP) with imatinib mesylate - The impact of fibrosarcomatous transformation. Eur J Surg Oncol. 2017 Jun;43(6):1134-1141. doi: 10.1016/j.ejso.2017.03.011. Epub 2017 Mar 22. PMID: 28365129. | 6 |
| 1. Lee RJ, Lee KK, Lin T, Arshi A, Lee SA, Christensen RE. Rhabdomyosarcoma of the head and neck: impact of demographic and clinicopathologic factors on survival. Oral Surg Oral Med Oral Pathol Oral Radiol. 2017 Sep;124(3):271-279. doi: 10.1016/j.oooo.2017.05.507. Epub 2017 May 30. PMID: 28732698. | 6 |
| 1. Chen Y, Gokavarapu S, Shen Q, Liu F, Cao W, Ling Y, Ji T. Chemotherapy in head and neck osteosarcoma: Adjuvant chemotherapy improves overall survival. Oral Oncol. 2017 Oct;73:124-131. doi: 10.1016/j.oraloncology.2017.08.017. Epub 2017 Sep 1. PMID: 28939064. | 6 |
| 1. Wang L, Lao IW, Yu L, Wang J. Clinicopathological features and prognostic factors in angiosarcoma: A retrospective analysis of 200 patients from a single Chinese medical institute. Oncol Lett. 2017 Nov;14(5):5370-5378. doi: 10.3892/ol.2017.6892. Epub 2017 Sep 6. PMID: 29113171; PMCID: PMC5656021. | 2 |
| 1. de Arruda JAA, Silva LVO, Kato CNAO, Schuch LF, Batista AC, Costa NL, Tarquinio SBC, Rivero ERC, Carrard VC, Martins MD, Sobral APV, Mesquita RA. A multicenter study of malignant oral and maxillofacial lesions in children and adolescents. Oral Oncol. 2017 Dec;75:39-45. doi: 10.1016/j.oraloncology.2017.10.016. Epub 2017 Nov 5. PMID: 29224821. | 2 |
| 1. Ludmir EB, Paulino AC, Grosshans DR, McAleer MF, McGovern SL, Huh WW, Okcu MF, Harrell LM, Mahajan A. Regional Nodal Control for Head and Neck Alveolar Rhabdomyosarcoma. Int J Radiat Oncol Biol Phys. 2018 May 1;101(1):169-176. doi: 10.1016/j.ijrobp.2018.01.052. Epub 2018 Jan 31. PMID: 29477293. | 6 |
| 1. Stojadinovic A, Karpoff HM, Antonescu CR, Shah JP, Singh B, Spiro RH, Dumornay W, Shaha AR. Dermatofibrosarcoma protuberans of the head and neck. Ann Surg Oncol. 2000 Oct;7(9):696-704. doi: 10.1007/s10434-000-0696-3. PMID: 11034249. | 2 |
| 1. Nagler RM, Malkin L, Ben-Arieh Y, Laufer D. Sarcoma of the maxillofacial region: follow-up of 25 cases. Anticancer Res. 2000 Sep-Oct;20(5C):3735-41. PMID: 11268447. | 7 |
| 1. Guo L, Zhao H, Chi F, Yan Y. [Clinical analysis of 17 cases rhabdomyosarcoma in ear nose and throat]. Lin Chuang Er Bi Yan Hou Ke Za Zhi. 2001 Aug;15(8):358-9. Chinese. PMID: 12541903. | 7 |
| 1. Pappo AS, Meza JL, Donaldson SS, Wharam MD, Wiener ES, Qualman SJ, Maurer HM, Crist WM. Treatment of localized nonorbital, nonparameningeal head and neck rhabdomyosarcoma: lessons learned from intergroup rhabdomyosarcoma studies III and IV. J Clin Oncol. 2003 Feb 15;21(4):638-45. doi: 10.1200/JCO.2003.01.032. PMID: 12586800. | 6 |
| 1. Bhurgri Y, Bhurgri A, Puri R, Ashraf S, Qidwai A, Ashraf K, Ahmed N, Mazhar A, Bhurgri H, Usman A, Faridi N, Malik J, Ahmed R, Muzaffar S, Kayani N, Pervez S, Hasan SH. Rhabdomyosarcoma in Karachi 1998-2002. Asian Pac J Cancer Prev. 2004 Jul-Sep;5(3):284-90. PMID: 15373708. | 2 |
| 1. Chindia ML, Swaleh SM, Godiah PM. Sarcomas of the head and neck at Kenyatta National Hospital. East Afr Med J. 2000 May;77(5):256-9. doi: 10.4314/eamj.v77i5.46629. PMID: 12858916. | 2 |
| 1. Morgan MB, Swann M, Somach S, Eng W, Smoller B. Cutaneous angiosarcoma: a case series with prognostic correlation. J Am Acad Dermatol. 2004 Jun;50(6):867-74. doi: 10.1016/j.jaad.2003.10.671. PMID: 15153886. | 2 |
| 1. Stojadinovic, A., Karpoff, H.M., Antonescu, C.R. *et al.* Dermatofibrosarcoma Protuberans of the Head and Neck. *Ann Surg Oncol* 7, 696–704 (2000). https://doi.org/10.1007/s10434-000-0696-3 | 2 |
| 1. He LJ, Wang L, Sun N, Huang CR, Zhu XD, Lang ZQ. [Clinicopathological study of 145 childhood rhabdomyosarcoma cases]. Zhonghua Bing Li Xue Za Zhi. 2004 Jun;33(3):225-8. Chinese. PMID: 15256113. | 2 |
| 1. Snow SN, Gordon EM, Larson PO, Bagheri MM, Bentz ML, Sable DB. Dermatofibrosarcoma protuberans: a report on 29 patients treated by Mohs micrographic surgery with long-term follow-up and review of the literature. Cancer. 2004 Jul 1;101(1):28-38. doi: 10.1002/cncr.20316. PMID: 15221986. | 2 |
| 1. Buwalda J, Blank LE, Schouwenburg PF, Copper MP, Strackee SD, Voûte PA, Merks JH, Caron HN. The AMORE protocol as salvage treatment for non-orbital head and neck rhabdomyosarcoma in children. Eur J Surg Oncol. 2004 Oct;30(8):884-92. doi: 10.1016/j.ejso.2004.06.005. PMID: 15336736. | 9 |
| 1. Xue H, Horwitz JR, Smith MB, Lally KP, Black CT, Cangir A, Takahashi H, Andrassy RJ. Malignant solid tumors in neonates: a 40-year review. J Pediatr Surg. 1995 Apr;30(4):543-5. doi: 10.1016/0022-3468(95)90126-4. PMID: 7595829. | 9 |
| 1. Dillon PW, Whalen TV, Azizkhan RG, Haase GM, Coran AG, King DR, Smith M. Neonatal soft tissue sarcomas: the influence of pathology on treatment and survival. Children's Cancer Group Surgical Committee. J Pediatr Surg. 1995 Jul;30(7):1038-41. doi: 10.1016/0022-3468(95)90337-2. PMID: 7472928. | 2 |
| 1. Pan M, Merchant M. Risk Factors Including Age, Stage and Anatomic Location that Impact the Outcomes of Patients with Synovial Sarcoma. Med Sci (Basel). 2018 Mar 6;6(1):21. doi: 10.3390/medsci6010021. PMID: 29509716; PMCID: PMC5872178. | 2 |
| 1. Schwager, K., & Klinker, H. (1994). Häufigkeit von HIV-assoziierten Erkrankungen im Kopf-Hals-Bereich. *Oto-Rhino-Laryngologia Nova*, *4*(4), 194-198. | 9 |
| 1. Penn I. De novo malignancy in pediatric organ transplant recipients. J Pediatr Surg. 1994 Feb;29(2):221-6; discussion 227-8. doi: 10.1016/0022-3468(94)90322-0. PMID: 8176596. | 2 |
| 1. LeVay J, O'Sullivan B, Catton C, Bell R, Fornasier V, Cummings B, Hao Y, Warr D, Quirt I. Outcome and prognostic factors in soft tissue sarcoma in the adult. Int J Radiat Oncol Biol Phys. 1993 Dec 1;27(5):1091-9. doi: 10.1016/0360-3016(93)90529-5. PMID: 8262833. | 2 |
| 1. Nakada, K. The clinical features and prognosis of rhabdomyosarcoma: Follow-up studies on pediatric tumors from the Japanese pediatric tumor registry 1971–1980. Part II. *The Japanese Journal of Surgery* 20, 503–509 (1990). https://doi.org/10.1007/BF02471005 | 2 |
| 1. Jaffe BF. Pediatric head and neck tumors: a study of 178 cases. Laryngoscope. 1973 Oct;83(10):1644-51. doi: 10.1288/00005537-197310000-00006. PMID: 4800746. | 2 |
| 1. Otoh EC, Johnson NW, Danfillo IS, Adeleke OA, Olasoji HA. Primary head and neck cancers in North Eastern Nigeria. West Afr J Med. 2004 Oct-Dec;23(4):305-13. doi: 10.4314/wajm.v23i4.28146. PMID: 15730089. | 2 |
| 1. Kuhnen C, Lehnhardt M, Steinau HU, Müller KM. Liposarkome. Aspekte zur Pathomorphologie--eine Analyse von 209 Tumoren [liposarcoma. Aspects of pathomorphology--an analysis of 209 tumos]. Chirurg. 2004 Dec;75(12):1151-8. German. doi: 10.1007/s00104-004-0901-1. PMID: 15248051. | 2 |
| 1. Chiles MC, Parham DM, Qualman SJ, Teot LA, Bridge JA, Ullrich F, Barr FG, Meyer WH; Soft Tissue Sarcoma Committee of the Children's Oncology Group. Sclerosing rhabdomyosarcomas in children and adolescents: a clinicopathologic review of 13 cases from the Intergroup Rhabdomyosarcoma Study Group and Children's Oncology Group. Pediatr Dev Pathol. 2004 Nov-Dec;7(6):583-94. doi: 10.1007/s10024-004-5058-x. Epub 2004 Nov 17. Erratum in: Pediatr Dev Pathol. 2005 Jan-Feb;8(1):141. PMID: 15630526. | 9 |
| 1. Chen SA, Morris CG, Amdur RJ, Werning JW, Villaret DB, Mendenhall WM. Adult head and neck soft tissue sarcomas. Am J Clin Oncol. 2005 Jun;28(3):259-63. doi: 10.1097/01.coc.0000158440.27229.d6. PMID: 15923798. | 2 |
| 1. Nascimento AF, Fletcher CD. Spindle cell rhabdomyosarcoma in adults. Am J Surg Pathol. 2005 Aug;29(8):1106-13. PMID: 16006807. | 7 |
| 1. Colville RJ, Charlton F, Kelly CG, Nicoll JJ, McLean NR. Multidisciplinary management of head and neck sarcomas. Head Neck. 2005 Sep;27(9):814-24. doi: 10.1002/hed.20232. PMID: 16086411. | 2 |
| 1. Meazza C, Ferrari A, Casanova M, Massimino M, Luksch R, Spreafico F, Cefalo G, Terenziani M, Polastri D, Podda M, Catania S, Cereda S, Gandola L, Fossati-Bellani F, Cantù G. Rhabdomyosarcoma of the head and neck region: experience at the pediatric unit of the Istituto Nazionale Tumori, Milan. J Otolaryngol. 2006 Feb;35(1):53-9. doi: 10.2310/7070.2005.4091. PMID: 16527019. | 2 |
| 1. Otoh EC, Johnson NW, Mandong BM, Danfillo IS. Primary head and neck cancers in Jos, Nigeria: a re-visit. West Afr J Med. 2006 Apr-Jun;25(2):92-100. doi: 10.4314/wajm.v25i2.28256. PMID: 16918178. | 2 |
| 1. Dave B, Shet T, Ramadwar M, Kane S. Cytological evaluation of head and neck tumors in children--a pattern analysis. Diagn Cytopathol. 2006 Jun;34(6):434-46. doi: 10.1002/dc.20441. PMID: 16680771. | 3 |
| 1. Sabesan T, Xuexi W, Yongfa Q, Pingzhang T, Ilankovan V. Malignant fibrous histiocytoma: outcome of tumours in the head and neck compared with those in the trunk and extremities. Br J Oral Maxillofac Surg. 2006 Jun;44(3):209-12. doi: 10.1016/j.bjoms.2005.06.006. Epub 2005 Jul 18. PMID: 16026908. | 2 |
| 1. Rodjan F, Graaf Pd, Brisse HJ, Verbeke JI, Sanchez E, Galluzzi P, Göricke S, Maeder P, Aerts I, Dendale R, Desjardins L, de Franscesco S, Bornfeld N, Sauerwein W, Popovic MB, Knol DL, Moll AC, Castelijns JA. Second cranio-facial malignancies in hereditary retinoblastoma survivors previously treated with radiation therapy: clinic and radiologic characteristics and survival outcomes. Eur J Cancer. 2013 May;49(8):1939-47. doi: 10.1016/j.ejca.2013.01.010. Epub 2013 Feb 14. PMID: 23415887. | 2 |
| 1. Brown BJ, Oluwasola AO. Childhood rhabdomyosarcoma in Ibadan, Nigeria: 1984-2003. Ann Trop Paediatr. 2006 Dec;26(4):349-55. doi: 10.1179/146532806X152881. PMID: 17132301. | 2 |
| 1. Sbeity S, Abella A, Arcand P, Quintal MC, Saliba I. Temporal bone rhabdomyosarcoma in children. Int J Pediatr Otorhinolaryngol. 2007 May;71(5):807-14. doi: 10.1016/j.ijporl.2007.02.002. Epub 2007 Mar 8. PMID: 17346806. | 9 |
| 1. Huber GF, Matthews TW, Dort JC. Radiation-induced soft tissue sarcomas of the head and neck. J Otolaryngol. 2007 Apr;36(2):93-7. doi: 10.2310/7070.2007.0001. PMID: 17459279. | 9 |
| 1. Combs SE, Behnisch W, Kulozik AE, Huber PE, Debus J, Schulz-Ertner D. Intensity Modulated Radiotherapy (IMRT) and Fractionated Stereotactic Radiotherapy (FSRT) for children with head-and-neck-rhabdomyosarcoma. BMC Cancer. 2007 Sep 13;7:177. doi: 10.1186/1471-2407-7-177. PMID: 17854490; PMCID: PMC2077337. | 2 |
| 1. Lee N, Shin E. Treatment outcomes for patients with synovial sarcoma of the head and neck. Expert Rev Anticancer Ther. 2008 Mar;8(3):371-3. doi: 10.1586/14737140.8.3.371. PMID: 18366285. | 4 |
| 1. DeMartelaere SL, Roberts D, Burgess MA, Morrison WH, Pisters PW, Sturgis EM, Ho V, Esmaeli B. Neoadjuvant chemotherapy-specific and overall treatment outcomes in patients with cutaneous angiosarcoma of the face with periorbital involvement. Head Neck. 2008 May;30(5):639-46. doi: 10.1002/hed.20757. PMID: 18213722. | 2 |
| 1. Lin JW, Wu GH, Zeng ZY, Chen WK, Guo ZM, Zhang Q, Li H. [Clinical features and diagnosis of head and neck rhabdomyosarcoma: a report of 24 cases]. Ai Zheng. 2008 Jun;27(6):618-21. Chinese. PMID: 18570736. | 7 |
| 1. Adeyemi BF, Adekunle LV, Kolude BM, Akang EE, Lawoyin JO. Head and neck cancer--a clinicopathological study in a tertiary care center. J Natl Med Assoc. 2008 Jun;100(6):690-7. doi: 10.1016/s0027-9684(15)31343-2. PMID: 18595571. | 2 |
| 1. Raney B, Anderson J, Breneman J, Donaldson SS, Huh W, Maurer H, Michalski J, Qualman S, Ullrich F, Wharam M, Meyer W; Soft-Tissue Sarcoma Committee of the Children's Oncology Group, Arcadia, California, USA. Results in patients with cranial parameningeal sarcoma and metastases (Stage 4) treated on Intergroup Rhabdomyosarcoma Study Group (IRSG) Protocols II-IV, 1978-1997: report from the Children's Oncology Group. Pediatr Blood Cancer. 2008 Jul;51(1):17-22. doi: 10.1002/pbc.21492. PMID: 18266224. | 2 |
| 1. Bahrami A, Gown AM, Baird GS, Hicks MJ, Folpe AL. Aberrant expression of epithelial and neuroendocrine markers in alveolar rhabdomyosarcoma: a potentially serious diagnostic pitfall. Mod Pathol. 2008 Jul;21(7):795-806. doi: 10.1038/modpathol.2008.86. Epub 2008 May 16. PMID: 18487991. | 2 |
| 1. Penel N, Grosjean J, Pichon-Watelle F, Giscard S, Hoppe H, Taieb S, Vanseymortier L, Adenis A. Factors favouring palliative treatment multidisciplinary decisions for newly diagnosed visceral and soft tissue sarcomas. Clin Oncol (R Coll Radiol). 2008 Sep;20(7):523-7. doi: 10.1016/j.clon.2008.03.014. Epub 2008 Apr 28. PMID: 18440792. | 2 |
| 1. Butt FM, Chindia ML, Rana F, Machigo FG. Pattern of head and neck malignant neoplasms in HIV-infected patients in Kenya. Int J Oral Maxillofac Surg. 2008 Oct;37(10):907-11. doi: 10.1016/j.ijom.2008.07.019. Epub 2008 Sep 9. PMID: 18783921. | 2 |
| 1. Khademi B, Taraghi A, Mohammadianpanah M. Anatomical and histopathological profile of head and neck neoplasms in Persian pediatric and adolescent population. Int J Pediatr Otorhinolaryngol. 2009 Sep;73(9):1249-53. doi: 10.1016/j.ijporl.2009.05.017. Epub 2009 Jun 13. PMID: 19525017. | 2 |
| 1. Samaila MO. Malignant tumours of childhood in Zaria. Afr J Paediatr Surg. 2009 Jan-Jun;6(1):19-23. doi: 10.4103/0189-6725.48570. PMID: 19661660. | 2 |
| 1. Matrka, L., Cronin, S., Agrawal, A., & Wakely, P. (2009). Head and neck rhabdomyosarcoma in the adult population. Laryngoscope, 119(SUPPL.3), S245. https://doi.org/10.1002/lary.21503 | 4 |
| 1. Lin X, Zeng S, Yang C, Li C, Yang D. [Clinical analysis on 28 cases of head-neck malignant fibrous histiocytoma]. Lin Chuang Er Bi Yan Hou Tou Jing Wai Ke Za Zhi. 2009 Oct;23(19):886-8, 893. Chinese. PMID: 20120875. | 7 |
| 1. Otoh EC, Johnson NW, Ajike SO, Mohammed A, Danfillo IS, Jallo PH. Primary head and neck cancers in North -Western Nigeria. West Afr J Med. 2009 Jul-Aug;28(4):227-33. PMID: 20425737. | 2 |
| 1. Sengupta S, Pal R. Clinicopathological correlates of pediatric head and neck cancer. J Cancer Res Ther. 2009 Jul-Sep;5(3):181-5. doi: 10.4103/0973-1482.57123. PMID: 19841559. | 2 |
| 1. Hong P, Taylor SM, Trites JR, Bullock M, Nasser JG, Hart RD. Chondrosarcoma of the head and neck: report of 11 cases and literature review. J Otolaryngol Head Neck Surg. 2009 Apr;38(2):279-85. PMID: 19442379. | 9 |
| 1. Person L, Lacour B, Faure L, Guissou S, Poulalhon C, Orbach D, Goujon S, Berger C, Clavel J, Desandes E. Childhood head and neck cancer in France: Incidence, survival and trends from 2000 to 2015. Int J Pediatr Otorhinolaryngol. 2021 Nov;150:110858. doi: 10.1016/j.ijporl.2021.110858. Epub 2021 Aug 3. PMID: 34388659. | 2 |
| 1. Tao YJ, Zhen HN, Guan H, Shen J, Zhang FQ, Liu ZK. [Parameningeal or non-parameningeal head and neck rhabdomyosarcoma: a study based on propensity score matching and survival analysis]. Zhonghua Er Bi Yan Hou Tou Jing Wai Ke Za Zhi. 2022 Dec 7;57(12):1409-1417. Chinese. doi: 10.3760/cma.j.cn115330-20220511-00261-1. PMID: 36707944. | 2 |
| 1. Eide JG, Kshirsagar RS, Harris JC, Civantos A, Brody RM, Lee JYK, Alonso-Basanta M, Lazor JW, Nabavizadeh A, Wang BY, Kuan EC, Palmer JN, Adappa ND. Multi-institutional review of sinonasal and skull base chondrosarcoma: 20-year experience. Head Neck. 2022 Dec;44(12):2686-2695. doi: 10.1002/hed.27178. Epub 2022 Sep 2. PMID: 36052545. | 2 |
| 1. Shi CJ, Xu SM, Li CW, Tian Z, Wang LZ, Hu YH, Xia RH, Zhang ZY, Li J. Epithelioid sarcoma: A clinicopathological study of 12 head and neck cases. Oral Dis. 2022 Sep;28(6):1519-1527. doi: 10.1111/odi.13835. Epub 2021 Apr 7. PMID: 33751732. | 2 |
| 1. Guo XC, Wang JL, Liu L, Sang JZ, Cao H. [Clinical analysis of 24 cases of synovial sarcoma of head and neck]. Zhonghua Er Bi Yan Hou Tou Jing Wai Ke Za Zhi. 2022 Jul 7;57(7):854-859. Chinese. doi: 10.3760/cma.j.cn115330-20210925-00631. PMID: 35866279. | 7 |
| 1. Irawati N, Moghadam A, Abdul-Razak M, Strach M, Elliott M, Ch'ng S, Shannon K, Palme CE, Clark J, Wykes J, Low TH. Outcomes after definitive treatment for head and neck angiosarcoma. ANZ J Surg. 2022 Jun;92(6):1407-1414. doi: 10.1111/ans.17695. Epub 2022 May 9. PMID: 35531880. | 2 |
| 1. Samina et al. Rhabdomyosarcoma in pediatric population; a 5 year experience at Children’s Hospital, Lahore. Pakistan Journal of Medical and Health SciencesVolume 12, Issue 3, Pages 1028 - 1030July-September 2018 | 2 |
| 1. Li W, Yang L, Li Q, Chen M, Zhang H. [Synovial sarcoma of the head and neck--a retrospective study of 39 cases]. Lin Chuang Er Bi Yan Hou Tou Jing Wai Ke Za Zhi. 2013 Nov;27(21):1167-70. Chinese. PMID: 24616993. | 7 |
| 1. Bouaoud J, Temam S, Galmiche L, Cozic N, Bolle S, Belhous K, Kolb F, Qassemyar Q, Bidault F, Couloigner V, Picard A, Le Deley MC, Mahier-Ait Oukhatar C, Gaspar N, Kadlub N. Head and neck Ewing sarcoma: French surgical practice analysis pleads for surgery centralization. J Craniomaxillofac Surg. 2022 May;50(5):439-448. doi: 10.1016/j.jcms.2021.01.030. Epub 2021 Feb 6. PMID: 35063337. | 2 |
| 1. Arboleda LP, Pérez-de-Oliveira ME, Hoffmann IL, Cardinalli IA, Gallagher KP, Santos-Silva AR, Mendonça RM. Clinical manifestations of head and neck cancer in pediatric patients, an analysis of 253 cases in a single Brazilian center. Med Oral Patol Oral Cir Bucal. 2022 May 1;27(3):e285-e293. doi: 10.4317/medoral.25255. PMID: 35368009; PMCID: PMC9054174. | 2 |
| 1. Smith JB, Cass LM, Simpson MC, Osazuwa-Peters N, Ward GM, Massa ST. Radiation-Associated Sarcoma of the Head and Neck: Incidence, Latency, and Survival. Laryngoscope. 2022 May;132(5):1034-1041. doi: 10.1002/lary.29942. Epub 2021 Nov 15. PMID: 34779517. | 2 |
| 1. Sargen MR, Cahoon EK, Yu KJ, Madeleine MM, Zeng Y, Rees JR, Lynch CF, Engels EA. Spectrum of Nonkeratinocyte Skin Cancer Risk Among Solid Organ Transplant Recipients in the US. JAMA Dermatol. 2022 Apr 1;158(4):414-425. doi: 10.1001/jamadermatol.2022.0036. PMID: 35262623; PMCID: PMC8908231. | 2 |
| 1. Rüller K, Sittel C, Kölmel JC, Burghartz M, Steimer J, Fiz F, Piazza C, Peretti G, Fiz I. Organ Preservation Strategies in Laryngeal Chondrosarcoma. Laryngoscope. 2022 Apr;132(4):838-843. doi: 10.1002/lary.29826. Epub 2021 Aug 21. PMID: 34418107. | 2 |
| 1. Zeitels SM, Baird BJ. Surgical Treatment Strategies for Laryngeal Chondrosarcomas: A Single Institution Investigation. Laryngoscope. 2022 Jan;132(1):169-176. doi: 10.1002/lary.29762. Epub 2021 Jul 22. PMID: 34291467. | 2 |
| 1. de Araújo GR, Costa SFDS, Mesquita RA, Gomez RS, Dos Santos JN, Pontes HAR, de Andrade BAB, Romañach MJ, Agostini M, Vargas PA, de Cáceres CVBL, Santos-Silva AR, Ribeiro ACP, Brandão TB, Tomasi RA, Ferreyra RS, de Almeida OP, Fonseca FP. Leiomyoma and Leiomyosarcoma (Primary and Metastatic) of the Oral and Maxillofacial Region: A Clinicopathological and Immunohistochemical Study of 27 Cases. Head Neck Pathol. 2022 Mar;16(1):294-303. doi: 10.1007/s12105-021-01336-2. Epub 2021 Jun 9. PMID: 34106410; PMCID: PMC9018928. | 9 |
| 1. Verbruggen C, Ricard AS, Cogrel O, Bondaz M, Carrier S. Marges d’exérèse des dermatofibrosarcomes cervico-faciaux par technique de Slow-Mohs : étude clinique rétrospective sur 20 cas [Dermatofibrosarcoma protuberans: Surgical margins using Slow-Mohs micrographic surgery. A clinical retrospective study about 20 cases]. Ann Chir Plast Esthet. 2018 Feb;63(1):47-53. French. doi: 10.1016/j.anplas.2017.06.005. Epub 2017 Jul 26. PMID: 28755830. | 2 |
| 1. Serra-Guillén C, Llombart B, Nagore E, Guillén C, Sanmartín O. Determination of Margins for Tumor Clearance in Dermatofibrosarcoma Protuberans: A Single-Center Study of 222 Cases Treated With Modified Mohs Surgery. Dermatol Surg. 2022 Jan 1;48(1):51-56. doi: 10.1097/DSS.0000000000003269. PMID: 34743125. | 7 |
| 1. Wakeman KM, Zhang QS, Bandhlish A, Cranmer LD, Ricciotti RW, Mantilla JG. Fédération Nationale Des Centres de Lutte Contre Le Cancer (FNCLCC) Grading, Margin Status and Tumor Location Associate With Survival Outcomes in Malignant Peripheral Nerve Sheath Tumors. Am J Clin Oncol. 2022 Jan 1;45(1):28-35. doi: 10.1097/COC.0000000000000877. PMID: 34962906. | 2 |
| 1. Al-Mowali AA, Hashim HS, Al-Haroon SS, Al-Abbasi AM, Al-Nakshabandi SA. Malignant Head and Neck Tumors in Basrah: A Clinicopathological Study. Biomed Pharmacol J. 2022;15(1). doi: 10.13005/bpj/2376. | 2 |
| 1. Tan Z, Liu J, Xue R, Fan Z, Bai C, Li S, Gao T, Zhang L, Wang X. Clinical features and therapeutic outcomes of alveolar soft part sarcoma in children: A single-center, retrospective study. Front Oncol. 2022 Nov 24;12:1019911. doi: 10.3389/fonc.2022.1019911. PMID: 36505873; PMCID: PMC9730233. | 2 |
| 1. Arosio AD, Turri-Zanoni M, Sileo G, Tirloni M, Volpi L, Lambertoni A, Margherini S, Mercuri A, Battaglia P, Cherubino M, Castelnuovo P, Bignami M. Maxillary Sinus Floor Infiltration: Results From a Series of 118 Maxillary Sinus Cancers. Laryngoscope. 2022 Jan;132(1):26-35. doi: 10.1002/lary.29697. Epub 2021 Jun 22. PMID: 34156096. | 2 |
| 1. Gebril MAB, Mukhtar WNO, Elhassan MMA, Mahmoud I. Incidence Characteristics and Histological Types of Head and Neck Cancer among Adults in Central Sudan: A Retrospective Study. Int J Environ Res Public Health. 2022 Oct 24;19(21):13814. doi: 10.3390/ijerph192113814. PMID: 36360694; PMCID: PMC9656960. | 2 |
| 1. Rodriguez KD, Schneider KW, Suttman A, Garrington T, Jellins T, Tholen K, Francom CR, Herrmann BW. Pediatric Head and Neck Tumors Associated with Li-Fraumeni Syndrome. Ann Otol Rhinol Laryngol. 2022 Feb;131(2):159-163. doi: 10.1177/00034894211014786. Epub 2021 May 10. PMID: 33971750. | 9 |
| 1. Daoud NA, Bhuiyan MAN, Koppada S, Alam M, Malek AE. 437. A Single-Center Experience of Solid versus Hematologic Malignancies in People Living with HIV. Open Forum Infect Dis. 2022 Dec 15;9(Suppl 2):ofac492.512. doi: 10.1093/ofid/ofac492.512. PMCID: PMC9752153. | 4 |
| 1. Alorjani MS, Matalka II, Alfaqih MA, Jahmani RA, Alsinglawi BS, Nimri FM, Matalka MI, Amr SS. Soft Tissue Sarcomas: A 16-Year Experience of a Tertiary Referral Hospital in North Jordan. Medicina (Kaunas). 2022 Jan 27;58(2):198. doi: 10.3390/medicina58020198. PMID: 35208522; PMCID: PMC8878199. | 2 |
| 1. El Shatanofy M, Thakkar P, Patel V, Joshi A, Goodman J, Siegel R, Haroun F, Ojong-Ntui M, Goyal S, Bauman J, Rao YJ. Intensified Adjuvant Treatment for High-Risk Resected Cutaneous Angiosarcoma of the Head and Neck. Otolaryngol Head Neck Surg. 2023 Nov;169(5):1225-1233. doi: 10.1002/ohn.429. Epub 2023 Jul 19. PMID: 37464928. | 2 |
| 1. Varghese BT, Nadarajan AR, Thomas S, Iype EM, George NA, K M JK, Lal SS, Somanathan T. Spectrum of malignant scalp tumours and its impact on management-a tertiary care cancer centre experience. World J Surg Oncol. 2023 Oct 17;21(1):330. doi: 10.1186/s12957-023-03200-9. PMID: 37845728; PMCID: PMC10580575. | 2 |
| 1. Ohno K, Tsunoda A, Shirakura S, Takahashi N, Kishimoto S. The approaches and outcomes of skull base surgery for pediatric sarcoma after initial therapy. Auris Nasus Larynx. 2011 Apr;38(2):208-14. doi: 10.1016/j.anl.2010.08.005. Epub 2010 Nov 4. PMID: 21055890. | 5 |
| 1. Mathew J, Arjunan R, Dasappa A, Namachivayam A. Prognostic Factors and Clinical Outcomes in Extraskeletal Ewing Sarcoma: A Cohort Study. Ann Surg Oncol. 2023 May;30(5):3084-3094. doi: 10.1245/s10434-022-12992-1. Epub 2022 Dec 23. PMID: 36564656. | 2 |
| 1. Cunha WA, Corazza AC, Rezende KM, Bönecker M, Gallottini M. Paediatric head and neck malignant neoplasms: A brazilian retrospective study. Med Oral Patol Oral Cir Bucal. 2023 Mar 1;28(2):e140-e147. doi: 10.4317/medoral.25614. PMID: 36641746; PMCID: PMC9985934. | 2 |
| 1. Suzuki H, Takano G, Tsukushi S, Ando M, Yatabe Y, Kodaira T, Nishikawa D, Beppu S, Hasegawa Y, Hanai N. Impact of age for overall survival in head and neck sarcoma. Medicine (Baltimore). 2023 Feb 17;102(7):e32966. doi: 10.1097/MD.0000000000032966. PMID: 36800630; PMCID: PMC9935989 | 2 |
| 1. Olamaeian F, Tayebi A, Pour Mohammad A, Gholizadeh Mesgarha M, Asef-Kabiri L, Seyedesfahani B, Akbari ME. Epidemiological Study of Salivary Sarcoma in Iran, 2009 - 2014. Int J Cancer Manag. 2022;15(1). doi: 10.5812/ijcm-118342. | 2 |
| 1. Fei-Zhang DJ, Park AC, Berry JM, Arch RS, Chelius DC, Sheyn AM, Rastatter JC. National assessment of lymph node status indicators & predictors in pediatric head and neck rhabdomyosarcomas in the US. Int J Pediatr Otorhinolaryngol. 2023 Jan;164:111419. doi: 10.1016/j.ijporl.2022.111419. Epub 2022 Dec 12. PMID: 36525697. | 2 |
| 1. Hofmann E, Preissner S, Hertel M, Preissner R, Rendenbach C, Flörcken A, Heiland M. A retrospective case-control study for the comparison of 5-year survival rates: the role of adjuvant and neoadjuvant chemotherapy in craniofacial bone sarcoma in adults. Ther Adv Med Oncol. 2023 Feb 15;15:17588359221148023. doi: 10.1177/17588359221148023. PMID: 36818689; PMCID: PMC9936400. | 2 |
| 1. Ma J, Gao J, Ming C, Ma X, Lou F, Li X, Zhou J, Yang H, Ruan B, Zhang T. [Clinical characteristics of 91 patients of otorhinolaryngology head and neck malignant tumors in children]. Lin Chuang Er Bi Yan Hou Tou Jing Wai Ke Za Zhi. 2023 Jan;37(1):36-41. Chinese. doi: 10.13201/j.issn.2096-7993.2023.01.007. PMID: 36597366; PMCID: PMC10128353. | 2 |
| 1. Di Ciaccio PR, Van Leeuwen MT, Amin J, Vajdic CM, McGregor S, Poynten IM, Templeton DJ, Law M, Grulich AE, Polizzotto MN, Jin F. Second Primary Cancers in People With HIV/AIDS: A National Data Linkage Study of Incidence and Risk Factors. J Acquir Immune Defic Syndr. 2023 May 1;93(1):25-33. doi: 10.1097/QAI.0000000000003172. PMID: 36804500; PMCID: PMC10484748. | 1 |
| 1. Hikmet RG, Klug TE, Gade SD, Sandfeld-Paulsen B, Aggerholm-Pedersen N. A Retrospective Study of 291 Patients With Head and Neck Sarcomas: Treatment, Outcomes, and Prognostic Factors. Clin Oncol (R Coll Radiol). 2024 Jul;36(7):409-419. doi: 10.1016/j.clon.2024.04.009. Epub 2024 Apr 27. PMID: 38744596. | 2 |
| 1. Xie S, Zhang J, Qin Y, Li M, Lan G, Wang Y, Huang B, Weng J, Wei J, Qu S. [Radiation-induced sarcoma in patients with nasopharyngeal carcinoma: a single-institution retrospective study]. Lin Chuang Er Bi Yan Hou Tou Jing Wai Ke Za Zhi. 2024 Jun;38(6):514-517. Chinese. doi: 10.13201/j.issn.2096-7993.2024.06.011. PMID: 38858117; PMCID: PMC11480581. | 2 |
| 1. Chen Y, Gokavarapu S, Abdelrehem A, Ji T. Head and Neck Soft Tissue Sarcoma Patient: Age and Tumor Region Are Predictive in Disease-Free Survival and Overall Survival. J Oral Maxillofac Surg. 2024 Jun;82(6):719-727. doi: 10.1016/j.joms.2024.02.015. Epub 2024 Mar 5. PMID: 38513711. | 2 |
| 1. Wang T, Wang J, Tang T, Li Q, Li Y, Song X. Surgery for adult head and neck rhabdomyosarcoma: A retrospective report from one institution. Int Forum Allergy Rhinol. 2024 Jun;14(6):1110-1114. doi: 10.1002/alr.23313. Epub 2023 Dec 28. PMID: 38153372. | 2 |
| 1. Li YH, Zheng L, Chen GC, Zhang YM, Xu YZ, Huang YF. Real-world impact of chemotherapy on overall survival in craniomaxillofacial osteosarcoma. J Stomatol Oral Maxillofac Surg. 2024 Jun;125(3S):101807. doi: 10.1016/j.jormas.2024.101807. Epub 2024 Feb 29. PMID: 38431082. | 2 |
| 1. Rikitake R, Mizushima Y, Yoshimoto S, Higashi T, Satake T, Morizane C, Kawai A. Current status of head and neck sarcomas in Japan in 2016-2019: an analysis using the national cancer registry. Int J Clin Oncol. 2024 May;29(5):564-570. doi: 10.1007/s10147-024-02484-5. Epub 2024 Feb 27. PMID: 38411881; PMCID: PMC11043187. | 2 |
| 1. Hofmann E, Preissner S, Hertel M, Preissner R, Rendenbach C, Flörcken A, Heiland M. A retrospective case-control study for the comparison of 5-year survival rates: the role of adjuvant and neoadjuvant chemotherapy in craniofacial bone sarcoma in adults. Ther Adv Med Oncol. 2023 Feb 15;15:17588359221148023. doi: 10.1177/17588359221148023. PMID: 36818689; PMCID: PMC9936400. | 2 |
| 1. Zhang G, Wang SC, Su Y, Liu ZK, Yu GX, Zhang J, Mei L, Sun N, Li YZ, Zhang XX, Liu QY, Liu ZY, Li XD, Ni X. [Retrospective study of 70 cases with the head and neck non-parameningeal rhabdomyosarcoma]. Zhonghua Er Bi Yan Hou Tou Jing Wai Ke Za Zhi. 2024 Feb 7;59(2):133-139. Chinese. doi: 10.3760/cma.j.cn115330-20230712-00002. PMID: 38369791. | 7 |
| 1. Gazda P, Baujat B, Sarini J, Gomez-Brouchet A, Philouze P, Moya-Plana A, Malard O, Fakhry N, De Mones Del Pujol E, Garrel R, Page C, Mouawad F, Vaz E, Evrard D, Bach C, Dufour X, Lelonge Y, Schultz P, Mauvais O, Brenet E, Vergez S, Atallah S. Functional or radical surgical treatment of laryngeal chondrosarcoma, analysis of survival and prognostic factors: A REFCOR and NetSarc-ResOs multicenter study of 74 cases. Eur J Surg Oncol. 2024 Feb;50(2):107315. doi: 10.1016/j.ejso.2023.107315. Epub 2023 Dec 22. PMID: 38219696. | 2 |
| 1. Bini A, Derka S, Stavrianos S. Management of head & neck sarcomas in adults: A retrospective study. J Craniomaxillofac Surg. 2024 Aug;52(8):877-883. doi: 10.1016/j.jcms.2024.01.005. Epub 2024 Jan 11. PMID: 38443189. | 1 |
| 1. Li HL, Mo CH, Xie L, Wu YX, Zeng M, Mao RJ. [Clinicopathological study of epithelioid and spindle cell rhabdomysarcoma with EWSR1/FUS-TFCP2 fusion]. Zhonghua Bing Li Xue Za Zhi. 2024 Jan 8;53(1):58-63. Chinese. doi: 10.3760/cma.j.cn112151-20230925-00214. PMID: 38178748. | 9 |
| 1. Raj R, Kim HG, Xu M, Roach T, Liebner D, Konieczkowski D, Tinoco G. Clinical Characteristics, Patterns of Care, and Treatment Outcomes of Radiation-Associated Sarcomas. Cancers (Basel). 2024 May 18;16(10):1918. doi: 10.3390/cancers16101918. PMID: 38791996; PMCID: PMC11119080. | 2 |
| 1. Jiang Z, Yao X, Lou Y, Feng S. Prognostic factors of tongue cancer in children and adolescents: A SEER population-based study. Head Neck. 2024 Nov;46(11):2798-2805. doi: 10.1002/hed.27828. Epub 2024 May 25. PMID: 38794884. | 2 |
| 1. Yoder AK, Farooqi A, Mitra D, Livingston JA, Araujo DM, Sturgis EM, Goepfert R, Bishop AJ, Guadagnolo BA. Outcomes for Patients With Head and Neck Sarcoma Treated Curatively With Radiation Therapy and Surgery. Pract Radiat Oncol. 2024 Sep-Oct;14(5):e373-e382. doi: 10.1016/j.prro.2024.05.006. Epub 2024 Jun 6. PMID: 38851534. | 7 |
| 1. Little DJ, Ballo MT, Zagars GK, Pisters PW, Patel SR, El-Naggar AK, Garden AS, Benjamin RS. Adult rhabdomyosarcoma: outcome following multimodality treatment. Cancer. 2002 Jul 15;95(2):377-88. doi: 10.1002/cncr.10669. PMID: 12124838. | 2 |
| 1. Rozan O, Janot F, Oberlin O, Schwaab G, Quintana E, Tran Ba Huy P, Georges B, Luboinski B. Les rhabdomyosarcomes de la fosse infratemporale de l'enfant [Pediatric rhabdomyosarcoma of the infratemporal fossa]. Ann Otolaryngol Chir Cervicofac. 2002 Sep;119(4):195-201. French. PMID: 12410115. | 7 |
| 1. Patel SG, Meyers P, Huvos AG, Wolden S, Singh B, Shaha AR, Boyle JO, Pfister D, Shah JP, Kraus DH. Improved outcomes in patients with osteogenic sarcoma of the head and neck. Cancer. 2002 Oct 1;95(7):1495-503. doi: 10.1002/cncr.10849. PMID: 12237918. | 2 |
| 1. Neves BMJ, Pontes PA, Caran EM, Figueiredo C, Weckx LL. Rabdomiosarcoma de cabeça e pescoço na infância. Rev Bras Otorrinolaringol. 2003;69(1):24-8.doi: 10.1590/S0034-72992003000100005 | 2 |
| 1. Wollina U, Schönlebe J, Koch A, Haroske G. Atypical fibroxanthoma: a series of 25 cases. J Eur Acad Dermatol Venereol. 2010 Aug;24(8):943-6. doi: 10.1111/j.1468-3083.2010.03578.x. Epub 2010 Feb 1. PMID: 20158588. | 5 |
| 1. Borucki RB, Neskey DM, Lentsch EJ. Malignant fibrous histiocytoma: Database review suggests a favorable prognosis in the head and neck. Laryngoscope. 2018 Apr;128(4):885-888. doi: 10.1002/lary.26909. Epub 2017 Oct 8. PMID: 28988458. | 2 |
| 1. Trofymenko O, Bordeaux JS, Zeitouni NC. Survival in patients with primary dermatofibrosarcoma protuberans: National Cancer Database analysis. J Am Acad Dermatol. 2018 Jun;78(6):1125-1134. doi: 10.1016/j.jaad.2017.11.030. Epub 2017 Nov 23. PMID: 29175214. | 2 |
| 1. Arboleda LPA, Hoffmann IL, Cardinalli IA, Santos-Silva AR, de Mendonça RMH. Demographic and clinicopathologic distribution of head and neck malignant tumors in pediatric patients from a Brazilian population: A retrospective study. J Oral Pathol Med. 2018 Aug;47(7):696-705. doi: 10.1111/jop.12724. Epub 2018 May 22. PMID: 29729040. | 2 |
| 1. Janz TA, Camilon PR, Cheung AY, Nguyen SA, White DR, Weber PC. A review of pediatric middle ear tumors and analysis of the demographics, management, and survival of pediatric rhabdomyosarcomas of the middle ear. Int J Pediatr Otorhinolaryngol. 2018 Sep;112:109-112. doi: 10.1016/j.ijporl.2018.06.017. Epub 2018 Jun 12. PMID: 30055718. | 2 |
| 1. Woods RH, Potter JA, Reid JL, Louise J, Bessen T, Farshid G, Neuhaus SJ. Patterns of head and neck sarcoma in Australia. ANZ J Surg. 2018 Sep;88(9):901-906. doi: 10.1111/ans.14018. Epub 2017 May 16. PMID: 28512869. | 2 |
| 1. Cannon RB, Carpenter PS, Boothe D, Buchmann LO, Hunt JP, Lloyd S, Hitchcock YJ, Houlton JJ, Weis JR, Shepherd HM, Monroe MM. Academic Facility Utilization and Survival Outcomes in Adult Head and Neck Sarcomas: An NCDB Analysis. Otolaryngol Head Neck Surg. 2018 Sep;159(3):473-483. doi: 10.1177/0194599818768495. Epub 2018 Apr 17. PMID: 29661049. | 6 |
| 1. Olson MD, Van Abel KM, Wehrs RN, Garcia JJ, Moore EJ. Ewing sarcoma of the head and neck: The Mayo Clinic experience. Head Neck. 2018 Sep;40(9):1999-2006. doi: 10.1002/hed.25191. Epub 2018 May 13. PMID: 29756246. | 6 |
| 1. Mpunga T, Znaor A, Uwizeye FR, Uwase A, Munyanshongore C, Franceschi S, Clifford GM. A case-control study of HIV infection and cancer in the era of antiretroviral therapy in Rwanda. Int J Cancer. 2018 Sep 15;143(6):1348-1355. doi: 10.1002/ijc.31537. Epub 2018 Apr 26. PMID: 29663358; PMCID: PMC6099235. | 2 |
| 1. Kobayashi K, Matsumoto F, Miyakita Y, Mori T, Shimoi T, Murakami N, Yoshida A, Arakawa A, Omura G, Fukasawa M, Matsumoto Y, Matsumura S, Itami J, Narita Y, Yoshimoto S. Impact of Surgical Margin in Skull Base Surgery for Head and Neck Sarcomas. J Neurol Surg B Skull Base. 2018 Oct;79(5):437-444. doi: 10.1055/s-0037-1615816. Epub 2018 Jan 9. PMID: 30210970; PMCID: PMC6133685. | 6 |
| 1. Cassidy RJ, Switchenko JM, Yushak ML, Madden N, Khan MK, Monson DK, Beitler JJ, Landry JC, Godette KD, Gillespie TW, Patel KR. The importance of surgery in scalp angiosarcomas. Surg Oncol. 2018 Dec;27(4):A3-A8. doi: 10.1016/j.suronc.2018.07.010. Epub 2018 Sep 13. PMID: 30237037; PMCID: PMC6261443. | 2 |
| 1. Cassidy RJ, Switchenko JM, Yushak ML, Madden N, Khan MK, Monson DK, Beitler JJ, Landry JC, Godette KD, Gillespie TW, Patel KR. The importance of surgery in scalp angiosarcomas. Surg Oncol. 2018 Dec;27(4):A3-A8. doi: 10.1016/j.suronc.2018.07.010. Epub 2018 Sep 13. PMID: 30237037; PMCID: PMC6261443. | 2 |
| 1. Coindre JM, Pelmus M, Hostein I, Lussan C, Bui BN, Guillou L. Should molecular testing be required for diagnosing synovial sarcoma? A prospective study of 204 cases. Cancer. 2003 Dec 15;98(12):2700-7. doi: 10.1002/cncr.11840. PMID: 14669292. | 2 |
| 1. Bouaoud J, Temam S, Cozic N, Galmiche-Rolland L, Belhous K, Kolb F, Bidault F, Bolle S, Dumont S, Laurence V, Plantaz D, Tabone MD, Marec-Berard P, Quassemyar Q, Couloigner V, Picard A, Gomez-Brouchet A, Le Deley MC, Mahier-Ait Oukhatar C, Kadlub N, Gaspar N. Ewing's Sarcoma of the Head and Neck: Margins are not just for surgeons. Cancer Med. 2018 Dec;7(12):5879-5888. doi: 10.1002/cam4.1801. Epub 2018 Nov 17. PMID: 30449071; PMCID: PMC6308064. | 6 |
| 1. Atarbashi-Moghadam S, Emami Razavi AN, Salehi Zalani S. Prevalence of Head and Neck Sarcoma in a Major Cancer Center in Iran- A 10-Year Study. Iran J Otorhinolaryngol. 2019 Mar;31(103):97-102. PMID: 30989075; PMCID: PMC6449527. | 2 |
| 1. Maddox, J. C., & Evans, H. L. (1981). Angiosarcoma of skin and soft tissue: a study of forty‐four cases. *Cancer*, *48*(8), 1907-1921. | 2 |
| 1. Simon JH, Paulino AC, Smith RB, Buatti JM. Prognostic factors in head and neck rhabdomyosarcoma. Head Neck. 2002 May;24(5):468-73. doi: 10.1002/hed.10070. PMID: 12001077. | 6 |
| 1. Chen C, Shu HK, Goldwein JW, Womer RB, Maity A. Volumetric considerations in radiotherapy for pediatric parameningeal rhabdomyosarcomas. Int J Radiat Oncol Biol Phys. 2003 Apr 1;55(5):1294-9. doi: 10.1016/s0360-3016(02)04290-6. PMID: 12654440. | 6 |
| 1. Pawlik TM, Paulino AF, McGinn CJ, Baker LH, Cohen DS, Morris JS, Rees R, Sondak VK. Cutaneous angiosarcoma of the scalp: a multidisciplinary approach. Cancer. 2003 Oct 15;98(8):1716-26. doi: 10.1002/cncr.11667. PMID: 14534889. | 6 |
| 1. Smith RB, Apostolakis LW, Karnell LH, Koch BB, Robinson RA, Zhen W, Menck HR, Hoffman HT. National Cancer Data Base report on osteosarcoma of the head and neck. Cancer. 2003 Oct 15;98(8):1670-80. doi: 10.1002/cncr.11716. PMID: 14534884. | 2 |
| 1. Rai B, Kapoor R, Ghoshal S, Sharma SC. Rhabdomyosarcoma of head and neck: a ten year review. JK Sci. 2003;5(3):101-103. | 6 |
| 1. Bentz BG, Singh B, Woodruff J, Brennan M, Shah JP, Kraus D. Head and neck soft tissue sarcomas: a multivariate analysis of outcomes. Ann Surg Oncol. 2004 Jun;11(6):619-28. doi: 10.1245/ASO.2004.03.006. PMID: 15172935. | 6 |
| 1. Liu WW, Chen ZH, Wu QL, Wu GH, Zeng ZY. [Clinicopathological features and prognosis of postirradiation osteosarcoma in nasopharyngeal carcinoma]. Zhonghua Er Bi Yan Hou Tou Jing Wai Ke Za Zhi. 2005 Nov;40(11):814-8. Chinese. PMID: 16408745. | 6 |
| 1. Rapidis AD, Gakiopoulou H, Stavrianos SD, Vilos GA, Faratzis G, Douzinas EE, Givalos N, Patsouris E. Sarcomas of the head and neck. Results from the treatment of 25 patients. Eur J Surg Oncol. 2005 Mar;31(2):177-82. doi: 10.1016/j.ejso.2004.09.020. PMID: 15698735. | 6 |
| 1. Ohguri T, Imada H, Nomoto S, Yahara K, Hisaoka M, Hashimoto H, Tokura Y, Nakamura K, Shioyama Y, Honda H, Terashima H, Moroi Y, Furue M, Korogi Y. Angiosarcoma of the scalp treated with curative radiotherapy plus recombinant interleukin-2 immunotherapy. Int J Radiat Oncol Biol Phys. 2005 Apr 1;61(5):1446-53. doi: 10.1016/j.ijrobp.2004.08.008. PMID: 15817349. | 6 |
| 1. Wolden SL, Wexler LH, Kraus DH, Laquaglia MP, Lis E, Meyers PA. Intensity-modulated radiotherapy for head-and-neck rhabdomyosarcoma. Int J Radiat Oncol Biol Phys. 2005 Apr 1;61(5):1432-8. doi: 10.1016/j.ijrobp.2004.08.005. PMID: 15817347. | 6 |
| 1. Lajer CB, Daugaard S, Hansen HS, Kirkegaard J, Holmgaard S, Christensen ME. Soft tissue sarcomas of the head and neck: a single-centre experience. Clin Otolaryngol. 2005 Apr;30(2):176-82. doi: 10.1111/j.1365-2273.2004.00951.x. PMID: 15839871. | 6 |
| 1. Buwalda J, Freling NJ, Blank LE, Balm AJ, Bras J, Voûte PA, Caron HN, Schouwenburg PF, Merks JH. AMORE protocol in pediatric head and neck rhabdomyosarcoma: descriptive analysis of failure patterns. Head Neck. 2005 May;27(5):390-6. doi: 10.1002/hed.20164. PMID: 15825203. | 6 |
| 1. Wurm J, Constantinidis J, Grabenbauer GG, Iro H. Rhabdomyosarcomas of the nose and paranasal sinuses: treatment results in 15 cases. Otolaryngol Head Neck Surg. 2005 Jul;133(1):42-50. doi: 10.1016/j.otohns.2005.03.023. PMID: 16025051. | 6 |
| 1. Huber GF, Matthews TW, Dort JC. Soft-tissue sarcomas of the head and neck: a retrospective analysis of the Alberta experience 1974 to 1999. Laryngoscope. 2006 May;116(5):780-5. doi: 10.1097/01.MLG.0000206126.48315.85. PMID: 16652087. | 2 |
| 1. de Bree R, van der Valk P, Kuik DJ, van Diest PJ, Doornaert P, Buter J, Eerenstein SE, Langendijk JA, van der Waal I, Leemans CR. Prognostic factors in adult soft tissue sarcomas of the head and neck: a single-centre experience. Oral Oncol. 2006 Aug;42(7):703-9. doi: 10.1016/j.oraloncology.2005.11.009. Epub 2006 Mar 10. PMID: 16529978. | 6 |
| 1. Ni S, Xu ZG, Wang XL, Liu SY, Lu N, Xue LY. [Clinical analysis of dermatofibrosarcoma protuberans in head and neck]. Zhonghua Er Bi Yan Hou Tou Jing Wai Ke Za Zhi. 2007 Jun;42(6):404-7. Chinese. PMID: 17702411. | 6 |
| 1. Harb WJ, Luna MA, Patel SR, Ballo MT, Roberts DB, Sturgis EM. Survival in patients with synovial sarcoma of the head and neck: association with tumor location, size, and extension. Head Neck. 2007 Aug;29(8):731-40. doi: 10.1002/hed.20564. PMID: 17274049. | 2 |
| 1. Penel N, Mallet Y, Robin YM, Fournier C, Grosjean J, Ceugnart L, Clisant S, Lefebvre JL. Prognostic factors for adult sarcomas of head and neck. Int J Oral Maxillofac Surg. 2008 May;37(5):428-32. doi: 10.1016/j.ijom.2008.01.019. Epub 2008 Mar 14. PMID: 18343096. | 2 |
| 1. Bisogno G, De Rossi C, Gamboa Y, Sotti G, Ferrari A, Dallorso S, Donfrancesco A, Cecchetto G, Calderone M, Gandola L, Rosolen A, Carli M. Improved survival for children with parameningeal rhabdomyosarcoma: results from the AIEOP soft tissue sarcoma committee. Pediatr Blood Cancer. 2008 Jun;50(6):1154-8. doi: 10.1002/pbc.21527. PMID: 18300319. | 6 |
| 1. Bonadies A, Bertozzi E, Cristiani R, Govoni FA, Migliano E. Electrochemotherapy in Skin Malignancies of Head and Neck Cancer Patients: Clinical Efficacy and Aesthetic Benefits. Acta Derm Venereol. 2019 Dec 1;99(13):1246-1252. doi: 10.2340/00015555-3341. PMID: 31612236. | 2 |
| 1. Vanidassane I, Sharma A, Aggarwal A, Gunasekar S, Barwad A, Dhamija E, Pandey R, Deo S, Garg R, Rastogi S. Multimodality treatment of head-and-neck soft-tissue sarcomas and short-term outcomes: Analysis from sarcoma medical oncology clinic. South Asian J Cancer. 2019 Jan-Mar;8(1):69-71. doi: 10.4103/sajc.sajc_229_18. PMID: 30766860; PMCID: PMC6348781. | 2 |
| 1. Köhler HF, Neves RI, Brechtbühl ER, Mattos Granja NV, Ikeda MK, Kowalski LP. Cutaneous angiosarcoma of the head and neck: report of 23 cases from a single institution. Otolaryngol Head Neck Surg. 2008 Oct;139(4):519-24. doi: 10.1016/j.otohns.2008.07.022. PMID: 18922337. | 2 |
| 1. Huber GF, Dziegielewski P, Wayne Matthews T, Dort JC. Head and neck osteosarcoma in adults: the province of alberta experience over 26 years. J Otolaryngol Head Neck Surg. 2008 Oct;37(5):738-43. PMID: 19128686. | 2 |
| 1. McDonald MW, Esiashvili N, George BA, Katzenstein HM, Olson TA, Rapkin LB, Marcus RB Jr. Intensity-modulated radiotherapy with use of cone-down boost for pediatric head-and-neck rhabdomyosarcoma. Int J Radiat Oncol Biol Phys. 2008 Nov 1;72(3):884-91. doi: 10.1016/j.ijrobp.2008.01.058. Epub 2008 May 1. PMID: 18455321. | 2 |
| 1. Davis EC, Ballo MT, Luna MA, Patel SR, Roberts DB, Nong X, Sturgis EM. Liposarcoma of the head and neck: The University of Texas M. D. Anderson Cancer Center experience. Head Neck. 2009 Jan;31(1):28-36. doi: 10.1002/hed.20923. PMID: 18767171. | 2 |
| 1. Fayda M, Aksu G, Yaman Agaoglu F, Karadeniz A, Darendeliler E, Altun M, Hafiz G. The role of surgery and radiotherapy in treatment of soft tissue sarcomas of the head and neck region: review of 30 cases. J Craniomaxillofac Surg. 2009 Jan;37(1):42-8. doi: 10.1016/j.jcms.2008.07.007. Epub 2008 Sep 18. PMID: 18804382. | 2 |
| 1. Blank LE, Koedooder K, Pieters BR, van der Grient HN, van de Kar M, Buwalda J, Balm AJ, Merks JH, Strackee SD, Freling NJ, Koning CC. The AMORE protocol for advanced-stage and recurrent nonorbital rhabdomyosarcoma in the head-and-neck region of children: a radiation oncology view. Int J Radiat Oncol Biol Phys. 2009 Aug 1;74(5):1555-62. doi: 10.1016/j.ijrobp.2008.10.029. Epub 2009 Feb 26. PMID: 19250759. | 6 |
| 1. Guadagnolo BA, Zagars GK, Raymond AK, Benjamin RS, Sturgis EM. Osteosarcoma of the jaw/craniofacial region: outcomes after multimodality treatment. Cancer. 2009 Jul 15;115(14):3262-70. doi: 10.1002/cncr.24297. PMID: 19382187. | 2 |
| 1. Zeng Q, Tang PZ, Xu ZG, Qi YF, Wu XX, Liu WS. Primary malignant fibrous histiocytoma of the thyroid. Eur J Surg Oncol. 2009 Jun;35(6):649-53. doi: 10.1016/j.ejso.2008.09.001. Epub 2008 Oct 14. PMID: 18922667. | 6 |
| 1. Velez Torres JM, Martinez Duarte E, Diaz-Perez JA, Leibowitz J, Weed DT, Thomas G, Civantos FJ, Arnold DJ, Gomez-Fernandez C, Rosenberg AE. Primary Sarcomas of the Larynx: A Clinicopathologic Study of 27 Cases. Head Neck Pathol. 2021 Sep;15(3):905-916. doi: 10.1007/s12105-021-01314-8. Epub 2021 Mar 8. PMID: 33686585; PMCID: PMC8384992. | 6 |
| 1. Mori S, Taki T, Murakami Y, Urata T, Okumura M, Akanabe H, Ebata A, Imai S, Yokota K, Akiyama M. Low plasma fibrinogen levels are associated with poor prognosis in cutaneous angiosarcoma of the head and neck. Cancer Sci. 2021 Sep;112(9):3924-3927. doi: 10.1111/cas.15037. Epub 2021 Jul 12. PMID: 34252257; PMCID: PMC8409292. | 6 |
| 1. Adeola JO, Patel JS, Povolotskiy R, Barinsky GL, Grube JG, Hsueh WD, Baredes S, Eloy JA. Clinicopathologic characteristics of laryngeal chondrosarcoma: An analysis of the National Cancer Database. Auris Nasus Larynx. 2021 Oct;48(5):956-962. doi: 10.1016/j.anl.2021.02.014. Epub 2021 Mar 31. PMID: 33812757. | 6 |
| 1. Shim T, Chillakuru Y, Darwish C, Chalif E, Strum D, Benito DA, Mulcahy CF, Monfared A. Head and neck osteosarcomas: Analysis of treatment trends and survival outcomes in the United States (2004-2016). Head Neck. 2021 Nov;43(11):3294-3305. doi: 10.1002/hed.26817. Epub 2021 Jul 17. PMID: 34272901. | 2 |
| 1. Yanzon A, Gomez NL, Picco P, Boccalatte L, Cayol F, Larrañaga J, Figari M. Head and neck sarcomas: treatment outcomes in a tertiary referral center in Argentina. Oral Maxillofac Surg. 2021 Dec;25(4):509-518. doi: 10.1007/s10006-021-00944-0. Epub 2021 Feb 15. PMID: 33587234. | 2 |
| 1. Schmidt Jensen J, Grønhøj C, Ruud Kjær EK, Charabi BW, von Buchwald C, Hjuler T. Second primary cancers in pediatric head and neck cancer survivors in Denmark during 1980-2014: A nationwide study. Int J Pediatr Otorhinolaryngol. 2019 Dec;127:109648. doi: 10.1016/j.ijporl.2019.109648. Epub 2019 Aug 23. PMID: 31472358. | 1 |
| 1. Sun N, Wang SC, Ma XL, Zhang J, Su Y, Liu ZK, Liu YH, Yu GX, Li YZ, Zhang XX, Liu QY, Liu ZY, Ni X. [Efficacy and influencing factors of surgery combined with neoadjuvant chemoradiotherapy in the treatment of children with non-orbital head and neck rhabdomyosarcoma]. Zhonghua Er Bi Yan Hou Tou Jing Wai Ke Za Zhi. 2022 Dec 7;57(12):1403-1408. Chinese. doi: 10.3760/cma.j.cn115330-20220429-00236. PMID: 36707943. | 2 |
| 1. Janz TA, Nagasubramanian R, Wei JL. Pediatric head and neck fibrosarcomas: A demographical, treatment, and survival analysis and review of a rare case. Int J Pediatr Otorhinolaryngol. 2019 Jan;116:92-96. doi: 10.1016/j.ijporl.2018.09.031. Epub 2018 Oct 2. PMID: 30554717. | 2 |
| 1. Turri-Zanoni M, Dalfino G, Lechner M, Dallan I, Battaglia P, Facco C, Franzi F, Gravante G, Ferrari M, Terzakis D, Jay A, Forster MD, Ambrosoli AL, Bignami M, Georgalas C, Herman P, Nicolai P, Lund VJ, Castelnuovo P. Biphenotypic sinonasal sarcoma: European multicentre case-series and systematic literature review. Acta Otorhinolaryngol Ital. 2022 Dec;42(6):545-553. doi: 10.14639/0392-100X-N2087. PMID: 36654521; PMCID: PMC9853106. | 2 |
| 1. Müller CSL, Kreie L, Bochen F, Pfuhl T, Smola S, Gräber S, Vogt T, Schick B, Linxweiler M. Expression of 3q oncogene SEC62 in atypical fibroxanthoma-immunohistochemical analysis of 41 cases and correlation with clinical, viral and histopathologic features. Oncol Lett. 2019 Feb;17(2):1768-1776. doi: 10.3892/ol.2018.9767. Epub 2018 Nov 27. PMID: 30675236; PMCID: PMC6341582. | 6 |
| 1. Logan IT, Vroobel KM, le Grange F, Perrett CM. Pleomorphic dermal sarcoma: Clinicopathological features and outcomes from a 5-year tertiary referral centre experience. Cancer Rep (Hoboken). 2022 Nov;5(11):e1583. doi: 10.1002/cnr2.1583. Epub 2021 Nov 11. PMID: 34766474; PMCID: PMC9675369. | 6 |
| 1. Roos JH, Mäkitie AA, Tarkkanen J, Ilmarinen TT. Pretreatment tumor sampling and prognostic factors in patients with soft-tissue sarcoma of the head and neck. Eur Arch Otorhinolaryngol. 2022 Jun;279(6):3147-3155. doi: 10.1007/s00405-021-07162-0. Epub 2021 Nov 12. Erratum in: Eur Arch Otorhinolaryngol. 2022 Jun;279(6):3157. doi: 10.1007/s00405-021-07205-6. PMID: 34773167; PMCID: PMC9072459. | 2 |
| 1. Rimmer RA, Mace JC, Andersen PE, Cetas JS, Ciporen JN, Dogan A, Smith TL, Geltzeiler M. Determinants of survival in sinonasal and skull base chondrosarcoma: An analysis of the National Cancer Database. Int Forum Allergy Rhinol. 2022 May;12(5):699-713. doi: 10.1002/alr.22909. Epub 2021 Oct 26. PMID: 34704402. | 2 |
| 1. Musha A, Kubo N, Kawamura H, Okano N, Sato H, Okada K, Osu N, Yumisaki H, Adachi A, Takayasu Y, Shino M, Nikkuni O, Ida S, Shirai K, Saitoh JI, Yokoo S, Chikamatsu K, Ohno T. Carbon-ion Radiotherapy for Inoperable Head and Neck Bone and Soft-tissue Sarcoma: Prospective Observational Study. Anticancer Res. 2022 Mar;42(3):1439-1446. doi: 10.21873/anticanres.15614. PMID: 35220237. | 2 |
| 1. Ben-Arush M, Minard-Colin V, Scarzello G, Fajardo RD, Terwisscha Van Scheltinga S, Bernier V, Jenney M, Gallego S, Zanetti I, Cesen M, Merks JHM, Bisogno G. Therapy and prognostic significance of regional lymph node involvement in embryonal rhabdomyosarcoma: a report from the European paediatric Soft tissue sarcoma Study Group. Eur J Cancer. 2022 Sep;172:119-129. doi: 10.1016/j.ejca.2022.05.033. Epub 2022 Jun 25. PMID: 35763871. | 2 |
| 1. Patra S, Trivedi P, Shah A. Primary sarcoma of the head and neck: three years' experience in a tertiary care cancer center. Asian J Oncol. 2021;8(2):63-8. doi: 10.1055/s-0041-1728219. | 6 |
| 1. Talati VM, Urban MJ, Patel TR, Wojcik C, Tajudeen BA, Stenson K, Bhayani MK, Al-Khudari S, Husain IA. Laryngeal Chondrosarcoma Characteristics and Survival Analysis in the National Cancer Database. Otolaryngol Head Neck Surg. 2022 Jan;166(1):101-108. doi: 10.1177/01945998211004578. Epub 2021 Apr 13. PMID: 33848444. | 6 |
| 1. Hahn E, Barot S, O'Sullivan B, Huang SH, Gupta A, Hosni A, Razak AA, Waldron J, Irish J, Gullane P, Brown D, Gilbert R, de Almeida JR, Tsang D, Shultz DB. Adult Head and Neck Rhabdomyosarcoma: Management, Outcomes, and the Effect of Intensity Modulated Radiation Therapy on Locoregional Control. Adv Radiat Oncol. 2022 Aug 27;7(6):101055. doi: 10.1016/j.adro.2022.101055. PMID: 36420200; PMCID: PMC9677199. | 2 |
| 1. Ramakrishnan N, Mokhtari R, Charville GW, Bui N, Ganjoo K. Cutaneous Angiosarcoma of the Head and Neck-A Retrospective Analysis of 47 Patients. Cancers (Basel). 2022 Aug 8;14(15):3841. doi: 10.3390/cancers14153841. PMID: 35954504; PMCID: PMC9367417. | 6 |
| 1. Chen JR, Li XY. Treatment and prognosis of head and neck rhabdomyosarcoma in children. Zhonghua Er Bi Yan Hou Tou Jing Wai Ke Za Zhi. 2022;57(12):1540-4. | 4 |
| 1. Machavoine R, Helfre S, Bernier V, Bolle S, Leseur J, Corradini N, Rome A, Defachelles AS, Deneuve S, Bernard S, Fayoux P, Nicollas R, Mondain M, Luscan R, Denoyelle F, Simon F, Kadlub N, Kolb F, Honart JF, Orbach D, Minard-Colin V, Moya-Plana A, Couloigner V. Locoregional Control and Survival in Children, Adolescents, and Young Adults With Localized Head and Neck Alveolar Rhabdomyosarcoma-The French Experience. Front Pediatr. 2022 Feb 4;9:783754. doi: 10.3389/fped.2021.783754. PMID: 35186818; PMCID: PMC8855824. | 2 |
| 1. Tsuchihashi K, Ito M, Arita S, Kusaba H, Kusano W, Matsumura T, Kitazono T, Ueno S, Taguchi R, Yoshihiro T, Doi Y, Arimizu K, Ohmura H, Kajitani T, Nio K, Nakano M, Oshima K, Tamura S, Shirakawa T, Shimokawa H, Uchino K, Hanamura F, Okumura Y, Komoda M, Isobe T, Ariyama H, Esaki T, Hashimoto K, Komune N, Matsuo M, Matsumoto K, Asai K, Yoshitake T, Yamamoto H, Oda Y, Akashi K, Baba E. Survival outcomes including salvage therapy of adult head and neck para-meningeal rhabdomyosarcoma: a multicenter retrospective study from Japan. BMC Cancer. 2023 Oct 31;23(1):1046. doi: 10.1186/s12885-023-11528-4. PMID: 37904096; PMCID: PMC10617040. | 6 |
| 1. Tzelnick S, Soroka HP, Tasnim N, Gilbert RW, Irish JC, Goldstein DP, Brown D, Gullane P, Chepeha DB, Yao CMKL, Sahovaler A, Witterick IJ, Monteiro E, Davies J, Huang SH, O'Sullivan B, Hahn E, Hosni A, Razak AA, Gupta AA, de Almeida JR. The impact of surgical resection margins on outcomes for adults with head and neck osteosarcomas: A Canadian sarcoma research and Clinical Collaboration (CanSaRCC) study. Oral Oncol. 2023 Oct;145:106495. doi: 10.1016/j.oraloncology.2023.106495. Epub 2023 Jul 19. PMID: 37478572. | 2 |
| 1. Yoder AK, Farooqi AS, Wernz C, Subramaniam A, Ravi V, Goepfert R, Sturgis EM, Mitra D, Bishop AJ, Guadagnolo BA. Outcomes after definitive treatment for cutaneous angiosarcomas of the face and scalp: Reevaluating the role of surgery and radiation therapy. Head Neck. 2023 Aug;45(8):1943-1951. doi: 10.1002/hed.27418. Epub 2023 Jun 5. PMID: 37272774; PMCID: PMC11350586. | 6 |
| 1. Liang W, Li L, Wang M, Liu Y, Qiu Y, Zhang B, Wu Q, Wei Y. Non-inferior efficacy of non-surgical treatment to surgical treatment in patients with nonmetastatic head and neck rhabdomyosarcoma: a SEER-based study. Clin Transl Oncol. 2023 Jun;25(6):1779-1792. doi: 10.1007/s12094-023-03076-x. Epub 2023 Jan 14. PMID: 36640207. | 6 |
| 1. Go CC, Lahaie Luna GM, Briceño CA. Epidemiological trends and survival outcomes for dermatofibrosarcoma protuberans of the head and neck region. Int J Dermatol. 2023 May;62(5):664-671. doi: 10.1111/ijd.16459. Epub 2022 Nov 1. PMID: 36318642. | 6 |
| 1. Janz TA, Long BD, Joshi RR, Coblens OM. Survival differences of low-grade versus high-grade head and neck pleomorphic dermal sarcomas and a review of a scalp case. World J Otorhinolaryngol Head Neck Surg. 2022 Apr 18;9(1):74-78. doi: 10.1002/wjo2.64. PMID: 37006751; PMCID: PMC10050964. | 6 |
| 1. Wu Q, Wang J, Li S, Liu J, Cheng Y, Jin J, Zhong Y. Comparison of Definitive Radiotherapy-Based Treatment and Surgical-Based Treatment for Locally Advanced Head and Neck Soft Tissue Sarcoma. J Clin Med. 2023 Apr 24;12(9):3099. doi: 10.3390/jcm12093099. PMID: 37176541; PMCID: PMC10179011. | 2 |
| 1. Evans LK, Sutton S, Echanique K, Armaneous M, Palacios V, Sajed D, St John M. Cutaneous head and neck angiosarcoma: The 30-year UCLA experience. Laryngoscope Investig Otolaryngol. 2023 Oct 17;8(6):1557-1563. doi: 10.1002/lio2.1173. PMID: 38130244; PMCID: PMC10731542. | 2 |
| 1. Chen WY, Lu SH, Wang YM, Wang CW, Fang KH, Lai SF, Liang HK, Huang BS. Post-irradiation sarcoma after definitive radiation therapy for nasopharyngeal carcinoma. Radiother Oncol. 2023 Jan;178:109423. doi: 10.1016/j.radonc.2022.11.012. Epub 2022 Nov 23. PMID: 36435339. | 6 |
| 1. Zhao D, Zhou F, Liu W, Huang Z, Xu X, Zheng B, Liu C, Bai C, Liu J, Sun Y, Wang W, Xiao S. Adult head and neck rhabdomyosarcoma: radiotherapy- based treatment, outcomes, and predictors of survival. BMC Cancer. 2024 Mar 14;24(1):340. doi: 10.1186/s12885-024-12079-y. PMID: 38486204; PMCID: PMC10938762. | 2 |
| 1. Schleich M, Laccourreye L, Marianowski R, Dufour X, Babin E, Bastit V, Marie JP, Badoual C, Philouze P, Espitalier F, Du Bouexic De Pinieux G, Moriniere S. Treatment strategy in laryngeal chondrosarcoma: a multicenter study of 43 cases. Eur Arch Otorhinolaryngol. 2024 Feb;281(2):883-890. doi: 10.1007/s00405-023-08248-7. Epub 2023 Sep 27. PMID: 37752251. | 6 |
| 1. Gangadharan J, Mathews A, Prasanna Kumary SN, Somanathan T, Jayasree K, Narayanan G. Angiosarcoma of the head and neck: A clinicopathologic study with special emphasis on diagnostic pitfalls. Indian J Pathol Microbiol. 2024 Jul 1;67(3):559-563. doi: 10.4103/ijpm.ijpm_655_22. Epub 2024 Feb 14. PMID: 38391320. | 2 |
| 1. Evans J, Chang C, Jones C, Anderson I, Berner JE, Crowley TP, Ragbir M. Clinical characteristics and treatment outcomes of angiosarcoma of the head and neck: A 17-year single-centre experience. J Plast Reconstr Aesthet Surg. 2024 Jan;88:452-456. doi: 10.1016/j.bjps.2023.11.039. Epub 2023 Nov 29. PMID: 38091688. | 6 |
| 1. Hammami B, Bouayed W, Siala W, Toumi N, Khabir A, Boudawara T, Frikha M, Daoud J, Charfeddine I, Ghorbel A. Les sarcomes de la tête et du cou [Head and neck sarcoma]. Ann Otolaryngol Chir Cervicofac. 2008 Dec;125(6):294-300. French. doi: 10.1016/j.aorl.2008.09.001. Epub 2008 Oct 19. PMID: 18937933. | 2 |
| 1. Sawhney R, Ahsanuddin S, Sheorey L, Wassef DW, Baredes S, Park RCW. Understanding giant cell sarcoma of the head and neck: A population-based study. Head Neck. 2021 Sep;43(9):2786-2794. doi: 10.1002/hed.26765. Epub 2021 Jun 14. PMID: 34121252. | 6 |
| 1. Wan H, Zhang D, Hu W, Xie Z, Du Q, Xia Q, Wen T, Jia H. Aberrant PTEN, PIK3CA, pMAPK, and TP53 expression in human scalp and face angiosarcoma. Medicine (Baltimore). 2021 Jul 30;100(30):e26779. doi: 10.1097/MD.0000000000026779. PMID: 34397726; PMCID: PMC8322557. | 6 |
| 1. Ram H, Kumar S, Singh SN, Kumar P, Singh G, Ganguly R, Sagar M, Howlader D. Head and Neck Sarcomas-clinicopathological Findings, Treatment Modalities and Its Outcome - A Retrospective Study. Ann Maxillofac Surg. 2021 Jul-Dec;11(2):280-286. doi: 10.4103/ams.ams_366_20. Epub 2022 Feb 1. PMID: 35265499; PMCID: PMC8848714. | 6 |
| 1. Glosli H, Bisogno G, Kelsey A, Chisholm JC, Gaze M, Kolb F, McHugh K, Shipley J, Gallego S, Merks JHM, Smeele LE, Mandeville H, Ferrari A, Minard-Colin V, Corradini N, Jenney M, Zanetti I, De Salvo GL, Orbach D; EpSSG members. Non-parameningeal head and neck rhabdomyosarcoma in children, adolescents, and young adults: Experience of the European paediatric Soft tissue sarcoma Study Group (EpSSG) - RMS2005 study. Eur J Cancer. 2021 Jul;151:84-93. doi: 10.1016/j.ejca.2021.04.007. Epub 2021 May 7. PMID: 33971448. | 6 |
| 1. Asioli S, Ruengwanichayakun P, Zoli M, Guaraldi F, Sollini G, Greco P, Facco C, Gibertoni D, Jiménez BV, Benini S, Turri-Zanoni M, Pasquini E, Mazzatenta D, Foschini MP, Righi A. Association of Clinicopathological Features With Outcome in Chondrosarcomas of the Head and Neck. Otolaryngol Head Neck Surg. 2021 Apr;164(4):807-814. doi: 10.1177/0194599820957271. Epub 2020 Sep 15. PMID: 32928034. | 6 |
| 1. Dombrowski ND, Wolter NE, Robson CD, Kawai K, Irace AL, Vargas SO, Marcus KJ, Mack JW, Collins NB, Rahbar R. Role of Surgery in Rhabdomyosarcoma of the Head and Neck in Children. Laryngoscope. 2021 Mar;131(3):E984-E992. doi: 10.1002/lary.28785. Epub 2020 Jul 20. PMID: 33107076. | 6 |
| 1. Curry SD, Jiang ZY, Jain KS. Population-based survival of pediatric rhabdomyosarcoma of the head and neck over four decades. Int J Pediatr Otorhinolaryngol. 2021 Mar;142:110599. doi: 10.1016/j.ijporl.2020.110599. Epub 2020 Dec 29. PMID: 33422992. | 6 |
| 1. Akagündüz B, Akin Telli T, Sezgin Goksu S, Yildirim HC, Ozer M, Göktaş Aydin S, Ozyurt N, Karacin C, Paydas S, Dogan M. Assessment of Prognostic Factors and Adjuvant Treatment Modalities in Adult Head and Neck Soft Tissue Sarcoma Patients Treated With Upfront Surgery. Cureus. 2021 Feb 13;13(2):e13324. doi: 10.7759/cureus.13324. PMID: 33738167; PMCID: PMC7958307. | 6 |
| 1. Rastatter JC, Sinard RN, Dilger A, Reichek J, Walterhouse DO, Patel U. Survival of Patients With Non-Rhabdomyosarcoma Soft Tissue Sarcomas of the Head and Neck. Laryngoscope. 2021 Feb;131(2):E500-E508. doi: 10.1002/lary.28789. Epub 2020 Jun 12. PMID: 32531087. | 6 |
| 1. Lee KT, Moon J, Jeong HS, Lim HS, Lim SY. Benefits of the Multidisciplinary Approach After Curative Surgery for the Treatment of Scalp Angiosarcoma. Ann Plast Surg. 2021 Jan;86(1):39-45. doi: 10.1097/SAP.0000000000002462. PMID: 32541541. | 6 |
| 1. González A, Etchichury D, Rivero JM, Adamo L. Treatment of dermatofibrosarcoma of the head and neck with Mohs surgery with paraffin sections. J Plast Reconstr Aesthet Surg. 2021 May;74(5):1061-1070. doi: 10.1016/j.bjps.2020.10.062. Epub 2020 Nov 7. PMID: 33317985. | 6 |
| 1. Fatusi OA, Ajike SO, Olateju SO, Adebayo AT, Gbolahan OO, Ogunmuyiwa SA. Clinico-epidemiological analysis of orofacial rhabdomyosarcoma in a Nigerian population. Int J Oral Maxillofac Surg. 2009 Mar;38(3):256-60. doi: 10.1016/j.ijom.2008.12.002. Epub 2009 Jan 18. PMID: 19153030. | 6 |
| 1. Choi YJ, Han SS, Lee C, Jeon KJ. CT and MR imaging findings of head and neck chondrosarcoma. Oral Radiol. 2024 Apr;40(2):242-250. doi: 10.1007/s11282-023-00729-z. Epub 2023 Dec 18. PMID: 38108955. | 2 |
| 1. Zhen H, Liu Z, Guan H, Ma J, Wang W, Shen J, Miao Z, Zhang F. Second Malignant Neoplasms in Patients With Rhabdomyosarcoma. Front Oncol. 2021 Oct 14;11:757095. doi: 10.3389/fonc.2021.757095. PMID: 34722311; PMCID: PMC8553267. | 6 |
| 1. Kuan CH, Yang HW, Huang HF, Jyuhn-Hsiarn Lee L, Tseng TY, Hsieh JH, Cheng NC, Tai HC, Lai HS. Prognostic significance of positive surgical margins for scalp angiosarcoma. J Formos Med Assoc. 2021 Jan;120(1 Pt 1):217-225. doi: 10.1016/j.jfma.2020.04.018. Epub 2020 May 21. PMID: 32446755. | 6 |
| 1. Darwish C, Shim T, Sparks AD, Chillakuru Y, Strum D, Benito DA, Monfared A. Pediatric head and neck rhabdomyosarcoma: An analysis of treatment and survival in the United States (1975-2016). Int J Pediatr Otorhinolaryngol. 2020 Dec;139:110403. doi: 10.1016/j.ijporl.2020.110403. Epub 2020 Sep 25. PMID: 33049553. | 6 |
| 1. Torabi SJ, Bourdillon A, Salehi PP, Kafle S, Mehra S, Rahmati R, Judson BL. The epidemiology, surgical management, and impact of margins in skull and mandibular osseous-site tumors. Head Neck. 2020 Nov;42(11):3352-3363. doi: 10.1002/hed.26389. Epub 2020 Aug 2. PMID: 32743892. | 6 |
| 1. Lonie S, Yau B, Henderson M, Gyorki D, Angel C, Webb A. Management of pleomorphic dermal sarcoma. ANZ J Surg. 2020 Nov;90(11):2322-2324. doi: 10.1111/ans.15909. Epub 2020 Apr 27. PMID: 32338819. | 6 |
| 1. Ong AC, Huh EH, Moreland AJ, Rooper LM, Aygun N, Akst LM, Best SR, Khan MA. Nonepithelial Tumors of the Larynx: Single-Institution 13-Year Review with Radiologic-Pathologic Correlation. Radiographics. 2020 Nov-Dec;40(7):2011-2028. doi: 10.1148/rg.2020190210. Epub 2020 Oct 9. PMID: 33035134. | 6 |
| 1. Torabi SJ, Izreig S, Kasle DA, Benchetrit L, Salehi PP, Judson BL. Clinical characteristics and treatment-associated survival of head and neck Ewing sarcoma. Laryngoscope. 2020 Oct;130(10):2385-2392. doi: 10.1002/lary.28412. Epub 2019 Nov 27. PMID: 31774563. | 6 |
| 1. Pondrom M, Bougeard G, Karanian M, Bonneau-Lagacherie J, Boulanger C, Boutroux H, Briandet C, Chevreau C, Corradini N, Coze C, Defachelles AS, Galmiche-Roland L, Orbach D, Piguet C, Scoazec JY, Vérité C, Willems M, Frebourg T, Minard V, Brugières L. Rhabdomyosarcoma associated with germline TP53 alteration in children and adolescents: The French experience. Pediatr Blood Cancer. 2020 Sep;67(9):e28486. doi: 10.1002/pbc.28486. Epub 2020 Jul 13. PMID: 32658383. | 6 |
| 1. Hala Aziz Shokralla, Ahmed Elsayed Fathalla. Head and Neck Synovial Sarcoma: Egyptian National Cancer Institute Experience.Medical Science, 2020, 24(105), 3121-3127 | 2 |
| 1. Wen et al. Risk factors for disease progression and prognosis of pediatric head and neck rhabdomyosarcoma. Chinese Journal of Applied Clinical Pediatrics 2021 36:15 (1152-1156) | 6 |
| 1. Zhang Y, Zhang WL, Huang DS, Wang YZ, Hu HM, Mei YY, Zhi T. Prognostic factors in children with head and neck rhabdomyosarcoma: A 12-year retrospective study. Brain Behav. 2020 Aug;10(8):e01697. doi: 10.1002/brb3.1697. Epub 2020 Jun 16. PMID: 32548972; PMCID: PMC7428493. | 6 |
| 1. Kang Y, Bae J, Choi S, Jang KT, Yu J, Hong JY, Lim SY, Jeong HS. Regional Lymph Node Metastasis of Scalp Angiosarcoma: A Detailed Clinical Observation Study of 40 Cases. Ann Surg Oncol. 2020 Aug;27(8):3018-3027. doi: 10.1245/s10434-020-08408-7. Epub 2020 May 26. PMID: 32458324. | 6 |
| 1. Chang C, Wu SP, Hu K, Li Z, Schreiber D, Oliver J, Givi B. Patterns of Care and Survival of Cutaneous Angiosarcoma of the Head and Neck. Otolaryngol Head Neck Surg. 2020 Jun;162(6):881-887. doi: 10.1177/0194599820905495. Epub 2020 Feb 11. PMID: 32043919. | 6 |
| 1. Wen Y, Huang D, Zhang W, Zhang Y, Hu H, Li J. Radiation therapy is an important factor to improve survival in pediatric patients with head and neck rhabdomyosarcoma by enhancing local control: a historical cohort study from a single center. BMC Pediatr. 2020 May 29;20(1):265. doi: 10.1186/s12887-020-02165-y. PMID: 32471472; PMCID: PMC7260775. | 6 |
| 1. Moon IJ, Kim YJ, Won CH, Chang SE, Lee MW, Choi JH, Lee WJ. Clinicopathological and survival analyses of primary cutaneous angiosarcoma in an Asian population: prognostic value of the clinical features of skin lesions. Int J Dermatol. 2020 May;59(5):582-589. doi: 10.1111/ijd.14828. Epub 2020 Mar 6. PMID: 32141614. | 6 |
| 1. Wen Y, Huang D, Zhang W, Zhang Y, Hu H, Li J. Radiation therapy is an important factor to improve survival in pediatric patients with head and neck rhabdomyosarcoma by enhancing local control: a historical cohort study from a single center. BMC Pediatr. 2020 May 29;20(1):265. doi: 10.1186/s12887-020-02165-y. PMID: 32471472; PMCID: PMC7260775. | 6 |
| 1. Moreira DGL, da Silva LP, de Morais EF, Queiroz SIML, de Moura Santos E, de Souza LB, de Almeida Freitas R. The occurrence and pattern of head and neck sarcomas: a comprehensive cancer center experience. Eur Arch Otorhinolaryngol. 2020 May;277(5):1473-1480. doi: 10.1007/s00405-020-05834-x. Epub 2020 Feb 4. PMID: 32020312. | 6 |
| 1. Jiménez I, Laé M, Tanguy ML, Savignoni A, Gauthier-Villars M, Desjardins L, Cassoux N, Dendale R, Rodriguez J, Doz F, Brisse HJ, Aerts I. Craniofacial second primary tumors in patients with germline retinoblastoma previously treated with external beam radiotherapy: A retrospective institutional analysis. Pediatr Blood Cancer. 2020 Apr;67(4):e28158. doi: 10.1002/pbc.28158. Epub 2020 Jan 6. PMID: 31904159. | 6 |
| 1. Iglesias-Pena N, López-Solache L, Martínez-Campayo N, Meilán-Sánchez I, Yebra-Pimentel MT, Balboa-Barreiro V, Paradela S, Fonseca E. Incidence rate and clinicopathological features of 62 atypical fibroxanthomas in a North-Western Spanish population. Australas J Dermatol. 2020 Feb;61(1):e22-e27. doi: 10.1111/ajd.13102. Epub 2019 Jul 1. PMID: 31264202. | 6 |
| 1. Luo Z, Chen W, Shen X, Qin G, Yuan J, Hu B, Lyu J, Wen C, Xu W. Head and neck osteosarcoma: CT and MR imaging features. Dentomaxillofac Radiol. 2020 Feb;49(2):20190202. doi: 10.1259/dmfr.20190202. Epub 2019 Oct 30. PMID: 31642708; PMCID: PMC7026925. | 2 |
| 1. Tian et al. Expression and clinical significance of lin-28 homolog B in adult head and neck rhabdomyosarcoma. Academic Journal of Second Military Medical University 2020 41:2 (129-134). | 7 |
| 1. Mantilla JG, Xu H, Ricciotti RW. Primary Sarcomas of the Larynx: A Single Institutional Experience with Ten Cases. Head Neck Pathol. 2020 Sep;14(3):707-714. doi: 10.1007/s12105-019-01106-1. Epub 2019 Dec 7. PMID: 31813100; PMCID: PMC7413934. | 6 |
| 1. Duhok, Iraq et al. Paediatric malignant blue cell tumours- A practical pathological and immunohistochemical study in Duhok, Iraq. Journal of Clinical and Diagnostic Research 2020 14:9 (EC10-EC15) | 2 |
| 1. Aytekin MN, Öztürk R, Amer K, Yapar A. Epidemiology, incidence, and survival of synovial sarcoma subtypes: SEER database analysis. J Orthop Surg (Hong Kong). 2020 Jan-Apr;28(2):2309499020936009. doi: 10.1177/2309499020936009. PMID: 32618221. | 6 |
| 1. Han S, Yin X, Xu W, Wang Y, Han W. The Management of Head and Neck Sarcoma. J Craniofac Surg. 2020 Mar/Apr;31(2):e189-e192. doi: 10.1097/SCS.0000000000006162. PMID: 31934973. | 6 |
| 1. Mremi A, Mswima J, Mlay MG, Bartholomew H, Alloyce JP, Mmbaga BT, Bartlett J. Cancer spectrum in HIV-infected patients: A zonal hospital experience in Tanzania. Cancer Treat Res Commun. 2020;25:100213. doi: 10.1016/j.ctarc.2020.100213. Epub 2020 Sep 29. PMID: 33038569; PMCID: PMC9887343. | 6 |
| 1. Amalia et al. Epidemiology Profile and Prognostic Factors of Childhood Rhabdomyosarcoma in Indonesia: A Five Year Single Institution Study. Mal J Med Health Sci 16(SUPP3): 27-31, June 2020 | 6 |
| 1. Aalling M, Klug TE, Ovesen T. Head and neck sarcomas: the first report addressing the duration of symptoms and diagnostic work up. Acta Otolaryngol. 2020 Jun;140(6):521-525. doi: 10.1080/00016489.2020.1733656. Epub 2020 Mar 18. PMID: 32186247. | 6 |
| 1. Liu Z, Zhu F, Cao W, Sun J, Zhang C, He Y. Surgical treatment of pediatric rhabdomyosarcoma in the parameningeal-nonparameningeal region. J Craniomaxillofac Surg. 2020 Jan;48(1):75-82. doi: 10.1016/j.jcms.2019.12.002. Epub 2019 Dec 16. PMID: 31902716. | 6 |
| 1. Zhu et al. Clinical characteristics and prognosis of pediatric non-rhabdomyosarcoma soft tissue sarcomas. Chinese Journal of Applied Clinical PediatricsVolume 35, Issue 15, Pages 1147 - 11515 August 2020 | 6 |
| 1. Kleinerman RA, Schonfeld SJ, Sigel BS, Wong-Siegel JR, Gilbert ES, Abramson DH, Seddon JM, Tucker MA, Morton LM. Bone and Soft-Tissue Sarcoma Risk in Long-Term Survivors of Hereditary Retinoblastoma Treated With Radiation. J Clin Oncol. 2019 Dec 10;37(35):3436-3445. doi: 10.1200/JCO.19.01096. Epub 2019 Oct 17. PMID: 31622129; PMCID: PMC7001778. | 6 |
| 1. Mowery A, Clayburgh D. Malignant peripheral nerve sheath tumors: Analysis of the national cancer database. Oral Oncol. 2019 Nov;98:13-19. doi: 10.1016/j.oraloncology.2019.09.010. Epub 2019 Sep 13. PMID: 31525622. | 6 |
| 1. Torabi SJ, Cheraghlou S, Kasle DA, Savoca EL, Judson BL. Nonsquamous cell laryngeal cancers: Incidence, demographics, care patterns, and effect of surgery. Laryngoscope. 2019 Nov;129(11):2496-2505. doi: 10.1002/lary.27785. Epub 2019 Jan 10. PMID: 30632157. | 6 |
| 1. Jaly AA, Thway K, Touska P, Wale A, Miah A, Kerawala C, Riva F, Moskovic E, Jones RL, Strauss D, Dafydd DA, Messiou C. Imaging Soft-tissue Sarcomas of the Head and Neck: A Tertiary Soft-tissue Sarcoma Unit Experience. Anticancer Res. 2019 Nov;39(11):6223-6230. doi: 10.21873/anticanres.13831. PMID: 31704851. | 6 |
| 1. Xu N, Duan C, Jin M, Zhang DW, Su Y, Yu T, He LJ, Fu LB, Zeng Q, Wang HM, Zhang WP, Ni X, Ma XL. [Clinical and prognostic analysis of single-center multidisciplinary treatment for rhabdomyosarcoma in children]. Zhonghua Er Ke Za Zhi. 2019 Oct 2;57(10):767-773. Chinese. doi: 10.3760/cma.j.issn.0578-1310.2019.10.008. PMID: 31594063. | 2 |
| 1. Lee KC, Chuang SK, Philipone EM, Peters SM. Characteristics and Prognosis of Primary Head and Neck Angiosarcomas: A Surveillance, Epidemiology, and End Results Program (SEER) Analysis of 1250 Cases. Head Neck Pathol. 2019 Sep;13(3):378-385. doi: 10.1007/s12105-018-0978-3. Epub 2018 Oct 24. PMID: 30357539; PMCID: PMC6684669. | 6 |
| 1. Bertucci F, Finetti P, Monneur A, Perrot D, Chevreau C, Le Cesne A, Blay JY, Mir O, Birnbaum D. PARP1 expression in soft tissue sarcomas is a poor-prognosis factor and a new potential therapeutic target. Mol Oncol. 2019 Jul;13(7):1577-1588. doi: 10.1002/1878-0261.12522. Epub 2019 Jun 7. PMID: 31131495; PMCID: PMC6599836. | 2 |
| 1. Toki S, Kobayashi E, Yoshida A, Ogura K, Wakai S, Yoshimoto S, Yonemori K, Kawai A. A clinical comparison between dedifferentiated low-grade osteosarcoma and conventional osteosarcoma. Bone Joint J. 2019 Jun;101-B(6):745-752. doi: 10.1302/0301-620X.101B6.BJJ-2018-1207.R1. PMID: 31154837. | 2 |
| 1. Cannon RB, Kull AJ, Carpenter PS, Francis S, Buchmann LO, Monroe MM, Lloyd S, Hitchcock YJ, Cannon D, Weis JR, Houlton JJ, Hunt JP. Adjuvant radiation for positive margins in adult head and neck sarcomas is associated with improved survival: Analysis of the National Cancer Database. Head Neck. 2019 Jun;41(6):1873-1879. doi: 10.1002/hed.25619. Epub 2019 Jan 16. PMID: 30652375. | 2 |
| 1. Casey DL, Wexler LH, Wolden SL. Worse Outcomes for Head and Neck Rhabdomyosarcoma Secondary to Reduced-Dose Cyclophosphamide. Int J Radiat Oncol Biol Phys. 2019 Apr 1;103(5):1151-1157. doi: 10.1016/j.ijrobp.2018.11.049. Epub 2018 Nov 30. Erratum in: Int J Radiat Oncol Biol Phys. 2020 Jan 1;106(1):223. doi: 10.1016/j.ijrobp.2019.10.013. PMID: 30508617; PMCID: PMC6441953. | 2 |
| 1. Martin E, Muskens IS, Coert JH, Smith TR, Broekman MLD. Treatment and survival differences across tumor sites in malignant peripheral nerve sheath tumors: a SEER database analysis and review of the literature. Neurooncol Pract. 2019 Mar;6(2):134-143. doi: 10.1093/nop/npy025. Epub 2018 Jul 19. PMID: 31386019; PMCID: PMC6656331. | 6 |
| 1. Tallegas M, Miquelestorena-Standley É, Labit-Bouvier C, Badoual C, Francois A, Gomez-Brouchet A, Aubert S, Collin C, Tallet A, de Pinieux G. IDH mutation status in a series of 88 head and neck chondrosarcomas: different profile between tumors of the skull base and tumors involving the facial skeleton and the laryngotracheal tract. Hum Pathol. 2019 Feb;84:183-191. doi: 10.1016/j.humpath.2018.09.015. Epub 2018 Oct 5. PMID: 30296521. | 6 |
| 1. Martin E, Radomski S, Harley EH. Pediatric Ewing sarcoma of the head and neck: A retrospective survival analysis. Int J Pediatr Otorhinolaryngol. 2019 Feb;117:138-142. doi: 10.1016/j.ijporl.2018.11.026. Epub 2018 Nov 26. PMID: 30579068. | 2 |
| 1. Siddiqui SH, Siddiqui E, Bavier RD, Patel NM, Kiliç S, Baredes S, Hsueh WD, Eloy JA. Clinicopathologic traits and prognostic factors associated with pediatric sinonasal rhabdomyosarcoma. Int Forum Allergy Rhinol. 2019 Apr;9(4):363-369. doi: 10.1002/alr.22267. Epub 2019 Jan 10. PMID: 30629809. | 6 |
| 743.Harrison L, McCulloch T, Beasley N. Soft tissue head and neck sarcoma: experience of a tertiary referral centre over a 15-year period. J Laryngol Otol. 2019 Dec;133(12):1053-1058. doi: 10.1017/S0022215119002299. Epub 2019 Nov 29. PMID: 31779724. | 6 |
| 744.Lou J, Jiang L, Dai X, Wang H, Yang J, Guo L, Fang M, Wang S. Radiation-Induced Sarcoma of the Head and Neck Following Radiotherapy for Nasopharyngeal Carcinoma: A Single Institutional Experience and Literature Review. Front Oncol. 2021 Jan 21;10:526360. doi: 10.3389/fonc.2020.526360. PMID: 33552942; PMCID: PMC7858657. | 9 |
| 745. Li X, Peng J, Liu Z, Cui Z, Zhang P, Jin H. [Clinical analysis of 35 cases of adult rhabdomyosarcoma of nasal cavity and sinuses]. Lin Chuang Er Bi Yan Hou Tou Jing Wai Ke Za Zhi. 2020 Mar;34(3):223-226. Chinese. doi: 10.13201/j.issn.2096-7993.2020.03.009. PMID: 32791587; PMCID: PMC10127844. | 6 |
| 746.Wen et al. Risk factors for disease progression and prognosis of pediatric head and neck rhabdomyosarcoma. Chinese Journal of Applied Clinical Pediatrics 2021 36:15 (1152-1156) | 6 |
| 747. Chen WL, Liu YM, Zhou B, Chen R, Lin ZY, Huang ZQ, Huang ZX. En bloc resection and reconstruction in patients with advanced recurrent nasopharyngeal carcinoma and radiation-induced sarcoma of the head and neck. Int J Oral Maxillofac Surg. 2021 Jun;50(6):711-717. doi: 10.1016/j.ijom.2020.04.022. Epub 2020 Nov 30. PMID: 33272770. | 6 |
| 748.Giannini L, Bresciani L, Paderno A, Incandela F, Fiore M, Gronchi A, Piazza C. Head and neck adult-type soft tissues sarcomas: survival analysis and comparison between the last two editions of the TNM staging system. Eur Arch Otorhinolaryngol. 2021 Aug;278(8):3003-3010. doi: 10.1007/s00405-020-06452-3. Epub 2020 Nov 5. PMID: 33151383. | 6 |
| 749. Fujisawa Y, Yoshino K, Kadono T, Miyagawa T, Nakamura Y, Fujimoto M. Chemoradiotherapy with taxane is superior to conventional surgery and radiotherapy in the management of cutaneous angiosarcoma: a multicentre, retrospective study. Br J Dermatol. 2014 Dec;171(6):1493-500. doi: 10.1111/bjd.13110. Epub 2014 Nov 10. PMID: 24814962. | 6 |
| 750.Salcedo-Hernández RA, Lino-Silva LS, Mosqueda-Taylor A, Luna-Ortiz K. Soft tissue sarcomas of the head and neck. Clinical and pathological evaluation of 108 cases in Mexico. J Craniomaxillofac Surg. 2014 Dec;42(8):1566-71. doi: 10.1016/j.jcms.2014.01.033. Epub 2014 Mar 5. PMID: 24704280. | 6 |
| 751.Raphael S, Yusuf I, Imam I. Childhood rhabdomyosarcoma in Kano, Nigeria: a retrospective analysis of 52 cases. Niger J Med. 2015 Jan-Mar;24(1):32-6. PMID: 25807671. | 4 |
| 752.Arshi A, Tajudeen BA, St John M. Malignant peripheral nerve sheath tumors of the head and neck: Demographics, clinicopathologic features, management, and treatment outcomes. Oral Oncol. 2015 Dec;51(12):1088-94. doi: 10.1016/j.oraloncology.2015.08.012. Epub 2015 Oct 9. PMID: 26442813. | 6 |
| 753.Patel TD, Shaigany K, Fang CH, Park RC, Baredes S, Eloy JA. Comparative Analysis of Head and Neck and Non-Head and Neck Malignant Peripheral Nerve Sheath Tumors. Otolaryngol Head Neck Surg. 2016 Jan;154(1):113-20. doi: 10.1177/0194599815606700. Epub 2015 Sep 25. PMID: 26408559. | 6 |
| 754.Mallen-St Clair J, Arshi A, Abemayor E, St John M. Factors Associated With Survival in Patients With Synovial Cell Sarcoma of the Head and Neck: An Analysis of 167 Cases Using the SEER (Surveillance, Epidemiology, and End Results) Database. JAMA Otolaryngol Head Neck Surg. 2016 Jun 1;142(6):576-83. doi: 10.1001/jamaoto.2016.0384. PMID: 27100936; PMCID: PMC6173585. | 6 |
| 755.Schoot RA, Saeed P, Freling NJ, Blank LE, Pieters BR, van der Grient JN, Strackee SD, Bras J, Caron HN, Merks JH; Amsterdam Head and Neck Working Group on Childhood Tumors. Local Resection and Brachytherapy for Primary Orbital Rhabdomyosarcoma: Outcome and Failure Pattern Analysis. Ophthalmic Plast Reconstr Surg. 2016 Sep-Oct;32(5):354-60. doi: 10.1097/IOP.0000000000000562. PMID: 26398242. | 6 |
| 756.Liuzzi JF, Da Cunha M, Salas D, Siso S, Garriga E. Soft-tissue sarcomas in the head and neck: 25 years of experience. Ecancermedicalscience. 2017 Jun 2;11:740. doi: 10.3332/ecancer.2017.740. PMID: 28626490; PMCID: PMC5464559. | 6 |
| 757.Ellis MA, Gerry DR, Neskey DM, Lentsch EJ. Ewing Sarcoma of the Head and Neck. Ann Otol Rhinol Laryngol. 2017 Mar;126(3):179-184. doi: 10.1177/0003489416681322. Epub 2017 Jan 5. PMID: 28056517; PMCID: PMC6477678. | 6 |
| 758.Rodríguez-Jiménez et al. Treatment and prognosis of angiosarcoma: Experience with 12 patients and review of new therapeutic lines. Medicina Cutanea Ibero-Latino-AmericanaVolume 45, Issue 2, Pages 112 - 118May-August 2017 | 6 |
| 759.Breakey RW, Crowley TP, Anderson IB, Milner RH, Ragbir M. The surgical management of head and neck sarcoma: The Newcastle experience. J Plast Reconstr Aesthet Surg. 2017 Jan;70(1):78-84. doi: 10.1016/j.bjps.2016.09.026. Epub 2016 Oct 5. PMID: 27836568. | 6 |
| 760.Owosho AA, Estilo CL, Huryn JM, Zhang L, Fletcher CDM, Antonescu CR. Head and Neck Round Cell Sarcomas: A Comparative Clinicopathologic Analysis of 2 Molecular Subsets: Ewing and CIC-Rearranged Sarcomas. Head Neck Pathol. 2017 Dec;11(4):450-459. doi: 10.1007/s12105-017-0808-z. Epub 2017 Mar 23. PMID: 28337592; PMCID: PMC5796609. | 9 |
| 761.Zheng et al. CT and MRI features of dermatofibrosarcoma protuberans. Chinese Journal of Medical Imaging Technology 2017 33:4 (586-589) | 6 |
| 762.Unsal AA, Chung SY, Unsal AB, Baredes S, Eloy JA. A Population-Based Analysis of Survival for Sinonasal Rhabdomyosarcoma. Otolaryngol Head Neck Surg. 2017 Jul;157(1):142-149. doi: 10.1177/0194599817696292. Epub 2017 Apr 11. PMID: 28397540. | 6 |
| 763.Llombart B, Serra-Guillén C, Rubio L, Nagore E, Requena C, Traves V, Calomarde L, Bancalari B, López-Guerrero JA, Guillen-Barona C, Sanmartín O. Subcutaneous dermatofibrosarcoma protuberans, a rare subtype with predilection for the head: A retrospective series of 18 cases. J Am Acad Dermatol. 2017 Sep;77(3):503-511.e1. doi: 10.1016/j.jaad.2017.02.046. Epub 2017 Apr 15. PMID: 28420485. | 6 |
| 764.Thompson LDR, Jo VY, Agaimy A, Llombart-Bosch A, Morales GN, Machado I, Flucke U, Wakely PE Jr, Miettinen M, Bishop JA. Sinonasal Tract Alveolar Rhabdomyosarcoma in Adults: A Clinicopathologic and Immunophenotypic Study of Fifty-Two Cases with Emphasis on Epithelial Immunoreactivity. Head Neck Pathol. 2018 Jun;12(2):181-192. doi: 10.1007/s12105-017-0851-9. Epub 2017 Sep 5. PMID: 28875443; PMCID: PMC5953873. | 6 |
| 765.Sharma N, George NA, Singh R, Iype EM, Varghese BT, Thomas S. Surgical Management of Head and Neck Soft Tissue Sarcoma: 11-Year Experience at a Tertiary Care Centre in South India. Indian J Surg Oncol. 2018 Jun;9(2):187-191. doi: 10.1007/s13193-018-0755-5. Epub 2018 Apr 20. PMID: 29887699; PMCID: PMC5984866. | 6 |
| 766.Häußler SM, Stromberger C, Olze H, Seifert G, Knopke S, Böttcher A. Head and neck rhabdomyosarcoma in children: a 20-year retrospective study at a tertiary referral center. J Cancer Res Clin Oncol. 2018 Feb;144(2):371-379. doi: 10.1007/s00432-017-2544-x. Epub 2017 Nov 16. PMID: 29143871. | 6 |
| 767.Pontes FS, de Oliveira JI, de Souza LL, de Almeida OP, Fregnani ER, Vilela RS, Silva WM, Fonseca FP, Pontes HA. Clinicopathological analysis of head and neck rhabdomyosarcoma: A series of 10 cases and literature review. Med Oral Patol Oral Cir Bucal. 2018 Mar 1;23(2):e188-e197. doi: 10.4317/medoral.22106. PMID: 29476676; PMCID: PMC5911360. | 9 |
| 768. Affinita MC, Ferrari A, Milano GM, Scarzello G, De Leonardis F, Coccoli L, Pericoli R, Basso E, Zanetti I, Scagnellato A, Bisogno G. Long-term results in children with head and neck rhabdomyosarcoma: A report from the Italian Soft Tissue Sarcoma Committee. Pediatr Blood Cancer. 2018 Mar;65(3). doi: 10.1002/pbc.26876. Epub 2017 Nov 8. PMID: 29115716. | 6 |
| 769.Zhang CG, Zhou SY, Liu P, Qin Y, Yang JL, He XH, Shi YK. [Clinical characteristics and prognosis analysis of 46 cases of newly diagnosed localized head and neck rhabdomyosarcoma]. Zhonghua Yi Xue Za Zhi. 2018 Sep 11;98(34):2722-2726. Chinese. doi: 10.3760/cma.j.issn.0376-2491.2018.34.009. PMID: 30220168. | 6 |
| 770.Woo CG, Lee B, Song JS, Cho KJ. Clinicopathologic Features of the Non-CNS Primary Ewing Sarcoma Family of Tumors in the Head and Neck Region. Appl Immunohistochem Mol Morphol. 2018 Oct;26(9):632-639. doi: 10.1097/PAI.0000000000000501. PMID: 28248728. | 6 |
| 771.Boccalatte LA, Gómez NL, Yanzon A, Mazzaro EL, Cayol F, Figari MF. Head and Neck Tumors: Management of Primary Undifferentiated Pleomorphic Sarcoma. Iran J Otorhinolaryngol. 2019 Nov;31(107):335-342. doi: 10.22038/ijorl.2019.30195.1990. PMID: 31857977; PMCID: PMC6914320. | 9 |
| 772.Yunteng W, Xuhui M, Guoxin R, Wei G. Radical Surgery for Head and Neck Rhabdomyosarcoma Failed Primary Chemotherapy. J Craniofac Surg. 2019 Mar/Apr;30(2):e113-e116. doi: 10.1097/SCS.0000000000005019. PMID: 30550438. | 6 |
| 773.Li W, Lu H, Wang D. Therapeutic outcome and prognostic factors in sinonasal rhabdomyosarcoma: a single-institution case series. J Cancer Res Clin Oncol. 2019 Nov;145(11):2793-2802. doi: 10.1007/s00432-019-03009-8. Epub 2019 Aug 23. PMID: 31444550. | 6 |
| 774.Dubal PM, Svider PF, Kanumuri VV, Patel AA, Baredes S, Eloy JA. Laryngeal chondrosarcoma: a population-based analysis. Laryngoscope. 2014 Aug;124(8):1877-81. doi: 10.1002/lary.24618. Epub 2014 Mar 11. PMID: 24474667. | 6 |
| 775.Dettenborn T, Wermker K, Schulze HJ, Klein M, Schwipper V, Hallermann C. Prognostic features in angiosarcoma of the head and neck: a retrospective monocenter study. J Craniomaxillofac Surg. 2014 Dec;42(8):1623-8. doi: 10.1016/j.jcms.2014.05.002. Epub 2014 May 15. PMID: 24962043. | 6 |
| 776.Wollina U, Schönlebe J, Ziemer M, Friedling F, Koch A, Haroske G, Kaatz M, Simon JC. Atypical fibroxanthoma: a series of 56 tumors and an unexplained uneven distribution of cases in southeast Germany. Head Neck. 2015 Jun;37(6):829-34. doi: 10.1002/hed.23673. Epub 2014 Jun 20. PMID: 24946737. | 6 |
| 777. Park JT, Roh JL, Kim SO, Cho KJ, Choi SH, Nam SY, Kim SY. Prognostic factors and oncological outcomes of 122 head and neck soft tissue sarcoma patients treated at a single institution. Ann Surg Oncol. 2015 Jan;22(1):248-55. doi: 10.1245/s10434-014-3870-8. Epub 2014 Jul 8. PMID: 25001093. | 6 |
| 778.Crowson MG, Lalich I, Keeney MG, Garcia JJ, Price DL. Clinicopathologic factors and adjuvant treatment effects on survival in adult head and neck synovial cell sarcoma. Head Neck. 2015 Mar;37(3):375-80. doi: 10.1002/hed.23605. Epub 2014 Apr 3. PMID: 24430934. | 9 |
| 779.Biswas B, Thakar A, Mohanti BK, Vishnubhatla S, Bakhshi S. Prognostic factors in head and neck Ewing sarcoma family of tumors. Laryngoscope. 2015 Mar;125(3):E112-7. doi: 10.1002/lary.24985. Epub 2014 Oct 27. PMID: 25345585. | 6 |
| 780.Patel SH, Hayden RE, Hinni ML, Wong WW, Foote RL, Milani S, Wu Q, Ko SJ, Halyard MY. Angiosarcoma of the scalp and face: the Mayo Clinic experience. JAMA Otolaryngol Head Neck Surg. 2015 Apr;141(4):335-40. doi: 10.1001/jamaoto.2014.3584. PMID: 25634014. | 6 |
| 781.Yasui N, Yoshida A, Kawamoto H, Yonemori K, Hosono A, Kawai A. Clinicopathologic analysis of spindle cell/sclerosing rhabdomyosarcoma. Pediatr Blood Cancer. 2015 Jun;62(6):1011-6. doi: 10.1002/pbc.25367. Epub 2014 Dec 31. PMID: 25557260. | 9 |
| 782.Suzuki G, Yamazaki H, Takenaka H, Aibe N, Masui K, Kimoto T, Tatekawa K, Nakashima A, Takenaka T, Asai J, Komori S, Wada M, Katoh N, Yamada K. Definitive Radiation Therapy for Angiosarcoma of the Face and Scalp. In Vivo. 2016 11-12;30(6):921-926. doi: 10.21873/invivo.11014. PMID: 27815481. | 6 |
| 783.Kobayashi K, Matsumoto F, Kodaira M, Mori T, Murakami N, Yoshida A, Maki D, Teshima M, Fukasawa M, Itami J, Asai M, Yoshimoto S. Significance of delayed primary excision in localized nonmetastatic adult head and neck rhabdomyosarcoma. Cancer Med. 2016 Oct;5(10):2708-2714. doi: 10.1002/cam4.855. Epub 2016 Aug 26. PMID: 27565892; PMCID: PMC5083723. | 6 |
| 784.Patel TD, Carniol ET, Vázquez A, Baredes S, Liu JK, Eloy JA. Sinonasal fibrosarcoma: analysis of the Surveillance, Epidemiology, and End Results database. Int Forum Allergy Rhinol. 2016 Feb;6(2):201-5. doi: 10.1002/alr.21639. Epub 2015 Sep 15. PMID: 26370489. | 6 |
| 785.Owosho AA, Chen S, Kashikar S, Zhang L, Chen CL, Wexler LH, Estilo CL, Huryn JM, Antonescu CR. Clinical and molecular heterogeneity of head and neck spindle cell and sclerosing rhabdomyosarcoma. Oral Oncol. 2016 Jul;58:e6-e11. doi: 10.1016/j.oraloncology.2016.05.009. Epub 2016 May 31. PMID: 27261172; PMCID: PMC5518412. | 6 |
| 786.Orbach D, Mosseri V, Gallego S, Kelsey A, Devalck C, Brenann B, van Noesel MM, Bergeron C, Merks JH, Rechnitzer C, Jenney M, Minard-Colin V, Stevens M. Nonparameningeal head and neck rhabdomyosarcoma in children and adolescents: Lessons from the consecutive International Society of Pediatric Oncology Malignant Mesenchymal Tumor studies. Head Neck. 2017 Jan;39(1):24-31. doi: 10.1002/hed.24547. Epub 2016 Jul 26. PMID: 27459057. | 6 |
| 787.Gopalakrishnan V, Amini B, Wagner MJ, Nowell EN, Lazar AJ, Lin PP, Benjamin RS, Araujo DM. Synovial Sarcoma of the Head and Neck: A Single Institution Review. Sarcoma. 2017;2017:2016752. doi: 10.1155/2017/2016752. Epub 2017 Jun 5. PMID: 28655993; PMCID: PMC5474548. | 6 |
| 788.Chan JY, Gooi Z, Wong EW, Ng SK, Tong MC, Vlantis AC. Low-grade myofibroblastic sarcoma: A population-based study. Laryngoscope. 2017 Jan;127(1):116-121. doi: 10.1002/lary.26146. Epub 2016 Jul 5. PMID: 27377169. | 9 |
| 789.Salman et al. A review of adult head and neck soft tissue sarcoma in a tertiary centre: Malaysia experience. Bangladesh Journal of Medical ScienceOpen AccessVolume 16, Issue 1, Pages 69 - 762017 | 9 |
| 790.Zhang P, Zhao L, Zhu YJ, Qiu B, Guo SP, Li Y, Liu Q, Liu MZ, Xi M. Prognosis of Fibrosarcoma in Patients With and Without a History of Radiation for Nasopharyngeal Carcinoma. Ann Surg Oncol. 2017 Feb;24(2):434-440. doi: 10.1245/s10434-016-5589-1. Epub 2016 Sep 21. PMID: 27654106. | 6 |
| 791. Bernstein JM, Irish JC, Brown DH, Goldstein D, Chung P, Razak ARA, Catton C, Gilbert RW, Gullane PJ, O'Sullivan B. Survival outcomes for cutaneous angiosarcoma of the scalp versus face. Head Neck. 2017 Jun;39(6):1205-1211. doi: 10.1002/hed.24747. Epub 2017 Apr 11. PMID: 28398688. | 6 |
| 792. Stepan K, Konuthula N, Khan M, Parasher A, Del Signore A, Govindaraj S, Genden E, Iloreta A. Outcomes in Adult Sinonasal Rhabdomyosarcoma. Otolaryngol Head Neck Surg. 2017 Jul;157(1):135-141. doi: 10.1177/0194599817696287. Epub 2017 Mar 14. PMID: 28669309. | 6 |
| 793. Rosko AJ, Birkeland AC, Chinn SB, Shuman AG, Prince ME, Patel RM, McHugh JB, Spector ME. Survival and Margin Status in Head and Neck Radiation-Induced Sarcomas and De Novo Sarcomas. Otolaryngol Head Neck Surg. 2017 Aug;157(2):252-259. doi: 10.1177/0194599817700389. Epub 2017 Apr 11. PMID: 28397585. | 6 |
| 794.Lee RJ, Lee KK, Lin T, Arshi A, Lee SA, Christensen RE. Rhabdomyosarcoma of the head and neck: impact of demographic and clinicopathologic factors on survival. Oral Surg Oral Med Oral Pathol Oral Radiol. 2017 Sep;124(3):271-279. doi: 10.1016/j.oooo.2017.05.507. Epub 2017 May 30. PMID: 28732698. | 6 |
| 795.Luna-Ortiz K, Navarro-Santiesteban S, Villavicencio-Valencia V, Salcedo-Hernandez RA, Lino-Silva LS, Delgado JA. Primary laryngeal sarcomas in a Mexican population: Case series of eleven cases. Clin Otolaryngol. 2017 Dec;42(6):1389-1392. doi: 10.1111/coa.12889. Epub 2017 May 22. PMID: 28429517. | 6 |
| 796.Ding J, Wang C, Xiang J, Shen C, Hu C, Xu T, Lu X. Treatment Outcomes and Prognostic Factors of Adult Sinonasal Sarcomas: A Single-Institution Case Series. Med Sci Monit. 2018 Sep 2;24:6113-6118. doi: 10.12659/MSM.909116. PMID: 30173244; PMCID: PMC6131979. | 6 |
| 797. Kauke M, Safi AF, Grandoch A, Nickenig HJ, Zöller J, Kreppel M. Sarcomas of the sinonasal tract. Head Neck. 2018 Jun;40(6):1279-1286. doi: 10.1002/hed.25108. Epub 2018 Feb 14. PMID: 29443431. | 6 |
| 798. Wang Y, Li J, Tian Z, Zhu Y. Clinicopathologic features and molecular spectrum of spindle cell and sclerosing rhabdomyosarcomas in the head and neck region. Int J Clin Exp Pathol. 2018 Jul 1;11(7):3436-3444. PMID: 31949721; PMCID: PMC6962886. | 6 |
| 799.Chow TL, Kwan WW, Kwan CK. Treatment of cutaneous angiosarcoma of the scalp and face in Chinese patients: local experience at a regional hospital in Hong Kong. Hong Kong Med J. 2018 Feb;24(1):25-31. doi: 10.12809/hkmj176813. Epub 2018 Jan 12. PMID: 29326400. | 9 |
| 800.Affinita MC, Ferrari A, Milano GM, Scarzello G, De Leonardis F, Coccoli L, Pericoli R, Basso E, Zanetti I, Scagnellato A, Bisogno G. Long-term results in children with head and neck rhabdomyosarcoma: A report from the Italian Soft Tissue Sarcoma Committee. Pediatr Blood Cancer. 2018 Mar;65(3). doi: 10.1002/pbc.26876. Epub 2017 Nov 8. PMID: 29115716. | 6 |
| 801. Owosho AA, Estilo CL, Huryn JM, Chi P, Antonescu CR. A Clinicopathologic Study of Head and Neck Malignant Peripheral Nerve Sheath Tumors. Head Neck Pathol. 2018 Jun;12(2):151-159. doi: 10.1007/s12105-017-0841-y. Epub 2017 Jul 31. PMID: 28762137; PMCID: PMC5953865. | 6 |
| 802.Agaimy A, Mueller SK, Harrer T, Bauer S, Thompson LDR. Head and Neck Kaposi Sarcoma: Clinicopathological Analysis of 11 Cases. Head Neck Pathol. 2018 Dec;12(4):511-516. doi: 10.1007/s12105-018-0902-x. Epub 2018 Mar 5. PMID: 29508130; PMCID: PMC6232196. | 9 |
| 803. Oashi K, Namikawa K, Tsutsumida A, Takahashi A, Itami J, Igaki H, Inaba K, Yamazaki N. Surgery with curative intent is associated with prolonged survival in patients with cutaneous angiosarcoma of the scalp and face -a retrospective study of 38 untreated cases in the Japanese population. Eur J Surg Oncol. 2018 Jun;44(6):823-829. doi: 10.1016/j.ejso.2018.02.246. Epub 2018 Mar 6. PMID: 29555155. | 6 |
| 804. Costa Arantes DA, Gonçalves AS, Jham BC, Duarte ECB, de Paula ÉC, de Paula HM, Mendonça EF, Batista AC. Evaluation of HLA-G, HLA-E, and PD-L1 proteins in oral osteosarcomas. Oral Surg Oral Med Oral Pathol Oral Radiol. 2017 Jun;123(6):e188-e196. doi: 10.1016/j.oooo.2016.12.002. Epub 2016 Dec 14. PMID: 28159587. | 9 |
| 805.Wang S, Ge M, Wang K, Lou J, Chen X. [The clinical features and prognosis of radiotherapy associated sarcoma (RAS) following radiotherapy for nasopharyngeal carcinoma]. Zhonghua Er Bi Yan Hou Tou Jing Wai Ke Za Zhi. 2014 Nov;49(11):955-8. Chinese. PMID: 25598378. | 7 |
| 806.Bai Y, Chen X, Yan Y, Zhang S, Zhou W, Fang J, Huang Z. [Clinical efficacy analysis of adult sinonasal rhabdomyosarcoma]. Lin Chuang Er Bi Yan Hou Tou Jing Wai Ke Za Zhi. 2015 May;29(9):804-10. Chinese. PMID: 26281056. | 6 |
| 807.Chen YM, Shen QC, Gokavarapu S, Ong HS, Cao W, Ji T. Osteosarcoma of the Mandible: A Site-Specific Study on Survival and Prognostic Factors. J Craniofac Surg. 2016 Nov;27(8):1929-1933. doi: 10.1097/SCS.0000000000002968. PMID: 28005728. | 5 |
| 808. Letsa I, Benson C, Al-Muderis O, Judson I. Angiosarcoma of the face and scalp: effective systemic treatment in the older patient. J Geriatr Oncol. 2014 Jul;5(3):276-80. doi: 10.1016/j.jgo.2014.02.004. Epub 2014 Mar 28. PMID: 24685486. | 2 |
| 809.BIRDSELL, D. C. M.D.; LINDSAY, W. K. M.D.. MALIGNANT TUMORS OF THE HEAD AND NECK IN CHILDREN. Plastic and Reconstructive Surgery 44(3):p 255-260, September 1969. | 2 |
| 810.Swain, R. E., Sessions, D. G., & Ogura, J. H. (1976). Fibrosarcoma of the head and neck in children. *The Laryngoscope*, *86*(1), 113-116. | 9 |
| 811.Daw, N. C., Mahmoud, H. H., Meyer, W. H., Jenkins, J. J., Kaste, S. C., Poquette, C. A., ... & Rao, B. N. (2000). Bone sarcomas of the head and neck in children: the St. Jude Children's Research Hospital experience. *Cancer: Interdisciplinary International Journal of the American Cancer Society*, *88*(9), 2172-2180. | 2 |
| 812.Gorsky M, Epstein JB. Craniofacial osseous and chondromatous sarcomas in British Columbia--a review of 34 cases. Oral Oncol. 2000 Jan;36(1):27-31. doi: 10.1016/s1368-8375(99)00042-1. PMID: 10889915. | 2 |
| 813.Moretti G, Guimarães R, Oliveira KM, Sanjar F, Voegels RL. Rhabdomyosarcoma of the head and neck: 24 cases and literature review. Braz J Otorhinolaryngol. 2010 Jul-Aug;76(4):533-7. doi: 10.1590/S1808-86942010000400020. PMID: 20835543; PMCID: PMC9446245. | 2 |
| 814.Guadagnolo BA, Zagars GK, Araujo D, Ravi V, Shellenberger TD, Sturgis EM. Outcomes after definitive treatment for cutaneous angiosarcoma of the face and scalp. Head Neck. 2011 May;33(5):661-7. doi: 10.1002/hed.21513. Epub 2010 Oct 19. PMID: 20960566; PMCID: PMC4090937. | 6 |
| 815.Salman M, Tamim H, Medlej F, El-Ariss T, Saad F, Boulos F, Eid T, Muwakkit S, Khoury N, Abboud M, Saab R. Rhabdomyosarcoma treatment and outcome at a multidisciplinary pediatric cancer center in Lebanon. Pediatr Hematol Oncol. 2012 May;29(4):322-34. doi: 10.3109/08880018.2012.676721. PMID: 22568795. | 9 |
| 816.Koch BB, Karnell LH, Hoffman HT, Apostolakis LW, Robinson RA, Zhen W, Menck HR. National cancer database report on chondrosarcoma of the head and neck. Head Neck. 2000 Jul;22(4):408-25. doi: 10.1002/1097-0347(200007)22:4<408::aid-hed15>3.0.co;2-h. PMID: 10862026. | 2 |
| 817.Loree, T. R., North Jr, J. H., Werness, B. A., Nangia, R., Mullins, A. P., & Hicks Jr, W. L. (2000). Malignant peripheral nerve sheath tumors of the head and neck: analysis of prognostic factors. *Otolaryngology—Head and Neck Surgery*, *122*(5), 667-672. | 6 |
| 818. Swain, R. E., Sessions, D. G., & Ogura, J. H. (1974). Fibrosarcoma of the head and neck: a clinical analysis of forty cases. *Annals of Otology, Rhinology & Laryngology*, *83*(4), 439-444. | 2 |
| 819.Weichert, K. A., Bove, K. C., Aron, B. S., & Lampkin, B. (1976). Rhabdomysarcoma in Children: A Clinicopathologic Study of 35 Patients. *American Journal of Clinical Pathology*, *66*(4), 692-701. | 9 |
| 820.Gerry D, Fox NF, Spruill LS, Lentsch EJ. Liposarcoma of the head and neck: analysis of 318 cases with comparison to non-head and neck sites. Head Neck. 2014 Mar;36(3):393-400. doi: 10.1002/hed.23311. Epub 2013 Jun 1. PMID: 23728920. | 2 |
| 821.Zhu, J., Zhang, J., Tang, G., Hu, S., Zhou, G., Liu, Y., ... & Wang, Z. (2014). Computed tomography and magnetic resonance imaging observations of rhabdomyosarcoma in the head and neck. *Oncology letters*, *8*(1), 155-160. | 9 |
| 822.Yeang MS, Tay K, Ong WS, Thiagarajan A, Tan DS, Ha TC, Teo PT, Soo KC, Tan HK, Iyer NG. Outcomes and prognostic factors of post-irradiation and de novo sarcomas of the head and neck: a histologically matched case-control study. Ann Surg Oncol. 2013 Sep;20(9):3066-75. doi: 10.1245/s10434-013-2979-5. Epub 2013 Apr 19. PMID: 23604715. | 2 |
| 823.Rahman HA, Sedky M, Mohsen I, Taha H, Loaye I, Zaghloul MS, Wakeel ME, Labib RM. Outcome of pediatric parameningeal rhabdomyosarcoma. The Children Cancer Hospital, Egypt, experience. J Egypt Natl Canc Inst. 2013 Jun;25(2):79-86. doi: 10.1016/j.jnci.2013.01.002. Epub 2013 Mar 7. Erratum in: J Egypt Natl Canc Inst. 2016 Mar;28(1):63. PMID: 23719406. | 2 |
| 824. Hardison SA, Davis PL 3rd, Browne JD. Malignant fibrous histiocytoma of the head and neck: a case series. Am J Otolaryngol. 2013 Jan-Feb;34(1):10-5. doi: 10.1016/j.amjoto.2012.06.010. Epub 2012 Sep 20. PMID: 22999710. | 2 |
| 825. Andrade CR, Takahama Junior A, Nishimoto IN, Kowalski LP, Lopes MA. Rhabdomyosarcoma of the head and neck: a clinicopathological and immunohistochemical analysis of 29 cases. Braz Dent J. 2010 Jan;21(1):68-73. doi: 10.1590/s0103-64402010000100011. PMID: 20464324. | 2 |
| 826.Gadwal, S. R., Fanburg‐Smith, J. C., Gannon, F. H., & Thompson, L. D. (2000). Primary chondrosarcoma of the head and neck in pediatric patients: a clinicopathologic study of 14 cases with a review of the literature. *Cancer: Interdisciplinary International Journal of the American Cancer Society*, *88*(9), 2181-2188. | 6 |
| 827.Farr, H. W., Carandang, C. M., & Huvos, A. G. (1970). Malignant vascular tumors of the head and neck. *The American Journal of Surgery*, *120*(4), 501-504. | 2 |
| 828.Gritli S, Khamassi K, Lachkhem A, Touati S, Chorfa A, Ben Makhlouf T, El May A, Gammoudi A. Head and neck liposarcomas: a 32 years experience. Auris Nasus Larynx. 2010 Jun;37(3):347-51. doi: 10.1016/j.anl.2009.08.003. Epub 2009 Oct 25. PMID: 19857936. | 2 |
| 829.Austin SA, Hawkshaw MJ, Sataloff RT. External ear sarcoma: a review of the Surveillance Epidemiology and End Result (SEER 17) database. Ear Nose Throat J. 2011 Aug;90(8):348-58. doi: 10.1177/014556131109000808. PMID: 21853439. | 6 |
| 830.Hollmig ST, Kirkland EB, Henderson MT, Tang JY, Gladstone HB. The evolving conception and management challenges of malignant fibrous histiocytoma. Dermatol Surg. 2012 Dec;38(12):1922-9. doi: 10.1111/j.1524-4725.2012.02538.x. Epub 2012 Aug 9. PMID: 22882717. | 2 |
| 831.Yang JC, Wexler LH, Meyers PA, Wolden SL. Parameningeal rhabdomyosarcoma: outcomes and opportunities. Int J Radiat Oncol Biol Phys. 2013 Jan 1;85(1):e61-6. doi: 10.1016/j.ijrobp.2012.08.019. Epub 2012 Sep 25. PMID: 23021437. | 2 |
| 832.Pobirci DD, Bogdan F, Pobirci O, Petcu CA, Roşca E. Study of malignant fibrous histiocytoma: clinical, statistic and histopatological interrelation. Rom J Morphol Embryol. 2011;52(1 Suppl):385-8. PMID: 21424079. | 2 |
| 833.Laffers W, Stöhr G, Göke F, Wardelmann E, Keiner S, Zipfel M, Schüller H, Gerstner AO. Weichteiltumoren des Kopf-Hals-Bereichs [Soft tissue tumors of the head and neck region]. HNO. 2013 Nov;61(11):928-36. German. doi: 10.1007/s00106-013-2755-7. PMID: 23913195. | 2 |
| 834.Smith VA, Overton LJ, Lentsch EJ. Head and neck soft tissue sarcomas: unique lack of significance of synchronous node metastases. J Surg Oncol. 2012 Dec;106(7):837-43. doi: 10.1002/jso.23148. Epub 2012 May 17. PMID: 22605652. | 2 |
| 835.Trifiletti, D., Amdur, R. J., Dagan, R., Indelicato, D. J., Mendenhall, W. M., Kirwan, J. M., ... & Morris, C. G. (2012). Radiotherapy following gross total resection of adult soft tissue sarcoma of the head and neck. *Practical Radiation Oncology*, *2*(4), e121-e128. | 2 |
| 836.Zhang WL, Zhang Y, Huang DS, Guo F, Han T, Hong L, Hu HM, Zhi T. Clinical character of pediatric head and neck rhabdomysarcomas: a 7-year retrospective study. Asian Pac J Cancer Prev. 2013;14(7):4089-93. doi: 10.7314/apjcp.2013.14.7.4089. PMID: 23991958. | 2 |
| 837.Vener, J., Rice, D. H., & Newman, A. N. (1984). Osteosarcoma and chondrosarcoma of the head and neck. *The Laryngoscope*, *94*(2), 240-242. | 2 |
| 838.Patel, S. G., See, A. C., Williamson, P. A., Archer, D. J., & Rhys Evans, P. H. (1999). Radiation induced sarcoma of the head and neck. *Head & Neck: Journal for the Sciences and Specialties of the Head and Neck*, *21*(4), 346-354. | 9 |
| 839.Daya H, Chan HS, Sirkin W, Forte V. Pediatric rhabdomyosarcoma of the head and neck: is there a place for surgical management? Arch Otolaryngol Head Neck Surg. 2000 Apr;126(4):468-72. doi: 10.1001/archotol.126.4.468. PMID: 10772299. | 2 |
| 840.Allam A, El-Husseiny G, Khafaga Y, Kandil A, Gray A, Ezzat A, Schultz H. Ewing's Sarcoma of the Head and Neck: A Retrospective Analysis of 24 Cases. Sarcoma. 1999;3(1):11-5. doi: 10.1080/13577149977811. PMID: 18521259; PMCID: PMC2395405. | 2 |
| 841.Skoog, L., Pereira, S. T., & Tani, E. (1999). Fine‐needle aspiration cytology and immunocytochemistry of soft‐tissue tumors and osteo/chondrosarcomas of the head and neck. *Diagnostic cytopathology*, *20*(3), 131-136. | 2 |
| 842.Giaoui L, Salvan D, Casiraghi O, Mamelle G, Julieron M, Janot F, Leridant AM, Marandas P, Schwaab G, Luboinski B. Synovialosarcomes cervico-faciaux. Expérience de 1'Institut Gustave Roussy. A propos de 13 cas [Primary synovial sarcoma of head and neck. Materials of The Gustave Roussy Institute. Report of 13 cases]. Ann Otolaryngol Chir Cervicofac. 1999 May;116(2):71-7. French. PMID: 10378035. | 6 |
| 843.Hessa, A., Schroder, U., & Schroder, R. (1998). Rhabdomyosarcoma in the area of the head-neck: A synopsis of some cases, therapeutic possibilities and prognoses. *Laryngorhinootologie*, *77*(10), 557-563. | 9 |
| 844.Hessa, A., Schroder, U., & Schroder, R. (1998). Rhabdomyosarcoma in the area of the head-neck: A synopsis of some cases, therapeutic possibilities and prognoses. *Laryngorhinootologie*, *77*(10), 557-563. | 8 |
| 845.Aust MR, Olsen KD, Lewis JE, Nascimento AG, Meland NB, Foote RL, Suman VJ. Angiosarcomas of the head and neck: clinical and pathologic characteristics. Ann Otol Rhinol Laryngol. 1997 Nov;106(11):943-51. doi: 10.1177/000348949710601110. PMID: 9373085. | 2 |
| 846.Oda D, Bavisotto LM, Schmidt RA, McNutt M, Bruckner JD, Conrad EU 3rd, Weymuller EA Jr. Head and neck osteosarcoma at the University of Washington. Head Neck. 1997 Sep;19(6):513-23. doi: 10.1002/(sici)1097-0347(199709)19:6<513::aid-hed9>3.0.co;2-1. PMID: 9278760. | 9 |
| 847.Le QT, Fu KK, Kroll S, Fitts L, Massullo V, Ferrell L, Kaplan MJ, Phillips TL. Prognostic factors in adult soft-tissue sarcomas of the head and neck. Int J Radiat Oncol Biol Phys. 1997 Mar 15;37(5):975-84. doi: 10.1016/s0360-3016(97)00103-x. PMID: 9169803. | 2 |
| 848.Gayner, S. M., Lewis, J. E., & McCaffrey, T. V. (1997). Effect of resection margins on dermatofibrosarcoma protuberans of the head and neck. *Archives of Otolaryngology–Head & Neck Surgery*, *123*(4), 430-433. | 6 |
| 849.Sercarz, J. A., Mark, R. J., Nasri, S., Wang, M. B., & Tran, L. M. (1995). Pediatric rhabdomyosarcoma of the head and neck. *International journal of pediatric otorhinolaryngology*, *31*(1), 15-22. | 2 |
| 850.Regine WF, Fontanesi J, Kumar P, Ayers D, Bowman LC, Pappo AS, Coffey DH, Avery L, Rao BN, Kun LE. Local tumor control in rhabdomyosarcoma following low-dose irradiation: comparison of group II and select group III patients. Int J Radiat Oncol Biol Phys. 1995 Feb 1;31(3):485-91. doi: 10.1016/0360-3016(94)00352-L. PMID: 7852110. | 2 |
| 851.Morrison, W. H., Byers, R. M., Garden, A. S., Evans, H. L., Ang, K. K., & Peters, L. J. (1995). Cutaneous angiosarcoma of the head and neck. A therapeutic dilemma. *Cancer*, *76*(2), 319-327. | 6 |
| 852.Mandell LR, Massey V, Ghavimi F. The influence of extensive bone erosion on local control in non-orbital rhabdomyosarcoma of the head and neck. Int J Radiat Oncol Biol Phys. 1989 Sep;17(3):649-53. doi: 10.1016/0360-3016(89)90118-1. PMID: 2777653. | 2 |
| 853.Rao BN, Santana VM, Fleming ID, Pratt CB, Shapiro D, Fontanesi J, Kumar AP, Austin BA. Management and prognosis of head and neck sarcomas. Am J Surg. 1989 Oct;158(4):373-7. doi: 10.1016/0002-9610(89)90136-0. PMID: 2802044. | 2 |
| 854.Freedman AM, Reiman HM, Woods JE. Soft-tissue sarcomas of the head and neck. Am J Surg. 1989 Oct;158(4):367-72. doi: 10.1016/0002-9610(89)90135-9. PMID: 2802043. | 2 |
| 855.Schuller, D. E., Lawrence, T. L., & Newton, W. A. (1979). Childhood rhabdomyosarcomas of the head and neck. *Archives of Otolaryngology*, *105*(12), 689-694. | 2 |
| 856.Sanghvi S, Misra P, Patel NR, Kalyoussef E, Baredes S, Eloy JA. Incidence trends and long-term survival analysis of sinonasal rhabdomyosarcoma. Am J Otolaryngol. 2013 Nov-Dec;34(6):682-9. doi: 10.1016/j.amjoto.2013.04.012. Epub 2013 Jun 4. PMID: 23743294. | 6 |
| 857.Tefft, M., Fernandez, C., Donaldson, M., Newton, W., & Moon, T. E. (1978). Incidence of meningeal involvement by rhabdomyosarcoma of the head and neck in children. A report of the Intergroup Rhabdomyosarcoma Study (IRS). *Cancer*, *42*(1), 253-258. | 6 |
| 858.Healy, G. B., Jaffe, N., & Cassady, J. R. (1979). Rhabdomyosarcoma of the head and neck: Diagnosis and management. *Head & Neck Surgery*, *1*(4), 334-339. | 9 |
| 859.Setzen, M., Sobol, S., & Toomey, J. M. (1979). Clinical course of unusual malignant sarcomas of head and neck. *Annals of Otology, Rhinology & Laryngology*, *88*(4), 486-494. | 2 |
| 860.Roth, J. A., Enzinger, F. M., & Tannenbaum, M. (1975). Synovial sarcoma of the neck: a followup study of 24 cases. *Cancer*, *35*(4), 1243-1253. | 9 |
| 861.Michiba T, Takenaka Y, Cho H, Yamamoto Y, Yoshii T, Nakahara S, Inohara H. [Head and neck soft tissue sarcoma]. Nihon Jibiinkoka Gakkai Kaiho. 2013 Mar;116(3):154-60. Japanese. doi: 10.3950/jibiinkoka.116.154. PMID: 23678671. | 6 |
| 862.Aung L, Soe TA, Chang KT, Quah TC. Singapore rhabdomyosarcoma (RMS) experience: shall we change our practice? Ann Acad Med Singap. 2014 Feb;43(2):86-95. PMID: 24652428. | 6 |
| 863.Piazza C, Del Bon F, Grazioli P, Mangili S, Barbieri D, Nicolai P, Peretti G. Organ preservation surgery for low- and intermediate-grade laryngeal chondrosarcomas: analysis of 16 cases. Laryngoscope. 2014 Apr;124(4):907-12. doi: 10.1002/lary.24416. Epub 2013 Oct 22. PMID: 24122809. | 6 |
| 864.Gerth DJ, Tashiro J, Thaller SR. Pediatric sinonasal tumors in the United States: incidence and outcomes. J Surg Res. 2014 Jul;190(1):214-20. doi: 10.1016/j.jss.2014.04.004. Epub 2014 Apr 12. PMID: 24793449. | 6 |
| 865.Barosa J, Ribeiro J, Afonso L, Fernandes J, Monteiro E. Head and neck sarcoma: analysis of 29 cases. Eur Ann Otorhinolaryngol Head Neck Dis. 2014 Apr;131(2):83-6. doi: 10.1016/j.anorl.2012.11.007. Epub 2014 Mar 18. PMID: 24656875. | 6 |
| 866.Wu Y, Li C, Zhong Y, Guo W, Ren G. Head and neck rhabdomyosarcoma in adults. J Craniofac Surg. 2014 May;25(3):922-5. doi: 10.1097/SCS.0000000000000704. PMID: 24777012. | 2 |
| 867.Minard-Colin V, Kolb F, Saint-Rose C, Fayard F, Janot F, Rey A, Canale S, Julieron M, Corradini N, Raquin MA, Habrand JL, Grill J, George B, Ba Huy PT, Couloignier V, Terrier-Lacombe MJ, Luboinski B, Valteau-Couanet D, Oberlin O. Impact of extensive surgery in multidisciplinary approach of pterygopalatine/infratemporal fossa soft tissue sarcoma. Pediatr Blood Cancer. 2013 Jun;60(6):928-34. doi: 10.1002/pbc.24374. Epub 2013 Jan 9. PMID: 23303699. | 2 |
| 868.Gadwal, S. R., Fanburg‐Smith, J. C., Gannon, F. H., & Thompson, L. D. (2000). Primary chondrosarcoma of the head and neck in pediatric patients: a clinicopathologic study of 14 cases with a review of the literature. *Cancer: Interdisciplinary International Journal of the American Cancer Society*, *88*(9), 2181-2188. | 9 |
| 869.Chan, J. Y. W., Wong, S. T. S., Lau, G. I. S. K., & Wei, W. I. (2012). Postradiation sarcoma after radiotherapy for nasopharyngeal carcinoma. *The Laryngoscope*, *122*(12), 2695-2699. | 2 |
| 870.Zhang WL, Zhang Y, Huang DS, Guo F, Han T. [Clinical features of 39 children with head and neck rhabdomysarcoma in a single medical center, and treatment outcomes]. Zhongguo Dang Dai Er Ke Za Zhi. 2012 Nov;14(11):847-51. Chinese. PMID: 23146733. | 6 |
| 871.Wei Z, Xie Y, Xu J, Luo Y, Chen F, Yang Y, Huang Q, Tang A, Huang G. Radiation-induced sarcoma of head and neck: 50 years of experience at a single institution in an endemic area of nasopharyngeal carcinoma in China. Med Oncol. 2012 Jun;29(2):670-6. doi: 10.1007/s12032-011-9828-9. Epub 2011 Jan 23. PMID: 21259056. | 9 |
| 872.Wu AW, Suh JD, Metson R, Wang MB. Prognostic factors in sinonasal sarcomas: analysis of the surveillance, epidemiology and end result database. Laryngoscope. 2012 Oct;122(10):2137-42. doi: 10.1002/lary.23442. Epub 2012 Jul 9. PMID: 22777866. | 2 |
| 873.Ogawa K, Takahashi K, Asato Y, Yamamoto Y, Taira K, Matori S, Iraha S, Yagi N, Yogi A, Haranaga S, Fujita J, Uezato H, Murayama S. Treatment and prognosis of angiosarcoma of the scalp and face: a retrospective analysis of 48 patients. Br J Radiol. 2012 Nov;85(1019):e1127-33. doi: 10.1259/bjr/31655219. Epub 2012 Jul 17. PMID: 22806620; PMCID: PMC3500812. | 6 |
| 874.Salcedo-Hernández RA, Lino-Silva LS, Luna-Ortiz K. Synovial sarcomas of the head and neck: comparative analysis with synovial sarcoma of the extremities. Auris Nasus Larynx. 2013 Oct;40(5):476-80. doi: 10.1016/j.anl.2012.11.015. Epub 2012 Dec 21. PMID: 23260343. | 9 |
| 875.Thompson CF, Kim BJ, Lai C, Grogan T, Elashoff D, St John MA, Wang MB. Sinonasal rhabdomyosarcoma: prognostic factors and treatment outcomes. Int Forum Allergy Rhinol. 2013 Aug;3(8):678-83. doi: 10.1002/alr.21157. Epub 2013 Feb 19. PMID: 23424037; PMCID: PMC4358753. | 9 |
| 876.Obeso S, Llorente JL, Díaz-Molina JP, Sánchez-Fernández R, Rodrigo JP, Suárez C. Tratamiento quirúrgico de los condrosarcomas de cabeza y cuello [Surgical treatment of head and neck chondrosarcomas]. Acta Otorrinolaringol Esp. 2010 Jul-Aug;61(4):262-71. Spanish. doi: 10.1016/j.otorri.2009.12.002. Epub 2010 Jan 22. PMID: 20096816. | 6 |
| 877.Liebner, E. J. (1976). Embryonal rhabdomyosarcoma of head and neck in children. Correlation of stage, radiation dose, local control, and survival. *Cancer*, *37*(6), 2777-2786. | 6 |
| 878.Akinyele AO, Israel AT, Akang EE. Paediatric head and neck cancers in Nigeria: Implications for treatment planning in resource limited settings. Niger Med J. 2012 Oct;53(4):245-8. doi: 10.4103/0300-1652.107604. PMID: 23661887; PMCID: PMC3640248. | 2 |
| 879.Singh, M., Mann, R., Ilankovan, V., Hussein, K., & D'arrigo, C. (2012). Atypical fibroxanthoma—a retrospective immunohistochemical study of 42 cases. *Journal of oral and maxillofacial surgery*, *70*(11), 2713-2718. | 2 |
| 880.Deneuve S, Teissier N, Jouffroy T, Helfre S, Boissonnet H, Freneaux P, Peuchmaur M, Brisse H, Van Den Abbeele T, Orbach D. Skull base surgery for pediatric parameningeal sarcomas. Head Neck. 2012 Aug;34(8):1057-63. doi: 10.1002/hed.21865. Epub 2011 Aug 30. PMID: 22128074. | 6 |
| 881.Ablanedo-Terrazas Y, Alvarado-de la Barrera C, Ormsby CE, Reyes-Terán G. Head and neck manifestations of the immune reconstitution syndrome in HIV-infected patients: a cohort study. Otolaryngol Head Neck Surg. 2012 Jul;147(1):52-6. doi: 10.1177/0194599812437321. Epub 2012 Feb 15. PMID: 22344183. | 1 |
| 882.Van Damme JP, Schmitz S, Machiels JP, Galant C, Grégoire V, Lengelé B, Hamoir M. Prognostic factors and assessment of staging systems for head and neck soft tissue sarcomas in adults. Eur J Surg Oncol. 2010 Jul;36(7):684-90. doi: 10.1016/j.ejso.2010.05.020. Epub 2010 Jun 12. PMID: 20542404. | 2 |
| 883.Mücke T, Mitchell DA, Tannapfel A, Hölzle F, Kesting MR, Wolff KD, Kolk A, Kanatas A. Outcome in adult patients with head and neck sarcomas--a 10-year analysis. J Surg Oncol. 2010 Aug 1;102(2):170-4. doi: 10.1002/jso.21595. PMID: 20648589. | 2 |
| 884.Charhi, H., Mansouri, N., Harmouch, A., Kili, A., Khorassani, M., Khattab, M., ... & Sefiani, S. (2011). Epidemiological, histological and immunohistochemical analysis of head and neck rhabdomyosarcoma in children. *Journal Africain du Cancer/African Journal of Cancer*, *4*(3), 222-226. | 2 |
| 885.Company F, Pedram M, Rezaei N. Clinical characteristics and the prognosis of childhood rhabdomyosarcoma in 60 patients treated at a single institute. Acta Med Iran. 2011;49(4):219-24. PMID: 21713731. | 2 |
| 886.Eppsteiner RW, DeYoung BR, Milhem MM, Pagedar NA. Leiomyosarcoma of the head and neck: a population-based analysis. Arch Otolaryngol Head Neck Surg. 2011 Sep;137(9):921-4. doi: 10.1001/archoto.2011.147. PMID: 21930982; PMCID: PMC4345042. | 2 |
| 887.Piñeiro Aguín Z, León Vintró X, García Lorenzo J, Sancho FJ, López Pousa A, Quer Agustí M. Sarcomas de cabeza y cuello. Nuestra experiencia [Head and neck sarcomas. Our experience]. Acta Otorrinolaringol Esp. 2011 Nov-Dec;62(6):436-42. Spanish. doi: 10.1016/j.otorri.2011.05.005. Epub 2011 Aug 5. PMID: 21820641. | 2 |
| 888.Turner JH, Richmon JD. Head and neck rhabdomyosarcoma: a critical analysis of population-based incidence and survival data. Otolaryngol Head Neck Surg. 2011 Dec;145(6):967-73. doi: 10.1177/0194599811417063. Epub 2011 Aug 26. PMID: 21873599. | 2 |
| 889.Greager, J. A., Patel, M. K., Briele, H. A., Walker, M. J., & Gupta, T. K. D. (1985). Soft tissue sarcomas of the adult head and neck. *Cancer*, *56*(4), 820-824. | 2 |
| 890.D'Angio, G. J., Months, S. R., & Raney, R. B. (1986). Proceedings of the tumor board of the children's hospital of Philadelphia. Rhabdomyosarcoma of the head and neck in children: The experience at the children's hospital of philadelphia. *Medical and Pediatric Oncology*, *14*(5), 288-292. | 2 |
| 891.Rhabdomyosarcoma of the head and neck. [R. M. Singhal](https://www.cambridge.org/core/search?filters%5BauthorTerms%5D=R.%20M.%20Singhal&eventCode=SE-AU), [Sudhir Bahadur](https://www.cambridge.org/core/search?filters%5BauthorTerms%5D=Sudhir%20Bahadur&eventCode=SE-AU) and [Rajesh Bhatia](https://www.cambridge.org/core/search?filters%5BauthorTerms%5D=Rajesh%20Bhatia&eventCode=SE-AU). [The Journal of Laryngology & Otology](https://www.cambridge.org/core/journals/journal-of-laryngology-and-otology) , [Volume 101](https://www.cambridge.org/core/journals/journal-of-laryngology-and-otology/volume/DE0438AFFDE9E918946214B741981595) , [Issue 9](https://www.cambridge.org/core/journals/journal-of-laryngology-and-otology/issue/0B54148C15C155B1F79B77EF9E87583C) , September 1987 , pp. 971 - 974 | 2 |
| 892.Cunningham, M. J., Myers, E. N., & Bluestone, C. D. (1987). Malignant tumors of the head and neck in children a twenty-year review. *International journal of pediatric otorhinolaryngology*, *13*(3), 279-292. | 2 |
| 893.Wharam MD, Beltangady MS, Heyn RM, Lawrence W, Raney RB Jr, Ruymann FB, Soule EH, Tefft M, Maurer HM. Pediatric orofacial and laryngopharyngeal rhabdomyosarcoma. An Intergroup Rhabdomyosarcoma Study report. Arch Otolaryngol Head Neck Surg. 1987 Nov;113(11):1225-7. doi: 10.1001/archotol.1987.01860110091014. PMID: 3663351. | 2 |
| 894.Figueiredo, M. T., Marques, L. A., & Campos‐Filho, N. (1988). Soft‐tissue sarcomas of the head and neck in adults and children: Experience at a single institution with a review of literature. *International journal of cancer*, *41*(2), 198-200. | 2 |
| 895.Fiorillo, A., Migliorati, R., Grimaldi, M., Fiore, M., Menna, G., Parasole, R., ... & Muto, P. (1991). Multidisciplinary treatment of primary orbital rhabdomyosarcoma. A single‐institution experience. *Cancer*, *67*(3), 560-563. | 6 |
| 896.Gilles R, Couanet D, Chevret S, Shapeero L, Flamant F, Meunier M, Masselot J. Importance of a post-therapeutic residue in the prognosis of head and neck rhabdomyosarcoma in children. Eur J Radiol. 1991 Nov-Dec;13(3):187-91. doi: 10.1016/0720-048x(91)90026-r. PMID: 1756745. | 1 |
| 897.Amble, F. R., Olsen, K. D., Nascimento, A. G., & Foote, R. L. (1992). Head and neck synovial cell sarcoma. *Otolaryngology–Head and Neck Surgery*, *107*(5), 631-637. | 6 |
| 898.COENE, I. M., SCHOUWENBURG, P. F., VOǓTE, P., BURGERS, J. M. V., & HILGERS, F. J. (1992). Rhabdomyosarcoma of the head and neck in children. *Clinical Otolaryngology & Allied Sciences*, *17*(4), 291-296. | 2 |
| 899.Tran, L. M., Mark, R., Meier, R., Calcaterra, T. C., & Parker, R. G. (1992). Sarcomas of the head and neck. Prognostic factors and treatment strategies. *Cancer*, *70*(1), 169-177. | 6 |
| 900.Wanebo, H. J., Koness, R. J., Macfarlane, J. K., Eilber, F. R., Byers, R. M., Elias, E. G., & Spiro, R. H. (1992). Head and neck sarcoma: report of the head and neck sarcoma registry. *Head & neck*, *14*(1), 1-7. | 2 |
| 901.Eeles, R., Fisher, C., A'Hern, R. *et al.* Head and neck sarcomas: prognostic factors and implications for treatment. *Br J Cancer* 68, 201–207 (1993). https://doi.org/10.1038/bjc.1993.314 | 2 |
| 902.El-Naggar, A. K., Batsakis, J. G., Ordónez, N. G., Luna, M. A., & Goepfert, H. (1993). Rhabdomyosarcoma of the adult head and neck: a clinicopathological and DNA ploidy study. *The Journal of Laryngology & Otology*, *107*(8), 716-720. | 9 |
| 903.Mark, R. J., Bailet, J. W., Tran, L. M., Poen, J., Fu, Y. S., & Calcaterra, T. C. (1993). Dermatofibrosarcoma protuberans of the head and neck: a report of 16 cases. *Archives of Otolaryngology–Head & Neck Surgery*, *119*(8), 891-896. | 9 |
| 904.Nayar, R. C., Prudhomme, F., Parise Jr, O., Gandia, D., Luboinski, B., & Schwaab, G. (1993). Rhabdomyosarcoma of the head and neck in adults: a study of 26 patients. *The Laryngoscope*, *103*(12), 1362-1366. | 6 |
| 905.Mark, R. J., Bailet, J. W., Tran, L. M., Poen, J., Fu, Y. S., & Calcaterra, T. C. (1993). Dermatofibrosarcoma protuberans of the head and neck: a report of 16 cases. *Archives of Otolaryngology–Head & Neck Surgery*, *119*(8), 891-896. | 8 |
| 906.Greager JA, Reichard K, Campana JP, Das Gupta TK. Fibrosarcoma of the head and neck. Am J Surg. 1994 Apr;167(4):437-9. doi: 10.1016/0002-9610(94)90131-7. PMID: 8179091. | 2 |
| 907.Kowalski, L. P., & San, C. I. (1994). Prognostic factors in head and neck soft tissue sarcomas: analysis of 128 cases. *Journal of surgical oncology*, *56*(2), 83-88. | 2 |
| 908.Kraus, D. H., Dubner, S., Harrison, L. B., Strong, E. W., Hajdu, S. I., Kher, U., ... & Brennan, M. F. (1994). Prognostic factors for recurrence and survival in head and neck soft tissue sarcomas. *Cancer*, *74*(2), 697-702. | 2 |
| 909.Le Vay, J., O'Sullivan, B., Catton, C., Cummings, B., Fornasier, V., Gullane, P., & Simm, J. (1994). An assessment of prognostic factors in soft-tissue sarcoma of the head and neck. *Archives of Otolaryngology–Head & Neck Surgery*, *120*(9), 981-986. | 2 |
| 910.Lydiatt, W. M., Shaha, A. R., & Shah, J. P. (1994). Angiosarcoma of the head and neck. *The American journal of surgery*, *168*(5), 451-454. | 2 |
| 911.Singh, B., Har-El, G., & Lucente, F. E. (1994). Kaposi's sarcoma of the head and neck in patients with acquired immunodeficiency syndrome. *Otolaryngology—Head and Neck Surgery*, *111*(5), 618-624. | 2 |
| 912.Naka, N., Ohsawa, M., Tomita, Y., Kanno, H., Aozasa, K., & Uchida, A. (1995). Angiosarcoma in Japan. A review of 99 cases. *Cancer*, *75*(4), 989-996. | 2 |
| 913.Callender, T. A., Weber, R. S., Janjan, N., Benjamin, R., Zaher, M., Wolf, P., & El-Naggar, A. (1995). Rhabdomyosarcoma of the nose and paranasal sinuses in adults and children. *Otolaryngology—Head and Neck Surgery*, *112*(2), 252-257. | 2 |
| 914.Dijkstra, M. D., Balm, A. J. M., Coevorden, F. V., Gregor, R. T., Hart, A. A. M., Hilgers, F. J. M., ... & Loftus, B. M. (1996). Survival of adult patients with head and neck soft tissue sarcomas. *Clinical Otolaryngology & Allied Sciences*, *21*(1), 66-71. | 2 |
| 915.Lyos, A. T., Goepfert, H., Luna, M. A., Jaffe, N., & Malpica, A. (1996). Soft tissue sarcoma of the head and neck in children and adolescents. *Cancer: Interdisciplinary International Journal of the American Cancer Society*, *77*(1), 193-200. | 2 |
| 916.Burkey, B. B., Hoffman, H. T., Baker, S. R., Thornton, A. F., & McClatchey, K. D. (1990). Chondrosarcoma of the head and neck. *The Laryngoscope*, *100*(12), 1301-1305. | 9 |
| 917.Gasparini, M., Lombardi, F., Gianni, M. C., Massimino, M., Gandola, L., & Fossati-Bellani, F. (1990). Questionable role of CNS radioprophylaxis in the therapeutic management of childhood rhabdomyosarcoma with meningeal extension. *Journal of clinical oncology*, *8*(11), 1854-1857. | 9 |
| 918.Yousem, D. M., Lexa, F. J., Bilaniuk, L. T., & Zimmerman, R. I. (1990). Rhabdomyosarcomas in the head and neck: MR imaging evaluation. *Radiology*, *177*(3), 683-686. | 6 |
| 919.Ng YY, Kingston JE, Perry NM, Reznek RH. The role of computerized tomographic scanning in the management of rhabdomyosarcoma in nonorbital head and neck sites. Pediatr Hematol Oncol. 1990;7(2):149-57. doi: 10.3109/08880019009033385. PMID: 2206856. | 6 |
| 920.Frankenthaler, R., Goepfert, H., Ayala, A. G., & Hartwick, R. W. (1990). Fibrosarcoma of the head and neck. *The Laryngoscope*, *100*(8), 799-802. | 2 |
| 921.Farhood AI, Hajdu SI, Shiu MH, Strong EW. Soft tissue sarcomas of the head and neck in adults. Am J Surg. 1990 Oct;160(4):365-9. doi: 10.1016/s0002-9610(05)80544-6. PMID: 2221235. | 2 |
| 922.Fontanesi J, Pratt C, Kun L, Hustu O, Pao WJ, Douglass E, Fleming I, Rao B. Local-regional non-rhabdomyosarcomatous soft tissue sarcomas of the head and neck. Int J Radiat Oncol Biol Phys. 1990 Oct;19(4):995-9. doi: 10.1016/0360-3016(90)90024-e. PMID: 2211268. | 9 |
| 923.Cheng, J., Yu, H., Wang, L., Wang, X., & Shen, G. (2012). Primary oral and maxillofacial liposarcoma: a clinicopathological and immunohistochemical study of eleven cases. *Archives of Medical Science*, *8*(2), 316-323. | 9 |
| 924.Littman P, Raney B, Zimmerman R, Handler S, Nelson L, Diamond G, Bilaniuk L. Soft-tissue sarcomas of the head and neck in children. Int J Radiat Oncol Biol Phys. 1983 Sep;9(9):1367-71. doi: 10.1016/0360-3016(83)90269-9. PMID: 6309711. | 9 |
| 925.Gasparini M, Lombardi F, Gianni C, Lovati C, Fossati-Bellani F. Childhood rhabdomyosarcoma with meningeal extension: results of combined therapy including central nervous system prophylaxis. Am J Clin Oncol. 1983 Aug;6(4):393-8. PMID: 6869313. | 6 |
| 926.Newman, A. N., & Rice, D. H. (1984). Rhabdomyosarcoma of the head and neck. *The Laryngoscope*, *94*(2), 234-239. | 9 |
| 927.Ou SM. [Soft tissue sarcoma in the head and neck--analysis of 87 patients]. Zhonghua Zhong Liu Za Zhi. 1988 Jul;10(4):289-92. Chinese. PMID: 3248487. | 2 |
| 928.Proops, D. W., & Mann, J. R. (1984). The presentation of rhabdomyosarcomas of the head and neck in children. *The Journal of Laryngology & Otology*, *98*(4), 381-390. | 6 |
| 929.Abemayor, E., & Calcaterra, T. C. (1983). Kaposi's Sarcoma and Community-Acquired Immune Deficiency Syndrome: An Update With Emphasis on Its Head and Neck Manifestations. *Archives of Otolaryngology*, *109*(8), 536-542. | 2 |
| 930.Menggao, Y., Yong, L., Youzhong, L., Yunkai, G., Xinming, Y., Xiangbo, H., ... & Jin, H. (1990). A clinical analysis of 7878 patients with tumors in the head and neck. *Chinese Journal of Cancer Research*, *2*, 59-67. | 1 |
| 931.Anderson, G. J., Tom, L. W., Womer, R. B., Handler, S. D., Wetmore, R. F., & Potsic, W. P. (1990). Rhabdomyosarcoma of the head and neck in children. *Archives of Otolaryngology–Head & Neck Surgery*, *116*(4), 428-431. | 2 |
| 932.Berry, M. P., & Jenkin, R. D. T. (1981). Parameningeal rhabdomyosarcoma in the young. *Cancer*, *48*(2), 281-288. | 9 |
| 933.Blatt, J., Snyderman, C., Wollman, M. R., Mirro Jr, J., Janecka, I. P., Albo, V. C., ... & Wiener, E. S. (1997). Delayed resection in the management of non‐orbital rhabdomyosarcoma of the head and neck in childhood. *Medical and Pediatric Oncology: The Official Journal of SIOP—International Society of Pediatric Oncology (Societé Internationale d'Oncologie Pédiatrique*, *28*(4), 294-298. | 9 |
| 934.Wollina U, Koch A, Hansel G, Schönlebe J, Kittner T, Pabst F, Haroske G, Nowak A. A 10-year analysis of cutaneous mesenchymal tumors (sarcomas and related entities) in a skin cancer center. Int J Dermatol. 2013 Oct;52(10):1189-97. doi: 10.1111/j.1365-4632.2012.05484.x. Epub 2013 Jul 8. PMID: 23829640. | 6 |
| 935.Abdel Rahman H, El-Baradie T, El-Baradie M, Bahaa S, Shalan M. Management Head and Neck Ewing's Sarcoma Family of Tumors: Experience of the National Cancer Institute, Cairo University. J Egypt Natl Canc Inst. 2010 Mar;22(1):41-7. PMID: 21503005. | 2 |
| 936.Fasunla, A. J., & Daniel, A. (2013). Retrospective review of soft tissue sarcoma of head and neck in a West African hospital. *Alexandria Journal of Medicine*, *49*(1), 43-48. | 6 |
| 937.Makino Y. A clinicopathological study on soft tissue tumors of the head and neck. Acta Pathol Jpn. 1979 May;29(3):389-408. doi: 10.1111/j.1440-1827.1979.tb00196.x. PMID: 222115. | 9 |
| 938.Benoit MM, Vargas SO, Bhattacharyya N, McGill TA, Robson CD, Ferraro N, Didas AE, Labow BI, Upton J, Taghinia A, Meara JG, Marcus KJ, Mack J, Rodriguez-Galindo C, Rahbar R. The presentation and management of mandibular tumors in the pediatric population. Laryngoscope. 2013 Aug;123(8):2035-42. doi: 10.1002/lary.24020. Epub 2013 May 13. PMID: 23670306. | 9 |
| 939.DeAngelis AF, Spinou C, Tsui A, Iseli T, Desai J, Wiesenfeld D, Chandu A. Outcomes of patients with maxillofacial osteosarcoma: a review of 15 cases. J Oral Maxillofac Surg. 2012 Mar;70(3):734-9. doi: 10.1016/j.joms.2011.03.020. Epub 2011 Jul 20. PMID: 21778010. | 2 |
| 940.Koka, V., Vericel, R., Lartigau, E., Lusinchi, A., & Schwaab, G. (1994). Sarcomas of nasal cavity and paranasal sinuses: chondrosarcoma, osteosarcoma and fibrosarcoma. *The Journal of Laryngology & Otology*, *108*(11), 947-953. | 2 |
| 941.Sercarz JA, Mark RJ, Tran L, Storper I, Calcaterra TC. Sarcomas of the nasal cavity and paranasal sinuses. Ann Otol Rhinol Laryngol. 1994 Sep;103(9):699-704. doi: 10.1177/000348949410300907. PMID: 8085730. | 2 |
| 942.Cartellieri, M., Lang, S., & Swoboda, H. (1996). Weichteilsarkome im HNO-Bereich. *Laryngo-Rhino-Otologie*, *75*(09), 538-542. | 6 |
| 943..Cartellieri, M., Lang, S., & Swoboda, H. (1996). Weichteilsarkome im HNO-Bereich. *Laryngo-Rhino-Otologie*, *75*(09), 538-542. | 8 |
| 944.Willers, H., Hug, E. B., Spiro, I. J., Efird, J. T., Rosenberg, A. E., & Wang, C. C. (1997). Weichteilsarkome des Kopf-Hals-Bereichs beim Erwachsenen. *Strahlentherapie und Onkologie*, *173*(3), 131. | 2 |
| 945.Cao Yanna, Zhang Guangchao, Yan Jie e Wang Huijuan (2012).Curative Effect and Role of Three-Dimensional Radiotherapy as a Part of Combined Therapy for Pediatric Head-and-Neck | 2 |
| 946.Lim S, Lee S, Rha SY, Rho JK. Cranofacial osteosarcoma: Single institutional experience in Korea. Asia Pac J Clin Oncol. 2016 Mar;12(1):e149-53. doi: 10.1111/ajco.12072. Epub 2013 May 29. PMID: 23718845. | 2 |
| 947.Wang, H. W., Qin, X. J., Yang, W. J., Xu, L. Q., Ji, T., & Zhang, C. P. (2015). Alveolar soft part sarcoma of the oral and maxillofacial region: clinical analysis in a series of 18 patients. *Oral Surgery, Oral Medicine, Oral Pathology and Oral Radiology*, *119*(4), 396-401. | 2 |
| 948.Laskar S, Basu A, Muckaden MA, D'Cruz A, Pai S, Jambhekar N, Tike P, Shrivastava SK. Osteosarcoma of the head and neck region: lessons learned from a single-institution experience of 50 patients. Head Neck. 2008 Aug;30(8):1020-6. doi: 10.1002/hed.20820. PMID: 18383528. | 2 |
| 949.Patow, C. A., Steis, R., Longo, D. L., Reichert, C. M., Findlay, P. A., Potter, D., ... & Macher, A. M. (1984). Kaposi's sarcoma of the head and neck in the acquired immune deficiency syndrome. *Otolaryngology—Head and Neck Surgery*, *92*(3), 255-260. | 9 |
| 950.Mark, R. J., Tran, L. M., Sercarz, J., Fu, Y. S., Calcaterra, T. C., & Parker, R. G. (1993). Chondrosarcoma of the head and neck: The UCLA experience, 1955–1988. *American journal of clinical oncology*, *16*(3), 232-237. | 9 |
| 951.Kamau MW, Chindia ML, Dimba EA, Awange D, Gathece L. Clinico-histopathologic types of maxillofacial malignancies with emphasis on sarcomas: a 10-year review. East Afr Med J. 2011 Feb;88(2):39-45. PMID: 24968590. | 2 |
| 952.Ketabchi A, Kalavrezos N, Newman L. Sarcomas of the head and neck: a 10-year retrospective of 25 patients to evaluate treatment modalities, function and survival. Br J Oral Maxillofac Surg. 2011 Mar;49(2):116-20. doi: 10.1016/j.bjoms.2010.02.012. Epub 2010 Apr 22. PMID: 20416997. | 2 |
| 953.Mark, R. J., Sercarz, J. A., Tran, L., Selch, M., & Calcaterra, T. C. (1991). Fibrosarcoma of the head and neck: the UCLA experience. *Archives of Otolaryngology–Head & Neck Surgery*, *117*(4), 396-401. | 2 |
| 954.Kontio R, Hagström J, Lindholm P, Böhling T, Sampo M, Mesimäki K, Saarilahti K, Koivunen P, Mäkitie AA. Craniomaxillofacial osteosarcoma - The role of surgical margins. J Craniomaxillofac Surg. 2019 Jun;47(6):922-925. doi: 10.1016/j.jcms.2019.03.020. Epub 2019 Mar 25. PMID: 31005379. | 2 |
| 955.Mark, R. J., Sercarz, J. A., Tran, L., Dodd, L. G., Selch, M., & Calcaterra, T. C. (1991). Osteogenic sarcoma of the head and neck: the UCLA experience. *Archives of Otolaryngology–Head & Neck Surgery*, *117*(7), 761-766. | 2 |
| 956.Mark, R. J., Tran, L. M., Sercarz, J., Fu, Y. S., Calcaterra, T. C., & Juillard, G. F. (1993). Angiosarcoma of the head and neck: the UCLA experience 1955 through 1990. *Archives of Otolaryngology–Head & Neck Surgery*, *119*(9), 973-978. | 2 |
| 957.Verdijk, R. M., den Bakker, M. A., Dubbink, H. J., Hop, W. C., Dinjens, W. N., & Kros, J. M. (2010). TP53 mutation analysis of malignant peripheral nerve sheath tumors. *Journal of Neuropathology & Experimental Neurology*, *69*(1), 16-26. | 9 |
| 958.Ha PK, Eisele DW, Frassica FJ, Zahurak ML, McCarthy EF. Osteosarcoma of the head and neck: a review of the Johns Hopkins experience. Laryngoscope. 1999 Jun;109(6):964-9. doi: 10.1097/00005537-199906000-00023. PMID: 10369291. | 2 |
| 959.Markowski J, Dziubdziela W, Podlejska K, Likus W, Pasternak K, Kajor M, Witkowska M, Gierek T, Paluch J. Soft tissues sarcomas of the head and neck in adult: histo-clinical analysis of 30-years material in the data of ENT Department of Silesian Medical University. Otolaryngol Pol. 2012 Nov-Dec;66(6):382-6. doi: 10.1016/j.otpol.2012.06.028. Epub 2012 Jul 6. PMID: 23200557. | 9 |
| 960.Salcedo-Hernández RA, Lino-Silva LS, Luna-Ortiz K. Maxillary Sinus Sarcomas: Epidemiological and Clinicopathological Experience of 25 Years in a National Reference Cancer Center. Indian J Otolaryngol Head Neck Surg. 2014 Dec;66(4):359-64. doi: 10.1007/s12070-012-0522-9. Epub 2012 Feb 22. PMID: 26396944; PMCID: PMC4571464. | 2 |
| 961.Paul AU, Mane SM, Swami SY, Gore A. Clinicopathological Study of Soft Tissue Neoplasms. Cureus. 2025 May 6;17(5):e83576. doi: 10.7759/cureus.83576. PMID: 40476108; PMCID: PMC12139693. | 1 |
| 962.Meazza C, Parisi F, Barretta F, Sironi G, Nigro O, Morosi C, Luksch R, Podda M, Ferrari A, Terenziani M, Spreafico F, Casanova M, Biassoni V, Schiavello E, Parafioriti A, Collini P, Chiaravalli S, Puma N, Bergamaschi L, Gattuso G, Colombo V, Vennarini S, Pecori E, Guzzo M, Colombo S, Trovò A, Massimino M. Pediatric Craniofacial Osteosarcoma: The Milano Experience. Pediatr Blood Cancer. 2025 May 30:e31808. doi: 10.1002/pbc.31808. Epub ahead of print. PMID: 40448365. | 6 |
| 963.Baratz HQ, Yin LX, Moore EJ, Molligan J, Tasche KK, Van Abel K, Price DL. Outcomes and Characteristics of Mesenchymal Tumors Involving the Parotid Gland. Ann Otol Rhinol Laryngol. 2025 May 15:34894251336840. doi: 10.1177/00034894251336840. Epub ahead of print. PMID: 40370050. | 9 |
| 964.Du D, Wu S, Wang Z, Guan Y, Jiang K, Xu B, Liang Y. Novel Location-Grading-Node-Metastasis Staging System in Patients With Head and Neck Soft Tissue Sarcoma. J Otolaryngol Head Neck Surg. 2025 Jan-Dec;54:19160216251333359. doi: 10.1177/19160216251333359. Epub 2025 May 1. PMID: 40310697; PMCID: PMC12049617. | 6 |
| 965.Rohde J, Henssen A, Eggert A, Scheer M. Rhabdomyosarcoma of head and neck varies in aggressiveness depending on the specific site of origin. Oral Oncol. 2025 May;164:107263. doi: 10.1016/j.oraloncology.2025.107263. Epub 2025 Apr 5. PMID: 40188643. | 6 |
| 966.Cha BB, Kim JY, Kim WS, Lee GY, Choi YJ. Relative Tumor Density of Soft-Tissue Sarcoma in Korean Population: An Institutional Review. Ann Dermatol. 2025 Apr;37(2):96-104. doi: 10.5021/ad.23.122. PMID: 40165567; PMCID: PMC11965874. | 6 |
| 967.Clara-Altamirano MA, de Los Santos-Quintanilla AR, Luna-Ortiz K, Alvarez-Cano A, Velazquez-Rodriguez S, Lizcano-Suárez AR, García-Ortega DY. Impact of the Pretreatment Neutrophil/Lymphocyte Ratio as a Prognostic Factor in Conventional Chondrosarcoma. Indian J Surg Oncol. 2025 Feb;16(1):356-363. doi: 10.1007/s13193-024-02100-y. Epub 2024 Sep 23. PMID: 40114908; PMCID: PMC11920533. | 6 |
| 968.Aye JM, Xue W, Gao Z, Ladra M, Indelicato DJ, Sheyn A, Dasgupta R, Arnold MA, Shenoy A, Linardic CM, Venkatramani R. Nonorbital, Nonparameningeal Head and Neck Rhabdomyosarcoma: A Report From the Children's Oncology Group. Pediatr Blood Cancer. 2025 Jun;72(6):e31673. doi: 10.1002/pbc.31673. Epub 2025 Mar 19. PMID: 40108481. | 6 |
| 969.Smith SC, Sweeney K, Evans MG, Angara K, Reynolds C, Price B, Park SJ, Elliott A, Oberley MJ, Boikos SA, Bahrami A. Genomic Profiling Uncovers a Broader Spectrum of Dermatofibrosarcoma Protuberans: Implications for Diagnosis and Therapy. Mod Pathol. 2025 Apr;38(4):100737. doi: 10.1016/j.modpat.2025.100737. Epub 2025 Feb 14. PMID: 39956270. | 6 |
| 970.Histopathologic Spectrum and Clinical Management of Pharyngeal and Laryngeal Sarcomas | 7 |
| 971.Bandora EA, Kampel L, Manisterski M, Elhasid R, Levin D, Horowitz G, Warshavsky A, Wolf R, DeRowe A, Muhanna N. Pediatric head and neck sarcomas: a retrospective study from a national tertiary referral center. Eur J Pediatr. 2025 Feb 1;184(2):169. doi: 10.1007/s00431-025-05991-3. PMID: 39893262. | 6 |
| 972.Sasi A, Ganguly S, Thakar A, Sikka K, Agarwala S, Pushpam D, Kumar A, Biswas B, Meel R, Biswas A, Barwad A, Mridha AR, Bakhshi S. Nuances in the Treatment of Ewing Sarcoma of the Head and Neck in a Low-Middle-Income Country Setting: A Multi-Disciplinary Approach. Head Neck. 2025 Jun;47(6):1690-1698. doi: 10.1002/hed.28086. Epub 2025 Jan 23. PMID: 39844767. | 6 |
| 973.Consensus statement for the diagnosis, treatment, and prognosis of kaposiform hemangioendothelioma | 6 |
| 974.Gallagher KPD, Hunter KD, Arboleda LPA, Pedroso CM, Mariz BALA, Penafort PVM, Souza LL, Rodrigues-Fernandes CI, Tager EMJR, Carlos R, Robinson L, Schouwstra CM, Villanueva-Sánchez FG, Gómez FJP, Del Carmen González-Galván M, Martins-de-Barros AV, de Vasconcelos Carvalho M, Cavalcante RB, Turatti E, Pontes HAR, Siqueira SAC, Mendonça RMH, Innocentini LMAR, de Macedo LD, Ribeiro-Silva A, Abrahão AC, Romañach MJ, van Heerden W, Vargas PA, Santos-Silva AR. Head and Neck Rhabdomyosarcoma in Pediatric Patients: An International Collaborative Study. J Oral Pathol Med. 2025 Feb;54(2):81-90. doi: 10.1111/jop.13600. Epub 2025 Jan 6. PMID: 39763173. | 6 |
| 975.Wang T, Huang C, Wang J, Tang T, Li Q, Li Y, Song X. Multimodality Treatment Outcome in Adult Patients with Head and Neck Rhabdomyosarcoma. Laryngoscope. 2025 Jun;135(6):2022-2029. doi: 10.1002/lary.31968. Epub 2024 Dec 21. PMID: 39707798. | 6 |
| 976.Duan C, He SD, Wang SC, Jin M, Zhao W, Wang XS, Liu ZK, Yu T, He LJ, Wang XM, Cui CY, Ni X, Su Y. [Mid-long term follow-up reports on head and neck rhabdomyosarcoma in children]. Zhonghua Er Ke Za Zhi. 2025 Jan 2;63(1):62-69. Chinese. doi: 10.3760/cma.j.cn112140-20241115-00833. PMID: 39694564. | 6 |
| 977.Patel RR, Gopalakrishnan V, Amini B, Lazar AJ, Lin PP, Benjamin RS, Bishop AJ, Goepfert RP, Araujo DM. Oncologic Outcomes in Patients with Localized, Primary Head and Neck Synovial Sarcoma. Cancers (Basel). 2024 Dec 9;16(23):4119. doi: 10.3390/cancers16234119. PMID: 39682304; PMCID: PMC11639992. | 6 |
| 978.Wu JF, Ding XC, Zhang XJ, Cui M, Wang JH, Fan J, An CM, Du W, Liu ST. [Clinical characteristics of undifferentiated pleomorphic sarcoma from the head and neck]. Zhonghua Yi Xue Za Zhi. 2024 Dec 17;104(47):4330-4334. Chinese. doi: 10.3760/cma.j.cn112137-20240808-01822. PMID: 39667771. | 6 |
| 979.Wang S, Wang Y, Xu J, Ren Q, Hu Y, Jia L, Wang X. Ultrasound characteristics of alveolar soft part sarcoma in pediatric patients: a retrospective analysis. BMC Cancer. 2024 Dec 2;24(1):1484. doi: 10.1186/s12885-024-13262-x. PMID: 39623317; PMCID: PMC11613896. | 6 |
| 980.Kelany MR, Abd Eltawab AA, Mohamed MN, Bayomy MF, Soliman DAM. Clinico-epidemiological and treatment factors impact on survival in Egyptian patients with head and neck sarcoma: a retrospective case-series analysis. J Egypt Natl Canc Inst. 2024 Nov 25;36(1):37. doi: 10.1186/s43046-024-00242-2. PMID: 39581939. | 6 |
| 981.Schoot RA, Taselaar P, Scarzello G, Kolb F, Coppadoro B, Horst ST, Mandeville H, Ferrari A, Hladun R, Helfre S, Ferman S, Kelsey A, Hol MLF, Devalck C, Ben-Arush M, Orbach D, Chisholm J, Jenney M, Minard-Colin V, Bisogno G, Merks JHM. Parameningeal Rhabdomyosarcoma: Results of the European Pediatric Soft Tissue Sarcoma Study Group RMS 2005 Study. Head Neck. 2025 Mar;47(3):974-982. doi: 10.1002/hed.27994. Epub 2024 Nov 15. PMID: 39545397; PMCID: PMC11816552. | 6 |
| 982.Bielack SS, Mettmann V, Hecker-Nolting S, Borkhardt A, Hardes J, Kager L, von Kalle T, Kevric M, Koscielniak E, Kratz CP, Kühne T, Nathrath M, Rossig C, Sorg B, Sparber-Sauer M, Werner M, Blattmann C. Osteosarcoma as a secondary malignancy following rhabdomyosarcoma: A report of 28 affected patients from the Cooperative Osteosarcoma Study Group (COSS). Pediatr Blood Cancer. 2024 Dec;71(12):e31344. doi: 10.1002/pbc.31344. Epub 2024 Sep 29. PMID: 39344062. | 6 |
| 983.Souza BDAF, Maglia DR, de Lima TB, da Silveira HLD, Visioli F. Systemic sequelae and craniofacial development in survivors of pediatric rhabdomyosarcoma. J Stomatol Oral Maxillofac Surg. 2025 Feb;126(1):102024. doi: 10.1016/j.jormas.2024.102024. Epub 2024 Aug 25. PMID: 39191300. | 6 |
| 984.Sun Y, Yilala MH, Musumano LB, Yang J, Sanna M. Surgical management of chondrosarcomas of the skull-base and temporal bone. Eur Arch Otorhinolaryngol. 2024 Dec;281(12):6339-6352. doi: 10.1007/s00405-024-08864-x. Epub 2024 Jul 31. PMID: 39085472. | 5 |
| 985.Patel RR, Delclos GL, DeSantis SM, Cannell MB, Lupo PJ, Lin PP, Araujo DM. Epidemiological trends of synovial sarcoma by primary tumor sites in the US from 2000 to 2020. Cancer Epidemiol. 2024 Oct;92:102627. doi: 10.1016/j.canep.2024.102627. Epub 2024 Jul 23. PMID: 39048411. | 6 |
| 986.Assadi A, Berjis N, Motamedi N, Eshaghian A. Epidemiological Study of Head and Neck Sarcomas. Iran J Otorhinolaryngol. 2024 Jul;36(4):537-543. doi: 10.22038/IJORL.2024.75749.3535. PMID: 39015687; PMCID: PMC11247447. | 6 |
| 987.Wang T, Wang J, Li Q, Li Y, Song X. Incidence and patterns of lymph node metastases in head and neck rhabdomyosarcoma: One-institution study. Head Neck. 2024 Dec;46(12):3001-3012. doi: 10.1002/hed.27870. Epub 2024 Jul 14. PMID: 39004953. | 6 |
| 988.Maghfour J, Genelin X, Olson J, Wang A, Schultz L, Blalock TW. The epidemiology of dermatofibrosarcoma protuberans incidence, metastasis, and death among various population groups: A Surveillance, Epidemiology, and End Results database analysis. J Am Acad Dermatol. 2024 Nov;91(5):826-833. doi: 10.1016/j.jaad.2024.05.088. Epub 2024 Jun 21. PMID: 38908718. | 6 |
| 989.Xie S, Zhang J, Qin Y, Li M, Lan G, Wang Y, Huang B, Weng J, Wei J, Qu S. [Radiation-induced sarcoma in patients with nasopharyngeal carcinoma: a single-institution retrospective study]. Lin Chuang Er Bi Yan Hou Tou Jing Wai Ke Za Zhi. 2024 Jun;38(6):514-517. Chinese. doi: 10.13201/j.issn.2096-7993.2024.06.011. PMID: 38858117; PMCID: PMC11480581. | 6 |
| 990.Yoder AK, Farooqi A, Mitra D, Livingston JA, Araujo DM, Sturgis EM, Goepfert R, Bishop AJ, Guadagnolo BA. Outcomes for Patients With Head and Neck Sarcoma Treated Curatively With Radiation Therapy and Surgery. Pract Radiat Oncol. 2024 Sep-Oct;14(5):e373-e382. doi: 10.1016/j.prro.2024.05.006. Epub 2024 Jun 6. PMID: 38851534; PMCID: PMC11622145. | 6 |
| 991.Chen Y, Gokavarapu S, Shen Q, Gao X, Ren Z, Ji T. Head and Neck Osteosarcoma: Perineural Invasion is Associated With Disease-Free Survival and Tumor Metastasis. J Oral Maxillofac Surg. 2024 Aug;82(8):992-998. doi: 10.1016/j.joms.2024.05.001. Epub 2024 May 7. PMID: 38797510. | 6 |
| 992.Muto Y, Fujimura T, Takahashi A, Namikawa K, Ogata D, Nakano E, Jinnai S, Hashimoto A, Kambayashi Y, Asano Y, Yamazaki N. Analysis of surgical margins and prognostic factors in dermatofibrosarcoma protuberans after wide local excision: A multicenter study of 116 Japanese patients. J Dermatol. 2024 Sep;51(9):1225-1232. doi: 10.1111/1346-8138.17280. Epub 2024 May 22. PMID: 38775205. | 6 |
| 993.Hikmet RG, Klug TE, Gade SD, Sandfeld-Paulsen B, Aggerholm-Pedersen N. A Retrospective Study of 291 Patients With Head and Neck Sarcomas: Treatment, Outcomes, and Prognostic Factors. Clin Oncol (R Coll Radiol). 2024 Jul;36(7):409-419. doi: 10.1016/j.clon.2024.04.009. Epub 2024 Apr 27. PMID: 38744596. | 6 |
| 994.Bini A, Derka S, Stavrianos S. Management of head & neck sarcomas in adults: A retrospective study. J Craniomaxillofac Surg. 2024 Aug;52(8):877-883. doi: 10.1016/j.jcms.2024.01.005. Epub 2024 Jan 11. PMID: 38443189. | 2 |
| 995.Ike E, Mai JZ, Sargen MR, Schonfeld SJ, Cahoon EK. Ambient UV radiation is associated with cutaneous angiosarcoma incidence in the United States, 1992 to 2020. J Am Acad Dermatol. 2024 Jul;91(1):102-104. doi: 10.1016/j.jaad.2024.01.084. Epub 2024 Mar 2. PMID: 38432461; PMCID: PMC11193604. | 4 |
| 996.Gangadharan J, Mathews A, Prasanna Kumary SN, Somanathan T, Jayasree K, Narayanan G. Angiosarcoma of the head and neck: A clinicopathologic study with special emphasis on diagnostic pitfalls. Indian J Pathol Microbiol. 2024 Jul 1;67(3):559-563. doi: 10.4103/ijpm.ijpm_655_22. Epub 2024 Feb 14. PMID: 38391320. | 6 |
| 997.Marcoval J, Moreno-Vílchez C, Torrecilla-Vall-Llosera C, Muntaner-Virgili C, Pérez Sidelnikova D, Sanjuán X, Penín RM. Dermatofibrosarcoma Protuberans: A Study of 148 Patients. Dermatology. 2024;240(3):487-493. doi: 10.1159/000536172. Epub 2024 Jan 16. PMID: 38228098; PMCID: PMC11168446. | 6 |
| 998.Odate T, Satomi K, Kubo T, Matsushita Y, Ueno T, Kurose A, Shomori K, Nakai T, Watanabe R, Segawa K, Ohshika S, Miyake N, Kudo S, Shimoi T, Kobayashi E, Komiyama M, Yoshimoto S, Nakatani F, Kawai A, Yatabe Y, Kohsaka S, Ichimura K, Ichikawa H, Yoshida A. Inflammatory Rhabdomyoblastic Tumor: Clinicopathologic and Molecular Analysis of 13 Cases. Mod Pathol. 2024 Jan;37(1):100359. doi: 10.1016/j.modpat.2023.100359. Epub 2023 Oct 21. PMID: 37871654. | 9 |
| 999.Xu B, Qiu H, Ou B, Chen S, Du D, Liu Y, Zhang X, Liang Y. The inflammatory-nutritional score and nomogram for R0 resected head and neck soft tissue sarcoma. Oral Dis. 2024 Apr;30(3):1139-1151. doi: 10.1111/odi.14499. Epub 2023 Jan 20. PMID: 36630573. | 6 |
| 1000.Dai Z, He Y, Zhang X, Tian Z, Zhu G, Ren Z, Ye L, Liu Z, Ma C, Cao W, Ji T. Head-and-neck dermatofibrosarcoma protuberans: Survival analysis and Clinically relevant immunohistochemical indicators. Oral Dis. 2024 Apr;30(3):1040-1051. doi: 10.1111/odi.14495. Epub 2023 Feb 3. PMID: 36597156. | 6 |
| 1001.Shabaan A, Alkashash A, Hou T, Saeed O, Yesensky J, Roshal A, Mesa H. Isolated Kaposi sarcoma of the upper aerodigestive tract in immunocompetent individuals, an underrecognized entity with favorable prognosis. Ear Nose Throat J. 2025 Mar;104(1_suppl):165S-169S. doi: 10.1177/01455613221128112. Epub 2022 Sep 15. PMID: 38124324. | 4 |
| 1002.Qian Shi, Wang R, Hou L, Huang Z, Ma H, Zhang Y, Zhong Q, Feng L, He S, Chen X, Li P, Yang Y, Li Y, Zhang L, Fang J. Prognostic Analysis of Individualized Treatments of Malignant Tumors Primary From Maxillary Sinus. Ear Nose Throat J. 2024 Mar;103(3):173-182. doi: 10.1177/01455613221115134. Epub 2022 Aug 6. PMID: 35938483. | 1 |
| 1003.de Souza LL, Correia-Neto IJ, Kalinin Y, Pontes FSC, Santos-Silva AR, Vargas PA, Lopes MA, Pontes HAR. Oral Kaposi sarcoma in Brazil: a case series of 40 patients highlighting a significant health burden. Oral Surg Oral Med Oral Pathol Oral Radiol. 2025 May;139(5):576-582. doi: 10.1016/j.oooo.2024.12.014. Epub 2024 Dec 19. PMID: 39779389. | 2 |
| 1004.Bae MR, Lee YH, Kim JH, Chung YS, Kim JH, Yu MS. Clinical Features and Prognostic Insights in Sinonasal Sarcomas: A 76-Case Single-Institution Experience. Laryngoscope Investig Otolaryngol. 2025 Apr 22;10(2):e70145. doi: 10.1002/lio2.70145. PMID: 40264921; PMCID: PMC12012637. | 5 |
| 1005.Kobayashi K, Yoshimoto S, Yamamura K, Kitayama M, Kawakita D, Nibu KI, Kondo K, Saito Y. Histological Type-Specific Behavior in Sarcomas: Analysis of Head and Neck Cancer Registry of Japan. Laryngoscope. 2025 Jan 27. doi: 10.1002/lary.32027. Epub ahead of print. PMID: 39868641. | 5 |
| 1006.Shaker N, Mansoori P, Fattah YH, Ellis M, Sexton T, O'Neill S, Qasem SA. P16 and HPV status in head and neck sarcomas and sarcomatoid carcinomas. Ann Diagn Pathol. 2024 Aug;71:152307. doi: 10.1016/j.anndiagpath.2024.152307. Epub 2024 Apr 10. PMID: 38626591. | 2 |
| 1007.Markowski J, Długosz-Karbowska A, Ciupińska M, Smółka W, Dobrosz Z, Ślaska-Kaspera A, Lesniewska-Skowerska O, Likus W, Mazurek K. Soft tissue sarcomas of the head and neck region: clinical and histopathological study of 39 patients. Otolaryngol Pol. 2024 Jul 25;78(4):21-28. PMID: 39051643. | 2 |

1. Studies that did not investigate the clinicopathological profile of oral and maxillofacial sarcoma;
2. Studies with insufficient clinicopathological information;
3. Studies that did not use histopathology as the reference standard for diagnosis;
4. Reviews, case reports, protocols, short communication, personal opinions, letter, conference abstracts, laboratory research, book chapter and in vitro or in vivo studies;
5. The diagnosis is not a primary oral and maxillofacial sarcoma;
6. Studies that included cases from other anatomic sites combined with oral and maxillofacial sarcomas, where data were aggregated and could not be separated
7. Studies whose full texts were not available;
8. Duplicated sample;
9. Sample number.

**Table S3** Excluded articles and reasons for exclusion – Grey literature (n=42)

| References | Reasons for exclusion |
| --- | --- |
| 1. Sofa, M., Soemitro, M. P., Abdurahman, M., Azhar, Y., & Rizky, K. A. (2023). Head and Neck Sarcomas Characteristics at Dr. Hasan Sadikin General Hospital for 5 Years (2017-2021). *Bioscientia Medicina: Journal of Biomedicine and Translational Research*, *7*(8), 3533-3537. | 9 |
| 1. AlOtaibi, M. N., Basfar, A. S., Jawhari, A. M., Alzahrani, E. S., Althomali, M. A., Alhindi, A. E., ... & Abdel-Moneim, A. S. (2023). The Burden of Skin Cancers in Saudi Arabia Through 2011-2022. *Cureus*, *15*(9). | 2 |
| 1. Jawad, M. U., Zeitlinger, L. N., Bewley, A. F., O’Donnell III, E. F., Traven, S. A., Carr-Ascher, J. R., ... & Randall, R. L. (2022). Head and neck cutaneous soft-tissue sarcoma demonstrate sex and racial/ethnic disparities in incidence and socioeconomic disparities in survival. *Journal of Clinical Medicine*, *11*(18), 5475. | 2 |
| 1. Rouhani P, Fletcher CD, Devesa SS, Toro JR. Cutaneous soft tissue sarcoma incidence patterns in the U.S. : an analysis of 12,114 cases. Cancer. 2008 Aug 1;113(3):616-27. doi: 10.1002/cncr.23571. PMID: 18618615. | 2 |
| 1. Gosepath, J., Spix, C., Talebloo, B., Blettner, M., & Mann, W. J. (2007). Incidence of childhood cancer of the head and neck in Germany. *Annals of oncology*, *18*(10), 1716-1721. | 1 |
| 1. Davies, L., & Welch, H. G. (2006). Epidemiology of head and neck cancer in the United States. *Otolaryngology—Head and Neck Surgery*, *135*(3), 451-457. | 1 |
| 1. Penel, N., Van Haverbeke, C., Lartigau, E., Vilain, M. O., Van, J. T., Mallet, Y., & Lefebvre, J. L. (2004). Head and neck soft tissue sarcomas of adult: prognostic value of surgery in multimodal therapeutic approach. *Oral oncology*, *40*(9), 890-897. | 2 |
| 1. O’Sullivan, B., Gullane, P., Irish, J., Neligan, P., Gentili, F., Mahoney, J., ... & Bell, R. (2003). Preoperative radiotherapy for adult head and neck soft tissue sarcoma: assessment of wound complication rates and cancer outcome in a prospective series. *World journal of surgery*, *27*, 875-883. | 2 |
| 1. Dudhat, S. B., Mistry, R. C., Varughese, T., Fakih, A. R., & Chinoy, R. F. (2000). Prognostic factors in head and neck soft tissue sarcomas. *Cancer: Interdisciplinary International Journal of the American Cancer Society*, *89*(4), 868-872. | 2 |
| 1. Hashimoto, H. (1995). Incidence of soft tissue sarcomas in adults. *Soft Tissue Tumors*, 1-16. | 4 |
| 1. Mark RJ, Bailet JW, Poen J, Tran LM, Calcaterra TC, Abemayor E, Fu YS, Parker RG. Postirradiation sarcoma of the head and neck. Cancer. 1993 Aug 1;72(3):887-93. doi: 10.1002/1097-0142(19930801)72:3<887::aid-cncr2820720338>3.0.co;2-5. PMID: 8334642. | 9 |
| 1. Weber RS, Benjamin RS, Peters LJ, Ro JY, Achon O, Goepfert H. Soft tissue sarcomas of the head and neck in adolescents and adults. Am J Surg. 1986 Oct;152(4):386-92. doi: 10.1016/0002-9610(86)90309-0. PMID: 3766868. | 2 |
| 1. Xu, Y., Xu, G., Liu, Z. *et al.* Incidence and prognosis of distant metastasis in malignant peripheral nerve sheath tumors. *Acta Neurochir* 163 , 521–529 (2021). https://doi.org/10.1007/s00701-020-04647-5 | 2 |
| 1. Snow A, Ring A, Struycken L, Mack W, Koç M, Lang JE. Incidence of radiation induced sarcoma attributable to radiotherapy in adults: A retrospective cohort study in the SEER cancer registries across 17 primary tumor sites. Cancer Epidemiol. 2021 Feb;70:101857. doi: 10.1016/j.canep.2020.101857. Epub 2020 Nov 26. PMID: 33249363; PMCID: PMC7856279. | 9 |
| 1. Smartt AA, Jang ES, Tyler WK. Is There an Association Between Insurance Status and Survival and Treatment of Primary Bone and Extremity Soft-tissue Sarcomas? A SEER Database Study. Clin Orthop Relat Res. 2020 Mar;478(3):527-536. doi: 10.1097/CORR.0000000000000889. PMID: 31390340; PMCID: PMC7145069. | 1 |
| 1. Han S, Yin X, Xu W, Wang Y, Han W. The Management of Head and Neck Sarcoma. J Craniofac Surg. 2020 Mar/Apr;31(2):e189-e192. doi: 10.1097/SCS.0000000000006162. PMID: 31934973. | 2 |
| 1. Fabiano, S., Contiero, P., Barigelletti, G., D’Agostino, A., Tittarelli, A., Mangone, L., ... & Tagliabue, G. (2020). Epidemiology of Soft Tissue Sarcoma and Bone Sarcoma inItaly: Analysis of Data from 15 Population‐Based Cancer Registries. *Sarcoma*, *2020*(1), 6142613. | 2 |
| 1. Wright CM, Halkett G, Carey Smith R, Moorin R. Sarcoma epidemiology and cancer-related hospitalisation in Western Australia from 1982 to 2016: a descriptive study using linked administrative data. BMC Cancer. 2020 Jul 6;20(1):625. doi: 10.1186/s12885-020-07103-w. PMID: 32631311; PMCID: PMC7336405. | 1 |
| 1. Basse C, Italiano A, Penel N, Mir O, Chemin C, Toulmonde M, Duffaud F, Le Cesne A, Chevreau C, Maynou C, Anract P, Gouin F, Rios M, Firmin N, Kurtz JE, Kerbrat P, Piperno-Neumann S, Bertucci F, Rosset P, Isambert N, Bompas E, Dubray-Longeras P, Fiorenza F, Le Maignan C, Chaigneau L, Thyss A, Bouché O, Eymard JC, Delcambre Lair C, Adam J, Karanian M, Lebbé C, Dupré A, Meeus P, Brahmi M, Dufresne A, Ducimetière F, Ray-Coquard I, Blay JY. Sarcomas in patients over 90: Natural history and treatment-A nationwide study over 6 years. Int J Cancer. 2019 Oct 15;145(8):2135-2143. doi: 10.1002/ijc.32307. Epub 2019 Apr 15. PMID: 30924137; PMCID: PMC6767526. | 2 |
| 1. Clinicopathological Study of Soft Tissue Tumors E., Murali Krishna.   Rajiv Gandhi University of Health Sciences (India) ProQuest Dissertations & Theses,  2019. 30585618. | 9 |
| 1. Joseph, N., St. Laurent, S., Nelson, J. J., Zheng, S., & Stirnadel-Farrant, H. (2019). Incidence and prevalence of synovial sarcoma in the US: An analysis using SEER* Stat. | 4 |
| 1. Grønhøj, C., Hjalgrim, L., Jakobsen, K. K., Charabi, B., Mirian, C., Laier, G. H., ... & Hjuler, T. (2018). Incidence of head and neck cancer in children: A Danish nationwide study from 1978 to 2014. *Pediatric Blood & Cancer*, *65*(7), e27037. | 1 |
| 1. Passos, H., Egal, E., Shinomia, S., Gabriel, A., Cintra, M. L., Amstalden, E., ... & Mariano, F. (2018). 76 Retrospective Study of Sarcomas in a Brazilian Institution: Clinical-Pathological Analysis. *American Journal of Clinical Pathology*, *149*, S33. | 4 |
| 1. Ferrari A, Sultan I, Huang TT, Rodriguez-Galindo C, Shehadeh A, Meazza C, Ness KK, Casanova M, Spunt SL. Soft tissue sarcoma across the age spectrum: a population-based study from the Surveillance Epidemiology and End Results database. Pediatr Blood Cancer. 2011 Dec 1;57(6):943-9. doi: 10.1002/pbc.23252. Epub 2011 Jul 25. PMID: 21793180; PMCID: PMC4261144. | 2 |
| 1. Turner JH, Richmon JD. Head and neck rhabdomyosarcoma: a critical analysis of population-based incidence and survival data. Otolaryngol Head Neck Surg. 2011 Dec;145(6):967-73. doi: 10.1177/0194599811417063. Epub 2011 Aug 26. PMID: 21873599. | 2 |
| 1. Sidappa, K. T., & Krishnamurthy, A. (2011). Adult soft-tissue sarcomas of the head and neck. *Indian Journal of Cancer*, *48*(3), 284-288. | 2 |
| 1. González-González R, Bologna-Molina R, Molina-Frechero N, Domínguez-Malagon HR. Prognostic factors and treatment strategies for adult head and neck soft tissue sarcoma. Int J Oral Maxillofac Surg. 2012 May;41(5):569-75. doi: 10.1016/j.ijom.2012.02.002. Epub 2012 Mar 6. PMID: 22398019. | 2 |
| 1. Maretty-Nielsen K, Aggerholm-Pedersen N, Safwat A, Baerentzen S, Pedersen AB, Keller J. Prevalence and prognostic impact of comorbidity in soft tissue sarcoma: a population-based cohort study. Acta Oncol. 2014 Sep;53(9):1188-96. doi: 10.3109/0284186X.2014.888494. Epub 2014 Mar 3. PMID: 24588412. | 2 |
| 1. Hsieh MC, Wu XC, Andrews PA, Chen VW. Racial and Ethnic Disparities in the Incidence and Trends of Soft Tissue Sarcoma Among Adolescents and Young Adults in the United States, 1995-2008. J Adolesc Young Adult Oncol. 2013 Sep;2(3):89-94. doi: 10.1089/jayao.2012.0031. PMID: 24066270; PMCID: PMC3778995. | 1 |
| 1. Stiller CA, Trama A, Serraino D, Rossi S, Navarro C, Chirlaque MD, Casali PG; RARECARE Working Group. Descriptive epidemiology of sarcomas in Europe: report from the RARECARE project. Eur J Cancer. 2013 Feb;49(3):684-95. doi: 10.1016/j.ejca.2012.09.011. Epub 2012 Oct 15. PMID: 23079473. | 1 |
| 1. Mattavelli D, Miceli R, Radaelli S, Mattavelli F, Cantù G, Barisella M, Quattrone P, Stacchiotti S, Sangalli C, Casali PG, Gronchi A, Fiore M. Head and neck soft tissue sarcomas: prognostic factors and outcome in a series of patients treated at a single institution. Ann Oncol. 2013 Aug;24(8):2181-9. doi: 10.1093/annonc/mdt126. Epub 2013 Apr 5. PMID: 23562930. | 2 |
| 1. Brady JS, Chung SY, Marchiano E, Eloy JA, Baredes S, Park RCW. Pediatric head and neck bone sarcomas: An analysis of 204 cases. Int J Pediatr Otorhinolaryngol. 2017 Sep;100:71-76. doi: 10.1016/j.ijporl.2017.06.003. Epub 2017 Jun 15. PMID: 28802390. | 2 |
| 1. Gerry D, Fox NF, Spruill LS, Lentsch EJ. Liposarcoma of the head and neck: analysis of 318 cases with comparison to non-head and neck sites. Head Neck. 2014 Mar;36(3):393-400. doi: 10.1002/hed.23311. Epub 2013 Jun 1. PMID: 23728920. | 2 |
| 1. Chao AH, Sturgis EM, Yu P, Skoracki RJ, Guadagnolo BA, Hanasono MM. Reconstructive outcomes in patients with head and neck sarcoma. Head Neck. 2013 May;35(5):677-83. doi: 10.1002/hed.23014. Epub 2012 Jun 19. PMID: 22711660. | 1 |
| 1. Piñeiro Aguín Z, León Vintró X, García Lorenzo J, Sancho FJ, López Pousa A, Quer Agustí M. Sarcomas de cabeza y cuello. Nuestra experiencia [Head and neck sarcomas. Our experience]. Acta Otorrinolaringol Esp. 2011 Nov-Dec;62(6):436-42. Spanish. doi: 10.1016/j.otorri.2011.05.005. Epub 2011 Aug 5. PMID: 21820641. | 9 |
| 1. Mücke T, Mitchell DA, Tannapfel A, Hölzle F, Kesting MR, Wolff KD, Kolk A, Kanatas A. Outcome in adult patients with head and neck sarcomas--a 10-year analysis. J Surg Oncol. 2010 Aug 1;102(2):170-4. doi: 10.1002/jso.21595. PMID: 20648589. | 2 |
| 1. Aytekin MN, Öztürk R, Amer K, Yapar A. Epidemiology, incidence, and survival of synovial sarcoma subtypes: SEER database analysis. Journal of Orthopaedic Surgery. 2020;28(2). doi:10.1177/2309499020936009 | 2 |
| 1. Windfuhr, J. P. (2004). Primitive neuroectodermal tumor of the head and neck: incidence, diagnosis, and management. *Annals of Otology, Rhinology & Laryngology*, *113*(7), 533-543. | 2 |
| 1. Patel SC, Silbergleit R, Talati SJ. Sarcomas of the head and neck. Top Magn Reson Imaging. 1999 Dec;10(6):362-75. doi: 10.1097/00002142-199912000-00003. PMID: 10643880. | 9 |
| 1. Gorsky, M., & Epstein, J. B. (1998). Head and neck and intra-oral soft tissue sarcomas. *Oral Oncology*, *34*(4), 292-296. | 2 |
| 41.Pandey M, Thomas G, Mathew A, Abraham EK, Somanathan T, Ramadas K, Iype EM, Ahamed IM, Sebastian P, Nair MK. Sarcoma of the oral and maxillofacial soft tissue in adults. Eur J Surg Oncol. 2000 Mar;26(2):145-8. doi: 10.1053/ejso.1999.0758. PMID: 10744932. | 9 |
| 42.Okechi, U. C., Akpeh, J. O., Odoh, E. O., Obi, D. I., Iwuchukwu, O. C., & Iwuchukwu, A. O. (2022). Challenges in the management of head and neck sarcomas in a resource scarce setting: A review of 54 cases. *Advances in Oral and Maxillofacial Surgery*, *7*, 100316. | 2 |

1. Studies that did not investigate the clinicopathological profile of oral and maxillofacial sarcoma;
2. Studies with insufficient clinicopathological information;
3. Studies that did not use histopathology as the reference standard for diagnosis;
4. Reviews, case reports, protocols, short communication, personal opinions, letter, conference abstracts, laboratory research, book chapter and in vitro or in vivo studies;
5. The diagnosis is not a primary oral and maxillofacial sarcoma;
6. Studies that included cases from other anatomic sites combined with oral and maxillofacial sarcomas, where data were aggregated and could not be separated;
7. Studies whose full texts were not available;
8. Duplicated sample;
9. Sample number.

**Figure S1** Flow diagram of literature search and selection criteria adapted from PRISMA.

**
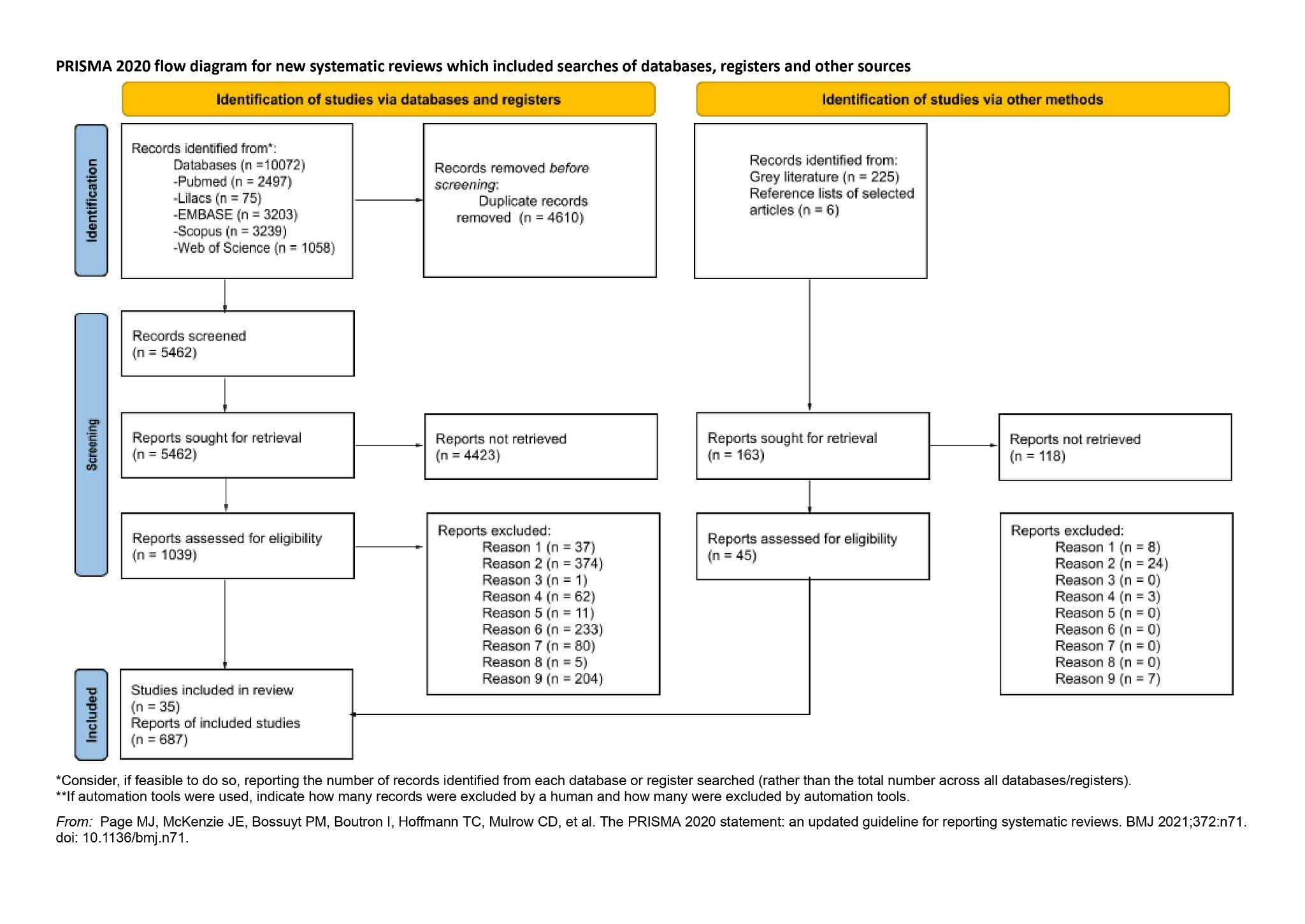
**

**Table S4.** Demographic and clinicopathological characteristics of the 35 studies (687 cases) of oral and maxillofacial sarcomas included in the systematic review.

| **Author/ Year** | **C** | **N** | **Age (y)** | **Sex** | **Site** | **Conditions/ comorbidities of patients** | **Clinical apperance and symtoms** | **Histological Type** | **Molecular alteration** | **T** | **N** | **M** | **Stage** | **Other stage** | **Treatment** | **Margin** | **LR** | **NM** | **DM** | **Status** | **Follow-up (m)** |
| --- | --- | --- | --- | --- | --- | --- | --- | --- | --- | --- | --- | --- | --- | --- | --- | --- | --- | --- | --- | --- | --- |
| Shmookler et al. 1982 | USA | 10 | 27 | F | Cheek | NI | Gradually enlarging mass 4 Gradually enlarging mass associated pain or tenderness 3  Cervical lymphadenopathy 1  Distinct polypoid shape 1  Pediclelike surface attachment 1  Exophytic mass 1  Hemoptysis and respiratory stridor 1 | Synovial Sarcoma | NI | NI | NI | NI | NI | NI | S | NI | No | NI | NI | NED | 15.6 |
|  |  |  | 35 | F | Facial Buccal pad | NI |  | Synovial Sarcoma | NI | NI | NI | NI | NI | NI | S | NI | No | NI | NI | NED | 48 |
|  |  |  | 36 | M | Cheek | NI |  | Synovial Sarcoma | NI | NI | NI | NI | NI | NI | S+RT+CT | NI | Yes | NI | NI | Dead | 25.2 |
|  |  |  | 26 | M | Cheek | NI |  | Synovial Sarcoma | NI | NI | NI | NI | NI | NI | S+RT+CT | NI | Yes | NI | NI | Dead | 31.2 |
|  |  |  | 19 | M | Parotid region | NI |  | Synovial Sarcoma | NI | NI | NI | NI | NI | NI | S+RT+CT | NI | Yes | NI | NI | Dead | 34.8 |
|  |  |  | 49 | M | Parotid region | NI |  | Synovial Sarcoma | NI | NI | NI | NI | NI | NI | S+RT | NI | No | NI | NI | NED | 27.6 |
|  |  |  | 36 | M | Submental | NI |  | Synovial Sarcoma | NI | NI | NI | NI | NI | NI | NI | NI | NI | NI | NI | NI | NI |
|  |  |  | 35 | M | Tonsil | NI |  | Synovial Sarcoma | NI | NI | NI | NI | NI | NI | NI | NI | NI | NI | NI | NI | NI |
|  |  |  | 34 | M | Tonsil | NI |  | Synovial Sarcoma | NI | NI | NI | NI | NI | NI | S+RT | NI | No | NI | NI | NED | 36 |
|  |  |  | 16 | M | Tongue | NI |  | Synovial Sarcoma | NI | NI | NI | NI | NI | NI | S | NI | No | NI | NI | NED | 96 |
| Finn et al. 1984 | USA | 10 | 48 | F | Maxilla | NI | NI | Chondrosarcoma | NI | NI | NI | NI | NI | NI | S | NI | NI | NI | NI | NED | 348 |
|  |  |  | 47 | M | Nasal cavity | NI | NI | Chondrosarcoma | NI | NI | NI | NI | NI | NI | S | NI | NI | NI | NI | NED | 240 |
|  |  |  | 49 | F | Mandible | NI | NI | Chondrosarcoma | NI | NI | NI | NI | NI | NI | S | NI | NI | NI | NI | NED | 168 |
|  |  |  | 44 | F | Palate | NI | NI | Chondrosarcoma | NI | NI | NI | NI | NI | NI | S | NI | NI | NI | NI | NED | 72 |
|  |  |  | 71 | F | Maxilla | NI | NI | Chondrosarcoma | NI | NI | NI | NI | NI | NI | S | NI | NI | NI | NI | NED | 132 |
|  |  |  | 40 | M | Nasal septum | NI | NI | Chondrosarcoma | NI | NI | NI | NI | NI | NI | S | NI | NI | NI | NI | NED | 36 |
|  |  |  | 50 | M | Maxilla | NI | NI | Chondrosarcoma | NI | NI | NI | NI | NI | NI | S | NI | NI | NI | NI | NED | 6 |
|  |  |  | 34 | F | Mandible | NI | NI | Chondrosarcoma | NI | NI | NI | NI | NI | NI | S | NI | NI | NI | NI | Dead | 12 |
|  |  |  | 58 | M | Mandible | NI | NI | Chondrosarcoma | NI | NI | NI | NI | NI | NI | None | NI | NI | NI | NI | Dead | 4 |
|  |  |  | 23 | F | Maxilla | NI | NI | Chondrosarcoma | NI | NI | NI | NI | NI | NI | S+RT | NI | NI | NI | NI | NED | 24 |
| Fernandez Sanroman et al. 1982 | Spain | 13 | 12 | M | Maxilla | NI | Swelling, pain and tooth mobility | Leiomyosarcoma | NI | NI | NI | NI | NI | NI | S+RT | Negative | NI | NI | NI | NED | 24 |
|  |  |  | 17 | M | Mandible | NI | Swelling and pain | Osteosarcoma | NI | NI | NI | NI | NI | NI | S+CT | Positive | Yes | NI | Yes (Lung) | DOD | 6 |
|  |  |  | 84 | M | Maxilla | NI | Swelling and ingival bleeding | Osteosarcoma | NI | NI | NI | NI | NI | NI | RT | NI | NI | NI | NI | DOD | 5 |
|  |  |  | 59 | M | Mandible | NI | Swelling and Pain | Undifferentiated pleomorphic sarcoma | NI | NI | NI | NI | NI | NI | S+RT | Positive | Yes | NI | NI | DOD | 6 |
|  |  |  | 43 | F | Maxilla | NI | Swelling | Osteosarcoma | NI | NI | NI | NI | NI | NI | S+RT+CT | Negative | Yes | NI | NI | DOD | 24 |
|  |  |  | 60 | M | Cheek | NI | Swelling | Undifferentiated pleomorphic sarcoma | NI | NI | NI | NI | NI | NI | S+RT | Negative | Yes | NI | Yes (Lung) | DOD | 36 |
|  |  |  | 33 | F | Chin | NI | Swelling | Undifferentiated pleomorphic sarcoma | NI | NI | NI | NI | NI | NI | S | Negative | Yes | NI | NI | DOC | 120 |
|  |  |  | 16 | F | Mandible | NI | Swelling, pain and tooth mobility | Chondrosarcoma | NI | NI | NI | NI | NI | NI | S+RT | Positive | Yes | NI | Yes (Brain) | DOD | 13 |
|  |  |  | 20 | F | Mandible | NI | Swelling | Fibrosarcoma | NI | NI | NI | NI | NI | NI | S+RT+CT | Negative | NI | NI | NI | NED | 60 |
|  |  |  | 22 | M | Mandible | NI | Swelling and pain | Osteosarcoma | NI | NI | NI | NI | NI | NI | S+RT | Negative | NI | NI | NI | DOC | 96 |
|  |  |  | 9 | M | Mandible | NI | Swelling and pain | Rhabdomyosarcoma | NI | NI | NI | NI | NI | NI | S+RT+CT | Negative | NI | NI | NI | NED | 72 |
|  |  |  | 11 | M | Mandible | NI | Swelling | Rhabdomyosarcoma | NI | NI | NI | NI | NI | NI | S+CT | Negative | NI | NI | NI | NED | 60 |
|  |  |  | 5 | M | Maxilla | NI | Swelling | Osteosarcoma | NI | NI | NI | NI | NI | NI | S+CT | NI | NI | NI | NI | NED | 72 |
| Ruark et al. 1982 | USA | 15 | 45 | F | Maxilla | NI | Nasal obstruction & discharge | Chondrosarcoma | NI | NI | NI | NI | NI | NI | S | Positive | Yes | NI | NI | DOD | 49 |
|  |  |  | 28 | M | Mandible | NI | Chin numbness and painful teeth | Chondrosarcoma | NI | NI | NI | NI | NI | NI | S | Positive | Yes | NI | Yes (Lung) | DOD | 9 |
|  |  |  | 31 | F | Mandible | NI | Painless mass | Chondrosarcoma | NI | NI | NI | NI | NI | NI | S+RT | NI | Yes | NI | Yes (Sternum) | DOD | 28 |
|  |  |  | 26 | M | Maxilla | NI | Painless mass | Chondrosarcoma | NI | NI | NI | NI | NI | NI | S | Negative | No | NI | NI | NED | 420 |
|  |  |  | 29 | F | Maxilla | NI | Nasal obstruction and sinusitis | Chondrosarcoma | NI | NI | NI | NI | NI | NI | S+RT | NI | Yes | NI | NI | DOD | 9 |
|  |  |  | 46 | F | Nasal septum | NI | Nasal obstruction and loss of smell | Chondrosarcoma | NI | NI | NI | NI | NI | NI | S+RT | Positive | Yes | NI | NI | DOD | 73 |
|  |  |  | 56 | F | Maxilla | NI | Nasal obstruction and mass | Chondrosarcoma | NI | NI | NI | NI | NI | NI | S | NI | Yes | NI | NI | DOD | 46 |
|  |  |  | 53 | F | Maxilla | NI | Painless enlargement | Chondrosarcoma | NI | NI | NI | NI | NI | NI | S | Negative | No | NI | NI | NED | 324 |
|  |  |  | 10 | M | Maxilla | NI | Painless mass | Chondrosarcoma | NI | NI | NI | NI | NI | NI | S+RT | NI | Yes | NI | NI | DOD | 23 |
|  |  |  | 44 | M | Maxilla | NI | Painless mass | Chondrosarcoma | NI | NI | NI | NI | NI | NI | S | Negative | No | NI | NI | NED | 300 |
|  |  |  | 18 | M | Maxilla | NI | Painless mass | Chondrosarcoma | NI | NI | NI | NI | NI | NI | S | Negative | No | NI | NI | NED | 240 |
|  |  |  | 36 | M | Maxilla | NI | Enlarging space between incisors | Chondrosarcoma | NI | NI | NI | NI | NI | NI | S | Positive | Yes | NI | NI | NED | 192 |
|  |  |  | 51 | M | Mandible | NI | Painless mass | Chondrosarcoma | NI | NI | NI | NI | NI | NI | S | NI | No | NI | NI | NED | 132 |
|  |  |  | 48 | M | Maxilla | NI | Progressive cheek swelling | Chondrosarcoma | NI | NI | NI | NI | NI | NI | S | NI | Yes | NI | NI | Alive | 120 |
|  |  |  | 18 | M | Maxilla | NI | Painless mass | Chondrosarcoma | NI | NI | NI | NI | NI | NI | S | Negative | No | NI | NI | NED | 84 |
| Dry et al. 2000 | USA | 10 | 31 | M | Maxilla | NI | Swelling | Leiomyosarcoma | NI | NI | NI | NI | NI | NI | S | NI | NI | NI | NI | NED | 61 |
|  |  |  | 58 | M | Maxilla | NI | Pain and swelling | Leiomyosarcoma | NI | NI | NI | NI | NI | NI | S | NI | NI | NI | NI | NED | 55 |
|  |  |  | 88 | F | Floor of mouth | NI | Inspiratory stridor | Leiomyosarcoma | NI | NI | NI | NI | NI | NI | S | NI | NI | NI | NI | DOD | 0.8 |
|  |  |  | 28 | M | Maxilla | NI | Pain | Leiomyosarcoma | NI | NI | NI | NI | NI | NI | S+RT | NI | Yes | NI | NI | DOD | 37 |
|  |  |  | 74 | F | Mandible | NI | Pain and swelling | Leiomyosarcoma | NI | NI | NI | NI | NI | NI | S | NI | NI | NI | NI | DOC | 2 |
|  |  |  | 15 | F | Tongue | NI | NI | Leiomyosarcoma | NI | NI | NI | NI | NI | NI | S+CT | NI | NI | NI | NI | NED | 50 |
|  |  |  | 34 | F | Palate | NI | Pain | Leiomyosarcoma | NI | NI | NI | NI | NI | NI | S+CT | NI | Yes | NI | Yes (Lung) | DOD | 14 |
|  |  |  | 91 | F | Upper lip | NI | Swelling | Leiomyosarcoma | NI | NI | NI | NI | NI | NI | S | NI | NI | Yes | NI | DOC | 46 |
|  |  |  | 27 | F | Mandible | NI | Pain | Leiomyosarcoma | NI | NI | NI | NI | NI | NI | S | NI | NI | NI | NI | NED | 28 |
|  |  |  | NI | M | Gingiva | NI | NI | Leiomyosarcoma | NI | NI | NI | NI | NI | NI | NI | NI | NI | NI | NI | NI | NI |
| Gadwal et al. 2001 | USA | 21 | 7 | F | Mandible | NI | Swelling, tenderness and paresthesias of the lip | Osteosarcoma | NI | NI | NI | NI | III | NI | S+CT | NI | No | No | No | NED | 140 |
|  |  |  | 9 | M | Mandible | NI | Swelling and painless | Osteosarcoma | NI | NI | NI | NI | I | NI | None | NI | No | No | No | DOC | 20 |
|  |  |  | 16 | M | Mandible | NI | Mass and Pain | Osteosarcoma | NI | NI | NI | NI | I | NI | S | NI | No | No | No | NED | 137 |
|  |  |  | 10 | M | Mandible | NI | Mass and paresthesia of lower lip | Osteosarcoma | NI | NI | NI | NI | III | NI | S+RT+CT | NI | Yes | No | No | DOC | 348 |
|  |  |  | 13 | F | Mandible | NI | Mass and malocclusion of teeth | Osteosarcoma | NI | NI | NI | NI | I | NI | CT | NI | No | No | No | NED | 48 |
|  |  |  | 13 | M | Mandible | NI | Swelling | Osteosarcoma | NI | NI | NI | NI | III | NI | S+CT | NI | Yes | No | No | NED | 336 |
|  |  |  | 17 | M | Mandible | NI | Mass | Osteosarcoma | NI | NI | NI | NI | I | NI | S | NI | No | No | No | NED | 90 |
|  |  |  | 9 | M | Mandible | NI | Mass | Osteosarcoma | NI | NI | NI | NI | I | NI | None | NI | No | No | No | NED | 104 |
|  |  |  | 14 | F | Mandible | NI | Mass and loosening of teeth | Osteosarcoma | NI | NI | NI | NI | I | NI | S | NI | No | No | No | NED | 31 |
|  |  |  | 14 | M | Mandible | NI | Mass and weight loss | Osteosarcoma | NI | NI | NI | NI | II | NI | S+CT | NI | Yes | No | No | NED | 51 |
|  |  |  | 4 | F | Mandible | NI | Mass | Osteosarcoma | NI | NI | NI | NI | II | NI | S+RT+CT | NI | Yes | No | No | DOD | 176 |
|  |  |  | 10 | F | Mandible | NI | Mass | Osteosarcoma | NI | NI | NI | NI | II | NI | None | NI | No | No | No | NED | 137 |
|  |  |  | 15 | F | Mandible | NI | Mass | Osteosarcoma | NI | NI | NI | NI | II | NI | S+RT | NI | No | No | No | NED | 350 |
|  |  |  | 15 | F | Maxilla | NI | Mass and loosening of teeth | Osteosarcoma | NI | NI | NI | NI | I | NI | NI | NI | NI | No | No | LFU | NI |
|  |  |  | 17 | F | Mandible | NI | Mass | Osteosarcoma | NI | NI | NI | NI | I | NI | S | NI | Yes | No | No | AWD | 15 |
|  |  |  | 11 | F | Mandible | NI | Swelling and weight loss | Osteosarcoma | NI | NI | NI | NI | III | NI | CT | NI | No | No | No | NED | 244 |
|  |  |  | 17 | M | Mandible | NI | Mass | Osteosarcoma | NI | NI | NI | NI | II | NI | NI | NI | NI | No | No | LFU | NI |
|  |  |  | 18 | M | Mandible | NI | Mass | Osteosarcoma | NI | NI | NI | NI | I | NI | S | NI | No | No | No | NED | 54 |
|  |  |  | 16 | F | Mandible | NI | Swelling | Osteosarcoma | NI | NI | NI | NI | I | NI | S | NI | Yes | No | No | NED | 316 |
|  |  |  | 13 | F | Mandible | NI | Swelling | Osteosarcoma | NI | NI | NI | NI | II | NI | None | NI | No | No | No | DOC | 465 |
|  |  |  | 1 | M | Nasal cavity | NI | Mass and ptosis of eyelid | Osteosarcoma | NI | NI | NI | NI | II | NI | NI | NI | Yes | No | No | LFU | NI |
| Nascimento et al.  2002 | USA | 23 | 36 | M | Tongue | NI | NI | Liposarcoma | NI | NI | NI | NI | NI | NI | NI | NI | NI | NI | NI | NED | 108 |
|  |  |  | 48 | F | Cheek | NI | NI | Liposarcoma | NI | NI | NI | NI | NI | NI | NI | NI | NI | NI | NI | NI | NI |
|  |  |  | 50 | M | Tongue | NI | NI | Liposarcoma | NI | NI | NI | NI | NI | NI | NI | NI | NI | NI | NI | NED | 48 |
|  |  |  | 76 | M | Tongue | NI | NI | Liposarcoma | NI | NI | NI | NI | NI | NI | NI | NI | NI | NI | NI | NED | 14 |
|  |  |  | 47 | F | Tongue | NI | NI | Liposarcoma | NI | NI | NI | NI | NI | NI | NI | NI | NI | NI | NI | NED | 16 |
|  |  |  | 29 | M | Cheek | NI | NI | Liposarcoma | NI | NI | NI | NI | NI | NI | NI | NI | NI | NI | NI | LFU | 5 |
|  |  |  | 74 | M | Tongue | NI | NI | Liposarcoma | NI | NI | NI | NI | NI | NI | NI | NI | Yes | NI | NI | AWD | 17 |
|  |  |  | 77 | F | Tongue | NI | NI | Liposarcoma | NI | NI | NI | NI | NI | NI | NI | NI | NI | NI | NI | NED | 20 |
|  |  |  | 43 | M | Tongue | NI | NI | Liposarcoma | NI | NI | NI | NI | NI | NI | NI | NI | NI | NI | NI | NED | 11 |
|  |  |  | 45 | M | Gingiva | NI | NI | Liposarcoma | NI | NI | NI | NI | NI | NI | NI | NI | NI | NI | NI | NED | 10 |
|  |  |  | 72 | M | Tongue | NI | NI | Liposarcoma | NI | NI | NI | NI | NI | NI | NI | NI | NI | NI | NI | NED | 24 |
|  |  |  | 83 | F | Tongue | NI | NI | Liposarcoma | NI | NI | NI | NI | NI | NI | NI | NI | NI | NI | NI | NI | NI |
|  |  |  | NI | NI | Cheek | NI | NI | Liposarcoma | NI | NI | NI | NI | NI | NI | NI | NI | NI | NI | NI | NI | NI |
|  |  |  | 49 | F | Floor of mouth | NI | NI | Liposarcoma | NI | NI | NI | NI | NI | NI | NI | NI | NI | NI | NI | NED | 66 |
|  |  |  | 80 | M | Tongue | NI | NI | Liposarcoma | NI | NI | NI | NI | NI | NI | NI | NI | NI | NI | NI | LFU | 2 |
|  |  |  | 35 | F | Floor of mouth | NI | NI | Liposarcoma | NI | NI | NI | NI | NI | NI | NI | NI | NI | NI | NI | NED | 25 |
|  |  |  | 49 | F | Cheek | NI | NI | Liposarcoma | NI | NI | NI | NI | NI | NI | NI | NI | NI | NI | NI | NED | 24 |
|  |  |  | 51 | F | Lip | NI | NI | Liposarcoma | NI | NI | NI | NI | NI | NI | NI | NI | NI | NI | NI | LFU | 3 |
|  |  |  | 53 | M | Tongue | NI | NI | Liposarcoma | NI | NI | NI | NI | NI | NI | NI | NI | NI | NI | NI | LFU | 2 |
|  |  |  | 47 | M | Cheek | NI | NI | Liposarcoma | NI | NI | NI | NI | NI | NI | NI | NI | Yes | NI | NI | AWD | 84 |
|  |  |  | 28 | F | Lip | NI | NI | Liposarcoma | NI | NI | NI | NI | NI | NI | NI | NI | NI | NI | NI | LFU | 9 |
|  |  |  | 55 | F | Palate | NI | NI | Liposarcoma | NI | NI | NI | NI | NI | NI | NI | NI | NI | NI | NI | NI | NI |
|  |  |  | 53 | M | Tongue | NI | NI | Liposarcoma | NI | NI | NI | NI | NI | NI | NI | NI | NI | NI | NI | NI | NI |
| Pandey et al. 2003 | India | 16 | 42 | F | Face NOS | NI | NI | Spindle cell sarcoma | NI | T2 | NI | NI | NI | NI | S+CT | NI | Yes | NI | No | AWD | 43 |
|  |  |  | 54 | M | Maxilla | NI | NI | Undifferentiated pleomorphic sarcoma | NI | T2 | NI | NI | NI | NI | S+RT | NI | Yes | NI | No | AWD | 65 |
|  |  |  | 53 | F | Check | NI | NI | Spindle cell sarcoma | NI | NI | NI | NI | NI | NI | S+RT | NI | No | NI | No | NED | 5 |
|  |  |  | 22 | M | Mandible | NI | NI | Spindle cell sarcoma | NI | T2 | NI | NI | NI | NI | S+RT | NI | NI | NI | NI | DOD | 19 |
|  |  |  | 40 | M | Parotid region | NI | NI | Malignant Peripheral Nerve Sheath Tumor | NI | T2 | NI | NI | NI | NI | S+RT | NI | NI | NI | Yes | AWD | 36 |
|  |  |  | 15 | M | Tongue | NI | NI | Spindle cell sarcoma | NI | NI | NI | NI | NI | NI | S+CT | NI | No | NI | No | NED | 44 |
|  |  |  | 16 | F | Check | NI | NI | Angiosarcoma | NI | T2 | NI | NI | NI | NI | S+RT | NI | No | NI | No | NED | 96 |
|  |  |  | 34 | M | Mandible | NI | NI | Liposarcoma | NI | T2 | NI | NI | NI | NI | S+RT | NI | Yes | NI | No | NED | 48 |
|  |  |  | 17 | M | Lip | NI | NI | Rhabdomyosarcoma | NI | T2 | NI | NI | NI | NI | S+RT+CT | NI | No | NI | Yes (Lung) | DOD | 20 |
|  |  |  | 68 | M | Tongue | NI | NI | Spindle cell sarcoma | NI | T1 | NI | NI | NI | NI | S | NI | Yes | NI | No | AWD | 5 |
|  |  |  | 28 | F | Maxilla | NI | NI | Undifferentiated pleomorphic sarcoma | NI | T2 | NI | NI | NI | NI | S | NI | No | NI | No | LFU | 2 |
|  |  |  | 15 | M | Alveolus | NI | NI | Malignant Peripheral Nerve Sheath Tumor | NI | T1 | NI | NI | NI | NI | S | NI | Yes | NI | No | AWD | 14 |
|  |  |  | 54 | F | Face NOS | NI | NI | Liposarcoma | NI | T2 | NI | NI | NI | NI | S+CT | NI | Yes | NI | Yes (Lung) | AWD | 8 |
|  |  |  | 15 | M | Alveolus | NI | NI | Rhabdomyosarcoma | NI | T2 | NI | NI | NI | NI | S+RT+CT | NI | Yes | NI | No | NED | 13 |
|  |  |  | 52 | M | Face NOS | NI | NI | Undifferentiated pleomorphic sarcoma | NI | T2 | NI | NI | NI | NI | S+RT | NI | Yes | NI | No | AWD | 8 |
|  |  |  | 13 | M | Mandible | NI | NI | Undifferentiated pleomorphic sarcoma | NI | T1 | NI | NI | NI | NI | S+RT+CT | NI | No | NI | No | NED | 1 |
| Knott et al. 2003 | USA | 11 | 11 | F | Nasal cavity | NI | Epistaxis and nasal obstruction | Chondrosarcoma | NI | NI | NI | NI | NI | NI | S | NI | No | NI | NI | LFU | NI |
|  |  |  | 15 | F | Maxillary sinus | NI | Epistaxis and nasal obstruction | Chondrosarcoma | NI | NI | NI | NI | NI | NI | S | NI | No | NI | NI | NED | 135 |
|  |  |  | 23 | F | Maxillary sinus | NI | Swelling and pain | Chondrosarcoma | NI | NI | NI | NI | NI | NI | S+RT | NI | No | NI | NI | NED | 264 |
|  |  |  | 24 | M | Nasal cavity | NI | Mass and nasal obstruction | Chondrosarcoma | NI | NI | NI | NI | NI | NI | S+CT | NI | No | NI | NI | NED | 96 |
|  |  |  | 24 | M | Maxillary sinus | NI | Epistaxis | Chondrosarcoma | NI | NI | NI | NI | NI | NI | S | NI | No | NI | NI | DOD | 31 |
|  |  |  | 29 | F | Maxillary sinus | NI | Epistaxis and headache | Chondrosarcoma | NI | NI | NI | NI | NI | NI | S+RT+CT | NI | Yes | NI | NI | NED | 318 |
|  |  |  | 30 | M | Maxillary sinus | NI | Swelling | Chondrosarcoma | NI | NI | NI | NI | NI | NI | S | NI | Yes | NI | NI | DOD | 12 |
|  |  |  | 36 | F | Maxillary sinus | NI | Nasal obstruction and pain | Chondrosarcoma | NI | NI | NI | NI | NI | NI | S | NI | No | NI | NI | NED | 278 |
|  |  |  | 60 | F | Maxillary sinus | NI | Epistaxis and nasal obstruction | Chondrosarcoma | NI | NI | NI | NI | NI | NI | S+CT | NI | Yes | NI | NI | DOD | 20 |
|  |  |  | 75 | M | Nasal cavity | NI | NI | Chondrosarcoma | NI | NI | NI | NI | NI | NI | S | NI | No | NI | NI | LFU | NI |
|  |  |  | 83 | F | Nasal cavity | NI | Nasal obstruction | Chondrosarcoma | NI | NI | NI | NI | NI | NI | S | NI | No | NI | NI | DOD | 4 |
| Yamaguchi et al. 2004 | Japan | 24 | 25 | M | Temporo-mandibular | NI | NI | Osteosarcoma | NI | NI | NI | NI | NI | NI | S+RT+CT | NI | No | No | NI | NED | 85 |
|  |  |  | 34 | F | Maxilla | NI | NI | Osteosarcoma | NI | NI | NI | NI | NI | NI | S | NI | No | No | NI | NED | 114 |
|  |  |  | 40 | F | Mandible | NI | NI | Osteosarcoma | NI | NI | NI | NI | NI | NI | S | NI | Yes | No | NI | NED | 76 |
|  |  |  | 46 | M | Mandible | NI | NI | Osteosarcoma | NI | NI | NI | NI | NI | NI | S | NI | No | No | Yes | DOD | 60 |
|  |  |  | 50 | M | Mandible | NI | NI | Osteosarcoma | NI | NI | NI | NI | NI | NI | S | NI | Yes | No | Yes | AWD | 163 |
|  |  |  | 50 | M | Maxilla | NI | NI | Osteosarcoma | NI | NI | NI | NI | NI | NI | S+CT | NI | No | No | NI | NED | 39 |
|  |  |  | 29 | M | Maxillary sinus | NI | NI | Undifferentiated pleomorphic sarcoma | NI | NI | NI | NI | NI | NI | S+CT | NI | Yes | No | NI | DOD | 16 |
|  |  |  | 43 | M | Mandible | NI | NI | Undifferentiated pleomorphic sarcoma | NI | NI | NI | NI | NI | NI | S | NI | Yes | No | Yes | DOD | 24 |
|  |  |  | 45 | M | Submandibular region | NI | NI | Undifferentiated pleomorphic sarcoma | NI | NI | NI | NI | NI | NI | S+RT | NI | No | No | NI | NED | 97 |
|  |  |  | 56 | M | Maxilla | NI | NI | Undifferentiated pleomorphic sarcoma | NI | NI | NI | NI | NI | NI | S+RT+CT | NI | No | No | NI | NED | 140 |
|  |  |  | 57 | M | Maxillary sinus | NI | NI | Undifferentiated pleomorphic sarcoma | NI | NI | NI | NI | NI | NI | S | NI | Yes | Yes | NI | DOD | 9 |
|  |  |  | 63 | M | Maxillary sinus | NI | NI | Undifferentiated pleomorphic sarcoma | NI | NI | NI | NI | NI | NI | S+RT+CT | NI | No | No | NI | NED | 60 |
|  |  |  | 11 | M | Buccal mucosa | NI | NI | Rhabdomyosarcoma | NI | NI | NI | NI | NI | NI | S | NI | Yes | No | NI | NED | 207 |
|  |  |  | 27 | F | Buccal mucosa | NI | NI | Rhabdomyosarcoma | NI | NI | NI | NI | NI | NI | S+RT+CT | NI | No | Yes | NI | DOD | 18 |
|  |  |  | 55 | M | Maxilla | NI | NI | Rhabdomyosarcoma | NI | NI | NI | NI | NI | NI | S+CT | NI | No | No | NI | DOD | 27 |
|  |  |  | 58 | M | Maxilla | NI | NI | Rhabdomyosarcoma | NI | NI | NI | NI | NI | NI | RT+CT | NI | No | No | Yes | DOD | 4 |
|  |  |  | 77 | M | Mandible | NI | NI | Rhabdomyosarcoma | NI | NI | NI | NI | NI | NI | S | NI | No | No | Yes | NED | 33 |
|  |  |  | 0.4 | F | Mandible | NI | NI | Fibrosarcoma | NI | NI | NI | NI | NI | NI | S+CT | NI | No | Yes | Yes | NED | 117 |
|  |  |  | 10 | F | Mandible | NI | NI | Fibrosarcoma | NI | NI | NI | NI | NI | NI | S | NI | No | No | NI | NED | 138 |
|  |  |  | 16 | F | Mandible | NI | NI | Fibrosarcoma | NI | NI | NI | NI | NI | NI | S | NI | No | No | NI | NED | 228 |
|  |  |  | 43 | M | Mandible | NI | NI | Leiomyosarcoma | NI | NI | NI | NI | NI | NI | S | NI | No | No | NI | AWD | 96 |
|  |  |  | 34 | M | Temporo-mandibular | NI | NI | Angiosarcoma | NI | NI | NI | NI | NI | NI | CT+RT | NI | No | No | Yes | DOD | 48 |
|  |  |  | 53 | M | Maxilla | NI | NI | Angiosarcoma | NI | NI | NI | NI | NI | NI | S+RT | NI | Yes | Yes | NI | DOD | 8 |
|  |  |  | 33 | F | Buccal mucosa | NI | NI | Liposarcoma | NI | NI | NI | NI | NI | NI | S | NI | Yes | No | NI | DOD | 32 |
| Canadian Society of Otolaryngology-Head and Neck Surgery Oncology Study Group 2004 | Canada | 35 | 15 | F | Mandible | NI | NI | Osteosarcoma | NI | NI | NI | NI | NI | NI | S+CT | Negative | No | NI | No | NED | 31 |
|  |  |  | 37 | F | Maxilla | NI | NI | Osteosarcoma | NI | NI | NI | NI | NI | NI | S+RT+CT | Negative | No | NI | No | NED | 34 |
|  |  |  | 62 | F | Mandible | NI | NI | Osteosarcoma | NI | NI | NI | NI | NI | NI | S+RT+CT | Negative | No | NI | No | NED | 110 |
|  |  |  | 36 | F | Mandible | NI | NI | Osteosarcoma | NI | NI | NI | NI | NI | NI | S+CT | Negative | No | NI | No | DOD | 42 |
|  |  |  | 26 | M | Maxilla | NI | NI | Osteosarcoma | NI | NI | NI | NI | NI | NI | S+CT | Negative | Yes | NI | No | DOD | 46 |
|  |  |  | 33 | F | Mandible | NI | NI | Osteosarcoma | NI | NI | NI | NI | NI | NI | S+RT+CT | Positive | Yes | NI | No | DOD | 53 |
|  |  |  | 53 | M | Mandible | NI | NI | Osteosarcoma | NI | NI | NI | NI | NI | NI | S | Negative | No | NI | Yes | NED | 24 |
|  |  |  | 30 | F | Maxilla | NI | NI | Osteosarcoma | NI | NI | NI | NI | NI | NI | S+CT | Negative | No | NI | No | NED | 78 |
|  |  |  | 23 | M | Maxilla | NI | NI | Osteosarcoma | NI | NI | NI | NI | NI | NI | S+CT | Negative | Yes | NI | No | NED | 108 |
|  |  |  | 19 | M | Maxilla | NI | NI | Osteosarcoma | NI | NI | NI | NI | NI | NI | S+CT | Negative | No | NI | No | DOC | 47 |
|  |  |  | 76 | F | Maxilla | NI | NI | Osteosarcoma | NI | NI | NI | NI | NI | NI | S | Negative | Yes | NI | No | DOD | 9 |
|  |  |  | 20 | F | Mandible | NI | NI | Osteosarcoma | NI | NI | NI | NI | NI | NI | S+RT | Negative | No | NI | No | NED | 77 |
|  |  |  | 59 | M | Maxilla | NI | NI | Osteosarcoma | NI | NI | NI | NI | NI | NI | CT | NI | Yes | NI | No | DOD | 34 |
|  |  |  | 35 | M | Maxilla | NI | NI | Osteosarcoma | NI | NI | NI | NI | NI | NI | S+RT | Negative | No | NI | No | NED | 78 |
|  |  |  | 55 | F | Maxilla | NI | NI | Osteosarcoma | NI | NI | NI | NI | NI | NI | S+RT | Negative | Yes | NI | No | DOD | 18 |
|  |  |  | 14 | M | Mandible | NI | NI | Osteosarcoma | NI | NI | NI | NI | NI | NI | S+CT | Negative | No | NI | No | NED | 28 |
|  |  |  | 61 | M | Mandible | NI | NI | Osteosarcoma | NI | NI | NI | NI | NI | NI | S | Positive | Yes | NI | No | DOD | 6 |
|  |  |  | 35 | M | Maxilla | NI | NI | Osteosarcoma | NI | NI | NI | NI | NI | NI | S+CT | Negative | No | NI | No | NED | 57 |
|  |  |  | 65 | F | Mandible | NI | NI | Osteosarcoma | NI | NI | NI | NI | NI | NI | CT | NI | Yes | NI | No | DOD | 3 |
|  |  |  | 25 | F | Mandible | NI | NI | Osteosarcoma | NI | NI | NI | NI | NI | NI | S | Negative | No | NI | Yes | NED | 104 |
|  |  |  | 36 | F | Mandible | NI | NI | Osteosarcoma | NI | NI | NI | NI | NI | NI | S+CT | Negative | No | NI | No | NED | 70 |
|  |  |  | 20 | M | Maxilla | NI | NI | Osteosarcoma | NI | NI | NI | NI | NI | NI | S | Negative | Yes | NI | No | NED | 48 |
|  |  |  | 58 | M | Maxilla | NI | NI | Osteosarcoma | NI | NI | NI | NI | NI | NI | S+RT | Negative | Yes | NI | No | DOC | 118 |
|  |  |  | 64 | F | Maxilla | NI | NI | Osteosarcoma | NI | NI | NI | NI | NI | NI | S+RT+CT | Negative | Yes | NI | No | AWD | 127 |
|  |  |  | 58 | M | Maxilla | NI | NI | Osteosarcoma | NI | NI | NI | NI | NI | NI | S+RT | Negative | No | NI | No | NED | 3 |
|  |  |  | 26 | M | Mandible | NI | NI | Osteosarcoma | NI | NI | NI | NI | NI | NI | S | Negative | No | NI | No | NED | 80 |
|  |  |  | 56 | M | Mandible | NI | NI | Osteosarcoma | NI | NI | NI | NI | NI | NI | S | Positive | Yes | NI | No | DOD | 35 |
|  |  |  | 29 | M | Mandible | NI | NI | Osteosarcoma | NI | NI | NI | NI | NI | NI | S+RT+CT | Positive | No | NI | Yes | AWD | 35 |
|  |  |  | 59 | F | Maxilla | NI | NI | Osteosarcoma | NI | NI | NI | NI | NI | NI | S+RT | Negative | Yes | NI | Yes | DOD | 6 |
|  |  |  | 73 | M | Mandible | NI | NI | Osteosarcoma | NI | NI | NI | NI | NI | NI | S | Positive | Yes | NI | No | DOD | 52 |
|  |  |  | 31 | F | Mandible | NI | NI | Osteosarcoma | NI | NI | NI | NI | NI | NI | CT+RT | NI | Yes | NI | No | DOD | 9 |
|  |  |  | 23 | F | Mandible | NI | NI | Osteosarcoma | NI | NI | NI | NI | NI | NI | S+CT | Positive | Yes | NI | Yes | DOD | 18 |
|  |  |  | 42 | M | Mandible | NI | NI | Osteosarcoma | NI | NI | NI | NI | NI | NI | S+CT | Negative | No | NI | No | DOC | 143 |
|  |  |  | 33 | F | Mandible | NI | NI | Osteosarcoma | NI | NI | NI | NI | NI | NI | S+RT+CT | Positive | No | NI | Yes | DOD | 101 |
|  |  |  | 25 | M | Mandible | NI | NI | Osteosarcoma | NI | NI | NI | NI | NI | NI | S+RT | Negative | No | NI | No | NED | 109 |
| Fernandes et al. 2007 | USA | 16 | Mean 41  Range 14-51 | M6/F10 | Mandible 9 Maxilla 7 | Li-Fraumani Syndrome 1 Polyostotic fibrous dysplasia 1 Previous RT 1 | Swelling 14 Loosening og the teeth 3 Pain 2 Hypoesthesia 1 Garrington's sign 5 | Osteosarcoma | NI | NI | NI | NI | NI | NI | S 10  S+CT 4 NI 2 | NI (16) | Yes 1 | NI | Yes 1 (Lung) | NED 12 DOD 2 LFU 2 | Mean 46 (14-108) |
| Thiele et al. 2008 | Germany | 12 | Mean 42 +/-16  Range 16-68 | M8/F4 | Mandible 5 Maxilla 7 | NI | NI | Osteosarcoma | NI | NI | NI | NI | NI | NI | S 3 S+RT 2 S+CT 7 | NI (12) | Yes 3 | No 12 | No 12 | DOD 2 NED 10 | NI |
| Wang et al. 2009 | Taiwan | 22 | 49 | F | Maxillary sinus | Nasopharyngeal carcinoma Latency 9.5 years | NI | RAS Undifferentiated pleomorphic sarcoma | NI | T1 | N0 | NI | NI | NI | S | Negative | Yes | No | No | DOD | 18 |
|  |  |  | 45 | M | Maxillary sinus | Nasopharyngeal carcinoma Latency 10.9 years | NI | RAS Undifferentiated pleomorphic sarcoma | NI | T2 | N0 | NI | NI | NI | S | Positive | Yes | No | No | DOD | 11 |
|  |  |  | 32 | M | Maxillary sinus | Nasopharyngeal carcinoma Latency 9 years | NI | RAS Undifferentiated pleomorphic sarcoma | NI | T1 | N0 | NI | NI | NI | S | Positive | Yes | No | No | DOD | 28 |
|  |  |  | 62 | F | Maxillary sinus | Nasopharyngeal carcinoma Latency 8.9 years | NI | RAS Undifferentiated pleomorphic sarcoma | NI | T2 | N0 | NI | NI | NI | S | Positive | Yes | No | No | DOD | 30 |
|  |  |  | 48 | M | Maxillary sinus | Nasopharyngeal carcinoma Latency 7.3 years | NI | RAS Undifferentiated pleomorphic sarcoma | NI | T2 | N0 | NI | NI | NI | S | Negative | Yes | No | No | DOD | 44 |
|  |  |  | 43 | M | Maxillary sinus | Nasopharyngeal carcinoma Latency 5.7 years | NI | RAS Undifferentiated pleomorphic sarcoma | NI | T2 | N0 | NI | NI | NI | S | Positive | Yes | No | No | DOD | 12 |
|  |  |  | 70 | M | Maxillary sinus | Nasopharyngeal carcinoma Latency 15 years | NI | RAS Undifferentiated pleomorphic sarcoma | NI | T2 | N0 | NI | NI | NI | S | Positive | Yes | No | No | DOD | 6 |
|  |  |  | 53 | M | Maxillary sinus | Nasopharyngeal carcinoma Latency 14 years | NI | RAS Undifferentiated pleomorphic sarcoma | NI | T2 | N0 | NI | NI | NI | S+RT | Positive | Yes | No | No | DOD | 12 |
|  |  |  | 45 | F | Maxillary sinus | Nasopharyngeal carcinoma Latency 2.25 years | NI | RAS Undifferentiated pleomorphic sarcoma | NI | T1 | N0 | NI | NI | NI | S | Negative | Yes | No | No | DOD | 7 |
|  |  |  | 51 | M | Maxillary sinus | Nasopharyngeal carcinoma Latency 17 years | NI | RAS Undifferentiated pleomorphic sarcoma | NI | T2 | N0 | NI | NI | NI | S | Positive | Yes | No | No | DOD | 2 |
|  |  |  | 54 | F | Maxillary sinus | Nasopharyngeal carcinoma Latency 8.5 years | NI | RAS Undifferentiated pleomorphic sarcoma | NI | T1 | N0 | NI | NI | NI | S | Negative | Yes | No | No | DOD | 18 |
|  |  |  | 43 | M | Maxillary sinus | Nasopharyngeal carcinoma Latency 8.6 years | NI | RAS Undifferentiated pleomorphic sarcoma | NI | T2 | N0 | NI | NI | NI | S+RT | Positive | Yes | No | No | AWD | 112 |
|  |  |  | 37 | F | Maxillary sinus | Nasopharyngeal carcinoma Latency 10 years | NI | RAS Undifferentiated pleomorphic sarcoma | NI | T2 | N0 | NI | NI | NI | S+RT | Positive | Yes | No | No | DOD | 24 |
|  |  |  | 62 | F | Maxillary sinus | Nasopharyngeal carcinoma Latency 9 years | NI | RAS Undifferentiated pleomorphic sarcoma | NI | T2 | N0 | NI | NI | NI | S | Negative | Yes | No | Yes (Brain) | DOD | 30 |
|  |  |  | 59 | F | Maxillary sinus | Nasopharyngeal carcinoma Latency 23.6 years | NI | RAS Undifferentiated pleomorphic sarcoma | NI | T2 | N0 | NI | NI | NI | S | Negative | No | No | Yes (Lung) | DOD | 7 |
|  |  |  | 46 | F | Nasal cavity | NI | NI | Undifferentiated pleomorphic sarcoma | NI | T1 | N0 | NI | NI | NI | S+RT | Negative | No | No | No | NED | 96 |
|  |  |  | 26 | M | Maxillary sinus | NI | NI | Undifferentiated pleomorphic sarcoma | NI | T2 | N0 | NI | NI | NI | S+RT | Negative | No | No | No | NED | 144 |
|  |  |  | 64 | F | Maxillary sinus | NI | NI | Undifferentiated pleomorphic sarcoma | NI | T2 | N0 | NI | NI | NI | S+RT | Positive | Yes | No | No | DOD | 15 |
|  |  |  | 11 | F | Maxillary sinus | NI | NI | Undifferentiated pleomorphic sarcoma | NI | T2 | N0 | NI | NI | NI | S | Negative | No | No | No | NED | 216 |
|  |  |  | 59 | M | Maxillary sinus | NI | NI | Undifferentiated pleomorphic sarcoma | NI | T1 | N0 | NI | NI | NI | S | Negative | No | No | No | NED | 132 |
|  |  |  | 64 | F | Maxillary sinus | NI | NI | Undifferentiated pleomorphic sarcoma | NI | T2 | N0 | NI | NI | NI | S+RT | Positive | Yes | No | No | DOD | 10 |
|  |  |  | 39 | F | Maxillary sinus | NI | NI | Undifferentiated pleomorphic sarcoma | NI | T1 | N0 | NI | NI | NI | S+RT | Negative | No | No | No | NED | 4 |
| Al-Daraji et al. 2009 | Egypt | 26 | 61 | M | Temporo-mandibular | NI | NI | Synovial Sarcoma | NI | NI | NI | NI | NI | NI | NI | NI | NI | NI | NI | DOD | 17 |
|  |  |  | 9 | F | Mandible | NI | NI | Synovial Sarcoma | NI | NI | NI | NI | NI | NI | NI | NI | NI | NI | NI | LFU | NI |
|  |  |  | 72 | F | Check | NI | NI | Synovial Sarcoma | NI | NI | NI | NI | NI | NI | S | NI | NI | NI | NI | NED | 209 |
|  |  |  | 12 | F | Nasolabial fold | NI | NI | Synovial Sarcoma | NI | NI | NI | NI | NI | NI | NI | NI | NI | NI | NI | DOC | 218 |
|  |  |  | 49 | M | Parotid region | NI | NI | Synovial Sarcoma | NI | NI | NI | NI | NI | NI | NI | NI | NI | NI | NI | DOC | 98 |
|  |  |  | 41 | M | Submaxillary region | NI | NI | Synovial Sarcoma | NI | NI | NI | NI | NI | NI | NI | NI | NI | NI | NI | LFU | NI |
|  |  |  | 39 | F | Parotid region | NI | NI | Synovial Sarcoma | NI | NI | NI | NI | NI | NI | NI | NI | NI | NI | NI | DOD | 101 |
|  |  |  | 49 | F | Parotid region | NI | NI | Synovial Sarcoma | NI | NI | NI | NI | NI | NI | NI | NI | NI | NI | NI | LFU | NI |
|  |  |  | 19 | M | Parotid region | NI | NI | Synovial Sarcoma | NI | NI | NI | NI | NI | NI | NI | NI | NI | NI | NI | DOC | 33 |
|  |  |  | 27 | F | Check | NI | NI | Synovial Sarcoma | NI | NI | NI | NI | NI | NI | NI | NI | NI | NI | NI | LFU | NI |
|  |  |  | 85 | F | Parotid region | NI | NI | Synovial Sarcoma | NI | NI | NI | NI | NI | NI | NI | NI | NI | NI | NI | DOC | 80 |
|  |  |  | 49 | M | Parotid region | NI | NI | Synovial Sarcoma | NI | NI | NI | NI | NI | NI | S+RT | NI | NI | NI | NI | NED | 445 |
|  |  |  | 41 | M | Parotid region | NI | NI | Synovial Sarcoma | NI | NI | NI | NI | NI | NI | S+RT | NI | NI | NI | NI | NED | 132 |
|  |  |  | 35 | F | Temporo-mandibular | NI | NI | Synovial Sarcoma | NI | NI | NI | NI | NI | NI | S+RT+CT | NI | NI | NI | NI | NED | 200 |
|  |  |  | 54 | F | Zygomatic area | NI | NI | Synovial Sarcoma | NI | NI | NI | NI | NI | NI | NI | NI | Yes | NI | NI | DOC | 168 |
|  |  |  | 26 | F | Parotid region | NI | NI | Synovial Sarcoma | NI | NI | NI | NI | NI | NI | NI | NI | Yes | NI | NI | NED | 168 |
|  |  |  | 55 | F | Check | NI | NI | Synovial Sarcoma | NI | NI | NI | NI | NI | NI | NI | NI | NI | NI | NI | DOD | 2 |
|  |  |  | 39 | M | Parotid region | NI | NI | Synovial Sarcoma | NI | NI | NI | NI | NI | NI | NI | NI | NI | NI | NI | DOD | 88 |
|  |  |  | 22 | M | Temporo-mandibular | NI | Mass | Synovial Sarcoma | NI | NI | NI | NI | NI | NI | NI | NI | NI | NI | NI | DOD | 8 |
|  |  |  | 64 | M | Parotid region | NI | NI | Synovial Sarcoma | NI | NI | NI | NI | NI | NI | NI | NI | NI | NI | NI | DOC | 8 |
|  |  |  | 23 | F | Parotid region | NI | NI | Synovial Sarcoma | NI | NI | NI | NI | NI | NI | NI | NI | NI | NI | NI | LFU | NI |
|  |  |  | 27 | M | Parotid region | NI | NI | Synovial Sarcoma | NI | NI | NI | NI | NI | NI | NI | NI | NI | NI | NI | DOC | 39 |
|  |  |  | 36 | M | Check | NI | NI | Synovial Sarcoma | NI | NI | NI | NI | NI | NI | NI | NI | NI | NI | NI | DOD | 27 |
|  |  |  | 7 | M | Parotid region | NI | NI | Synovial Sarcoma | NI | NI | NI | NI | NI | NI | NI | NI | NI | NI | NI | DOD | 209 |
|  |  |  | 27 | M | Maxillary area | NI | NI | Synovial Sarcoma | NI | NI | NI | NI | NI | NI | NI | NI | NI | NI | NI | LFU | NI |
|  |  |  | 33 | M | Parotid region | NI | NI | Synovial Sarcoma | NI | NI | NI | NI | NI | NI | NI | NI | NI | NI | NI | DOC | 41 |
| Prado et al. 2009 | Brazil | 13 | 32 | F | Maxilla | NI | Swelling | Chondrosarcoma | NI | NI | NI | NI | NI | NI | S | NI | Yes | NI | NI | DOD | 468 |
|  |  |  | 45 | M | Mandible | NI | Swelling | Chondrosarcoma | NI | NI | NI | NI | NI | NI | S | NI | Yes | NI | NI | DOD | 36 |
|  |  |  | 25 | F | Maxilla | NI | Swelling | Chondrosarcoma | NI | NI | NI | NI | NI | NI | None | NI | NI | NI | NI | DOD | 2 |
|  |  |  | 25 | M | Mandible | NI | Swelling | Chondrosarcoma | NI | NI | NI | NI | NI | NI | RT | NI | NI | NI | NI | DOD | 3 |
|  |  |  | 58 | M | Maxilla | NI | Swelling | Chondrosarcoma | NI | NI | NI | NI | NI | NI | CT | NI | NI | NI | NI | DOD | 12 |
|  |  |  | 37 | M | Mandible | NI | Swelling | Chondrosarcoma | NI | NI | NI | NI | NI | NI | NI | NI | NI | NI | NI | NI | NI |
|  |  |  | 52 | M | Maxilla | NI | Swelling | Chondrosarcoma | NI | NI | NI | NI | NI | NI | S | NI | No | NI | NI | DOC | 72 |
|  |  |  | 38 | M | Mandible | NI | Swelling | Chondrosarcoma | NI | NI | NI | NI | NI | NI | S | NI | No | NI | NI | NED | 144 |
|  |  |  | 47 | F | Maxilla | NI | Swelling | Chondrosarcoma | NI | NI | NI | NI | NI | NI | S+RT | NI | Yes | NI | NI | DOD | 16 |
|  |  |  | 15 | M | Maxilla | NI | Swelling | Chondrosarcoma | NI | NI | NI | NI | NI | NI | S+RT+CT | NI | No | NI | NI | NED | 96 |
|  |  |  | 55 | F | Maxilla | NI | Swelling | Chondrosarcoma | NI | NI | NI | NI | NI | NI | S | NI | No | NI | NI | NED | 60 |
|  |  |  | 22 | F | Nasal fossa | NI | Nasal obstruction | Chondrosarcoma | NI | NI | NI | NI | NI | NI | S+RT | NI | No | NI | NI | NED | 36 |
|  |  |  | 11 | F | Nasal fossa | NI | Nasal obstruction | Chondrosarcoma | NI | NI | NI | NI | NI | NI | S | NI | No | NI | NI | NED | 36 |
| Fyrmpas et al. 2009 | Greece and Germany | 10 | 2 | NI | Nasal cavity | NI | NI | Rhabdomyosarcoma | NI | NI | NI | NI | III | NI | CT+RT | NI | Yes | No | No | DOD | 25 |
|  |  |  | 8 | NI | Nasal cavity | NI | NI | Rhabdomyosarcoma | NI | NI | NI | NI | II | NI | S+RT+CT | Negative | No | No | No | NED | 132 |
|  |  |  | 7 | NI | Nasal cavity | NI | NI | Rhabdomyosarcoma | NI | NI | NI | NI | II | NI | S+RT+CT | NI | No | No | No | NED | 276 |
|  |  |  | 3 | NI | Nasal cavity | NI | NI | Rhabdomyosarcoma | NI | NI | NI | NI | III | NI | CT+RT | NI | No | No | No | NED | 96 |
|  |  |  | 17 | NI | Nasal cavity | NI | NI | Rhabdomyosarcoma | NI | NI | NI | NI | III | NI | CT+RT | NI | Yes | Yes | Yes | DOD | 20 |
|  |  |  | 18 | NI | Maxillary sinus | NI | NI | Rhabdomyosarcoma | NI | NI | NI | NI | II | NI | CT+RT | NI | Yes | No | Yes | DOD | 33 |
|  |  |  | 17 | NI | Nasal cavity | NI | NI | Rhabdomyosarcoma | NI | NI | NI | NI | III | NI | S+RT+CT | Positive | Yes | Yes | No | DOD | 7 |
|  |  |  | 13 | NI | Maxillary sinus | NI | NI | Rhabdomyosarcoma | NI | NI | NI | NI | II | NI | S+RT+CT | Negative | Yes | No | No | NED | 16 |
|  |  |  | 3 | NI | Maxillary sinus | NI | NI | Rhabdomyosarcoma | NI | NI | NI | NI | III | NI | S+CT | Negative | No | No | No | NED | 36 |
|  |  |  | 3 | NI | Nasal cavity | NI | NI | Rhabdomyosarcoma | NI | NI | NI | NI | II | NI | CT | NI | No | No | No | NED | 125 |
| Yan et al.  2010 | China | 20 | 55 | M | Palate | NI | NI | Leiomyosarcoma | NI | NI | NI | NI | NI | NI | S+CT | NI | No | No | No | NED | 20 |
|  |  |  | 33 | F | Palate | NI | NI | Leiomyosarcoma | NI | NI | NI | NI | NI | NI | S | NI | Yes | No | No | DOD | 7 |
|  |  |  | 42 | M | Maxilla | NI | NI | Leiomyosarcoma | NI | NI | NI | NI | NI | NI | S | NI | No | Yes | No | DOD | 6 |
|  |  |  | 11 | F | Cheek | NI | NI | Leiomyosarcoma | NI | NI | NI | NI | NI | NI | S+CT | NI | Yes | No | No | AWD | 20 |
|  |  |  | 40 | M | Mandible | NI | NI | Leiomyosarcoma | NI | NI | NI | NI | NI | NI | S | NI | NI | No | No | LFU | NI |
|  |  |  | 34 | M | Mandible | NI | NI | Leiomyosarcoma | NI | NI | NI | NI | NI | NI | S+RT+CT | NI | No | No | No | NED | 53 |
|  |  |  | 21 | F | Cheek | NI | NI | Leiomyosarcoma | NI | NI | NI | NI | NI | NI | S+RT+CT | NI | No | No | No | NED | 48 |
|  |  |  | 13 | M | Mandible | NI | NI | Leiomyosarcoma | NI | NI | NI | NI | NI | NI | S+RT | NI | Yes | No | No | DOD | 11 |
|  |  |  | 25 | M | Maxilla | NI | NI | Leiomyosarcoma | NI | NI | NI | NI | NI | NI | S | NI | NI | No | No | LFU | NI |
|  |  |  | 16 | F | Cheek | NI | NI | Leiomyosarcoma | NI | NI | NI | NI | NI | NI | S | NI | NI | No | No | LFU | NI |
|  |  |  | 46 | F | Mandible | NI | NI | Leiomyosarcoma | NI | NI | NI | NI | NI | NI | S+RT | NI | Yes | No | No | DOD | 21 |
|  |  |  | 63 | F | Maxilla | NI | NI | Leiomyosarcoma | NI | NI | NI | NI | NI | NI | S | NI | Yes | No | No | DOD | 5 |
|  |  |  | 48 | M | Floor of mouth | NI | NI | Leiomyosarcoma | NI | NI | NI | NI | NI | NI | S | NI | Yes | No | No | DOD | 17 |
|  |  |  | 12 | F | Cheek | NI | NI | Leiomyosarcoma | NI | NI | NI | NI | NI | NI | S+RT | NI | Yes | No | No | DOD | 8 |
|  |  |  | 56 | M | Mandible | NI | NI | Leiomyosarcoma | NI | NI | NI | NI | NI | NI | S | NI | Yes | No | No | DOD | 15 |
|  |  |  | 6 | F | Cheek | NI | NI | Leiomyosarcoma | NI | NI | NI | NI | NI | NI | None | NI | No | No | No | DOD | 3 |
|  |  |  | 47 | F | Mandible | NI | NI | Leiomyosarcoma | NI | NI | NI | NI | NI | NI | S | NI | Yes | No | No | DOD | 16 |
|  |  |  | 19 | M | Mandible | NI | NI | Leiomyosarcoma | NI | NI | NI | NI | NI | NI | S+RT | NI | Yes | No | No | DOD | 35 |
|  |  |  | 52 | M | Mandible | NI | NI | Leiomyosarcoma | NI | NI | NI | NI | NI | NI | S | NI | Yes | No | No | DOD | 11 |
|  |  |  | 49 | M | Maxilla | NI | NI | Leiomyosarcoma | NI | NI | NI | NI | NI | NI | S | NI | Yes | No | No | DOD | 12 |
| Luna-Ortiz et al. 2010 | Mexico | 21 | 75 | M | Maxilla | NI | Swelling 10   Pain 8  Gingival tumor 7   hard palate tumor 4 gingival ulceration in 4  bleeding 2  proptosis 2 paresthesia/ dysesthesia V2 2  dysphagia 2  weight loss 2  nasal cavity tumor 1  teeth mobility 1  trismus 1 epiphora 1 | Osteosarcoma | NI | NI | NI | NI | NI | NI | S+RT | Positive | NI | NI | NI | NED | 24 |
|  |  |  | 28 | M | Maxilla | NI |  | Osteosarcoma | NI | NI | NI | NI | NI | NI | CT+RT | Positive | NI | NI | NI | LFU | 2 |
|  |  |  | 28 | F | Maxilla | NI |  | Osteosarcoma | NI | NI | NI | NI | NI | NI | S+CT | Negative | Yes | NI | NI | LFU | 33 |
|  |  |  | 16 | F | Maxilla | NI |  | Osteosarcoma | NI | NI | NI | NI | NI | NI | RT | Negative | Yes | NI | NI | LFU | 21 |
|  |  |  | 49 | F | Maxilla | NI |  |  | NI | NI | NI | NI | NI | NI | S+CT | Negative | NI | NI | NI | LFU | 12 |
|  |  |  | 32 | M | Maxilla | NI |  | Osteosarcoma | NI | NI | NI | NI | NI | NI | S | Positive | NI | NI | NI | NED | 134 |
|  |  |  | 39 | F | Maxilla | NI |  | Osteosarcoma | NI | NI | NI | NI | NI | NI | S+RT | NI | Yes | NI | NI | LFU | 34 |
|  |  |  | 23 | M | Maxilla | NI |  | Osteosarcoma | NI | NI | NI | NI | NI | NI | S+RT | Positive | Yes | NI | NI | LFU | 90 |
|  |  |  | 25 | M | Maxilla | NI |  | Osteosarcoma | NI | NI | NI | NI | NI | NI | S+RT | Positive | NI | NI | NI | NED | 45 |
|  |  |  | 49 | F | Maxilla | NI |  | Osteosarcoma | NI | NI | NI | NI | NI | NI | S+RT | Positive | NI | NI | NI | NED | 9 |
|  |  |  | 32 | F | Maxilla | NI |  | Osteosarcoma | NI | NI | NI | NI | NI | NI | S+RT | Positive | Yes | NI | NI | LFU | 17 |
|  |  |  | 63 | M | Maxilla | NI |  | Osteosarcoma | NI | NI | NI | NI | NI | NI | S+RT | Positive | Yes | NI | NI | LFU | 17 |
|  |  |  | 17 | M | Maxilla | NI |  | Osteosarcoma | NI | NI | NI | NI | NI | NI | S+RT | Positive | Yes | NI | NI | LFU | 17 |
|  |  |  | 31 | M | Maxilla | NI |  | Osteosarcoma | NI | NI | NI | NI | NI | NI | S+RT+CT | Negative | Yes | NI | NI | DOD | 29 |
|  |  |  | 40 | M | Maxilla | NI |  | Osteosarcoma | NI | NI | NI | NI | NI | NI | S+RT | Positive | NI | NI | NI | LFU | 10 |
|  |  |  | 76 | F | Maxilla | NI |  | Osteosarcoma | NI | NI | NI | NI | NI | NI | S+RT | Negative | NI | NI | NI | NED | 44 |
|  |  |  | 28 | F | Maxilla | NI |  | Osteosarcoma | NI | NI | NI | NI | NI | NI | S+RT+CT | Negative | NI | NI | NI | NED | 44 |
|  |  |  | 16 | F | Maxilla | NI |  | Osteosarcoma | NI | NI | NI | NI | NI | NI | S+RT+CT | Negative | NI | NI | NI | NED | 43 |
|  |  |  | 29 | F | Maxilla | NI |  | Osteosarcoma | NI | NI | NI | NI | NI | NI | S+RT+CT | Negative | NI | NI | NI | NED | 21 |
|  |  |  | 28 | M | Maxilla | NI |  | Osteosarcoma | NI | NI | NI | NI | NI | NI | S | Negative | Yes | NI | NI | NED | 72 |
|  |  |  | 66 | F | Maxilla | NI |  | Osteosarcoma | NI | NI | NI | NI | NI | NI | S+RT | Negative | NI | NI | NI | NED | 2 |
| Debnam et al. 2012 | USA | 11 | 34 | F | Mandible | Melanoma  Latency NI | NI | RAS Osteosarcoma | NI | NI | NI | NI | NI | NI | CT | NI | NI | NI | NI | Dead | 9 |
|  |  |  | 45 | M | Mandible | Hodgkin lymphoma  Latency 9 years | NI | RAS Osteosarcoma | NI | NI | NI | NI | NI | NI | S+CT | NI | NI | NI | NI | Dead | 13 |
|  |  |  | 50 | M | Maxilla | Squamous cell  Latency NI | NI | RAS Osteosarcoma | NI | NI | NI | NI | NI | NI | S | NI | NI | NI | NI | Alive | 68 |
|  |  |  | 48 | M | Mandible | Squamous cell   Latency 57.6 | NI | RAS Osteosarcoma | NI | NI | NI | NI | NI | NI | S+CT | NI | NI | NI | NI | Dead | 14 |
|  |  |  | 52 | M | Maxilla | Basal cell  Latency 25 years | NI | RAS Osteosarcoma | NI | NI | NI | NI | NI | NI | S | NI | NI | NI | NI | Alive | 77 |
|  |  |  | 50 | F | Mandible | Adenoid cystic  Latency 20 years | NI | RAS Osteosarcoma | NI | NI | NI | NI | NI | NI | CT | NI | NI | NI | NI | Dead | 9 |
|  |  |  | 67 | M | Mandible | Squamous cell  Latency 12.8 years | NI | RAS Osteosarcoma | NI | NI | NI | NI | NI | NI | S | NI | NI | NI | NI | Alive | 16 |
|  |  |  | 74 | M | Mandible | Squamous cell  Latency NI | NI | RAS Osteosarcoma | NI | NI | NI | NI | NI | NI | S | NI | NI | NI | NI | Alive | 3 |
|  |  |  | 83 | F | Right maxillary mass | Squamous cell  Latency NI | NI | RAS Osteosarcoma | NI | NI | NI | NI | NI | NI | NI | NI | NI | NI | NI | Dead | 12 |
|  |  |  | 65 | M | Retromolar trigone | Squamous cell  Latency 10 years | NI | RAS Spindle cell sarcoma | NI | NI | NI | NI | NI | NI | CT | NI | NI | NI | NI | Alive | 16 |
|  |  |  | 47 | F | Retromolar trigone | Mucoepidermoid tumor   Latency NI | NI | RAS Osteosarcoma | NI | NI | NI | NI | NI | NI | S | NI | NI | NI | NI | Dead | 14 |
| Santamaría et al.  2012 | Spain | 12 | Median 30.5 | M8/F4 | Mandible 12 | NI | NI | Ewing's sarcoma 2  Chondrosarcoma 2 Osteosarcoma 4 Low-grade sarcoma 1 Synovial sarcoma 1 Rhabdomyosarcoma 2 | NI | NI | NI | NI | NI | NI | S+RT 2 S+CT 2 S+RT+CT 7 S 1 | Negative 10 Positive 2 | NI | NI | Yes 2 | NED 5  Dead 5 AWD 2 | 45.25 (6-76) |
| Mazeron et al. 2014 | France | 16 | 3 | M | Nasolabial fold | NI | NI | Rhabdomyosarcoma | NI | NI | NI | NI | NI | IRS: II | S+RT+CT | NI | NI | NI | NI | Alive | 33 |
|  |  |  | 3 | M | Nasolabial fold | NI | NI | Rhabdomyosarcoma | NI | NI | NI | NI | NI | III | CT+RT | NI | NI | NI | NI | Alive | 30.3 |
|  |  |  | 2 | F | Nasolabial fold | NI | NI | Rhabdomyosarcoma | NI | NI | NI | NI | NI | III | CT+RT | NI | NI | NI | NI | Alive | 24.5 |
|  |  |  | 1 | F | Nasolabial fold | NI | NI | Rhabdomyosarcoma | NI | NI | NI | NI | NI | III | CT+RT | NI | NI | Yes | NI | Alive | 26.3 |
|  |  |  | 1 | M | Nasolabial fold | NI | NI | Rhabdomyosarcoma | NI | NI | NI | NI | NI | III | S+RT+CT | NI | Yes | Yes | NI | Dead | 1.7 |
|  |  |  | 2 | M | Nasolabial fold | NI | NI | Rhabdomyosarcoma | NI | NI | NI | NI | NI | III | S+RT+CT | NI | NI | Yes | Yes | Dead | 2 |
|  |  |  | 1 | M | Nasolabial fold | NI | NI | Rhabdomyosarcoma | NI | NI | NI | NI | NI | III | S+RT+CT | NI | NI | NI | NI | Alive | 11 |
|  |  |  | 13 | M | Nasolabial fold | NI | NI | Rhabdomyosarcoma | NI | NI | NI | NI | NI | III | S+RT+CT | NI | Yes | Yes | NI | Dead | 2.8 |
|  |  |  | 13 | F | Nasolabial fold | NI | NI | Rhabdomyosarcoma | NI | NI | NI | NI | NI | III | S+RT+CT | NI | NI | NI | NI | Alive | 8.3 |
|  |  |  | 0.3 | F | Nasolabial fold | NI | NI | Rhabdomyosarcoma | NI | NI | NI | NI | NI | III | S+RT+CT | NI | Yes | NI | NI | Dead | 3.3 |
|  |  |  | 1 | F | Nasolabial fold | NI | NI | Rhabdomyosarcoma | NI | NI | NI | NI | NI | III | S+RT+CT | NI | NI | NI | Yes | Dead | 2.3 |
|  |  |  | 0.3 | M | Nasolabial fold | NI | NI | Rhabdomyosarcoma | NI | NI | NI | NI | NI | II | CT+RT | NI | NI | Yes | NI | Dead | 3.7 |
|  |  |  | 6 | F | Nasolabial fold | NI | NI | Rhabdomyosarcoma | NI | NI | NI | NI | NI | III | S+RT+CT | NI | NI | NI | NI | Alive | 8.9 |
|  |  |  | 6 | F | Nasolabial fold | NI | NI | Rhabdomyosarcoma | NI | NI | NI | NI | NI | III | S+RT+CT | NI | NI | Yes | NI | Dead | 2.1 |
|  |  |  | 1 | F | Nasolabial fold | NI | NI | Rhabdomyosarcoma | NI | NI | NI | NI | NI | III | S+RT+CT | NI | Yes | NI | NI | Dead | 1.8 |
|  |  |  | 1 | M | Nasolabial fold | NI | NI | Rhabdomyosarcoma | NI | NI | NI | NI | NI | III | S+RT+CT | NI | NI | NI | NI | Alive | 6.4 |
| Qureshi et al. 2014 | India | 11 | <10 6 >10 5  Median 10 Range 5-16 | M7/F4 | Mandible 6 Maxilla 5 | NI | NI | Ewing’s sarcoma | NI | NI | NI | NI | NI | NI | S+CT 11 | Negative 10 Positive 1 | Yes 1 | NI | 1 (Bone) | NED 9 AWD 1 DOD 1 | Median 63 (12-109) |
| Liu et al.  2015 | China | 15 | Mean 35 Median 38 Range 14-62 | M5/F10 | Mandible 8 Maxilla 7 | NI | Swelling 12 Numbness 6 Pain 5 Trismus 5 Tooth motility 3 | Synovial Sarcoma | NI | NI | NI | NI | IIA 10 IIB 4 IVB 1 | NI | S 15 RT 9 CT 6 | Positive 3 Negative 12 | Yes 6 | Yes 1 | Yes 1 (Lung) | DOD 4 Alive 11 | Mean 42 (12-90) |
| Quereshi et al. 2016 | India | 21 | ≤12 11  >12 10 Median 11.6 Range 5-17 | M11/F10 | Mandible 8 Maxilla 13 | NI | NI | Ewing’s Sarcoma | NI | NI | NI | NI | NI | NI | CT 21  S 17 RT 4 | Negative 14 Positive 3 | NI | NI | NI | NED 14 AWD 1 DOD 6 | Mean 36 (7-123m) |
| Zhu et al. 2016 | China | 15 | 50 | F | Maxilla | Nasopharyngeal carcinoma Latency 7 years | Mass 13 Trismus 9 Pain 7 Osteoradionecrosis 5 Numbness 2 Bleeding 2 Ocular signs 2 Facial paralysis 1 | RAS Fibrosarcoma | NI | NI | NI | NI | NI | NI | S | NI | Yes | No | NI | LFU | NI |
|  |  |  | 55 | F | Maxilla | Nasopharyngeal carcinoma Latency 5 years |  | RAS Fibrosarcoma | NI | NI | NI | NI | NI | NI | S | NI | Yes | No | NI | DOD | 25 |
|  |  |  | 31 | F | Mandible | Nasopharyngeal carcinoma Latency 13 years |  | RAS Fibrosarcoma | NI | NI | NI | NI | NI | NI | S | NI | NI | No | NI | LFU | NI |
|  |  |  | 26 | M | Mandible | Nasopharyngeal carcinoma Latency 12 years |  | RAS Osteosarcoma | NI | NI | NI | NI | NI | NI | S | NI | NI | No | NI | LFU | NI |
|  |  |  | 43 | F | Maxilla | Malignant teratoma Latency 12 years |  | RAS Fibrosarcoma | NI | NI | NI | NI | NI | NI | S+RT+CT | NI | NI | No | NI | NED | 80 |
|  |  |  | 10 | M | Mandible | Nasopharyngeal carcinoma Latency 5 years |  | RAS Fibrosarcoma | NI | NI | NI | NI | NI | NI | S | NI | NI | No | NI | NED | 77 |
|  |  |  | 41 | F | Mandible | Non-Hodgkin lymphoma Latency 4 years |  | RAS Fibrosarcoma | NI | NI | NI | NI | NI | NI | S | NI | Yes | No | NI | NED | 67 |
|  |  |  | 35 | M | Maxilla | Nasopharyngeal carcinoma Latency 9 years |  | RAS Osteosarcoma | NI | NI | NI | NI | NI | NI | S | NI | NI | No | NI | DOD | 12 |
|  |  |  | 42 | M | Maxilla | Nasopharyngeal carcinoma Latency 3 years |  | RAS Fibrosarcoma | NI | NI | NI | NI | NI | NI | S+RT | NI | NI | No | NI | DOC | 11 |
|  |  |  | 54 | M | Mandible | Nasopharyngeal carcinoma Latency 10 years |  | RAS Undifferentiated pleomorphic sarcoma | NI | NI | NI | NI | NI | NI | S | NI | NI | No | NI | DOD | 5 |
|  |  |  | 56 | M | Mandible | Nasopharyngeal carcinoma Latency 8 years |  | RAS Osteosarcoma | NI | NI | NI | NI | NI | NI | S | NI | Yes | No | NI | DOD | 10 |
|  |  |  | 32 | M | Mandible | Nasopharyngeal carcinoma Latency 10 years |  | RAS Osteosarcoma | NI | NI | NI | NI | NI | NI | S+RT | NI | NI | No | NI | NED | 7 |
|  |  |  | 57 | F | Mandible | Nasopharyngeal carcinoma Latency 20 years |  | RAS Osteosarcoma | NI | NI | NI | NI | NI | NI | S | NI | Yes | No | NI | AWD | 8 |
|  |  |  | 46 | F | Mandible | Nasopharyngeal carcinoma Latency 21 years |  | RAS Osteosarcoma | NI | NI | NI | NI | NI | NI | S | NI | NI | No | NI | NED | 5 |
|  |  |  | 51 | F | Maxilla | Nasopharyngeal carcinoma Latency 12 years |  | RAS Osteosarcoma | NI | NI | NI | NI | NI | NI | S | NI | Yes | No | NI | DOD | 7 |
| Liao et al. 2016 | China | 45 | ≤49 21   >49 24  Median 49 Range 18-69 | M33/F12 | Mandible 12 Maxilla 33 | Nasopharyngeal carcinoma Median latency 8 years  (3–34 years) ≤8 23  >8 22 | NI | RAS Osteosarcoma | NI | NI | NI | M1 2 | I/II 19 III/IV 26 | NI | S 30 S+CT 8 S+RT+CT 1 CT 6 | Positive 24 Negative 15 | Yes 10 | NI | NI | DOD 45 | Mean 17.9 (2.1-56.5) |
| Seng et al. 2019 | China | 55 | ≤32 19   >32 16 Mean 32.5 Range 14-66 | M35/F20 | Mandible 55 | No | Swelling 55 Pain 10 Lower lip numbness 6 Trismus 3 Cachexia 15 | Osteosarcoma | NI | NI | NI | NI | IA 11 IB 12 IIA 21 IIB 8 NI 3 | NI | S+CT 50 S+RT+CT 5 | Positive 5 | Yes 11 | NI | Yes 8 | LFU 8 DOD 12 Alive 35 | Mean 63.3 (10-163) |
| Bouaoud et al. 2019 | France | 35 | Median 36.8 Range 18.5-84.4 | M23/F12 | Mandible 21 Maxilla 14 | NI | Evolving mass syndrome 29 Pain 6 | Osteosarcoma | NI | NI | NI | NI | IIA 24 IIB 2 IVA 4 IVB 3 NI 2 | NI | S 31  CT 40  RT 8 | Negative 17  Positive 10   NI | Yes 8 | NI | Yes 3 | Dead 8 Alive 27 | Median 43.6 (1-160.7) |
| Kumar et al. 2019 | India | 26 | 18 | M | Mandible | Trauma 2 | NI | Osteosarcoma | NI | NI | NI | NI | NI | NI | S+CT | NI | NI | NI | NI | Alive | 90 |
|  |  |  | 65 | M | Mandible |  | NI | Osteosarcoma | NI | NI | NI | NI | NI | NI | S+CT | NI | Yes | NI | NI | Dead | 19 |
|  |  |  | 21 | M | Maxilla |  | NI | Osteosarcoma | NI | NI | NI | NI | NI | NI | S | NI | NI | NI | NI | Alive | 38 |
|  |  |  | 22 | M | Maxilla |  | NI | Osteosarcoma | NI | NI | NI | NI | NI | NI | S+RT+CT | NI | NI | NI | NI | Alive | 61 |
|  |  |  | 60 | M | Maxilla |  | NI | Osteosarcoma | NI | NI | NI | NI | NI | NI | S+CT | NI | NI | NI | NI | Dead | 26 |
|  |  |  | 18 | M | Maxilla |  | NI | Osteosarcoma | NI | NI | NI | NI | NI | NI | S | NI | NI | NI | NI | Dead | 12 |
|  |  |  | 14 | F | Maxilla |  | NI | Osteosarcoma | NI | NI | NI | NI | NI | NI | S | NI | NI | NI | NI | Alive | 11 |
|  |  |  | 17 | M | Maxilla |  | NI | Ewing’s Sarcoma | NI | NI | NI | NI | NI | NI | RT+CT | NI | NI | NI | NI | Alive | 119 |
|  |  |  | 30 | M | Mandible |  | NI | Ewing’s Sarcoma | NI | NI | NI | NI | NI | NI | S+CT | NI | Yes | NI | NI | Alive | 120 |
|  |  |  | 18 | M | Mandible |  | NI | Ewing’s Sarcoma | NI | NI | NI | NI | NI | NI | S+CT | NI | NI | NI | NI | Alive | 54 |
|  |  |  | 4 | M | Mandible |  | NI | Ewing’s Sarcoma | NI | NI | NI | NI | NI | NI | S+RT+CT | NI | NI | NI | NI | Alive | 33 |
|  |  |  | 15 | F | Mandible |  | NI | Ewing’s Sarcoma | NI | NI | NI | NI | NI | NI | S+CT | NI | NI | NI | NI | Alive | 50 |
|  |  |  | 75 | M | Maxilla |  | NI | Chondrosarcoma | NI | NI | NI | NI | NI | NI | NI | NI | NI | NI | NI | Dead | 25 |
|  |  |  | 40 | M | Mandible |  | NI | Chondrosarcoma | NI | NI | NI | NI | NI | NI | NI | NI | NI | NI | NI | LFU | NI |
|  |  |  | 21 | M | Buccal Vestibule |  | NI | Chondrosarcoma | NI | NI | NI | NI | NI | NI | S+RT | NI | Yes | NI | NI | Alive | 78 |
|  |  |  | 21 | F | Maxilla |  | NI | Leiomyosarcoma | NI | NI | NI | NI | NI | NI | S+RT | NI | Yes | NI | NI | Dead | 24 |
|  |  |  | 20 | F | Mandible |  | NI | Leiomyosarcoma | NI | NI | NI | NI | NI | NI | S | NI | NI | NI | NI | Alive | 12 |
|  |  |  | 17 | M | Gingiva |  | NI | Leiomyosarcoma | NI | NI | NI | NI | NI | NI | S | NI | NI | NI | NI | LFU | NI |
|  |  |  | 55 | M | Maxillary Alveolus |  | NI | Undifferentiated pleomorphic sarcoma | NI | NI | NI | NI | NI | NI | CT | NI | NI | NI | NI | Dead | 8 |
|  |  |  | 20 | M | Maxilla |  | NI | Undifferentiated pleomorphic sarcoma | NI | NI | NI | NI | NI | NI | RT+CT | NI | NI | NI | NI | Dead | 23 |
|  |  |  | 59 | M | Maxillary Tuberosity |  | NI | Rhabdomyosarcoma | NI | NI | NI | NI | NI | NI | S+RT+CT | NI | NI | NI | NI | Alive | 59 |
|  |  |  | 23 | F | Buccal Mucosa |  | NI | Rhabdomyosarcoma | NI | NI | NI | NI | NI | NI | CT | NI | NI | NI | NI | Alive | 13 |
|  |  |  | 38 | F | Mandible |  | NI | Malignant Peripheral Nerve Sheath Tumor | NI | NI | NI | NI | NI | NI | S+RT | NI | NI | NI | NI | Alive | 18 |
|  |  |  | 60 | F | Maxilla |  | NI | Malignant Peripheral Nerve Sheath Tumor | NI | NI | NI | NI | NI | NI | S+RT+CT | NI | NI | NI | NI | Alive | 25 |
|  |  |  | 28 | M | Mandible |  | NI | Myeloid Sarcoma | NI | NI | NI | NI | NI | NI | CT | NI | NI | NI | NI | Alive | 30 |
|  |  |  | 5 | M | Mandible |  | NI | Myeloid Sarcoma | NI | NI | NI | NI | NI | NI | CT | NI | NI | NI | NI | Alive | 28 |
| Cassoni et al.  2020 | Italy | 15 | Mean 38.53  +/- 18.91 | M4/F11 | Mandible 7 Maxilla 8 | NI | NI | Osteosarcoma 12  RAS Osteosarcoma 3 | NI | NI | NI | NI | IA 2 IIA 11 IIB 1 | NI | S 8 Multimodality 7 | Negative 12 Positive 2 Marginal resection 1 | Yes 4 | NI | NI | Dead 3 NI 12 | Mean 7.46 +/- 4.59 |
| You et al.  2021 | China | 10 | 45 | M | Gingiva | Previous RT: no | NI | Undifferentiated pleomorphic sarcoma | NI | T4 | N0 | M0 | IV | NI | S | NI | Yes | NI | No | DOD | 9 |
|  |  |  | 42 | M | Tongue | Previous RT: yes Squamous cell carcinoma | NI | Undifferentiated pleomorphic sarcoma | NI | T3 | N0 | M0 | III | NI | S+CT/Target therapy | NI | Yes | NI | No | DOD | 5 |
|  |  |  | 46 | F | Gingiva | Previous RT: no | NI | Undifferentiated pleomorphic sarcoma | NI | T4 | N0 | M0 | IV | NI | S+RT+CT/Target therapy | NI | No | NI | Yes (Lung) | DOD | 18 |
|  |  |  | 63 | F | Gingiva | Previous RT: no | NI | Undifferentiated pleomorphic sarcoma | NI | T4 | N0 | M0 | IV | NI | S+RT | NI | Yes | NI | No | DOD | 5 |
|  |  |  | 39 | M | Tongue | Previous RT: no | NI | Undifferentiated pleomorphic sarcoma | NI | T4 | N0 | M0 | IV | NI | S+RT+CT/Target therapy | NI | Yes | NI | No | DOD | 5 |
|  |  |  | 79 | F | Buccal area | Previous RT: no | NI | Undifferentiated pleomorphic sarcoma | The molecular features were demonstrated by using whole exonic sequencing for 1 included case. Cancer driver gene detection revealed GBP4 as a candidate driver gene for the primary oralmaxillary UPS. Additionally. a missense mutation in gene PIK3CA (p.E545K) was also identified. | T4 | N0 | M0 | IV | NI | S | NI | No | NI | Yes (Lung) | DOD | 6.5 |
|  |  |  | 49 | M | Tongue | Previous RT: yes Squamous cell carcinoma | NI | Undifferentiated pleomorphic sarcoma | NI | T4 | N0 | M0 | IV | NI | S+RT+CT/Target therapy | NI | Yes | NI | No | DOD | 6 |
|  |  |  | 20 | F | Palate | Previous RT: no | NI | Undifferentiated pleomorphic sarcoma | NI | T4 | N0 | M0 | IV | NI | S+RT+CT/Target therapy | NI | Yes | NI | No | DOD | 17 |
|  |  |  | 59 | M | Gingiva | Previous RT: no | NI | Undifferentiated pleomorphic sarcoma | NI | T4 | N0 | M0 | IV | NI | S+RT+CT/Target therapy | NI | Yes | NI | No | DOD | 12 |
|  |  |  | 40 | F | Maxillary sinus | Previous RT: no | NI | Undifferentiated pleomorphic sarcoma | NI | T4 | N0 | M0 | IV | NI | S+RT+CT/Target therapy | NI | Yes | NI | Yes (Lung) | DOD | 17 |
| Kotecha et al. 2021 | UK | 14 | 16 | M | Mandible | NI | NI | Osteosarcoma | NI | NI | NI | NI | NI | NI | S+RT | Negative | Yes | No | No | NED | 479 |
|  |  |  | 69 | M | Mandible | NI | NI | Osteosarcoma | NI | NI | NI | NI | NI | NI | S+RT+CT | Negative | No | NI | Yes | DOD | 7 |
|  |  |  | 47 | F | Maxilla | NI | NI | Chondrosarcoma | NI | NI | NI | NI | NI | NI | NI | NI | No | No | No | NED | 228 |
|  |  |  | 75 | F | Mandible | NI | NI | Osteosarcoma | NI | NI | NI | NI | NI | NI | S+RT | Negative | No | No | No | NED | 35 |
|  |  |  | 63 | F | Maxilla | NI | NI | Chondrosarcoma | NI | NI | NI | NI | NI | NI | S+RT | Positive | No | No | No | NED | 33 |
|  |  |  | 72 | M | Mandible | NI | NI | Osteosarcoma | NI | NI | NI | NI | NI | NI | S+CT | Positive | Yes | No | No | DOD | 12 |
|  |  |  | 60 | M | Oral cavity NOS | NI | NI | Liposarcoma | NI | NI | NI | NI | NI | NI | RT+CT | NI | No | No | No | NED | 33 |
|  |  |  | 21 | M | Maxilla | NI | NI | Chondrosarcoma | NI | NI | NI | NI | NI | NI | S+CT | Positive | No | No | No | NED | 92 |
|  |  |  | 67 | F | Maxilla | NI | NI | Osteosarcoma | NI | NI | NI | NI | NI | NI | RT+CT | NI | No | No | No | NED | 25 |
|  |  |  | 28 | M | Maxilla | NI | NI | Chondrosarcoma | NI | NI | NI | NI | NI | NI | RT+CT | Negative | Yes | NI | Yes | NED | 40 |
|  |  |  | 88 | M | Face NOS | NI | NI | Dermatofibrosarcoma protuberans | NI | NI | NI | NI | NI | NI | S+RT+CT | Positive | Yes | No | No | NED | 114 |
|  |  |  | 71 | F | Face NOS | NI | NI | Angiosarcoma | NI | NI | NI | NI | NI | NI | S+RT+CT | Positive | Yes | NI | Yes | DOD | 44 |
|  |  |  | 76 | F | Mandible | NI | NI | Osteosarcoma | NI | NI | NI | NI | NI | NI | RT+CT | NI | No | No | No | DOD | 11 |
|  |  |  | 50 | M | Maxilla | NI | NI | Chondrosarcoma | NI | NI | NI | NI | NI | NI | NI | NI | No | No | No | DOD | 10 |
| Zhang et al. 2024 | China | 21 | 0.8 | F | Nasolabial fold | NI | NI | Rhabdomyosarcoma | PAX3/7FOXO1 gene fusion positive in 14 patients and negative in 3 | T1a | N1 | M0 | III | IRS: III | S+RT+CT | NI | Yes | NI | NI | Alive | 42.5 |
|  |  |  | 9 | F | Nasolabial fold | NI | NI | Rhabdomyosarcoma |  | T1a | N0 | M0 | I | III | S+RT+CT | NI | NI | NI | NI | Alive | 10.9 |
|  |  |  | 0.8 | F | Nasolabial fold | NI | NI | Rhabdomyosarcoma |  | T1a | N0 | M0 | I | III | S+RT+CT | NI | NI | NI | NI | Alive | 20.6 |
|  |  |  | 4 | M | Nasolabial fold | NI | NI | Rhabdomyosarcoma |  | T1a | N1 | M0 | III | III | S+RT+CT | NI | NI | Yes | NI | Alive | 64.1 |
|  |  |  | 1 | M | Nasolabial fold | NI | NI | Rhabdomyosarcoma |  | T2b | N1 | M0 | III | III | S+RT+CT | NI | NI | NI | NI | Alive | 45.2 |
|  |  |  | 3 | M | Nasolabial fold | NI | NI | Rhabdomyosarcoma |  | T1a | N0 | M0 | I | III | S+RT+CT | NI | NI | NI | NI | Alive | 58.1 |
|  |  |  | 1 | F | Nasolabial fold | NI | NI | Rhabdomyosarcoma |  | T1a | N0 | M0 | I | III | S+RT+CT | NI | NI | Yes | NI | Alive | 24.8 |
|  |  |  | 0.6 | M | Nasolabial fold | NI | NI | Rhabdomyosarcoma |  | T2b | N0 | M0 | II | III | S+RT+CT | NI | NI | NI | NI | Alive | 18.1 |
|  |  |  | 3 | M | Nasolabial fold | NI | NI | Rhabdomyosarcoma |  | T2b | N1 | M0 | III | III | S+RT+CT | NI | NI | Yes | Yes (Zygomatic and paerietal bones) | Dead | 32.5 |
|  |  |  | 5 | M | Nasolabial fold | NI | NI | Rhabdomyosarcoma |  | T1a | N0 | M0 | I | III | S+RT+CT | NI | NI | NI | NI | Alive | 26.6 |
|  |  |  | 3 | M | Nasolabial fold | NI | NI | Rhabdomyosarcoma |  | T2b | N1 | M0 | III | III | S+RT+CT | NI | NI | NI | NI | Alive | 78.3 |
|  |  |  | 0.7 | M | Nasolabial fold | NI | NI | Rhabdomyosarcoma |  | T1a | N0 | M0 | I | III | S+RT+CT | NI | NI | NI | NI | Alive | 20.6 |
|  |  |  | 2 | M | Nasolabial fold | NI | NI | Rhabdomyosarcoma |  | T2a | N1 | M0 | III | III | S+RT+CT | NI | NI | NI | NI | Alive | 14.9 |
|  |  |  | 1 | F | Nasolabial fold | NI | NI | Rhabdomyosarcoma |  | T1a | N0 | M0 | I | II | S+RT+CT | NI | NI | NI | NI | Alive | 15.6 |
|  |  |  | 1 | F | Nasolabial fold | NI | NI | Rhabdomyosarcoma |  | T2a | N1 | M0 | III | III | S+RT+CT | NI | NI | Yes | NI | Alive | 22.7 |
|  |  |  | 10 | M | Nasolabial fold | NI | NI | Rhabdomyosarcoma |  | T1a | N0 | M0 | I | III | S+RT+CT | NI | NI | NI | NI | Alive | 12.7 |
|  |  |  | 1 | F | Nasolabial fold | NI | NI | Rhabdomyosarcoma |  | T1a | N0 | M0 | I | II | S+RT+CT | NI | Yes | NI | NI | Alive | 34.3 |
|  |  |  | 3 | M | Nasolabial fold | NI | NI | Rhabdomyosarcoma |  | T1a | N1 | M1 | IV | IV | S+RT+CT | NI | NI | NI | Yes (Zygomatic and paerietal bones) | Dead | 24.5 |
|  |  |  | 4 | F | Nasolabial fold | NI | NI | Rhabdomyosarcoma |  | T2a | N1 | M0 | III | III | S+RT+CT | NI | Yes | NI | NI | Dead | 13.8 |
|  |  |  | 1 | F | Nasolabial fold | NI | NI | Rhabdomyosarcoma |  | T2a | N0 | M0 | II | III | S+RT+CT | NI | Yes | NI | NI | Dead | 23.9 |
|  |  |  | 10 | M | Nasolabial fold | NI | NI | Rhabdomyosarcoma |  | T2a | N1 | M0 | III | III | S+RT+CT | NI | NI | NI | NI | Alive | 66 |
| Cattan et al. 2024 | Brazil | 37 | 33 | F | Mandible | NI | Painful lesion and paresthesia | Ewing’s Sarcoma | NI | NI | NI | NI | NI | NI | S+RT | Negative | NI | NI | NI | NED | 252 |
|  |  |  | 32 | M | Gingiva | NI | Bledding lesion | Sindle cell sarcoma | NI | NI | NI | NI | NI | NI | NI | NI | NI | NI | NI | NI | NI |
|  |  |  | 10 | M | Nasal cavity | NI | Mass | Chondrosarcoma | NI | NI | NI | NI | NI | NI | S | NI | Yes | NI | NI | NED | 168 |
|  |  |  | 33 | M | Mandible | NI | NI | Osteosarcoma | NI | NI | NI | NI | NI | NI | S | NI | NI | NI | NI | NED | 6 |
|  |  |  | 15 | M | Temporomandibular joint | NI | Facial paralysis | Chondrosarcoma | NI | NI | NI | NI | NI | NI | S+RT | NI | Yes | NI | NI | NI | NI |
|  |  |  | 79 | M | Parotid gland | NI | Painfull mass | Angiosarcoma | NI | NI | NI | NI | NI | NI | S+RT | NI | NI | NI | NI | NED | 216 |
|  |  |  | 15 | M | Maxillary sinus | NI | Bledding and painful mass | Osteosarcoma | NI | NI | NI | NI | NI | NI | S+CT | Positive | Yes | NI | Yes | DOD | 25 |
|  |  |  | 45 | M | Oral mucosa | NI | Bledding lesion | Kaposi´s sarcoma | NI | NI | NI | NI | NI | NI | NI | NI | NI | NI | NI | NED | 213 |
|  |  |  | 50 | M | Parotid gland | NI | Mass | Liposarcoma | NI | NI | NI | NI | NI | NI | S+RT | Positive | NI | NI | NI | NED | 204 |
|  |  |  | 20 | F | Mouth | NI | Mass | Alveolar sarcoma | NI | NI | NI | NI | NI | NI | S+RT | Positive | NI | NI | Yes | NED | 210 |
|  |  |  | 11 | F | Nasal cavity | NI | Expansive lesion | Rabdomyosarcoma | NI | NI | NI | NI | NI | NI | NI | NI | NI | NI | NI | NI | NI |
|  |  |  | 64 | F | Nasal cavity | NI | NI | Chondrosarcoma | NI | NI | NI | NI | NI | NI | S+RT | Positive | NI | NI | NI | NED | 162 |
|  |  |  | 43 | M | Maxillary sinus | NI | NI | Osteosarcoma | NI | NI | NI | NI | NI | NI | S | Positive | Yes | NI | NI | DOD | 4 |
|  |  |  | 47 | M | Nasal cavity | NI | Mass causing nasal obstruction | Rabdomyosarcoma | NI | NI | NI | NI | NI | NI | S+CT | NI | NI | NI | NI | DOD | 4 |
|  |  |  | 81 | F | Mandible | NI | Painful mass | Osteosarcoma | NI | NI | NI | NI | NI | NI | CT | NI | NI | NI | NI | DOD | 4 |
|  |  |  | 15 | F | Nasal cavity | NI | Mass | Rabdomyosarcoma | NI | NI | NI | NI | NI | NI | RT+CT | NI | NI | NI | NI | NI | NI |
|  |  |  | 14 | M | Mandible | NI | Mass | Rabdomyosarcoma | NI | NI | NI | NI | NI | NI | S+CT | Negative | NI | NI | NI | NED | 24 |
|  |  |  | 19 | M | Nasal cavity | NI | Mass causing nasal obstruction | Rabdomyosarcoma | NI | NI | NI | NI | NI | NI | S+RT+CT | NI | NI | NI | Yes | DOD | 16 |
|  |  |  | 35 | F | Gingiva | NI | NI | Kaposi´s sarcoma | NI | NI | NI | NI | NI | NI | Antiretroviral medication | NI | NI | NI | NI | NED | 87 |
|  |  |  | 43 | M | Nasal cavity | NI | Lesion and loss of vision | Chondrosarcoma | NI | NI | NI | NI | NI | NI | S+RT | NI | NI | NI | NI | NED | 94 |
|  |  |  | 14 | F | Nasal cavity | NI | Mass causing nasal obstruction | Rabdomyosarcoma | NI | NI | NI | NI | NI | NI | S+RT+CT | NI | NI | NI | NI | DOD | 10 |
|  |  |  | 17 | M | Oral mucosa | NI | Painful mass | Spindle cell sarcoma | NI | NI | NI | NI | NI | NI | S+RT+CT | Positive | NI | NI | NI | NED | 86 |
|  |  |  | 39 | F | Mandible | NI | Lesion | Chondrosarcoma | NI | NI | NI | NI | NI | NI | S | Negative | NI | NI | NI | NED | 66 |
|  |  |  | 72 | F | Nasal cavity | NI | Mass causing nasal obstruction | Granulocytic sarcoma | NI | NI | NI | NI | NI | NI | RT+CT | NI | NI | NI | NI | AWD | 15 |
|  |  |  | 25 | M | Mandible | NI | NI | Osteosarcoma | NI | NI | NI | NI | NI | NI | S+RT+CT | NI | Yes | NI | NI | DOD | 62 |
|  |  |  | 50 | F | Maxillary sinus | NI | Mass causing nasal obstruction and loss of weight | Leiomyosarcoma | NI | NI | NI | NI | NI | NI | S+RT | Positive | NI | NI | NI | NED | 58 |
|  |  |  | 50 | F | Nasal cavity | NI | Mass causing nasal obstruction | Chondrosarcoma | NI | NI | NI | NI | NI | NI | S | Negative | NI | NI | NI | NED | 54 |
|  |  |  | 51 | M | Nasal cavity | NI | Lesion | Leiomyosarcoma | NI | NI | NI | NI | NI | NI | S | NI | NI | NI | NI | NED | 36 |
|  |  |  | 51 | M | Nasal cavity | NI | Painful mass | Angiosarcoma | NI | NI | NI | NI | NI | NI | S+RT | Positive | NI | NI | NI | NED | 37 |
|  |  |  | 59 | M | Nasal cavity | NI | Mass causing nasal obstruction | Biphenotypic sinonasal sarcoma | NI | NI | NI | NI | NI | NI | S+RT+CT | Positive | Yes | NI | NI | NED | 32 |
|  |  |  | 37 | F | Maxilla | NI | Lesion | Dermatofibrosarcoma protuberans | NI | NI | NI | NI | NI | NI | S+RT | Positive | NI | NI | NI | NED | 27 |
|  |  |  | 40 | F | Mandible | NI | Mass | Chondrosarcoma | NI | NI | NI | NI | NI | NI | RT+CT | NI | NI | NI | NI | NED | 12 |
|  |  |  | 36 | F | Nasal cavity | NI | Mass causing nasal obstruction | Pleomorphic sarcoma | NI | NI | NI | NI | NI | NI | S+RT+CT | NI | Yes | NI | NI | NED | 21 |
|  |  |  | 43 | M | Maxilla | NI | Hard nodule | Dermatofibrosarcoma protuberans | NI | NI | NI | NI | NI | NI | S+RT | Positive | NI | NI | NI | NED | 16 |
|  |  |  | 37 | M | Maxillary sinus | NI | Painfull mass | Osteosarcoma | NI | NI | NI | NI | NI | NI | S+RT+CT | NI | Yes | NI | NI | NED | 3 |
|  |  |  | 18 | F | Nasal cavity | NI | Mass causing nasal obstruction | Rabdomyosarcoma | NI | NI | NI | NI | NI | NI | S+CT | NI | NI | NI | NI | NED | 11 |
|  |  |  | 25 | M | Mouth | NI | Erithematous lesion | Kaposi´s sarcoma | NI | NI | NI | NI | NI | NI | NI | NI | NI | NI | NI | NED | NI |

*Note:* N: number of cases; F: female; M: male; NI: not informed; RT: radiotherapy; CT: chemotherapy; NED: no evidence of disease; AWD: alive with disease; LFU: lost follow-up; DOD: died of disease; DOC: died of other cause; RAS: radiation-associated sarcoma.

**Figure S2** Summary of the risk of bias in cross-sectional studies, assessed using the Joanna Briggs Institute Critical Appraisal Checklist.


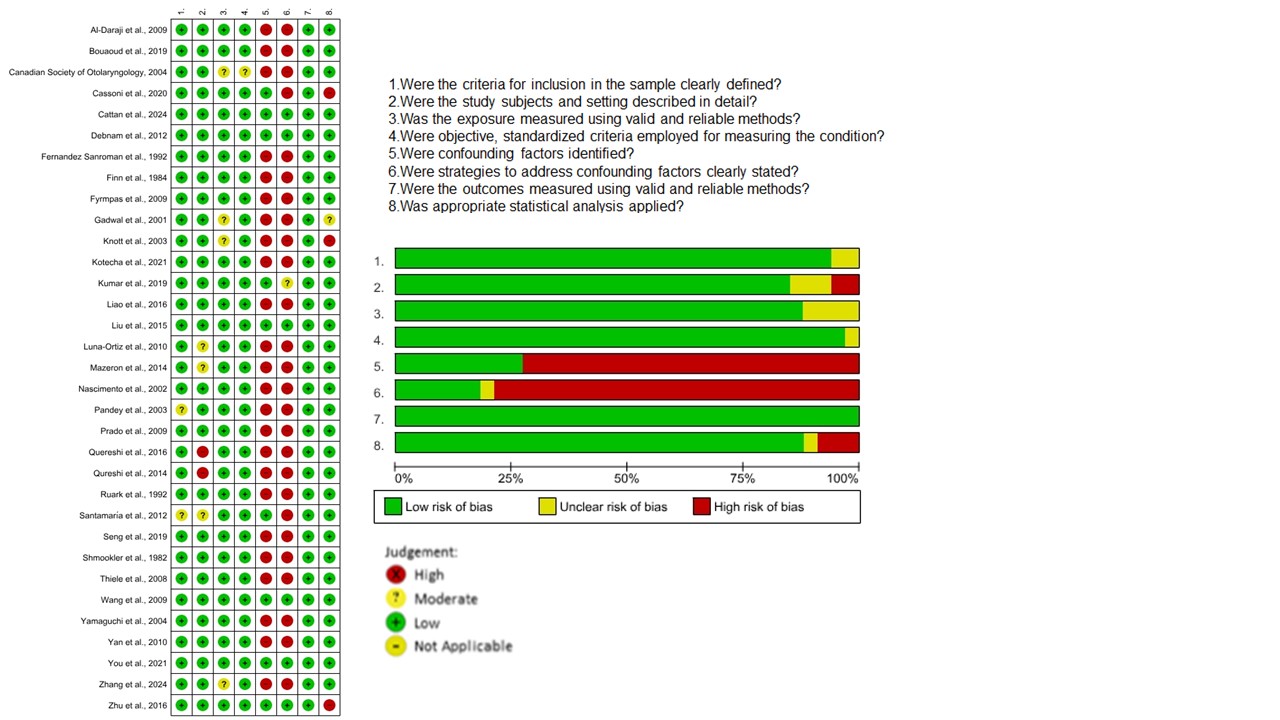


**Figure S3** Summary of the risk of bias in case report study, assessed using the Joanna Briggs Institute Critical Appraisal Checklist.
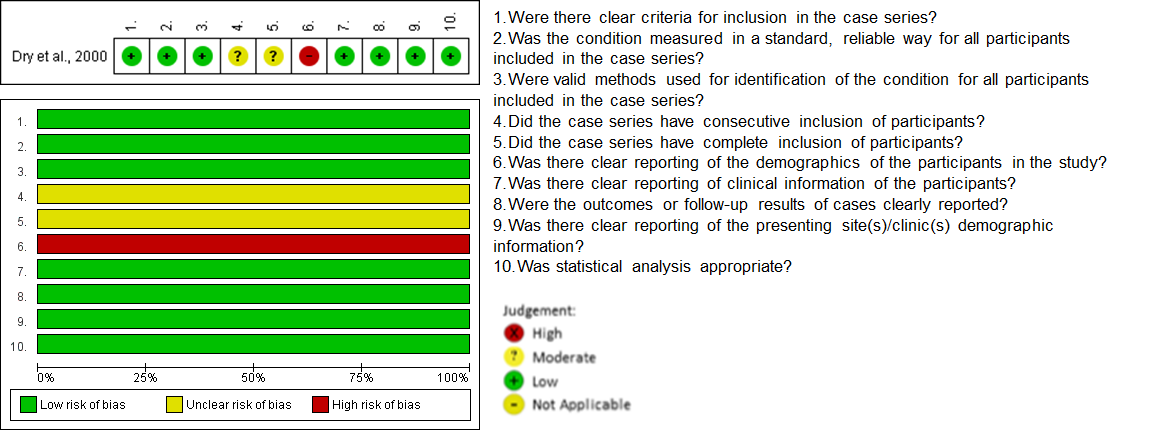


**Figure S4** Overall Survival (OS) curves. (a) Kaplan-Meyer curve demonstrating the OS of patients affected by oral and maxillofacial sarcomas. Using Log-Rank univariate analysis, (b) age (*P*<0.0001), (c) histological type (*P*<0,0001), (d) T classification (*P*<0.0001), (e) N classification (*P*=0.0314), (f) stage grouping (*P*=0.0003), (g) margin status (*P*<0.0001), (h) local recurrence (*P*<0.0001), (i) nodal metastasis (*P*=0.0012) and (j) distant metastasis (*P*=0.0021) significantly impact the survival rate of oral and maxillofacial sarcoma.


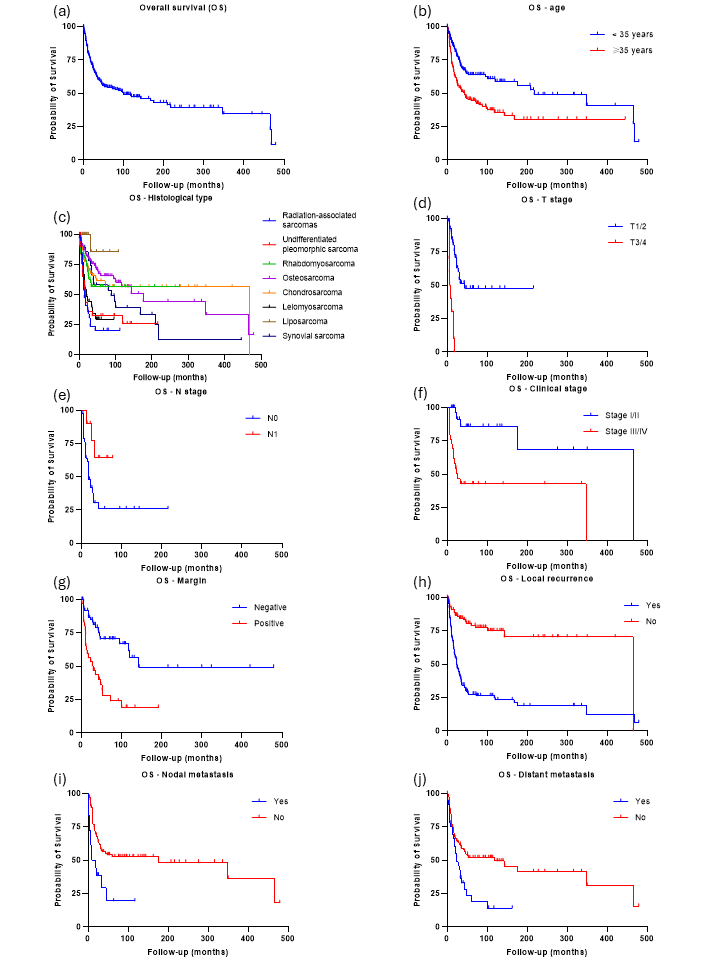


**Table S5** Clinical and Tumor Characteristics Influencing Disease-Specific Survival in Oral and Maxillofacial Sarcomas: Univariate Cox Analysis

|  | **HR (95% CI)** | **p-value** |
| --- | --- | --- |
| **Sex** | | |
| Male | 1 | 0.594 |
| Female | 1.107 (0.761 – 1.611) |  |
| **Age** | | |
| < 35 | 0.372 (0.251 – 0.551) | **<0.001** |
| ≥ 35 | 1 |  |
| **Anatomical Location** | | |
| Soft tissue | 1 | 0.788 |
| Intraosseous | 0.951 (0.657 – 1.375) |  |
| **Histological Subtype** | | |
| Soft tissue | 0.373 (0.216–0.643) | **<0.001** |
| Bone | 0.081 (0.161-0.493) |  |
| RAS | 1 |  |
| **T Stage** | | |
| T1/T2 | 0.162 (0.072 – 0.366) | **<0.001** |
| T3/T4 | 1 |  |
| **N Stage** | | |
| N0 | 29.299 (0.477 – 1798.258) | 0.108 |
| N1 | 1 |  |
| **Clinical Stage** | | |
| I/II | 0.108 (0.024 – 0.481) | **0.003** |
| III/IV | 1 |  |
| **Treatment** | | |
| CT and/or RT | 1 |  |
| Surgery | 0.854 (0.451 – 1.619) | 0.629 |
| Multimodal | 0.574 (0.305 – 1.082) | 0.086 |
| **Margins status** | | |
| Positive | 1 | **<0.001** |
| Negative | 0.270 (0.142 – 0.514) |  |
| **Local Recurrence** | | |
| Yes | 1 | **<0.001** |
| No | 0.182 (0.109 – 0.304) |  |
| **Nodal Metastasis** | | |
| Yes | 1 | 0.525 |
| No | 0.758 (0.322 – 1.782) |  |
| **Distant Metastasis** |  |  |
| Yes | 1 | **0.029** |
| No | 0.575 (0.350 – 0.945) |  |

*Note:* S: Surgery, RT: Radiation Therapy, CT: Chemotherapy, HR:Hazard Ratio, RAS: radiation-associated sarcoma; CI:Confidence Interval. Reference categories are indicated by 1 in the corresponding row.
